# Deuterated reagents in multicomponent reactions to afford deuterium-labeled products

**DOI:** 10.3762/bjoc.20.195

**Published:** 2024-09-06

**Authors:** Kevin Schofield, Shayna Maddern, Yueteng Zhang, Grace E Mastin, Rachel Knight, Wei Wang, James Galligan, Christopher Hulme

**Affiliations:** 1 Department of Chemistry and Biochemistry, College of Science, University of Arizona, Tucson, Arizona, 85721, USA; 2 Department of Pharmacology and Toxicology, University of Arizona, Tucson, Arizona, 85721, USA

**Keywords:** deuterated aldehydes, deuterated formamides, deuterated isocyanides, DHPs, kinetic isotope effect, Leuckart–Wallach, microsomal stability, multicomponent reactions

## Abstract

The utility of bio-isosteres is broad in drug discovery and methodology herein enables the preparation of deuterium-labeled products is the most fundamental of known bio-isosteric replacements. As such we report the use of both [D_1_]-aldehydes and [D_2_]-isonitriles across 8 multicomponent reactions (MCRs) to give diverse arrays of deuterated products. A highlight is the synthesis of several FDA-approved calcium channel blockers, selectively deuterated at a *t*_1/2_ limiting metabolic soft-spot via use of [D_1_]-aldehydes. Surrogate pharmacokinetic analyses of microsomal stability confirm prolongation of *t*_1/2_ of the new deuterated analogs. We also report the first preparation of [D_2_]-isonitriles from [D_3_]-formamides via a modified Leuckart–Wallach reaction and their use in an MCR to afford products with [D_2_]-benzylic positions and likely significantly enhanced metabolic stability, a key parameter for property-based design efforts.

## Introduction

Multicomponent reactions (MCRs) are one-pot reactions that utilize three or more readily available starting materials [1–4]. Typically, MCRs use reactive functional groups such as ketones or aldehydes, carboxylic acids, amines, and isocyanides where these simple building blocks can be utilized to form large libraries of drug-like compounds with synthetic ease [5–6]. In recent years use of deuterium in drug discovery has expanded beyond mechanistic and tracer studies to deuterium incorporation in small molecules in attempts to hijack the deuterium kinetic isotope effect to induce longer drug *t*_1/2_ and greater systemic exposure [7–9]. Herein, we describe applications of deuterium-labeled reagents with MCRs through use of deuterated aldehydes and deuterated isocyanides, an area of study with sparingly few examples.

**Scheme 1:**
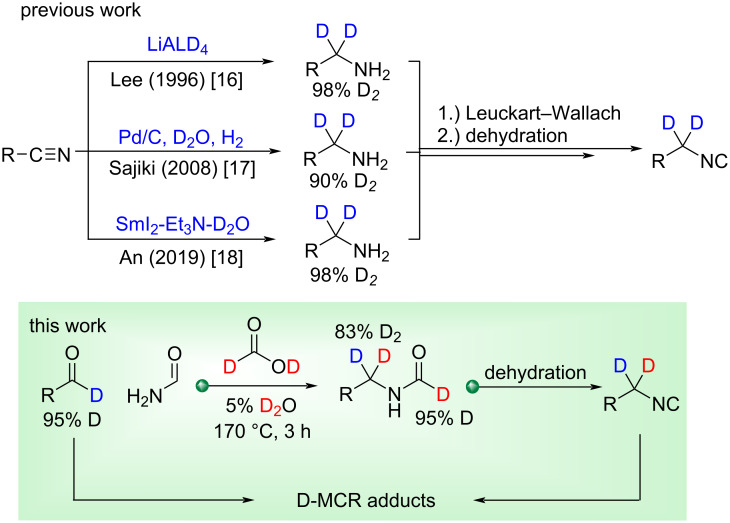
Competitive examples of D_2_-benzylamine formation via phenyl-nitriles.

One example by Srivastava obtained a 65% deuterated Passerini product starting from a 65% deuterated aldehyde [10]. Latterly, Yamamoto utilized a 90% deuterated [D_2_]-isocyanide in a copper catalyzed [3 + 2] cycloaddition to afford a 60% deuterated [D_2_]-pyrrole [11]. The utility of the Leuckart–Wallach reaction towards the generation of isocyanides was first explored by Dömling [12], yet the use of such reagents in MCRs and determination of discrepancies in deuterium retention with MCRs has yet to be explored, although one would expect scrambling to be limited. Thus, we began by gathering highly deuterated aldehydes (>95% D) prepared via NHC catalysis [13] and developed a route to deuterated [D_2_]-benzylic isocyanides with a goal to apply them to the field of isocyanide multicomponent reaction (IMCR) chemistry which enables rapid access to arrays of biologically relevant chemotypes or secondary reactions thereafter [14–15]. Many of these chemotypes have populated corporate collections through in-house production or external purchase and have progressed along the value chain to the clinic and full approval [5]. Literature inspection reveals that an established common method to prepare deuterated benzylic isonitriles is reduction of a nitrile in the presence of a deuterium source (Scheme 1) [16–18].

## Results and Discussion

We hypothesized that [D_1_]-aldehydes could be converted to [D_2_]-benzylic isocyanides using [D_2_]-formic acid via Leuckart–Wallach reaction followed by dehydration. Surprisingly, the Leuckart–Wallach reaction gave [D_3_]-formamides which are scarce in the primary literature. The common method to prepare [D_1_]-formamides (D–C=O) is through a Leuckart–Wallach reaction with an amine and [D_1_]-methyl/ethyl formate or [D_1_]-dimethylformamide [19–20]. Stockmann and co-workers produced [D_2_]-formamides (N–D, D–C=O) via acid-catalyzed nitrile hydrolysis with HCl and D_2_O [21]. Thus, using the Leuckart–Wallach methodology developed herein, deuterated aldehydes can be converted into [D_2_]-isocyanides. The optimized conditions for this reaction are summarized below (Table 1).

**Table 1:** Optimization of deuterated Leuckart–Wallach reaction^a^.

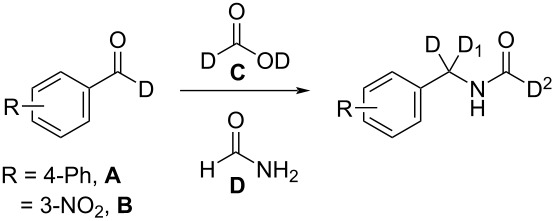					

Entry	Aldehyde	C equiv	D equiv	Yield %	Deuteration% D^1^, D^2^

1	**A**	7.2	12	61	51, 42
2	**A**	6	1	24	70, 88
3	**A**	20	1	27	83, 94
4	**A**	12	10	80	27, 61
**5** ^b^	**B**	**20**	**2**	**83**	**78, 40**

^a^Standard conditions: 170 °C, 3 h. ^b^Reaction was heated to 180 °C for 5 minutes in a microwave reactor. D installed in product from D-aldehyde (**A or B**), D_1_ and D_2_ come from **C**. % Deuteration was determined via ^1^H NMR.

It is important to note that 1 equivalent of formamide and excess [D_2_]-formic acid (Table 1, entry 2) leads to increased deuteration of formamide product while increasing formamide equivalents increases the yield at the cost of deuterated %. Excess reaction time increases side product formation and thermal degradation of the aldehyde starting material. To combat this, microwave irradiation was employed which dramatically increased the overall yield of the reaction (Table 1, entry 5). In summary, minimal formamide (1–2 equiv), excess [D_2_]-formic acid, and heating to 170 °C in a microwave reactor for 5 minutes is expected to give excellent yields in good deuteration %.

**Scheme 2:**
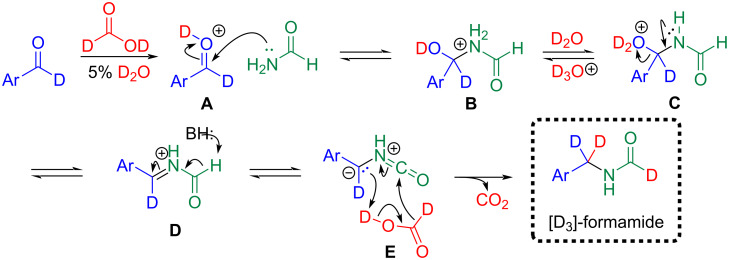
Proposed tentative mechanism of [D_3_]-formamide formation via modified Leuckart–Wallach reaction with [D_2_]-formic acid.

A tentative mechanism to [D_3_]-formamides is shown in Scheme 2 but is open to debate. Thus, formamide adds to the [D_1_]-aldehyde **A** to form hemiaminal **B** which eliminates D_2_O to give imine **D**. Deprotonation of formamide **D** forms the resonance and zwitterrion-stabilized isocyanate **E** [22]. We then hypothesize that zwitterion **E** rearranges with loss of CO_2_ to form [D_3_]-formamide.

The ability to deuterate at benzylic positions is particularly relevant as benzylic C–H bonds are common in biologically relevant chemotypes and moreover appear in approximately 25% of the top selling 200 pharmaceuticals [23]. Benzyl cation stability is a driver of metabolism at these sites where benzylic C–H bonds readily undergo metabolism driven by cytochrome P450 oxidases via single-electron oxidation [24]. This metabolic lability may be tempered by hydrogen replacement with deuterium, an almost perfect bio-isosteric replacement (C–H to C–D) which maintains 3D surface, shape and flexibility [8]. Indeed, early incorporation of deuterium during hit generation may negate the need for late-stage C–H functionalization which often requires strong external oxidants or affords products with significantly lower biological activity [25–27]. Thus, eight MCRs were evaluated for D-reagent scope of reactivity and determination of deuterium retention using a combination of deuterated aldehydes, [D_1_]*-*, and/or [D_2_]-isocyanides.

**Scheme 3:**
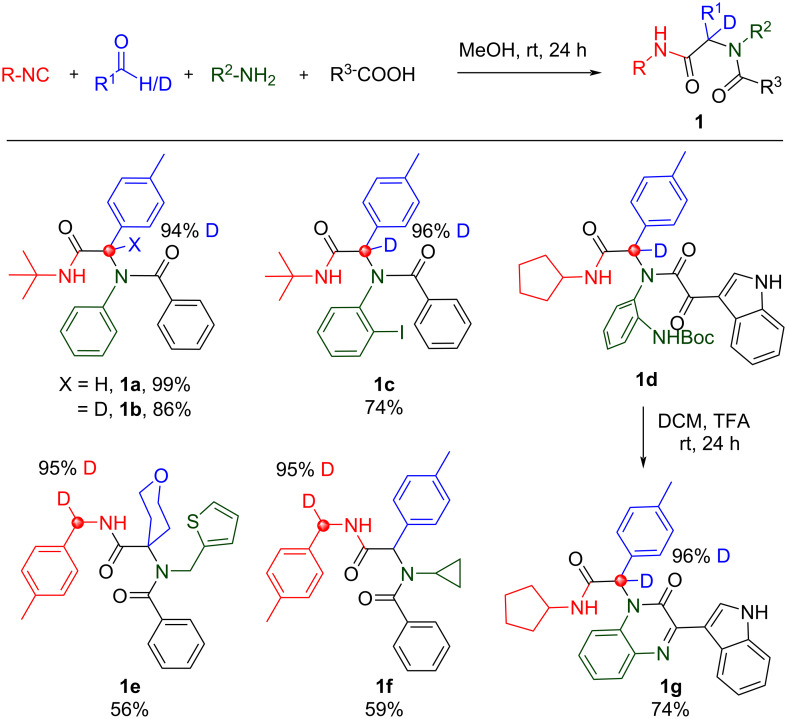
Ugi-4CR products: no deuterium scrambling observed.

We began with the venerable Ugi 4-component reaction (Ugi-4CR), first reported by Ivar Ugi in 1959 [28]. The Ugi-4CR utilizes an amine, carbonyl, carboxylic acid, and isocyanide component to afford α-aminoacyl amide derivatives **1a**–**g** in good yield (Scheme 3). A [D_1_]-aldehyde and [D_1_]-isocyanide were independently used in conjunction with supporting reagents to afford **1b**–**d** and **1e** and **1f**, respectively, with no observation of deuterium scrambling. A post-condensation modification of **1d**, representative of the large swath of chemical space accessible by Ugi-deprotect-cyclize (UDC) methodology, gave the dihydroquinoxaline **1g** in good yield with high deuterium retention [29–32].

**Scheme 4:**
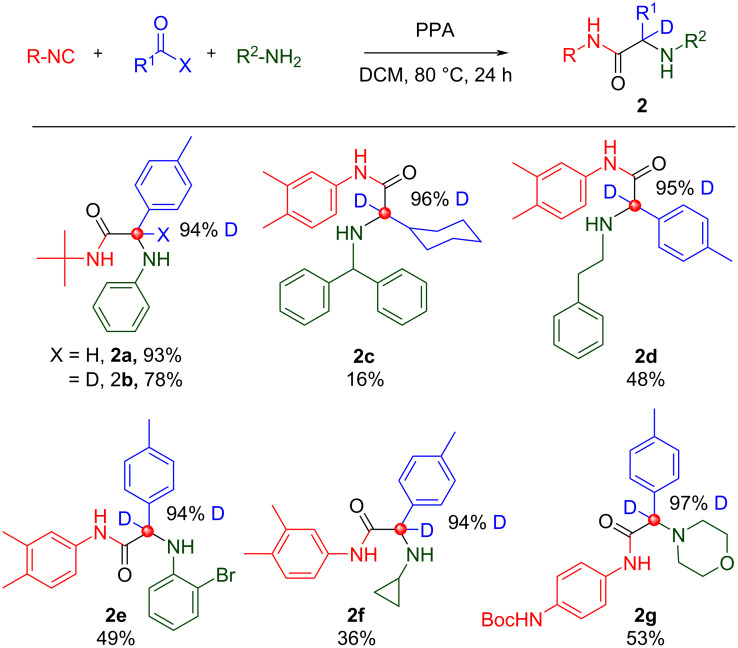
Ugi-3CR products. No deuterium scrambling observed.

The catalytic three-component Ugi reaction was first reported by List in 2008 [33–34] and is comprised of reaction of an isonitrile, amine, and aldehyde/ketone, in the presence of phenylphosphinic acid (PPA), to give α-amino amides [35]. Examples of deuterated Ugi-3CR products are represented in Scheme 4. Like the Ugi 4-CR reaction, there was no deuterium scrambling in the Ugi 3-CR. Using a >95% deuterated aldehyde gave a >95% deuterated product. The low yield in **2c** is likely due to the difficult preparation of aliphatic deuterated aldehydes which led us to believe that the starting material was partially decomposed. Nonetheless, both aliphatic and aromatic deuterated aldehydes have been demonstrated to work without loss of deuterium in the Ugi-3CR.

**Scheme 5:**
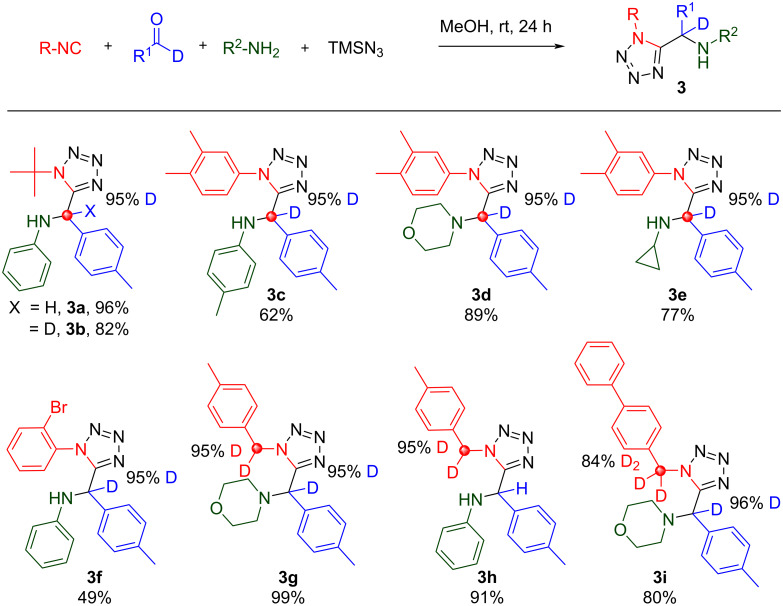
Ugi-azide reaction products, no deuterium scrambling observed.

First reported in 1961, the Ugi-azide reaction differs from the classical Ugi 4-CR in that an azide anion traps out the intermediate nitrilium ion, leading to formation of α-aminotetrazoles [36–39]. Thus, it comprises reaction of an isocyanide, carbonyl, amine and TMSN_3_ to give tetrazole containing products. Isolated yields for eight analogs are reported with excellent retention of the deuterium label (Scheme 5). Of note, **3i** was prepared from combination of a [D_2_]-isocyanide and a [D_1_]-aldehyde.

**Scheme 6:**
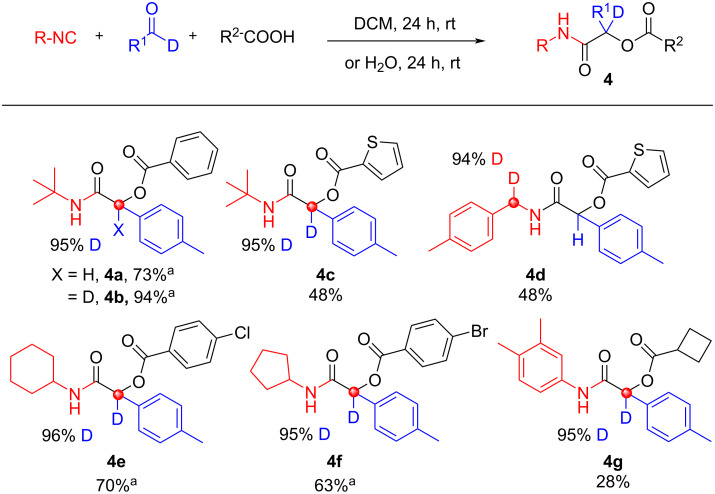
Passerini products, no deuterium scrambling observed. ^a^Water was used as solvent.

Another versatile IMCR is the Passerini reaction [40–43] discovered 60 years prior to the Ugi reaction. It uses the reactivity of isocyanides, aldehydes, and carboxylic acids to yield α-acyloxy amides (Scheme 6). Six deuterated analogs are reported in good yield with no observation of deuterium scrambling. For the preparation of **4d**, a deuterated isocyanide was solely employed and for **4a**,**b** and **4e**,**f**, water was used as solvent. For the latter, product precipitates from the aqueous solution which deters undesirable side-reactions whilst also aiding rate of the reaction.

**Scheme 7:**
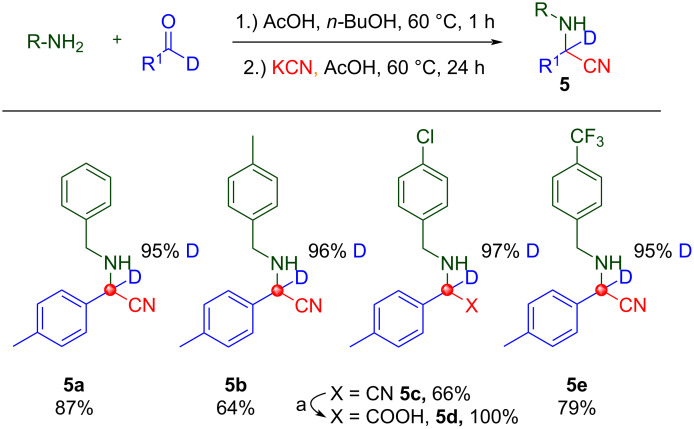
Strecker reaction products (precursors to [D_1_]-α-amino acids), no deuterium scrambling was observed. ^a^The cyano-group was converted to a carboxylic acid via typical saponification conditions without loss of deuterium (See Supporting Information File 1 for conditions).

The fifth MCR employed herein is the ubiquitous Strecker reaction [44–45] where a cyanide, aldehyde, and amine react to afford α-aminonitriles. Compound **5c** was converted to the deuterated amino acid **5d** under acidic conditions. This finding opens up the possibility of scale-production of deuterium-labeled α-amino acids. Deuterated Strecker products are presented in Scheme 7 in good yield with no observed deuterium scrambling.

**Scheme 8:**
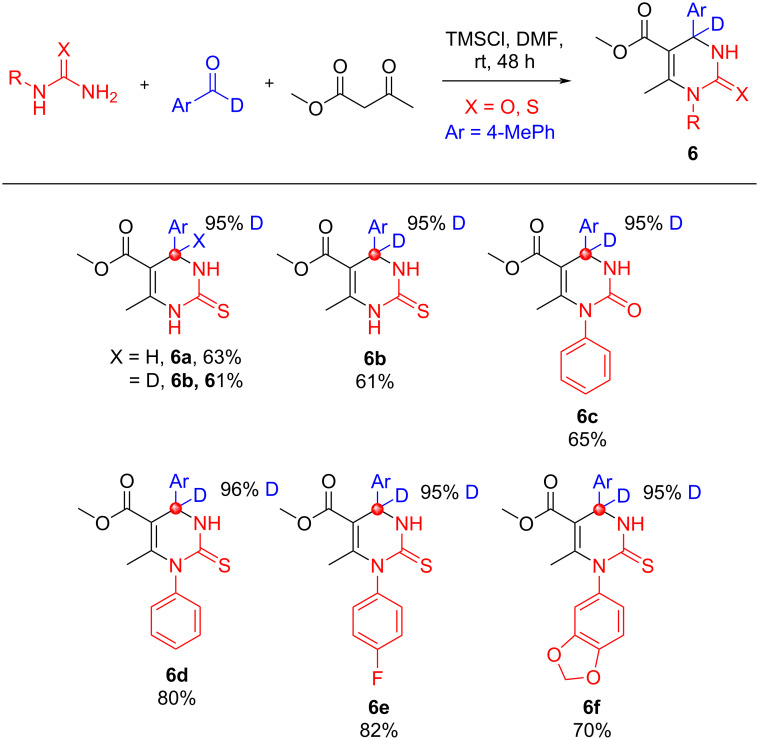
Biginelli reaction products, no deuterium scrambling was observed. Six site-specific deuterated Biginelli products are revealed in good yield with no deuterium scrambling.

The 19th century Biginelli reaction utilizes an arylaldehyde, urea, and acetoacetate component to give 3,4-dihydropyrimidin-2(1*H*)-ones [46]. Such molecules are widely used as calcium channel blockers and antihypertensive agents (Scheme 8) [47].

**Scheme 9:**
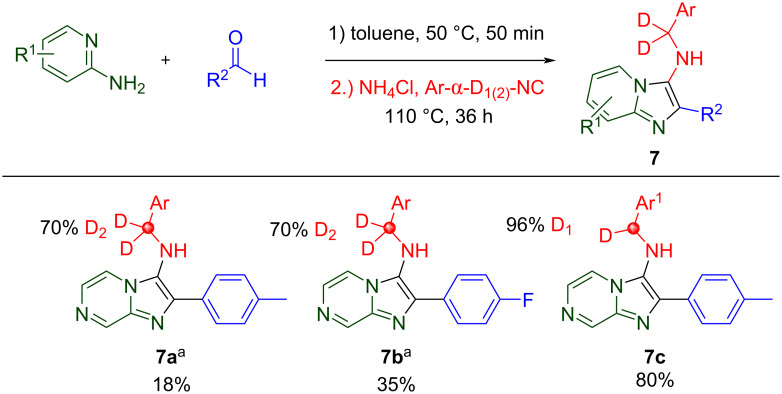
GBB reaction products, no deuterium scrambling was observed. ^a^A 70% [D_2_]-isocyanide was used in **7a** and **7b**. Ar = 4-PhPh, Ar^1^ = 4-MePh.

The Groebke–Blackburn–Bienaymé (GBB) reaction is an intramolecular variant of the Ugi reaction where the intermediate nitrilium ion is intercepted by heteroatoms from the amino-heterocyclic input. Discovered in 1998, and reported independently by three different research groups, it is a three-component reaction of α-amino-heterocycles, aldehydes, and isocyanides which affords various aza-bicyclic molecules [48–52]. The methodology has been widely used for file enhancement purposes. Three deuterated GBB products are presented, Scheme 9. Unlike the previous MCRs, labeling via a deuterated aldehyde is not feasible as deuterium is removed in favor of the aromatic bicyclic product. Both [D_2_]- and [D_1_]-isocyanides are exemplified in **7a**–**c**.

**Scheme 10:**
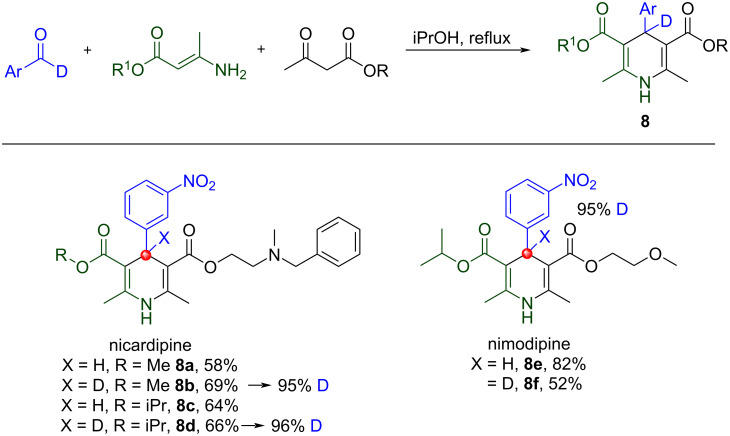
Modified Hantzsch pyridine synthesis to afford 1,4-dihydropyridines. No deuterium scrambling was observed.

Finally, we studied the compatibility of the Hantzsch dihydropyridine synthesis with [D_1_]-aldehydes. The reaction is a condensation of ethyl acetoacetate with aldehyde and ammonia to give 1,4-dihydropyridine [53]. Such scaffolds are seen in several FDA-approved calcium channel blockers including nifedipine, nicardipine and nimodipine and three site-specific deuterated analogs of approved 1,4-dihydropyridines (DHPs) are presented, Scheme 10. In line with all prior MCRs, good yields were observed with no deuterium scrambling.

**Scheme 11:**
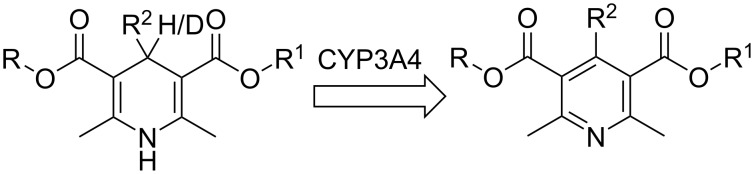
CYP3A4 mediated dehydrogenation of dihydropyridines.

Calcium channel blockers derived from this methodology are heavily metabolized by CYP3A4 via dehydrogenation to afford inactive pyridines, Scheme 11 [54]. As such, bio-isosteric deuterium–hydrogen exchange at this position was thought a reasonable approach to extend drug *t*_1/2_ through exploitation of the kinetic isotope effect underpinned by the C–D bond being slightly shorter and stronger than a C–H bond. Such site-specific labeling was hypothesized to slow CYP3A4 metabolism.

To evaluate the hypothesis, surrogate studies were conducted in mouse liver microsomes to compare deuterated DHPs with their non-deuterated counterparts (Table 2).

**Table 2:** Microsomal stability package of deuterated and non-deuterated dihydropyridines.

Sample ID	MLM *t*_1/2_	MLM Cl^a^

H-nicardipine	2.8 min	1968.2
D-nicardipine	3.0 min	1860.8
H-nimodipine	2.1 min	2572.3
D-nimodipine	2.6 min	2098.5
H-iPr-nicardipine	8.0 min	687.0
D-iPr-nicardipine	14.2 min	386.6

^a^Mouse liver microsomes clearance (MLM Cl), unit: mL/min/kg.

D-Nicardipine saw a marginal increase in stability when compared to its non-deuterated counterpart H-nicardipine (3.0 vs 2.8 min) with both molecules being rapidly metabolized. D-Nimodipine witnessed a 23% improvement in *t*_1/2_ over its non-deuterated counterpart although both molecules were quickly metabolized. The most significant improvement was seen with D-iPr-nicardipine – a 77% increase in MLM stability compared to H-iPr-nicardipine. Note that baseline stability of H-iPr-nicardipine was higher (8 min) related to enhanced ester stability with an isopropyl group versus a methyl ester. Collectively these results point to high potential for translation in vivo where novel deuterated analogs exhibit longer *t*_1/2_ and by extension oral bioavailability.

## Conclusion

In summary we have presented the first method to prepare highly deuterated [D_3_]-formamides and [D_2_]-isocyanides from deuterated aldehydes. Furthermore, we have demonstrated that large libraries of deuterated drug-like molecules can be produced rapidly with MCR technology, with particular value for site selective deuteration of often metabolically soft benzylic C–H sites. Lastly and most importantly, preliminary surrogate metabolic stability studies on site selective [D_1_]-DHPs suggest these novel deuterated analogs may afford increased exposure in an in vivo setting. The methodology is likely to have wide utility for the drug-hunting community at large.

## Supporting Information

File 1 Experimental and analytical data and copies of NMR spectra.

## Data Availability

All data that supports the findings of this study is available in the published article and/or the supporting information to this article.
