# Supplementary material for: Deuterated reagents in multicomponent reactions to afford deuterium-labeled products
Source: Beilstein J Org Chem. 2024 Sep 6;20:2270–9. doi: 10.3762/bjoc.20.195 (PMC11403814; doi:10.3762/bjoc.20.195)
Supplement: File 1 — Experimental and analytical data and copies of NMR spectra. [file Beilstein_J_Org_Chem-20-2270-s001.pdf]

BEILSTEIN JOURNAL OF ORGANIC CHEMISTRY

Supporting Information

for

Deuterated reagents in multicomponent reactions to afford deuterium-labeled products

Kevin Schofield, Shayna Maddern, Yueteng Zhang, Grace E. Mastin, Rachel Knight, Wei Wang, James Galligan and Christopher Hulme

Beilstein J. Org. Chem. **2024,** *20,* 2270–2279. doi:10.3762/bjoc.20.195

Experimental and analytical data and copies of NMR spectra

License and Terms: This is a supporting information file under the terms of the Creative Commons Attribution License (https://creativecommons.org/licenses/by/4.0). Please note that the reuse, redistribution and reproduction in particular requires that the author(s) and source are credited and that individual graphics may be subject to special legal provisions.
The license is subject to the *Beilstein Journal of Organic Chemistry* terms and conditions: (https://www.beilstein-journals.org/bjoc/terms)

Table of Contents

1.) General information S3

2.) Ugi-4 component reaction S3 – S6

3.) Ugi-3 component reaction S6 – S10

4.) Ugi-azide reaction S10 – S15

5.) Passerini reaction S15 – S18

6.) Strecker reaction S18 – S22

7.) Biginelli reaction S22 – S25

8.) Groebke–Blackburn–Bienaymé reaction S25 – S27

9.) Asymmetric Hantzsch dihydropyridine synthesis S27 – S31

10.) Deuterated isocyanide synthesis S31 – S35

11.) Mouse liver microsome procedure S35 – S36

12.) References S36

13.) NMR Spectra S37 – S93

1. General Information

All reagents and solvents were acquired from commercially available suppliers and used without further purification. Deuterated aldehydes were prepared from using previous methodology described by Wang.[1] The products were purified using a Teledyne CombiFlash Rf automated flash chromatography apparatus with a cartridge utilizing the compounds dry loaded using a Teledyne Isco silica column (12–40g). High resolution mass spectra were obtained using an OrbitrapTM for all the compounds, obtained in an Ion Cyclotron Resonance (ICR) spectrometer. ^{1}H and ^{13}C NMR spectra were obtained on a Bruker NMR spectrometer at 400 and 100 MHz respectively. The data is reported as follows: chemical shift in ppm (δ), multiplicity (s = singlet, bs = broad singlet, d = doublet, dd = doublet of doublets, t = triplet, q = quartet, p = pentet, m = multiplet). Coupling constants are reported in Hertz (Hz) and were automatically generated using known NMR analyzer software (MestReNova), these were then subsequently curated. The reactions were always carried out in vacuum-oven dried 5,8, and 20 mL Biotage microwave vials (MWV) and sealed with Blue-Biotage-Teflon septum.

General Ugi-4 component reaction procedure 1

Aldehyde (1.0 equiv, 0.15 mmol), isocyanide (1.0 equiv, 0.15 mmol), amine (1.0 equiv, 0.15 mmol), carboxylic acid (1.0 equiv, 0.15 mmol) and DCM (0.15 mL, 1.0 M) were added simultaneously to a sealed and dry 5 mL microwave vial equipped with a magnetic stir bar. The reaction was stirred for 24 h at room temperature and was then diluted with DCM and concentrated under reduced pressure. The crude residue was purified by automated flash column chromatography using a Teledyne ISCOTM (gradient 0 – 30% EtOAc,/hexanes typically) to give the Ugi 4-component reaction product. For a representative example, see **1a.**

N-(2-(*tert*-Butylamino)-2-oxo-1-(*p*-tolyl)ethyl)-*N*-phenylbenzamide (1a)

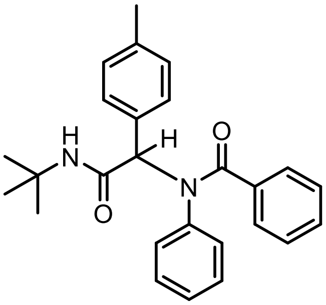

Preparation according to general procedure 1 where 4-methylbenzaldehyde (1.0 equiv, 0.06 mL, 0.50 mmol), *tert*-butyl isocyanide (1.0 equiv, 0.06 mL, 0.50 mmol), aniline (1.0 equiv, 0.05 mL, 0.50 mmol), benzoic acid (1.0 equiv, 61 mg, 0.50 mmol) and MeOH (0.34 mL) were added to a sealed MWV equipped with a magnetic stir bar. The resulting crude residue was purified by flash column chromatography to give title compound **1a** (200 mg, 0.50 mmol, 99% yield, eluting at 20% ethyl acetate/hexanes), a glassy solid. **^{1}H NMR** (400 MHz, $CDCl_3$) δ 7.34 – 7.28 (m, 2H), 7.21 – 7.06 (m, 5H), 7.03 (d, *J* = 8.0 Hz, 2H), 7.00 (s, 5H), 6.05 (s, 1H), 5.79 (s, 1H), 2.28 (s, 3H), 1.37 (s, 9H). **^{13}C NMR** (101 MHz, $CDCl_3$) δ 171.24, 168.97, 141.65, 138.24, 136.35, 132.10, 130.32,

130.09, 129.48, 129.27, 128.68, 128.45, 127.69, 127.11, 67.20, 51.73, 28.82, 21.26. **HRMS** (ESI) m/z: [M + H]+ calculated for $C_{26}H_{28}N_2O_2$ requires 401.2224; found 401.2226.

***N*-(2-(*tert*-Butylamino)-2-oxo-1-(*p*-tolyl)ethyl-1-*d*)-*N*-phenylbenzamide (1b)**

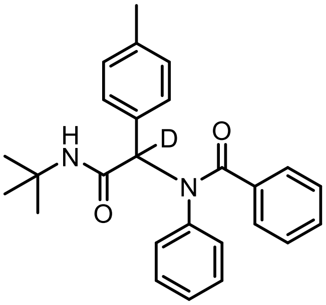

Preparation according to general procedure 1 where [α-D_1]-4-methylbenzaldehyde (1.0 equiv, 0.06 mL, 0.50 mmol), *tert*-butyl isocyanide (1.0 equiv, 0.06 mL, 0.50 mmol), aniline (1.0 equiv, 0.05 mL, 0.50 mmol), benzoic acid (1.0 equiv, 61 mg, 0.50 mmol) and MeOH (0.34 mL) were added to a sealed 5 mL MWV equipped with a magnetic stir bar. The resulting crude residue was purified by flash column chromatography to give title compound **1b** (172 mg, 0.43 mmol, 86% yield, eluting at 20% ethyl acetate/hexanes, 94% D), a glassy solid. **^{1}H NMR** (400 MHz, $CDCl_3$) δ 7.33 – 7.29 (m, 2H), 7.19 – 7.08 (m, 5H), 7.04 (d, *J* = 7.9 Hz, 2H), 7.00 (s, 5H), 6.05 (s, 94% D), 5.80 (s, 1H), 2.29 (s, 3H), 1.38 (s, 9H). **^{13}C NMR** (101 MHz, $CDCl_3$) δ 171.25, 168.98, 141.63, 138.25, 136.35, 132.06, 130.32, 130.07, 129.49, 129.28, 128.69, 128.46, 127.70, 127.12, 51.74, 28.83, 21.27. **HRMS** (ESI) m/z: [M + H]+ calculated for $C_{26}H_{27}DN_2O_2$ requires 402.2286; found 402.2283.

***N*-(2-(*tert*-Butylamino)-2-oxo-1-(*p*-tolyl)ethyl-1-*d*)-*N*-(2-iodophenyl)benzamide (1c)**

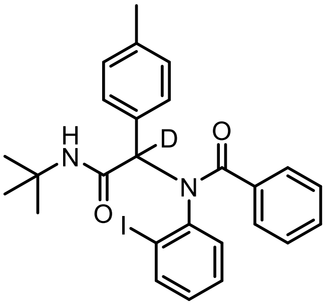

Preparation according to general procedure 1 where [α-D_1]-4-methylbenzaldehyde (1.0 equiv, 0.10 mL, 0.74 mmol), *tert*-butyl isocyanide (1.0 equiv, 0.09 mL, 0.74 mmol), 2-iodoaniline (1.0 equiv, 163 mg, 0.74 mmol), benzoic acid (1.0 equiv, 90 mg, 0.74 mmol) and MeOH (0.53 mL) were added to a sealed MWV equipped with a magnetic stir bar. The resulting crude residue was purified by flash column chromatography to give title compound **1c** (292 mg, 0.55 mmol, 74% yield, eluting at 18% ethyl acetate/hexanes, 96% D), a clear oil. **^{1}H NMR** (400 MHz, DMSO) δ 7.97 (dd, *J* = 8.0, 1.6 Hz, 1H), 7.80 (s, 1H), 7.34 (dd, *J* = 7.8, 1.4 Hz, 1H), 7.34 – 7.27 (m, 2H), 7.26 – 7.12 (m, 6H), 6.90 (d, *J* = 7.9 Hz, 2H), 6.70 (td, *J* = 7.6, 1.6 Hz, 1H), 6.13 (s, 96% D), 2.13 (s, 3H), 1.28 (s, 9H). **^{13}C NMR** (101 MHz, DMSO) δ 169.35, 168.94, 142.02, 138.50, 136.95, 136.83,

133.69, 130.77, 130.67, 129.56, 129.25, 128.26, 127.97, 127.77, 127.07, 50.29, 40.14, 39.94, 39.73, 39.52, 39.31, 39.10, 38.89, 28.42, 20.58. **HRMS** (ESI) m/z: [M + H]+ calculated for $C_{26}H_{26}DIN_2O_2$ requires 528.12527; found 528.12488.

4-(*N*-(Thiophen-2-ylmethyl)benzamido)-*N*-(*p*-tolylmethyl-*d*)tetrahydro-*2H*-pyran-4-carboxamide (1e)

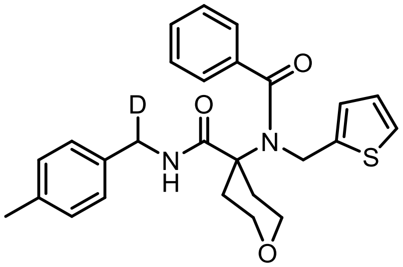

Preparation according to general procedure 1 where 4-oxotetrahydropyran (1.0 equiv, 20 mg, 0.20 mmol), α-deutero-4-methylbenzyl isocyanide (1.0 equiv, 30 mg, 0.20 mmol), 2-thiophenemethylamine (1.0 equiv, 24 mg, 0.20 mmol), benzoic acid (1.0 equiv, 25 mg, 0.20 mmol) and MeOH (0.16 mL) were added to a sealed 5 mL MWV equipped with a magnetic stir bar. The resulting crude residue was purified by flash column chromatography to give title compound **1e** (51 mg, 0.11 mmol, 56% yield, eluting at 50% ethyl acetate/hexanes), a yellow solid. **^{1}H NMR** (400 MHz, $CDCl_3$) δ 7.74 (d, *J* = 5.6 Hz, 1H), 7.51 – 7.36 (m, 5H), 7.18 – 7.09 (m, 5H), 6.83 (dd, *J* = 5.1, 3.5 Hz, 1H), 6.75 – 6.69 (m, 1H), 4.79 (d, *J* = 0.9 Hz, 2H), 4.37 (d, *J* = 5.8 Hz, 95% D), 4.25 (d, *J* = 5.5 Hz, 1H), 3.94 – 3.72 (m, 4H), 2.70 – 2.58 (m, 2H), 2.35 (s, 3H), 2.26 (ddd, *J* = 13.5, 8.6, 4.6 Hz, 2H). **^{13}C NMR** (101 MHz, $CDCl_3$) δ 175.39, 172.01, 140.51, 137.00, 136.97, 135.45, 130.71, 129.40, 128.83, 127.92, 127.56, 127.04, 126.62, 125.39, 64.61, 63.85, 46.38, 43.30 (t, *J* = 21.4 Hz), 33.82, 21.25. **HRMS** (ESI) m/z: [M + H]+ calculated for $C_{26}H_{27}DN_2O_3S$ requires 472.17756; found 472.17743.

***N*-Cyclopropyl-*N*-(2-oxo-1-(*p*-tolyl)-2-((*p*-tolylmethyl-*d*)amino)ethyl)benzamide (1f)**

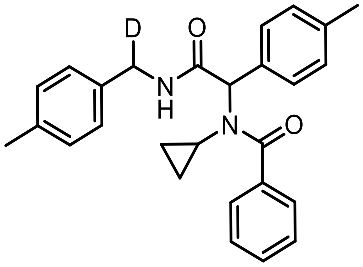

Preparation according to general procedure 1 where 4-methylbenzaldehyde (1.0 equiv, 22 mg, 0.18 mmol), α-deutero-4-methylbenzyl isocyanide (1.0 equiv, 25 mg, 0.18 mmol), cyclopropylamine (1.0 equiv, 10 mg, 0.18 mmol), benzoic acid (1.0 equiv, 22 mg, 0.18 mmol) and MeOH (0.11 mL) were added to a sealed 5 mL MWV equipped with a magnetic stir bar. The resulting crude residue was purified by flash column chromatography to give title compound **1f** (44 mg, 0.11 mmol, 59% yield), a white solid. **^{1}H NMR** (400 MHz, $CDCl_3$) δ 7.61 – 7.54 (m, 2H), 7.44 – 7.36 (m, 5H), 7.21 – 7.08 (m, 6H), 6.40 (s, 1H), 5.71 (s, 1H), 4.45 (d, *J* = 5.7 Hz, 1H), 4.27 (d, *J* = 5.1 Hz, 95% D),

2.62 (tt, J = 6.7, 4.0 Hz, 1H), 2.36 (s, 3H), 2.32 (s, 3H), 0.78 – 0.69 (m, 1H), 0.43 – 0.30 (m, 3H). **^{13}C NMR** (101 MHz, $CDCl_3$) δ 173.60, 170.30, 138.35, 137.19, 137.08, 135.23, 132.45, 129.95, 129.80, 129.51, 129.40, 128.09, 127.72, 127.70, 66.94, 43.16, 32.82, 21.28, 21.21, 11.18, 10.15. **HRMS** (ESI) m/z: [M + H]+ calculated for $C_{27}H_{27}DN_2O_2$ requires 414.2286; found 414.2276.

2-(3-(*1H*-Indol-3-yl)-2-oxoquinoxalin-1(*2H*)-yl)-*N*-cyclopentyl-2-(*p*-tolyl)acetamide-2-*d* (1g)

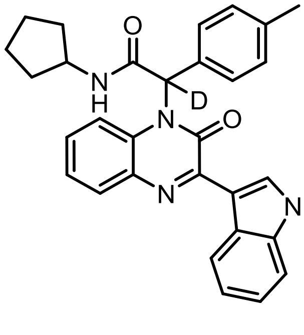

[α-D_1]-4-methylbenzaldehyde (1.3 equiv, 34 mg, 0.28 mmol), cyclopentyl isocyanide (1.3 equiv, 26 mg, 0.27mmol), 2-(Boc-amino)aniline (1.0 equiv, 44 mg, 0.21 mmol), 3-indoleglyoxylic acid (1.0 equiv, 40 mg, 0.21 mmol) and MeOH (0.21 mL) were added to a sealed 5 mL MWV equipped with a magnetic stir bar. The reaction mixture was stirred for 48 hours to form **1d** and 10% TFA/DCM was added to the sealed MWV and was allowed to stir for an additional 24 hours. The reaction vessel was then diluted with DCM and concentrated under reduced pressure. The resulting crude residue was purified by flash column chromatography to give title compound **1g** (75 mg, 0.16 mmol, 74% yield, eluting at 35% ethyl acetate/hexanes, 96% D), a yellow solid. **^{1}H NMR** (400 MHz, $CDCl_3$) δ 9.04 (s, 1H), 8.93 (d, J = 8.0 Hz, 1H), 8.88 (d, J = 2.9 Hz, 1H), 8.01 – 7.96 (m, 1H), 7.40 – 7.27 (m, 7H), 7.25 – 7.13 (m, 3H), 6.57 (s, 96% D), 5.95 (d, J = 7.5 Hz, 1H), 4.29 (h, J = 6.8 Hz, 1H), 3.93 (s, 1H), 2.34 (s, 3H), 1.96 (ddt, J = 15.3, 13.1, 6.4 Hz, 1H), 1.66 – 1.29 (m, 6H). **^{13}C NMR** (101 MHz, $CDCl_3$) δ 167.65, 154.97, 151.23, 138.64, 136.57, 136.31, 134.63, 132.86, 130.98, 130.79, 129.98, 129.75, 128.16, 128.08, 126.69, 124.06, 123.50, 123.21, 122.66, 121.78, 114.96, 112.58, 111.64, 111.36, 52.83, 52.04, 32.92, 32.83, 23.82, 23.78, 21.27. There appears to be a minor rotamer present in ^{1}H and ^{13}C NMRs. **HRMS** (ESI) m/z: [M + H]+ calculated for $C_{30}H_{27}DN_4O_2$ requires 478.2348; found 478.2348.

General Ugi-3 component reaction procedure 2

Aldehyde (1.0 equiv, 0.15 mmol), isocyanide (1.0 equiv, 0.15 mmol), amine (1.0 equiv, 0.15 mmol), and DCM (0.15 mL, 1.0 M) were added simultaneously to a sealed and dry 5 mL microwave vial equipped with a magnetic stir bar containing phenylphosphinic acid (1.0 equiv, 0.15 mmol). The reaction was stirred for 16 h at 80 °C and was then diluted with DCM and concentrated under reduced pressure. The crude residue was extracted with DCM (3 x 10 mL) and washed with saturated sodium bicarbonate. The organic layers were recombined, dried over sodium sulfate, and concentrated under reduced pressure. The crude residue was purified by automated flash column chromatography using a Teledyne ISCOTM (gradient 0–30% EtOAc,/hexanes typically) to give the Ugi 3-component reaction product. For a representative example, see **2a.**

***N*-(*tert*-Butyl)-2-(phenylamino)-2-(*p*-tolyl)acetamide (2a)**

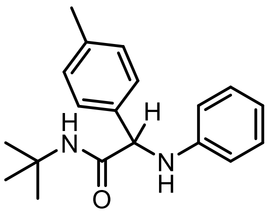

Preparation according to general procedure 2 where 4-methylbenzaldehyde (1.0 equiv, 0.10 mL, 0.84 mmol), aniline (1.0 equiv, 0.08 mL, 0.84 mmol), *tert*-butyl isocyanide (1.0 equiv, 0.10 mL, 0.84 mmol), and DCM (0.53 mL) were added to a sealed 5 mL MWV equipped with a magnetic stir bar containing phenylphosphinic acid (1.0 equiv, 120 mg, 0.84 mmol). The resulting crude residue was purified by flash column chromatography to give title compound **2a** (233 mg, 0.78 mmol, 93% yield, eluting at 10% ethyl acetate/hexanes), a white solid. **^{1}H NMR** (400 MHz, $CDCl_3$) δ 7.30 (d, *J* = 8.0 Hz, 2H), 7.21 – 7.15 (m, 4H), 6.79 (tt, *J* = 7.3, 1.2 Hz, 1H), 6.62 (d, *J* = 7.9 Hz, 2H), 6.51 (s, 1H), 4.56 (d, *J* = 2.2 Hz, 1H), 4.46 (s, 1H), 2.35 (s, 3H), 1.32 (s, 9H). **^{13}C NMR** (101 MHz, $CDCl_3$) δ 170.56, 147.03, 138.37, 136.48, 130.00, 129.39, 127.33, 119.12, 114.03, 64.76, 51.27, 28.70, 21.29. **HRMS** (ESI) m/z: [M + H]+ calculated for $C_{19}H_{24}N_2O$ requires 297.1961; found 297.1964.

***N*-(*tert*-Butyl)-2-(phenylamino)-2-(*p*-tolyl)acetamide-2-*d* (2b)**

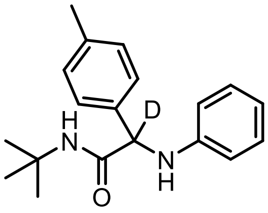

Preparation according to general procedure 2 where [α-D_1]-4-methylbenzaldehyde (1.0 equiv, 0.11 mL, 0.84 mmol), aniline (1.0 equiv, 0.08 mL, 0.84 mmol), *tert*-butyl isocyanide (1.0 equiv, 0.10 mL, 0.84 mmol), and DCM (0.52 mL) were added to a sealed MWV equipped with a magnetic stir bar containing phenylphosphinic acid (1.0 equiv, 120 mg, 0.84 mmol). The resulting crude residue was purified by flash column chromatography to give title compound **2b** (196 mg, 0.66 mmol, 78% yield, eluting at 10% ethyl acetate/hexanes, 94% D), a white solid. **^{1}H NMR** (400 MHz, $CDCl_3$) δ 7.31 (d, *J* = 8.1 Hz, 2H), 7.22 – 7.15 (m, 4H), 6.80 (tt, *J* = 7.4, 1.1 Hz, 1H), 6.63 (dd, *J* = 8.7, 1.1 Hz, 2H), 6.54 – 6.49 (m, 1H), 4.57 (d, *J* = 2.1 Hz, 94% D), 4.45 (s, 1H), 2.35 (s, 3H), 1.33 (s, 9H). **^{13}C NMR** (101 MHz, $CDCl_3$) δ 170.55, 147.03, 138.35, 136.42, 129.99, 129.39, 127.30, 119.11, 114.01, 51.25, 28.70, 21.29. **HRMS** (ESI) m/z: [M + H]+ calculated for $C_{19}H_{23}DN_2O$ requires 298.2024; found 298.2023.

2-(Benzhydrylamino)-2-cyclohexyl-*N*-(3,4-dimethylphenyl)acetamide-2-*d* (2c)

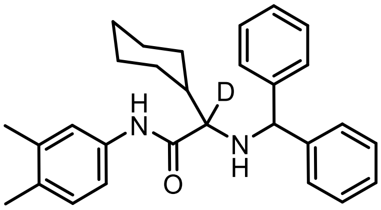

Preparation according to general procedure 2 where cyclohexanecarboxaldehyde-*d* (1.0 equiv, 0.05 mL, 0.38 mmol), diphenylmethanamine (1.0 equiv, 0.07 mL, 0.38 mmol), and DCM (0.25 mL) were added to a sealed 5 mL MWV equipped with a magnetic stir bar containing 3,4-dimethylphenylisocyanide (1.0 equiv, 50 mg, 0.38 mmol) and phenylphosphinic acid (1.0 equiv, 54 mg, 0.38 mmol). The resulting crude residue was purified by flash column chromatography to give title compound **2c** (26 mg, 0.06 mmol, 16% yield, 96% D), a yellow semisolid. ^{1}**H NMR** (400 MHz, $CDCl_3$) δ 8.79 (s, 1H), 7.40 – 7.27 (m, 10H), 7.25 – 7.19 (m, 2H), 7.07 (d, *J* = 8.1 Hz, 1H), 4.78 (s, 1H), 4.13 (q, *J* = 6.9 Hz, 96% D), 3.05 (d, *J* = 4.7 Hz, 1H), 2.26 (s, 3H), 2.23 (s, 3H), 1.92 – 1.55 (m, 7H), 1.33 – 1.03 (m, 4H). ^{13}**C NMR** (101 MHz, $CDCl_3$) δ 171.95, 143.50, 142.85, 137.37, 135.48, 132.53, 130.07, 128.87, 128.69, 127.73, 127.61, 127.41, 127.36, 120.91, 117.04, 66.57, 65.86, 41.87, 30.38, 28.77, 26.45, 26.37, 26.33, 20.01, 19.31.

***N*-(3,4-Dimethylphenyl)-2-(phenethylamino)-2-(*p*-tolyl)acetamide-2-*d* (2d)**

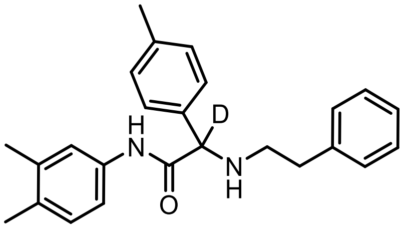

Preparation according to general procedure 2 where [α-D_1]-4-methylbenzaldehyde (1.0 equiv, 24 mg, 0.20 mmol), phenethylamine (1.0 equiv, 24 mg, 0.20 mmol), and DCM (0.14 mL) were added to a sealed 5 mL MWV equipped with a magnetic stir bar containing 3,4-dimethylphenylisocyanide (1.0 equiv, 26 mg, 0.20 mmol) and phenylphosphinic acid (1.0 equiv, 28 mg, 0.20 mmol). The resulting crude residue was purified by flash column chromatography to give title compound **2d** (36 mg, 0.10 mmol, 48% yield, eluting at 26% ethyl acetate/hexanes). ^{1}**H NMR** (400 MHz, $CDCl_3$) δ 9.09 (s, 1H), 7.38 – 7.31 (m, 2H), 7.30 – 7.22 (m, 5H), 7.20 – 7.11 (m, 4H), 7.03 (d, *J* = 8.1 Hz, 1H), 4.23 (s, 94% D), 3.16 – 3.03 (m, 1H), 3.01 – 2.74 (m, 3H), 2.33 (s, 3H), 2.23 (s, 3H), 2.22 (s, 3H), 1.93 (bs, 1H). ^{13}**C NMR** (101 MHz, $CDCl_3$) δ 170.21, 139.75, 138.08, 137.13, 136.31, 135.53, 132.36, 129.92, 129.66, 128.99, 128.77, 127.08, 126.53, 120.69, 116.91, 67.62 (t, *J* = 19.5 Hz), 50.00, 36.57, 21.22, 19.85, 19.25. **HRMS** (ESI) m/z: [M + H]+ calculated for $C_{25}H_{27}DN_2O$ requires 374.2337; found 374.2332.

2-((2-Bromophenyl)amino)-*N*-(3,4-dimethylphenyl)-2-(*p*-tolyl)acetamide-2-*d* (2e)

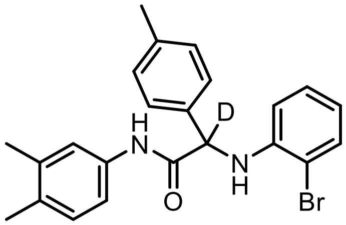

Preparation according to general procedure 2 where [α-D_1]-4-methylbenzaldehyde (1.0 equiv, 46 mg, 0.20 mmol), 2-bromoaniline (1.0 equiv, 66 mg, 0.38 mmol), and DCM (0.25 mL) were added to a sealed 5 mL MWV equipped with a magnetic stir bar containing 3,4-dimethylphenylisocyanide (1.0 equiv, 50 mg, 0.38 mmol) and phenylphosphinic acid (1.0 equiv, 54 mg, 0.38 mmol). The resulting crude residue was purified by flash column chromatography to give title compound **2e** (79 mg, 0.19 mmol, eluting at 12% ethyl acetate in hexanes, 48% yield, 95% D), a yellow semisolid. **^{1}H NMR** (400 MHz, $CDCl_3$) δ 8.34 (s, 1H), 7.49 (dd, *J* = 7.9, 1.5 Hz, 1H), 7.45 – 7.37 (m, 2H), 7.31 (d, *J* = 2.3 Hz, 1H), 7.25 – 7.19 (m, 3H), 7.15 (td, *J* = 7.7, 1.5 Hz, 1H), 7.04 (d, *J* = 8.1 Hz, 1H), 6.70 (td, *J* = 7.6, 1.5 Hz, 1H), 6.63 (dd, *J* = 8.1, 1.4 Hz, 1H), 4.83 (d, *J* = 2.6 Hz, 95% D), 2.37 (s, 3H), 2.22 (s, 3H), 2.21 (s, 3H). **^{13}C NMR** (101 MHz, $CDCl_3$) δ 169.10, 143.76, 138.85, 137.40, 135.09, 135.05, 133.18, 132.69, 130.20, 130.05, 128.89, 127.19, 121.37, 120.10, 117.55, 113.08, 110.92, 64.19 (t, *J* = 20.5 Hz) 21.29, 19.91, 19.29. **HRMS** (ESI) m/z: [M + H]+ calculated for $C_{23}H_{22}DBrN_2O$ requires 424.1129 and 426.1107; found 424.1128 and 426.1107.

2-(Cyclopropylamino)-*N*-(3,4-dimethylphenyl)-2-(*p*-tolyl)acetamide-2-*d* (2f)

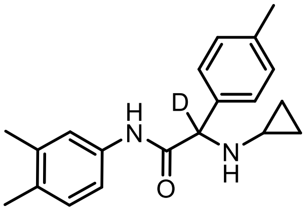

Preparation according to general procedure 2 where [α-D_1]-4-methylbenzaldehyde (1.0 equiv, 0.06 mL, 0.47 mmol), cyclopropylamine (1.0 equiv, 27 mg, 0.47 mmol), and DCM (0.35 mL) were added to a sealed MWV equipped with a magnetic stir bar containing 3,4-dimethylphenylisocyanide (1.0 equiv, 62 mg, 0.47 mmol) and phenylphosphinic acid (1.0 equiv, 67 mg, 0.47 mmol). The resulting crude residue was purified by flash column chromatography to give title compound **2f** (53 mg, 0.06 mmol, 36% yield, eluting at 19% ethyl acetate/hexanes), a brown semisolid. **^{1}H NMR** (400 MHz, $CDCl_3$) δ 9.00 (s, 1H), 7.41 (d, *J* = 2.3 Hz, 1H), 7.35 – 7.27 (m, 3H), 7.17 (d, *J* = 7.8 Hz, 2H), 7.09 (d, *J* = 8.1 Hz, 1H), 4.38 (s, 94% D), 2.36 (s, 3H), 2.34 – 2.28 (m, 1H), 2.27 (s, 3H), 2.25 (s, 3H), 0.60 – 0.46 (m, 4H). **^{13}C NMR** (101 MHz, $CDCl_3$) δ 170.75, 137.94, 137.32, 136.75, 135.55, 132.52, 130.06, 129.67, 127.19, 120.80, 116.95, 68.10 (t, *J* = 20.8 Hz), 30.75, 21.22, 19.96, 19.28, 6.70, 6.42. **HRMS** (ESI) m/z: [M + H]+ calculated for $C_{20}H_{23}DN_2O$ requires 310.2024; found 310.2017.

***tert*-butyl (4-(2-morpholino-2-(*p*-tolyl)acetamido-2-*d*)phenyl)carbamate (2g)**

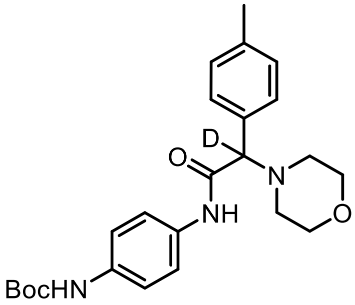

Preparation according to general procedure 2 where [α-D_1]-4-methylbenzaldehyde (36 mg, 0.30 mmol, 1.0 equiv) and morpholine (26 mg, 0.30 mmol, 1.0 equiv) and DCM (0.22 mL) were added to a sealed 5 mL MWV equipped with a magnetic stir bar containing *tert*-butyl (4-isocyanophenyl)carbamate (65 mg, 0.30 mmol, 1.0 equiv) and phenylphosphinic acid (43 mg, 0.30 mmol, 1.0 equiv). The resulting crude residue was purified by flash column chromatography to give title compound **2g** (68 mg, 0.16 mmol, 53% yield, eluting at 75% ethyl acetate in hexanes, 97% D), an orange solid. **^{1}H NMR** (400 MHz, $CDCl_3$) δ 8.98 (s, 1H), 7.50 – 7.45 (m, 2H), 7.31 (d, *J* = 8.6 Hz, 2H), 7.25 – 7.20 (m, 2H), 7.14 (d, *J* = 7.8 Hz, 2H), 6.60 (s, 1H), 4.30 (t, *J* = 4.9 Hz, 97% D), 3.75 (t, *J* = 4.6 Hz, 4H), 2.49 (t, *J* = 4.6 Hz, 4H), 2.32 (s, 3H), 1.51 (s, 9H). **^{13}C NMR** (101 MHz, $CDCl_3$) δ 169.20, 152.93, 138.48, 134.94, 132.97, 131.89, 129.59, 128.85, 120.37, 119.30, 80.58, 67.18, 52.19, 28.46, 21.22. **HRMS** (ESI) m/z: [M + H]+ calculated for $C_{24}H_{30}DN_3O$ requires 427.2450; found 427.2435.

General Ugi-azide reaction procedure 3

Aldehyde (1.0 equiv, 0.15 mmol), isocyanide (1.0 equiv, 0.15 mmol), amine (1.0 equiv, 0.15 mmol), TMS-N3 (1.0 equiv, 0.15 mmol), and methanol (0.15 mL, 1.0M) were added simultaneously to a sealed and dry 5 mL microwave vial equipped with a magnetic stir bar. The reaction was stirred for 24 h at room temperature and was then diluted with DCM and concentrated under reduced pressure. The crude residue was purified by automated flash column chromatography using a Teledyne ISCOTM (gradient 0–30% EtOAc,/hexanes typically) to give the Ugi-azide tetrazole product. For a representative example, see **3a**.

***N*-((1-(*tert*-Butyl)-*1H*-tetrazol-5-yl)(*p*-tolyl)methyl)aniline (3a)**

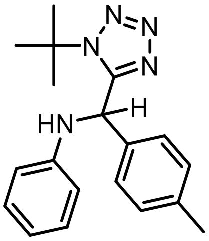

Preparation according to general procedure 3 where MeOH (0.36 mL), 4-methylbenzaldehyde (1.0 equiv, 0.10 mL, 0.75 mmol), *tert*-butyl isocyanide (1.0 equiv, 0.09 mL, 0.75 mmol), aniline (1.0 equiv, 0.07 mL, 0.75 mmol), and TMS-N_3 (1.0 equiv, 0.10 mL, 0.75 mmol) were added

simultaneously to a sealed 5 mL microwave vial equipped with a magnetic stir bar. The crude residue was purified by flash column chromatography to afford title compound **3a** (230 mg, 0.72 mmol, 96% yield), a white solid. **^{1}H NMR** (400 MHz, DMSO) δ 7.36 (d, J = 8.0 Hz, 2H), 7.16 (d, J = 7.9 Hz, 2H), 7.09 – 7.01 (m, 2H), 6.75 – 6.63 (m, 3H), 6.57 (tt, J = 7.3, 1.1 Hz, 1H), 6.18 (d, J = 8.8 Hz, 1H), 2.29 (s, 3H), 1.71 (s, 8H). **^{13}C NMR** (101 MHz, DMSO) δ 155.3, 146.5, 136.9, 135.9, 128.8, 127.9, 117.0, 113.2, 61.8, 51.7, 29.2, 20.6. **HRMS** (ESI) m/z: [M + H]+ calculated for $C_{19}H_{23}N_5$ requires 322.2026; found 323.2023.

***N*-((1-(*tert*-Butyl)-*1H*-tetrazol-5-yl)(*p*-tolyl)methyl-*d*)aniline** (**3b**)

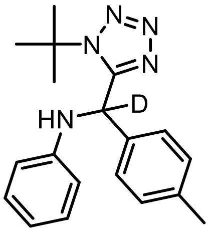

Preparation according to general procedure 3 where MeOH (0.36 mL), [α-D_1]-4-methylbenzaldehyde (1.0 equiv, 0.10 mL, 0.74 mmol, 95% D), *tert*-butyl isocyanide (1.0 equiv, 0.09 mL, 0.74 mmol), aniline (1.0 equiv, 0.07 mL, 0.74 mmol), and TMS-N_3 (1.0 equiv, 0.10 mL, 0.74 mmol) were added simultaneously to a sealed 5 mL microwave vial equipped with a magnetic stir bar. The crude residue was purified by flash column chromatography to afford title compound **3b** (197 mg, 0.61 mmol, 82% yield, 95% D), a white solid. **^{1}H NMR** (400 MHz, DMSO) δ 7.35 (d, J = 8.1 Hz, 2H), 7.14 (d, J = 7.9 Hz, 2H), 7.07 – 6.99 (m, 2H), 6.70 – 6.66 (m, 2H), 6.65 (s, 1H), 6.56 (tt, J = 7.3, 1.1 Hz, 1H), 6.17 (d, J = 9.0 Hz, 95% D), 2.27 (s, 3H), 1.70 (s, 9H). **^{13}C NMR** (101 MHz, DMSO) δ 155.2, 146.5, 136.9, 135.8, 128.8, 127.9, 117.0, 113.2, 61.8, 51.4-51.3 (m, weak signal), 29.2, 20.6. **HRMS** (ESI) m/z: [M + H]+ calculated for $C_{19}H_{22}DN_5$ requires 323.2089; found 323.2092.

***N*-((1-(3,4-dimethylphenyl)-*1H*-tetrazol-5-yl)(*p*-tolyl)methyl-*d*)-4-methylaniline** (**3c**)

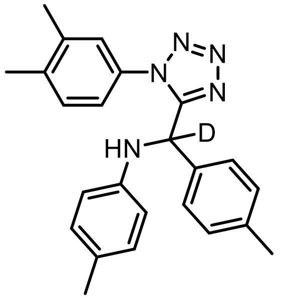

Preparation according to general procedure 3 where MeOH (0.70 mL), [α-D_1]-4-methylbenzaldehyde (1.0 equiv, 0.14 mL, 1.14 mmol, 95% D), 3,4-dimethylphenyl isocyanide (1.0 equiv, 150 mg, 1.14 mmol), 4-methylaniline (1.0 equiv, 123 mg, 1.14 mmol), and TMS-N_3 (1.0 equiv, 0.15 mL, 1.14 mmol) were added simultaneously to a sealed 5 mL microwave vial equipped with a magnetic stir bar. The crude residue was purified by flash column chromatography to afford title compound **3c** (274 mg, 0.71 mmol, 62% yield, 95% D), a yellow solid. **^{1}H NMR**

(400 MHz, CDCl3) δ 7.26 (d, J = 7.9 Hz, 1H), 7.22 – 7.15 (m, 2H), 7.15 – 7.09 (m, 2H), 7.00 – 6.93 (m, 2H), 6.90 (d, J = 8.5 Hz, 2H), 6.42 (d, J = 8.4 Hz, 2H), 5.77 (s, 95% D), 4.63 (bs, 1H), 2.37 (s, 3H), 2.33 (s, 3H), 2.27 (s, 3H), 2.19 (s, 3H). ^{13}**C NMR** (101 MHz, $CDCl_3$) δ 156.21, 143.29, 140.01, 138.74, 138.68, 134.86, 131.17, 130.83, 129.93, 129.84, 128.52, 127.46, 126.63, 122.83, 114.29, 21.26, 20.53, 19.85, 19.83. **HRMS** (ESI) m/z: [M + H]+ calculated for $C_{24}H_{25}DN_{25}$ requires 385.2245; found 385.2243.

4-((1-(3,4-dimethylphenyl)-*1H*-tetrazol-5-yl)(*p*-tolyl)methyl-*d*)morpholine (**3d**)

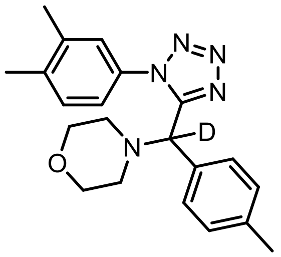

Preparation according to general procedure 3 where MeOH (0.70 mL), [α-D_1]-4-methylbenzaldehyde (1.0 equiv, 0.14 mL, 1.14 mmol, 95% D), 3,4-dimethylphenyl isocyanide (1.0 equiv, 150 mg, 1.14 mmol), morpholine (1.0 equiv, 0.10 mL, 1.14 mmol), and TMS-N_3 (1.0 equiv, 0.15 mL, 1.14 mmol) were added simultaneously to a sealed 5 mL microwave vial equipped with a magnetic stir bar. The crude residue was purified by flash column chromatography to afford title compound **3d** (372 mg, 1.02 mmol, 89% yield, 95% D), a yellow oil. **1H NMR** (400 MHz, CDCl3) δ 7.30 – 7.21 (m, 3H), 7.13 (d, J = 7.9 Hz, 2H), 7.02 – 6.94 (m, 2H), 4.68 (s, 95% D) 3.67 (t, J = 4.7 Hz, 4H), 2.58 – 2.51 (m, 2H), 2.40 – 2.35 (m, 5H), 2.34 (s, 3H), 2.30 (s, 3H). ^{13}**C NMR** (101 MHz, CDCl3) δ 154.64, 139.91, 138.75, 138.65, 131.72, 131.29, 130.76, 129.48, 129.41, 126.83, 123.12, 67.01, 51.12, 21.25, 19.89, 19.80. **HRMS** (ESI) m/z: [M + H]+ calculated for $C_{21}H_{25}DN_5O$ requires 365.2195, found 365.2188.

***N*-((1-(3,4-Dimethylphenyl)-*1H*-tetrazol-5-yl)(*p*-tolyl)methyl-*d*)cyclopropanamine** (**3e**)

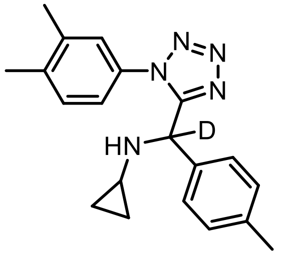

Preparation according to general procedure 3 where MeOH (0.45 mL), [α-D_1]-4-methylbenzaldehyde (1.0 equiv, 0.10 mL, 0.78 mmol, 95% D), cyclopropylamine (1.0 equiv, 0.05 mL, 0.78 mmol), and TMS-N_3 (1.0 equiv, 0.10 mL, 0.78 mmol) were added simultaneously to a sealed 5 mL microwave vial equipped with a magnetic stir bar containing 3,4-dimethylphenyl isocyanide (1.0 equiv, 100 mg, 0.78 mmol). The crude residue was purified by flash column chromatography to afford title compound **3e** (203 mg, 0.78 mmol, 77% yield, 95% D), a yellow oil. ^{1}**H NMR** (400 MHz, CDCl3) δ 7.28 – 7.21 (m, 1H), 7.18 – 7.10 (m, 4H), 7.01 – 6.95 (m, 2H), 4.98 (s, 95% D), 2.85 (s, 1H), 2.35 (s, 3H), 2.33 (s, 3H), 2.28 (s, 3H), 2.02 – 1.95 (m, 1H), 0.43 –

0.31 (m, 4H). **^{13}C NMR** (101 MHz, CDCl3) δ 156.52, 139.69, 138.55, 138.33, 135.65, 131.28, 130.72, 129.70, 127.71, 126.47, 122.72, 28.53, 21.24, 19.83, 19.78, 6.75, 6.69. **HRMS** (ESI) m/z: [M + H]+ calculated for $C_{20}H_{23}DN_5$ requires 335.2089; found 335.2085.

***N*-((1-(2-Bromophenyl)-*1H*-tetrazol-5-yl)(*p*-tolyl)methyl-*d*)aniline** (**3f**)

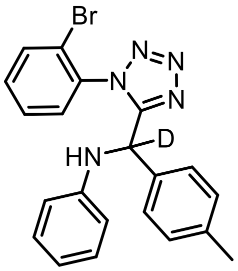

Preparation according to general procedure 3 where MeOH (0.25 mL), [α-D_1]-4-methylbenzaldehyde (1.1 equiv, 50 mg, 0.37 mmol, 95% D), aniline (1.0 equiv, 31 mg, 0.34 mmol), and TMS-N_3 (1.0 equiv, 39 mg, 0.34 mmol) were added simultaneously to a sealed 5 mL microwave vial equipped with a magnetic stir bar containing 3,4-dimethylphenyl isocyanide (1.0 equiv, 61 mg, 0.34 mmol). The crude residue was purified by flash column chromatography to afford title compound **3e** (70 mg, 0.17 mmol, 49% yield, eluting at 15% ethyl acetate,95% D), a white solid**. ^{1}H NMR** (400 MHz, $CDCl_3$) δ 7.77 (d, *J* = 8.0 Hz, 1H), 7.46 (td, *J* = 7.8, 1.6 Hz, 1H), 7.36 (s, 1H), 7.16 – 7.06 (m, 2H), 7.02 (s, 5H), 6.72 (tt, *J* = 7.3, 1.1 Hz, 1H), 6.59 (d, *J* = 7.9 Hz, 2H), 5.64 (d, *J* = 6.8 Hz, 95% D), 5.02 (s, 1H), 2.29 (s, 3H). **^{13}C NMR** (101 MHz, $CDCl_3$) δ 156.87, 145.67, 138.77, 134.03, 132.84, 132.73, 129.75, 129.36, 128.54, 127.36, 119.01, 114.01, 21.21. **HRMS** (ESI) m/z: [M + H]+ calculated for $C_{21}H_{17}DBrN_5$ requires 421.08811 and 423.08629, found 421.08786 and 423.08569.

4-(*p*-Tolyl(1-(*p*-tolylmethyl-*d*)-*1H*-tetrazol-5-yl)methyl-*d*)morpholine (**3g**)

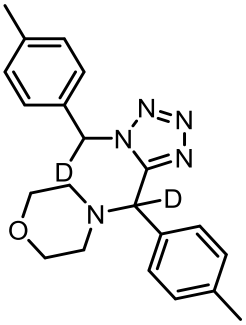

Preparation according to general procedure 1 where MeOH (0.08 mL), 4-methylbenzaldehyde-α-D_1 (1.0 equiv, 21 mg, 0.17 mmol), [α-D_1]-4-methylbenzyl isocyanide (1.0 equiv, 23 mg, 0.17 mmol, 95% D), morpholine (1.0 equiv, 14 mg, 0.17 mmol), and TMS-N_3 (1.0 equiv, 19 mg, 0.17 mmol) were added simultaneously to a sealed 5 mL microwave vial equipped with a magnetic stir bar. The crude residue was purified by flash column chromatography to afford title compound **3g** (60 mg, 0.16 mmol, 99% yield, 95% D), a clear semisolid. **^{1}H NMR** (400 MHz, CDCl3) δ 7.26 – 7.13 (m, 2H), 7.14 – 7.08 (m, 4H), 6.97 (d, J = 8.0 Hz, 2H), 5.63 (s, 95% D), 5.59 (s, 0.5 H), 5.33 (s, 98% D, 95% overall D), 5.30 (s, 0.5 H), 4.68 (s, 95% D), 3.63 (t, J = 4.7 Hz, 4H), 2.50 – 2.39

(m, 2H), 2.34 (s, 3H), 2.33 (s, 3H), 2.31 – 2.24 (m, 2H). **^{13}C NMR** (101 MHz, CDCl3) δ 155.61, 145.65, 139.21, 139.01, 133.98, 130.08, 130.03, 129.37, 127.81, 127.80, 127.46, 119.13, 113.85, 53.22, 50.99, 21.30, 21.26. **HRMS** (ESI) m/z: [M + H]+ calculated for $C_{21}H_{24}D_2N_5O$ requires 366.2257, found 366.2245.

***N*-(*p*-Tolyl(1-(*p*-tolylmethyl-*d*)-1*H*-tetrazol-5-yl)methyl)aniline** (**3h**)

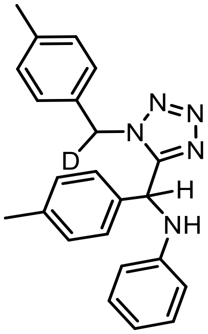

Preparation according to general procedure 1 where MeOH (0.08 mL), 4-methylbenzaldehyde (1.0 equiv, 24 mg, 0.20 mmol), [α-D_1]-4-methylbenzyl isocyanide (1.0 equiv, 28 mg, 0.20 mmol, 95% D), aniline (1.0 equiv, 19 mg, 0.20 mmol), and TMS-N_3 (1.0 equiv, 23 mg, 0.20 mmol) were added simultaneously to a sealed 5 mL microwave vial equipped with a magnetic stir bar. The crude residue was purified by flash column chromatography to afford title compound **3h** (68 mg, 0.18 mmol, 91% yield, 95% D), as an off-white solid. **^{1}H NMR** (400 MHz, CDCl3) δ 7.18 – 7.09 (m, 6H), 7.09 – 7.02 (m, 2H), 7.00 (d, J = 7.9 Hz, 2H), 6.71 (tt, J = 7.3, 1.1 Hz, 1H), 6.36 (d, J = 8.5 Hz, 2H), 5.69 (d, J = 1.4 Hz, 1H), 5.57 (s, 0H), 5.54 (s, 0.5H), 5.57 (s, 95% D), 5.20 (s, 97% D), 5.17 (s, 0.5H), 4.60 (s, 1H), 2.37 (s, 3H), 2.34 (s, 3H), (Overall Deuteration: 95%). **^{13}C NMR** (101 MHz, CDCl3) δ 155.60, 145.64, 139.20, 139.00, 133.97, 130.07, 130.02, 129.36, 127.81, 127.79, 127.45, 119.12, 113.84, 53.21, 50.98 (t, J = 22.6 Hz), 21.29, 21.25. **HRMS** (ESI) m/z: [M + H]+ calculated for $C_{23}H_{23}DN_5$ requires 371.2089, found 371.2081.

4-((1-([1,1'-Biphenyl]-4-ylmethyl-*d*2)-1*H*-tetrazol-5-yl)(*p*-tolyl)methyl-*d*)morpholine (3i)

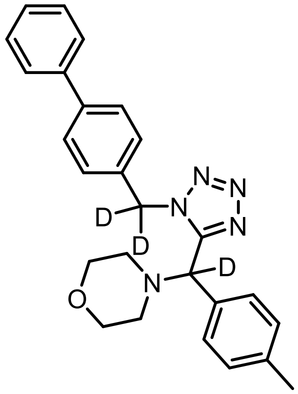

Preparation according to general procedure 1 where MeOH (0.12 mL), morpholine (1.5 equiv, 13 mg, 0.15 mmol), and TMS-N_3 (1.0 equiv, 12 mg, 0.10 mmol) were added simultaneously to a sealed 5 mL microwave vial equipped with a magnetic stir bar containing 4-methylbenzaldehyde-

α-D_1 (1.5 equiv, 20 mg, 0.16 mmol), 4-(isocyanomethyl-d2)-1,1'-biphenyl (1.0 equiv, 20 mg, 0.10 mmol, 84% D_2),. The crude residue was purified by flash column chromatography to afford title compound **3i** (35mg, 0.08 mmol, 80% yield, eluting at 60% ethyl acetate in hexanes, 96% D), a yellow solid. **^{1}H NMR** (400 MHz, $CDCl_3$) δ 7.54 (td, *J* = 7.5, 1.7 Hz, 4H), 7.48 – 7.41 (m, 2H), 7.40 – 7.33 (m, 1H), 7.22 – 7.16 (m, 2H), 7.16 – 7.06 (m, 4H), 5.64 (s, 92% D), 5.45 (s, 92% D, Overall: 84% D_2), 4.75 (s, 96% D), 3.64 (t, *J* = 4.7 Hz, 4H), 2.48 (dq, *J* = 10.3, 5.3 Hz, 2H), 2.37 – 2.25 (m, 5H). **^{13}C NMR** (101 MHz, $CDCl_3$) δ 154.58, 141.93, 140.15, 138.90, 132.22, 130.72, 129.59, 129.15, 129.03, 128.02, 128.00, 127.90, 127.80, 127.17, 66.86, 51.42, 21.21. **HRMS** (ESI) m/z: [M + H]+ calculated for $C_{26}H_{24}D_3N_5O$ requires 429.24767; found 429.24729.

General Passerini reaction procedure 4

Aldehyde (1.0 equiv, 0.15 mmol), isocyanide (1.0 equiv, 0.15 mmol), carboxylic acid (1.0 equiv, 0.15 mmol) and DCM (0.15 mL, 1.0 M) or water (0.75 mL, 0.20 M) were added simultaneously to a sealed and dry 5 mL microwave vial equipped with a magnetic stir bar. The reaction was stirred for 16 h at room temperature and was then diluted with DCM and concentrated under reduced pressure. The crude residue was purified by automated flash column chromatography using a Teledyne ISCOTM (gradient 0–30% EtOAc/hexanes typically) to give the Passerini reaction product. For a representative example, see **4a.**

2-(*tert*-Butylamino)-2-oxo-1-(*p*-tolyl)ethyl benzoate (**4a**)

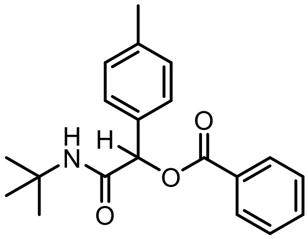

Preparation according to general procedure 4 where 4-methylbenzaldehyde (1.0 equiv, 72 mg, 0.60 mmol), *tert*-butyl isocyanide (1.0 equiv, 50 mg, 0.60 mmol), benzoic acid (1.0 equiv, 73 mg, 0.60 mmol), and H_2O (2.90 mL) were added to a sealed 5 mL MWV equipped with a magnetic stir bar. The resulting crude residue was purified by flash column chromatography to give title compound **4a** (144 mg, 0.44 mmol, 73%, eluting at 100% ethyl acetate/hexanes), a white solid. **^{1}H NMR** (400 MHz, $CDCl_3$) δ 8.12 – 8.05 (m, 2H), 7.64 – 7.55 (m, 1H), 7.46 (t, *J* = 7.9 Hz, 2H), 7.40 (d, *J* = 8.0 Hz, 2H), 7.20 (d, *J* = 8.0 Hz, 2H), 6.19 (s, 1H), 5.96 (s, 1H), 2.36 (s, 3H), 1.37 (s, 9H). **^{13}C NMR** (101 MHz, $CDCl_3$) δ 167.65, 165.08, 138.95, 133.65, 133.17, 129.89, 129.64, 129.61, 128.74, 127.59, 76.12, 51.68, 28.85, 21.37.

2-(*tert*-Butylamino)-2-oxo-1-(*p*-tolyl)ethyl-1-d benzoate (4b)

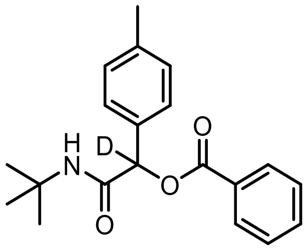

Preparation according to general procedure 4 where [α-D_1]-4-methylbenzaldehyde (1.0 equiv, 36 mg, 0.30 mmol), *tert*-butyl isocyanide (1.0 equiv, 25 mg, 0.30 mmol), benzoic acid (1.0 equiv, 37 mg, 0.30 mmol), and H_2O (1.15 mL) were added to a sealed 5 mL MWV equipped with a magnetic stir bar. The resulting crude residue was purified by flash column chromatography to give title compound **4b** (92 mg, 0.28 mmol, 94% yield, eluting at 42% ethyl acetate in hexanes, 95% D). a white solid. **^{1}H NMR** (400 MHz, $CDCl_3$) δ 8.08 (dd, *J* = 8.3, 1.4 Hz, 2H), 7.60 (tt, *J* = 7.4, 1.2 Hz, 1H), 7.52 – 7.43 (m, 2H), 7.41 (d, *J* = 8.0 Hz, 2H), 7.20 (d, *J* = 7.9 Hz, 2H), 6.19 (s, 95% D), 5.97 (s, 1H), 2.36 (s, 3H), 1.37 (s, 9H). **^{13}C NMR** (101 MHz, $CDCl_3$) δ 167.63, 165.08, 138.94, 133.64, 133.10, 129.88, 129.62, 129.60, 128.72, 127.57, 51.67, 28.83, 21.36.

2-(*tert*-Butylamino)-2-oxo-1-(*p*-tolyl)ethyl-1-*d* thiophene-2-carboxylate (4c)

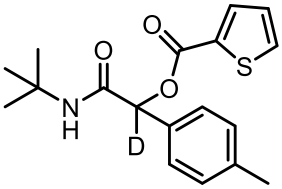

Preparation according to general procedure 4 where [α-D_1]-4-methylbenzaldehyde (1.0 equiv, 0.11 mL, 0.90 mmol), *tert*-butyl isocyanide (1.0 equiv, 0.10 mL, 0.90 mmol), 2-thiophenecarboxylic acid (1.0 equiv, 100 mg, 0.90 mmol), and DCM (0.59 mL) were added to a sealed 5 mL MWV equipped with a magnetic stir bar. The resulting crude residue was purified by flash column chromatography to give title compound **4c** (143 mg, 0.43 mmol, 48%, eluting at 17% ethyl acetate/hexanes, 95% D), a white solid. **^{1}H NMR** (400 MHz, $CDCl_3$) δ 7.87 (dd, *J* = 3.8, 1.3 Hz, 1H), 7.61 (dd, *J* = 5.0, 1.3 Hz, 1H), 7.40 – 7.35 (m, 2H), 7.21 – 7.16 (m, 2H), 7.14 (dd, *J* = 5.0, 3.8 Hz, 1H), 6.14 (s, 95% D), 6.07 (s, 1H), 2.35 (s, 3H), 1.38 (s, 9H). **^{13}C NMR** (101 MHz, $CDCl_3$) δ 167.41, 160.35, 138.97, 134.42, 133.19, 132.98, 132.85, 129.59, 128.27, 127.54, 51.67, 28.84, 21.37. **HRMS** (ESI) m/z: [M + H]+ calculated for $C_{18}H_{20}DNO_3S$ requires 333.1378; found 333.1372. Note: The signal for the deuterated carbon was too weak to be observed in ^{13}C.

2-Oxo-1-(*p*-tolyl)-2-((*p*-tolylmethyl-*d*)amino)ethyl thiophene-2-carboxylate (4d)

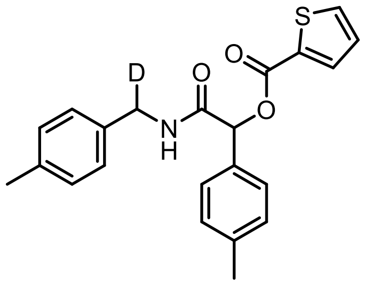

Preparation according to general procedure 4 where 4-methylbenzaldehyde (1.0 equiv, 21 mg, 0.17 mmol), α-deutero-4-methylbenzyl isocyanide (1.0 equiv, 23 mg, 0.17 mmol), 2-thiophenecarboxylic acid (1.0 equiv, 22 mg, 0.17 mmol), and DCM (0.13 mL) were added to a sealed 5 mL MWV equipped with a magnetic stir bar. The resulting crude residue was purified by flash column chromatography to give title compound **4d** (32 mg, 0.08 mmol, 48%, eluting at 16% ethyl acetate/hexanes), a white solid. ^{1}**H NMR** (400 MHz, $CDCl_3$) δ 7.86 (dd, *J* = 3.8, 1.3 Hz, 1H), 7.59 (dd, *J* = 5.0, 1.3 Hz, 1H), 7.44 – 7.36 (m, 2H), 7.20 (d, *J* = 7.7 Hz, 2H), 7.13 (s, 4H), 7.11 (dd, *J* = 5.0, 3.8 Hz, 1H), 6.46 (d, *J* = 5.8 Hz, 1H), 6.30 (s, 1H), 4.56 (d, *J* = 6.0 Hz, 94% D), 4.54 – 4.37 (m, 1H), 2.36 (s, 3H), 2.34 (s, 3H). ^{13}**C NMR** (101 MHz, $CDCl_3$) δ 168.38, 160.53, 139.14, 137.46, 134.78, 134.51, 133.37, 132.66, 132.59, 129.63, 129.56, 128.20, 127.71, 127.50, 76.06, 43.02 (t, *J* = 20.3 Hz), 21.37, 21.22. **HRMS** (ESI) m/z: [M + H]+ calculated for $C_{22}H_{20}DN_3O_3S$ requires 381.1378; found 381.1365.

2-(Cyclohexylamino)-2-oxo-1-(*p*-tolyl)ethyl-1-*d* 4-chlorobenzoate (4e)

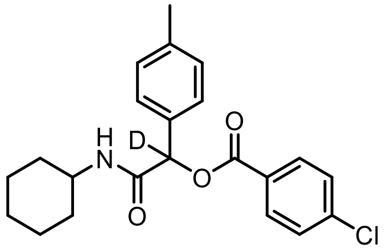

Preparation according to general procedure 4 where [α-D_1]-4-methylbenzaldehyde (1.3 equiv, 36 mg, 0.30 mmol), cyclohexyl isocyanide (1.0 equiv, 25 mg, 0.23 mmol), 4-chlorobenzoic acid (1.3 equiv, 47 mg, 0.30 mmol), and H_2O (1.13 mL) were added to a sealed 5 mL MWV equipped with a magnetic stir bar. The resulting crude residue was purified by flash column chromatography to give title compound **4e** (56 mg, 0. 14 mmol, 63%, eluting at 15% ethyl acetate/hexanes), a white solid. ^{1}**H NMR** (400 MHz, $CDCl_3$) δ 8.01 (d, *J* = 8.6 Hz, 2H), 7.44 (d, *J* = 8.5 Hz, 2H), 7.40 (d, *J* = 8.1 Hz, 2H), 7.20 (d, *J* = 7.9 Hz, 2H), 6.22 (s, 96% D), 5.88 (d, *J* = 8.2 Hz, 1H), 3.82 (dddd, *J* = 14.5, 10.5, 8.0, 3.9 Hz, 1H), 2.36 (s, 3H), 2.00 – 1.83 (m, 2H), 1.75 – 1.55 (m, 3H), 1.45 – 1.29 (m, 2H), 1.24 – 1.04 (m, 3H). ^{13}**C NMR** (101 MHz, $CDCl_3$) δ 167.36, 164.46, 140.19, 139.24, 132.63, 131.32, 129.70, 129.09, 128.06, 127.66, 48.42, 33.10, 33.01, 25.57, 24.86, 24.82, 21.39, **HRMS** (ESI) m/z: [M + H]+ calculated for $C_{22}H_{23}DClNO_3$ requires 387.1575 (major isotope) and 389.1551 (minor isotope); found 387.1575 (major isotope) and 389.1546 (minor isotope).The signal for the deuterated carbon was too weak to be observed in ^{13}C.

2-(Cyclopentylamino)-2-oxo-1-(*p*-tolyl)ethyl-1-*d* 4-bromobenzoate (4f)

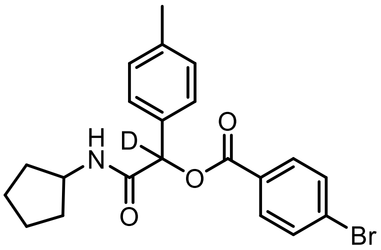

Preparation according to general procedure 4 where [α-D_1]-4-methylbenzaldehyde (1.0 equiv, 36 mg, 0.30 mmol), cyclopentyl isocyanide (1.0 equiv, 28 mg, 0.30 mmol), 4-bromobenzoic acid (1.0 equiv, 60 mg, 0.30 mmol), and H_2O (1.13 mL) were added to a sealed 5 mL MWV equipped with a magnetic stir bar. The resulting crude residue was purified by flash column chromatography to give title compound **4f** (87 mg, 0.21 mmol, 70%, eluting at 14% ethyl acetate/hexanes), a white solid. **^{1}H NMR** (400 MHz, $CDCl_3$) δ 7.93 (d, *J* = 8.6 Hz, 2H), 7.60 (d, *J* = 8.3 Hz, 2H), 7.39 (d, *J* = 8.1 Hz, 2H), 7.20 (d, *J* = 7.8 Hz, 2H), 6.21 (s, 0H), 5.93 (d, *J* = 7.6 Hz, 1H), 4.23 (h, *J* = 7.0 Hz, 1H), 2.36 (s, 3H), 2.07 – 1.91 (m, 2H), 1.72 – 1.53 (m, 4H), 1.47 – 1.26 (m, 2H). **^{13}C NMR** (101 MHz, $CDCl_3$) δ 167.81, 164.62, 139.25, 132.53, 132.08, 131.41, 129.70, 128.87, 128.48, 127.64, 51.43, 33.20, 33.17, 23.84, 23.83, 21.38. **HRMS** (ESI) m/z: [M + H]+ calculated for $C_{21}H_{21}DBrNO_3$ requires 417.0919 and 419.0896; found 417.0923 and 419.0902. Note: the signal for the deuterated carbon was too weak to be observed in ^{13}C.

2-((3,4-Dimethylphenyl)amino)-2-oxo-1-(*p*-tolyl)ethyl-1-*d* cyclobutanecarboxylate (4g)

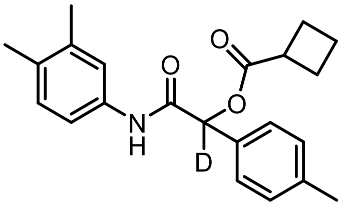

Preparation according to general procedure 4 where to a sealed 5 mL MWV equipped with a magnetic stir bar containing 4-isocyano-1,2-dimethylbenzene (1.00 equiv, 82 mg, 0.63 mmol) was added DCM (0.45 mL), cyclobutanecarboxylic acid (1.00 equiv, 63 mg, 0.63 mmol), and [α-D_1]-4-methylbenzaldehyde (1.00 equiv, 80 mg, 0.63 mmol). The resulting crude residue was purified by flash column chromatography to give the title compound **4g** (61 mg, 0.17 mmol, 28% yield), a light-yellow solid. **^{1}H NMR** (400 MHz, $CDCl_3$) δ 7.68 (s, 1H), 7.41 – 7.34 (m, 2H), 7.32 (d, *J* = 2.3 Hz, 1H), 7.24 – 7.15 (m, 3H), 7.06 (d, *J* = 8.1 Hz, 1H), 6.38 (s, 96% D), 3.39 – 3.26 (m, 1H), 2.50 – 2.26 (m, 7H), 2.23 (s, 3H), 2.22 (s, 3H), 2.12 – 1.90 (m, 2H). **^{13}C NMR** (101 MHz, $CDCl_3$) δ 139.16, 137.50, 134.83, 133.32, 130.13, 129.68, 127.46, 121.36, 117.50, 38.06, 25.38, 25.24, 21.37, 19.98, 19.34, 18.59. **HRMS** (ESI) m/z: [M + H]+ calculated for $C_{22}H_{24}DNO_3$ requires 353.19700; found 353.19678.

General Strecker reaction procedure 5

Preparation according to general procedure 5 where aldehyde (1.2 equiv, 0.18 mmol), amine (1.0 equiv, 0.15 mmol), acetic acid (1.2 equiv, 0.18 mmol) and *n*-butanol (0.60 mL, 0.25 M) were added

simultaneously to a sealed and dry 5 mL microwave vial equipped with a magnetic stir bar and stirred for 1 hour at 60 °C. The reaction mixture was allowed to cool down to room temperature where KCN (2.2 equiv, 0.33 mmol) and acetic acid (1.2 equiv, 0.18 mmol) were added simultaneously. The reaction was stirred for 20 h at 60 °C and was then diluted with DCM and concentrated under reduced pressure. The crude residue was washed with saturated sodium bicarbonate and extracted with DCM (3 x 10 mL). The organic layers were recombined, dried over sodium sulfate, and concentrated under reduced pressure. The crude residue was purified by flash column chromatography (gradient 0–30% EtOAc,/hexanes typically) to give the Strecker reaction product. For a representative example, see **5a.** The residual KCN in the aqueous layer was oxidized to KOCN by addition of hydrogen peroxide for safety purposes.

2-(Benzylamino)-2-(*p*-tolyl)acetonitrile-*d* (5a)

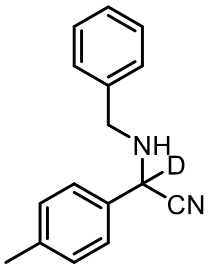

Preparation according to general procedure 5 where [α-D_1]-4-methylbenzaldehyde (1.1 equiv, 45 mg, 0.37 mmol), benzylamine (1.0 equiv, 35 mg, 0.32 mmol), acetic acid (1.1 equiv, 22 mg, 0.37 mmol) and n-butanol (1.30 mL) were added simultaneously to a sealed and dry 5 mL microwave vial equipped with a magnetic stir bar and stirred for 1 hour at 60 °C. KCN (2.1 equiv, 44 mg, 0.68 mmol) and acetic acid (1.1 equiv, 22 mg, 0.37 mmol) were then added to the reaction mixture and was stirred at 60 °C for 20 hours. The resulting crude residue was purified by flash column chromatography to give title compound **5a** (64 mg, 0.27 mmol, 87%, eluting at 10% ethyl acetate/hexanes), a clear oil. **^{1}H NMR** (400 MHz, $CDCl_3$) δ 7.49 – 7.21 (m, 9H), 4.86 (s, 95% D), 4.09 (d, J = 13.0 Hz, 1H), 3.98 (d, J = 13.0 Hz, 1H), 2.41 (s, 3H), 1.87 (s, 1H). **^{13}C NMR** (101 MHz, $CDCl_3$) δ 139.08, 138.33, 131.90, 129.73, 128.74, 128.52, 127.72, 127.32, 119.03, 53.07 (t, J = 22.2 Hz), 51.32, 21.26. **HRMS** (ESI) m/z: [M + H]+ calculated for $C_{16}H_{15}DN_2$ requires 238.1449; found 211.1332.

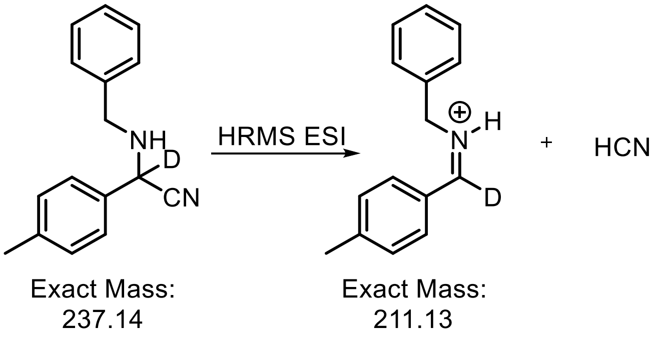

Scheme S1. Explanation for HRMS result with Strecker products.

2-((4-Methylbenzyl)amino)-2-(*p*-tolyl)acetonitrile-*d* (5b)

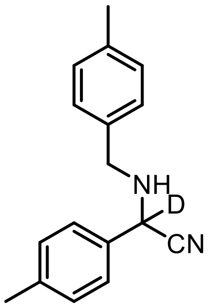

Preparation according to general procedure 5 where [α-D_1]-4-methylbenzaldehyde (1.2 equiv, 36 mg, 0.30 mmol), 4-methylbenzylamine (1.0 equiv, 30 mg, 0.25 mmol), acetic acid (1.2 equiv, 18 mg, 0.30 mmol) and *n*-butanol (1.20 mL) were added simultaneously to a sealed and dry 5 mL microwave vial equipped with a magnetic stir bar and stirred for 1 hour at 60 °C. KCN (2.2 equiv, 36 mg, 0.55 mmol) and acetic acid (1.2 equiv, 18 mg, 0.30 mmol) were then added to the reaction mixture and was stirred at 60 °C for 20 hours. The resulting crude residue was purified by flash column chromatography to give title compound **5b** (40 mg, 0.16 mmol, 64% yield, eluting at 12% ethyl acetate/hexanes), a clear oil ^{1}H NMR (400 MHz, $CDCl_3$) δ 7.42 (d, *J* = 8.2 Hz, 2H), 7.30 (d, *J* = 7.9 Hz, 2H), 7.22 (d, *J* = 7.9 Hz, 2H), 7.18 (d, *J* = 7.7 Hz, 2H), 4.79 (s, 96% D), 4.03 (d, *J* = 12.8 Hz, 1H), 3.92 (d, *J* = 12.9 Hz, 1H), 2.38 (s, 3H), 2.37 (s, 3H), 1.81 (s, 1H).**^{13}C NMR** (101 MHz, $CDCl_3$) δ 139.02, 137.37, 135.25, 132.03, 131.98, 129.70, 129.41, 128.48, 127.31, 119.07, 53.23, 52.96 (t, *J* = 22.0 Hz), 51.08, 51.05, 21.25, 21.23. **HRMS** (ESI) m/z: [M + H]+ calculated for $C_{17}H_{17}DN_2$ requires 252.1606; found 225.1494 (Imine adduct).

2-((4-Chlorobenzyl)amino)-2-(*p*-tolyl)acetonitrile-*d* (5c)

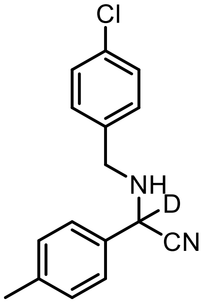

Preparation according to general procedure 5 where [α-D_1]-4-methylbenzaldehyde (1.2 equiv, 36 mg, 0.30 mmol, 96% d_1), (4-chlorophenyl)methanamine (1.0 equiv, 42 mg, 0.30 mmol), acetic acid (1.2 equiv, 22 mg, 0.36 mmol) and n-butanol (1.20 mL) were added simultaneously to a sealed and dry 5 mL microwave vial equipped with a magnetic stir bar and stirred for 1 hour at 60 °C. KCN (2.2 equiv, 43 mg, 0.66 mmol) and acetic acid (1.2 equiv, 22 mg, 0.36 mmol) were then added to the reaction mixture and was stirred at 60 °C for 20 hours. The resulting crude residue was purified by flash column chromatography to give title compound **5c** (54 mg, 0.20 mmol, 66% yield, eluting at 13% ethyl acetate in hexanes, 97% d_1), a clear oil. **^{1}H NMR** (400 MHz, $CDCl_3$) δ 7.41 (d, *J* = 8.2 Hz, 2H), 7.36 – 7.29 (m, 4H), 7.22 (d, *J* = 7.9 Hz, 2H), 4.77 (s, 97% D), 4.01 (d, *J* = 13.2 Hz, 1H), 3.92 (d, *J* = 13.2 Hz, 1H), 2.38 (s, 3H), 1.85 (s, 1H). **^{13}C NMR** (101 MHz, $CDCl_3$)

δ 139.20, 136.82, 133.46, 131.67, 129.82, 129.77, 128.85, 127.28, 118.85, 53.04, 50.55 (t, J = 22.2 Hz), 21.25. **HRMS** (ESI) m/z: [M + H]+ calculated for $C_{16}H_{14}DClN_2$ requires 272.10593; found 272.10571.

2-((4-Chlorobenzyl)amino)-2-(*p*-tolyl)acetic-2-*d* acid (5d)

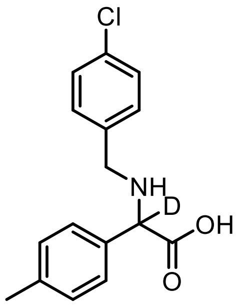

To a stirring solution of 2-((4-chlorobenzyl)amino)-2-(p-tolyl)acetonitrile-*d* (52 mg, 0.19 mmol, 1.0 equiv, 96% d_1) in acetic acid (0.25 mL, 4.40 mmol, 23.0 equiv) was added HCl (1.5 mL, 18 mmol, 96.0 equiv, 37% w/w) and the reaction mixture was stirred at 95 C for 16 hours where it was transferred to a round bottom flask with methanol, concentrated under reduced pressure while triturating with ether (3x) to give 2-((4-chlorobenzyl)amino)-2-(*p*-tolyl)acetic-2-*d* acid, HCl (2x) (**5d**) (70 mg, 0.19 mmol, 100% yield, 96% D), an off-white solid. ^{1}**H NMR** (500 MHz, DMSO) δ 7.54 – 7.48 (m, 3H), 7.47 – 7.43 (m, 4H), 7.41 (s, 1H), 7.30 (s, 1H), 7.27 (d, J = 7.8 Hz, 2H), 5.07 (s, 96% d_1), 4.06 (d, J = 13.2 Hz, 1H), 3.89 (d, J = 13.2 Hz, 1H), 2.32 (s, 3H). ^{13}**C NMR** (126 MHz, DMSO) δ 168.99, 139.21, 133.62, 132.32, 130.63, 129.53, 128.86, 128.83, 128.37, 48.55, 48.04, 40.02, 39.86, 39.69, 39.52, 39.36, 39.19, 39.02, 20.79. **LCMS** (ESI) $C_{16}H_{15}DClNO_2$ requires 290.09, found 291.31 (M/Z + H).

Note: C-D identified from similar structure reported in literature: 2-(benzylamino)-2-(2-methoxyphenyl)acetic acid.[2]

2-(*p*-Tolyl)-2-((4-(trifluoromethyl)phenyl)amino)acetonitrile-*d* (5e)

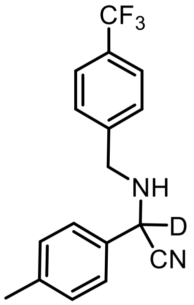

Preparation according to general procedure 5 where [α-D_1]-4-methylbenzaldehyde (1.2 equiv, 36 mg, 0.30 mmol), 4-(trifluoromethyl)phenyl)methanamine (1.0 equiv, 44 mg, 0.25 mmol), acetic acid (1.2 equiv, 18 mg, 0.30 mmol) and *n*-butanol (1.20 mL) were added simultaneously to a sealed and dry 5 mL microwave vial equipped with a magnetic stir bar and stirred for 1 hour at 60 °C. KCN (2.2 equiv, 36 mg, 0.55 mmol) and acetic acid (1.2 equiv, 18 mg, 0.30 mmol) were then added to the reaction mixture and was stirred at 60 °C for 20 hours. The resulting crude residue

was purified by flash column to give title compound **5e** (52 mg, 0.17 mmol, 79% yield, eluting at 15% ethyl acetate/hexanes), a clear oil. **^{1}H NMR** (400 MHz, $CDCl_3$) δ 7.61 (d, *J* = 8.1 Hz, 2H), 7.53 (d, *J* = 8.0 Hz, 2H), 7.42 (d, *J* = 8.1 Hz, 2H), 7.23 (d, *J* = 8.0 Hz, 2H), 5.03 (d, *J* = 7.1 Hz, 95% D), 4.14 – 4.07 (m, 1H), 4.06 – 3.97 (m, 1H), 2.38 (s, 3H), 1.92 (s, 1H). **^{13}C NMR** (101 MHz, $CDCl_3$) δ 142.47, 139.32, 131.53, 129.82, 128.70, 127.30, 125.66 (q, *J* = 3.9 Hz), 122.90, 118.78, 53.20 (t, *J* = 21.9 Hz), 50.71, 21.25. **HRMS** (ESI) m/z: [M + H]+ calculated for $C_{17}H_{14}F_3N_2$ requires 306.13229; found 306.13208.

General Biginelli reaction procedure 6

DMF (0.50 mL, 0.30M), methyl acetoacetate (1.0 equiv, 0.15 mmol), and 4-methylbenzaldehyde (1.0 equiv, 0.15 mmol) were added simultaneously to a sealed MWV containing urea (1.2 equiv, 0.18 mmol). TMSCl (4.0 equiv, 0.60 mmol) was added dropwise, and the reaction was allowed to stir for 48 hours at room temperature. The reaction mixture was quenched with water (1 mL) and sonicated for 5 minutes. The precipitate was filtered, washed with water, and purified by automated column chromatography using a Teledyne ISCO to give the Biginelli reaction product. For a representative example, see **6a**.

Methyl 6-methyl-2-thioxo-4-(*p*-tolyl)-1,2,3,4-tetrahydropyrimidine-5-carboxylate (6a)

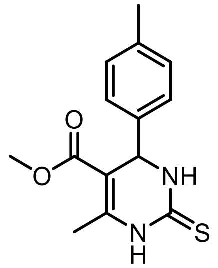

Preparation according to general procedure 6 where DMF (2.08 mL), methyl acetoacetate (1.0 equiv, 0.10 mL, 0.83 mmol), and 4-methylbenzaldehyde (1.0 equiv, 0.10 mL, 0.83 mmol) were added simultaneously to a sealed 5 mL MWV containing thiourea (1.2 equiv, 76 mg, 1.00 mmol). TMS-Cl (4.0 equiv, 0.42 mL, 3.33 mmol) was added dropwise and the reaction mixture was stirred for 48 hours at room temperature. The resulting crude solid was purified by flash column chromatography to give title compound **6a** (146 mg, 0.52 mmol, 63% yield, eluting at 28% ethyl acetate/hexanes), a white solid. **^{1}H NMR** (400 MHz, $CDCl_3$) δ 8.05 (s, 1H), 7.41 (s, 1H), 7.22 – 7.09 (m, 4H), 5.35 (d, *J* = 3.1 Hz, 1H), 3.64 (s, 3H), 2.35 (d, *J* = 0.8 Hz, 3H), 2.32 (s, 3H). **^{13}C NMR** (101 MHz, $CDCl_3$) δ 174.73, 165.88, 142.91, 139.51, 138.39, 129.74, 126.72, 103.04, 55.99, 51.57, 21.27, 18.53. **HRMS** (ESI) m/z: [M + H]+ calculated for $C_{14}H_{16}N_2O_2S$ requires 277.1005; found 277.1006.

Methyl 6-methyl-2-thioxo-4-(*p*-tolyl)-1,2,3,4-tetrahydropyrimidine-5-carboxylate-4-*d* (6b)

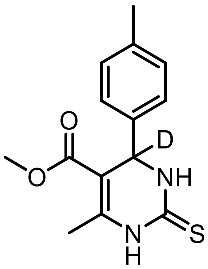

Preparation according to general procedure 6 where DMF (2.08 mL), methyl acetoacetate (1.0 equiv, 0.10 mL, 0.83 mmol), and [α-D_1]-4-methylbenzaldehyde (1.0 equiv, 0.10 mL, 0.83 mmol) were added simultaneously to a sealed 5 mL MWV containing thiourea (1.2 equiv, 76 mg, 1.00 mmol). TMS-Cl (4.0 equiv, 0.42 mL, 3.33 mmol) was added dropwise and the reaction mixture was stirred for 48 hours at room temperature. The resulting crude solid was purified by flash column chromatography give title compound **6b** (142 mg, 0.51 mmol, 61% yield, eluting at 28% ethyl acetate/hexanes), a white solid. **^{1}H NMR** (400 MHz, $CDCl_3$) δ 8.30 (s, 1H), 7.64 (s, 1H), 7.20 – 7.09 (m, 4H), 5.35 (d, *J* = 3.2 Hz, 95% D), 3.64 (s, 3H), 2.35 (s, 3H), 2.32 (s, 3H). **^{13}C NMR** (101 MHz, $CDCl_3$) δ 174.55, 165.89, 143.13, 139.46, 138.32, 129.70, 126.70, 102.90, 55.87, 51.55, 21.26, 18.40. **HRMS** (ESI) m/z: [M + H]+ calculated for $C_{14}H_{15}N_2O_2S$ requires 278.1068; found 278.1064.

Methyl 6-methyl-2-oxo-1-phenyl-4-(*p*-tolyl)-1,2,3,4-tetrahydropyrimidine-5-carboxylate-4-*d* (6c)

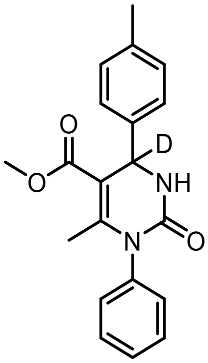

Preparation according to general procedure 6 where DMF (0.60 mL), methyl acetoacetate (1.0 equiv, 46 mg, 0.39 mmol), and [α-D_1]-4-methylbenzaldehyde (1.0 equiv, 50 mg, 39 mmol) were added simultaneously to a sealed 5 mL MWV containing 1-phenylurea (1.2 equiv, 61 mg, 0.45 mmol). TMS-Cl (6.0 equiv, 0.30 mL, 2.4 mmol) was added dropwise and the reaction mixture was stirred for 48 hours at room temperature. The resulting crude solid was purified by flash column chromatography to give title compound **6c** (86 mg, 0.25 mmol, 64% yield), a light-yellow solid. **^{1}H NMR** (400 MHz, $CDCl_3$) δ 7.48 – 7.34 (m, 3H), 7.25 (d, *J* = 8.0 Hz, 2H), 7.19 (s, 2H), 7.13 (d, *J* = 7.8 Hz, 2H), 6.16 (s, 1H), 5.42 (d, *J* = 3.1 Hz, 95% D), 3.65 (s, 3H), 2.34 (s, 3H), 2.08 (s, 3H). **^{13}C NMR** (101 MHz, $CDCl_3$) δ 166.61, 153.47, 149.05, 140.41, 137.67, 137.65, 129.57, 129.31,

128.54, 126.21, 104.92, 53.57 (t, J = 21.0 Hz), 51.48, 21.21, 18.62. **HRMS** (ESI) m/z: [M + H]+ calculated for $C_{20}H_{19}DN_2O_3$ requires 277.1005; found 277.1006.

Methyl 6-methyl-1-phenyl-2-thioxo-4-(*p*-tolyl)-1,2,3,4-tetrahydropyrimidine-5-carboxylate-4-*d* (6d)

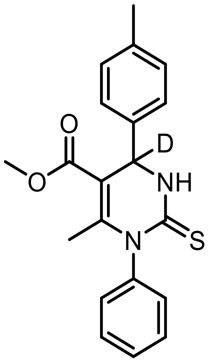

Preparation according to general procedure 6 where DMF (0.60 mL), methyl acetoacetate (1.0 equiv, 46 mg, 0.39 mmol), and [α-D_1]-4-methylbenzaldehyde (1.0 equiv, 50 mg, 39 mmol) were added simultaneously to a sealed 5 mL MWV containing 1-phenylthiourea (1.2 equiv, 69 mg, 0.45 mmol). TMS-Cl (6.0 equiv, 0.30 mL, 2.4 mmol) was added dropwise and the reaction mixture was stirred for 48 hours at room temperature. The resulting crude solid was purified by flash column chromatography to give title compound **6d** (111 mg, 0.31 mmol, 80%, eluting at 22% ethyl acetate/hexanes, 96% deuterated), a white solid. **^{1}H NMR** (400 MHz, $CDCl_3$) δ 7.60 (s, 1H), 7.50 – 7.38 (m, 3H), 7.38 – 7.21 (m, 1H), 7.27 (d, J = 8.2 Hz, 2H), 7.18 (d, J = 7.9 Hz, 2H), 7.15 – 6.90 (m, 1H), 5.45 (s, 96% D) 3.69 (s, 3H), 2.36 (s, 3H), 2.11 (s, 3H). **^{13}C NMR** (101 MHz, $CDCl_3$) δ 166.34, 146.37, 140.73, 139.28, 138.27, 129.84, 128.96, 126.38, 106.91, 51.81, 21.28, 18.86. **HRMS** (ESI) m/z: [M + H]+ calculated for $C_{20}H_{19}N_2O_2S$ requires 354.1381; found 354.1370.

Methyl 1-(4-fluorophenyl)-6-methyl-2-thioxo-4-(*p*-tolyl)-1,2,3,4-tetrahydropyrimidine-5-carboxylate-4-*d* (6e)

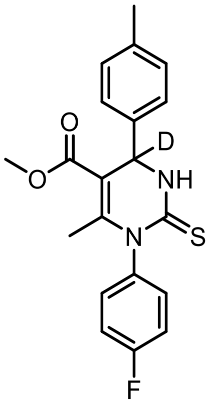

Preparation according to general procedure 6 where DMF (0.30 mL), methyl acetoacetate (1.0 equiv, 23 mg, 0.20 mmol), and [α-D_1]-4-methylbenzaldehyde (1.0 equiv, 26 mg, 0.20 mmol) were added simultaneously to a sealed 5 mL MWV containing 1-(4-fluorophenyl)thiourea (1.15 equiv,

39 mg, 0.23 mmol). TMS-Cl (6.0 equiv, 0.15 mL, 1.2 mmol) was added dropwise and the reaction mixture was stirred for 48 hours at room temperature. The resulting residue was purified by flash column chromatography to give title compound **6e** (61 mg, 0.16 mmol, 82% yield, eluting at 17% ethyl acetate/hexanes, 95% deuterated), a white solid**.** **^{1}H NMR** (400 MHz, $CDCl_3$) δ 8.06 (s, 1H), 7.51 – 7.29 (m, 1H), 7.29 – 7.21 (m, 2H), 7.20 – 7.08 (m, 4H), 7.07 – 6.88 (m, 1H), 5.43 (d, *J* = 3.7 Hz, 95% D), 3.69 (s, 3H), 2.35 (s, 3H), 2.11 (s, 3H). ^{13}C NMR (101 MHz, $CDCl_3$) δ 178.85, 166.22, 163.66, 161.18, 146.21, 139.07, 138.18, 136.53, 136.48, 129.78, 126.28, 107.17, 51.83, 21.24, 18.77. **HRMS** (ESI) m/z: [M + H]+ calculated for $C_{20}H_{18}DFN_2O_2S$ requires 372.1287; found 372.1290. Note: this signal for the deuterated carbon was too weak to be observed in ^{13}C.

Methyl 1-(benzo[d][1,3]dioxol-5-yl)-6-methyl-2-thioxo-4-(*p*-tolyl)-1,2,3,4-tetrahydropyrimidine-5-carboxylate-4-*d* (6f)

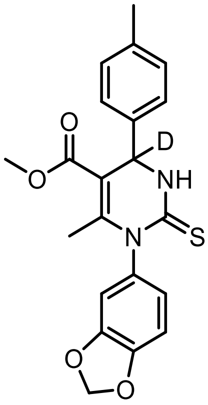

Preparation according to general procedure 6 where DMF (0.32 mL), methyl acetoacetate (1.0 equiv, 23 mg, 0.20 mmol), and [α-D_1]-4-methylbenzaldehyde (1.0 equiv, 26 mg, 0.20 mmol) were added simultaneously to a sealed 5 mL MWV containing 1-([1,3]dioxolo[4,5-*b*]pyridine-5-yl)thiourea (1.15 equiv, 45 mg, 0.23 mmol). TMS-Cl (6.0 equiv, 0.15 mL, 1.2 mmol) was added dropwise and the reaction mixture was stirred for 48 hours at room temperature. The resulting crude solid was purified by flash column chromatography to give title compound **6f** (56 mg, 0.14 mmol, 70% yield, eluting at 26% ethyl acetate/hexanes, 95% deuterated), a white solid. ^{1}H NMR (400 MHz, CHLOROFORM-*D*) δ 7.72 (s, 1H), 7.23 (d, *J* = 8.0 Hz, 2H), 7.17 (d, *J* = 7.9 Hz, 2H), 6.83 (s, 2H), 6.50 (s, 1H), 6.05 (s, 2H), 5.42 (d, *J* = 3.6 Hz, 96% D), 3.69 (s, 3H), 2.34 (s, 3H), 2.16 (s, 3H). **HRMS** (ESI) m/z: [M + H]+ calculated for $C_{21}H_{19}N_2O_4S$ requires 398.1279; found 398.1275.

General Groebke–Blackburn–Bienaymé reaction procedure 7

Toluene (1.95 mL, 0.1M) was added to a dry 5 mL MWV equipped with a magnetic stir bar containing pyrazin-2-amine (1.0 equiv, 0.20 mmol) and aldehyde (1.0 equiv, 0.20 mmol). The reaction mixture was heated to 50 °C and stirred for 50 minutes. Ammonium chloride (2.0 equiv, 0.40 mmol) and isonitrile (1.0 equiv, 0.20 mmol) were added and the reaction mixture was stirred at 110 °C for 36 hours. The reaction mixture was allowed to cool to room temperature, was diluted with DCM, and concentrated under reduced pressure. The resulting crude solid was purified by

automated flash column chromatography using a CombiFlash® to give the desired GBB reaction product. For a representative example, see **7a**.

***N*-([1,1'-Biphenyl]-4-ylmethyl-*d*$_2$)-2-(*p*-tolyl)imidazo[1,2-*a*]pyrazin-3-amine (7a)**

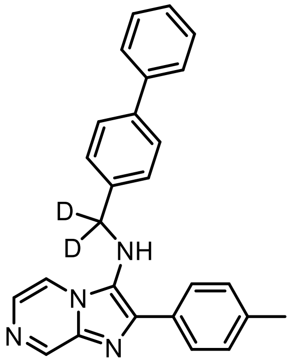

Preparation according to general procedure 7 where a solution of 2-aminopyrazine (1.0 equiv, 19 mg, 0.20 mmol), 4-methylbenzaldehyde (1.0 equiv, 24 mg, 0.20 mmol) in toluene (1.95 mL) was heated at 50 °C for 30 min. Ammonium chloride (2.0 equiv, 21 mg, 0.40 mmol) and 4-(isocyanomethyl-d2)-1,1'-biphenyl (1.0 equiv, 39 mg, 0.20 mmol) were added and the reaction was heated at 110 °C for 36 h. The resulting crude solid was purified by flash column chromatography to give title compound **7a** (14 mg, 0.03 mmol, 18% yield, eluting at 5% methanol in DCM), a yellow solid. **^{1}H NMR** (400 MHz, $CDCl_3$) δ 8.44 (dd, *J* = 4.1, 2.0 Hz, 1H), 8.17 (dd, *J* = 6.7, 1.9 Hz, 1H), 7.98 (d, *J* = 8.2 Hz, 2H), 7.61 – 7.49 (m, 4H), 7.44 (t, *J* = 7.3 Hz, 2H), 7.41 – 7.32 (m, 3H), 7.28 (d, *J* = 13.3 Hz, 3H), 6.71 (dd, *J* = 6.8, 4.1 Hz, 1H), 4.22 (d, *J* = 4.3 Hz, 70% D_2), 2.41 (s, 3H). **HRMS** (ESI) m/z: [M + H]+ calculated for $C_{26}H_{20}D_2N_4$ requires 392.1980, found 392.1981.

***N*-([1,1'-Biphenyl]-4-ylmethyl-*d*$_2$)-2-(4-fluorophenyl)imidazo[1,2-*a*]pyrazin-3-amine (7b)**

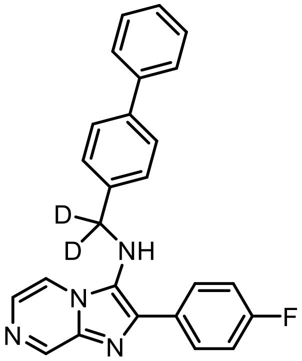

Preparation according to general procedure 7 where a solution of 2-aminopyrazine (1.0 equiv, 19 mg, 0.20 mmol), 4-fluorobenzaldehyde (1.0 equiv, 25 mg, 0.20 mmol) in toluene (1.95 mL) was heated at 50 °C for 30 min. Ammonium chloride (2.0 equiv, 21 mg, 0.40 mmol) and 4-(isocyanomethyl-d2)-1,1'-biphenyl (1.0 equiv, 39 mg, 0.20 mmol) were added and the reaction was heated at 110 °C for 36 h. The resulting crude solid was purified by flash column chromatography to give title compound **7b** (28 mg, 0.07 mmol, 35%, eluting at 5% methanol in

DCM), a brown solid. **^{1}H NMR** (400 MHz, $CDCl_3$) δ 8.44 (dd, *J* = 4.1, 2.0 Hz, 1H), 8.16 (dd, *J* = 6.8, 0.9 Hz, 1H), 8.06 (dd, *J* = 8.8, 5.5 Hz, 2H), 7.61 – 7.39 (m, 6H), 7.40 – 7.28 (m, 3H), 7.13 (t, *J* = 8.7 Hz, 2H), 6.72 (dd, *J* = 6.7, 4.0 Hz, 1H), 4.21 (d, *J* = 5.8 Hz, 2D, 70% D2), 3.57 (d, *J* = 6.4 Hz, 1H). **^{13}C NMR** (101 MHz, $CDCl_3$) δ 163.8, 161.4, 149.4, 144.6, 140.9, 140.7 (d, *J* = 38.0 Hz), 129.9, 129.1 (d, *J* = 8.1 Hz), 128.9, 128.7, 127.5, 127.0, 123.4, 115.7 (d, *J* = 21.6 Hz), 108.0. **HRMS** (ESI) m/z: [M + H]+ calculated for $C_{25}H_{17}D_2FN_4$ requires 396.1729, found 396.1727. Note: The deuterated carbon signal was too weak to be observed.

2-(*p*-Tolyl)-*N*-(*p*-tolylmethyl-*d*)imidazo[1,2-*a*]pyrazin-3-amine (7c)

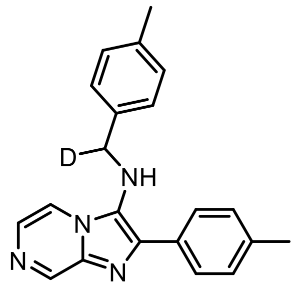

Preparation according to general procedure 7 where a solution of 2-aminopyrazine (1.0 equiv, 19 mg, 0.20 mmol), 4-methylbenzaldehyde (1.0 equiv, 24 mg, 0.20 mmol) in toluene (1.95 mL) was heated at 50 C for 30 min. Ammonium chloride (2.0 equiv, 21 mg, 0.40 mmol) and 1-(isocyanomethyl-*d*)-4-methylbenzene (1.0 equiv, 26 mg, 0.20 mmol) were added and the reaction mixture was heated at 110 °C for 36 h. The resulting crude solid was purified by automated flash column chromatography using a CombiFlash® to give title compound **7c** (53 mg, 0.16 mmol, 80%, eluting at 100% ethyl acetate, 96% deuterated), a yellow solid. **^{1}H NMR** (400 MHz, $CDCl_3$) δ 8.94 (d, *J* = 1.5 Hz, 1H), 7.83 (d, *J* = 7.9 Hz, 2H), 7.79 (dd, *J* = 4.6, 1.4 Hz, 1H), 7.74 (d, *J* = 4.6 Hz, 1H), 7.26 (d, *J* = 8.4 Hz, 2H), 7.19 – 7.14 (m, 2H), 7.11 (d, *J* = 7.9 Hz, 2H), 5.12 (s, 96% D), 4.14 (d, *J* = 5.7 Hz, 1H), 3.61 (d, *J* = 6.2 Hz, 1H), 2.41 (s, 3H), 2.33 (s, 3H). **^{13}C NMR** (101 MHz, $CDCl_3$) δ 143.21, 138.79, 138.28, 137.74, 136.69, 135.57, 130.49, 129.65, 129.56, 128.93, 128.17, 127.24, 126.99, 115.32, 51.68 (t, *J* = 20.7 Hz), 51.47, 21.43, 21.22. **HRMS** (ESI) m/z: [M + H]+ calculated for $C_{21}H_{19}DN_4$ requires 330.1823; found 330.1816.

Asymmetric Hantzsch dihydropyridine synthesis

2-(Benzyl(methyl)amino)ethyl 3-oxobutanoate (8a1)[1]

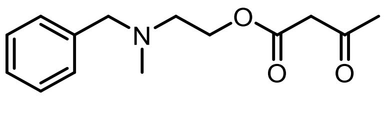

Triethylamine (1.69 mL, 12.10 mmol, 2.0 equiv), 2-(benzyl(methyl)amino)ethan-1-ol (0.98 mL, 6.05 mmol, 1.0 equiv), and toluene (8.50 mL) were added to a 20 mL MWV equipped with a magnetic stir bar and was stirred for 5 minutes at room temperature. Ethyl acetoacetate (0.85 mL, 6.65 mmol, 1.1 equiv) was added and the reaction mixture was stirred at 110 °C for 24 hours. The reaction mixture was cooled down, concentration under reduced pressure, and purified by flash column chromatography to **8a1** (486 mg, 1.95 mmol, 32% yield). **^{1}H NMR** (400 MHz, $CDCl_3$) δ 7.38 – 7.23 (m, 5H), 4.29 (t, *J* = 5.8 Hz, 2H), 3.57 (s, 2H), 3.48 (s, 2H), 2.70 (t, *J* = 5.9 Hz, 2H),

2.29 (d, J = 4.1 Hz, 6H). Note: 2-(benzyl(methyl)amino)ethan-1-ol (263 mg, 2.02 mmol, 33%) was recovered. [1]Commercially available from several vendors. Fischer J, Ganellin CR (2006). Analogue-based Drug Discovery. John Wiley & Sons. p. 464. ISBN 978-3-527-60749-5.

3-(2-(Benzyl(methyl)amino)ethyl) 5-methyl 2,6-dimethyl-4-(3-nitrophenyl)-1,4-dihydropyridine-3,5-dicarboxylate (H-nicardipine, 8a)

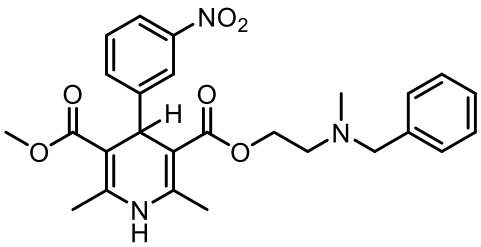

2-(Benzyl(methyl)amino)ethyl 3-oxobutanoate (500 mg, 2.01 mmol, 1.0 equiv) and 2-propanol (6.00 mL) were added to a sealed 20 mL MWV equipped with a magnetic stir bar containing methyl (Z)-3-aminobut-2-enoate (231 mg, 2.01 mmol, 1.0 equiv) and 3-nitrobenzaldehyde (303 mg, 2.01 mmol, 1.0 equiv). The reaction mixture was stirred at 83 °C for 4 hours where it was cooled down room temperature, diluted with DCM, concentrated under reduced pressure, and purified by flash column chromatography to give **8a** (560 mg, 58% yield, 1.17 mmol, eluting at 43% ethyl acetate in hexanes), a yellow oil. **^{1}H NMR** (400 MHz, $CDCl_3$) δ 8.12 (t, J = 2.0 Hz, 1H), 7.99 (ddd, J = 8.2, 2.4, 1.1 Hz, 1H), 7.67 (dt, J = 7.7, 1.4 Hz, 1H), 7.39 – 7.22 (m, 6H), 5.84 (s, 1H), 5.15 (s, 1H), 4.21 (t, J = 6.0 Hz, 2H), 3.67 (s, 3H), 3.58 – 3.52 (m, 2H), 2.70 (dd, J = 12.7, 6.3 Hz, 2H), 2.40 (s, 3H), 2.39 (s, 3H), 2.24 (s, 3H). **^{13}C NMR** (101 MHz, $CDCl_3$) δ 167.62, 167.08, 149.71, 148.46, 145.01, 134.51, 129.09, 128.82, 128.44, 128.40, 122.97, 121.53, 103.35, 62.62, 55.66, 51.28, 39.81, 19.86, 19.81, 14.34. **LCMS** (ESI) $C_{26}H_{29}N_3O_6$ requires 479.20, found 480.2 (M/Z + H).

3-(2-(Benzyl(methyl)amino)ethyl) 5-methyl 2,6-dimethyl-4-(3-nitrophenyl)-1,4-dihydropyridine-3,5-dicarboxylate-4-*d* (D-nicardipine, 8b)

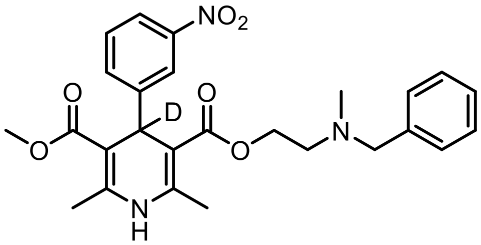

2-(Benzyl(methyl)amino)ethyl 3-oxobutanoate (328 mg, 1.31 mmol, 1.0 equiv) and 2-propanol (6.00 mL) were added to a sealed 20 mL MWV equipped with a magnetic stir bar containing methyl (Z)-3-aminobut-2-enoate (151 mg, 1.31 mmol, 1.0 equiv) and [α-D_1]-3-nitrobenzaldehyde (200 mg, 1.31 mmol, 1.0 equiv). The reaction mixture was stirred at 83 °C for 4 hours and was cooled down to room temperature. The reaction mixture was diluted with DCM, concentrated under reduced pressure, and purified by flash column chromatography to give 3-(2-(benzyl(methyl)amino)ethyl) 5-methyl 2,6-dimethyl-4-(3-nitrophenyl)-1,4-dihydropyridine-3,5-

dicarboxylate-4-d (**D-nicardipine**) (369 mg, 0.76 mmol, 69% yield, eluting at 45% ethyl acetate in hexanes, 95% D), a yellow oil. ^{1}H NMR (400 MHz, $CDCl_3$) δ 8.12 (t, *J* = 2.0 Hz, 1H), 7.99 (ddd, *J* = 8.2, 2.4, 1.1 Hz, 1H), 7.67 (dt, *J* = 7.8, 1.3 Hz, 1H), 7.40 – 7.20 (m, 6H), 5.86 (s, 1H), 5.15 (s, 0H), 4.21 (t, *J* = 6.0 Hz, 2H), 3.67 (s, 3H), 3.54 (d, *J* = 2.2 Hz, 2H), 2.69 (hept, *J* = 6.4 Hz, 2H), 2.40 (s, 3H), 2.39 (s, 3H), 2.24 (s, 3H). **^{13}C NMR** (101 MHz, $CDCl_3$) δ 167.62, 167.09, 149.66, 148.45, 145.20, 145.05, 134.49, 129.07, 128.82, 128.39, 127.22, 122.95, 121.53, 103.26, 62.64, 61.88, 55.67, 51.27, 42.40, 19.83, 19.78. **LCMS** (ESI) $C_{26}H_{28}DN_3O_6$ requires 480.21, found 481.1 (M/Z + H).

3-(2-(Benzyl(methyl)amino)ethyl) 5-isopropyl 2,6-dimethyl-4-(3-nitrophenyl)-1,4-dihydropyridine-3,5-dicarboxylate (H-iPr-nicardipine, 8c)

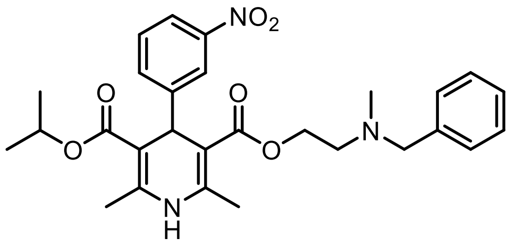

Isopropyl 3-aminocrotonate (251 mg, 1.75 mmol, 1.0 equiv), 2-(benzyl(methyl)amino)ethyl 3-oxobutanoate (437 mg, 1.75 mmol, 1.0 eq), and 2-propanol (5.30 mL) were added to a sealed 20 mL MWV equipped with a magnetic stir bar containing 3-nitrobenzaldehyde (265 mg, 1.75 mmol, 1.0 equiv). The reaction mixture was stirred at 83 °C for 3 hours. The reaction mixture was allowed to cool to room temperature and was concentrated under reduced pressure. The crude residue was purified by flash column chromatography to give **H-iPr-nicardipine (8c)** (570 mg, 1.12 mmol, 64% yield), a yellow oil. **^{1}H NMR** (500 MHz, $CDCl_3$) δ 8.13 (t, *J* = 2.0 Hz, 1H), 7.98 (ddd, *J* = 8.1, 2.4, 1.1 Hz, 1H), 7.65 (dt, *J* = 7.6, 1.4 Hz, 1H), 7.35 – 7.26 (m, 5H), 7.26 – 7.21 (m, 1H), 5.66 (s, 1H), 5.10 (s, 1H), 4.95 (hept, *J* = 6.3 Hz, 1H), 4.17 (t, *J* = 6.0 Hz, 2H), 3.50 (d, *J* = 2.5 Hz, 2H), 2.63 (q, *J* = 6.2 Hz, 2H), 2.36 (s, 3H), 2.36 (s, 3H), 2.21 (s, 3H), 1.24 (d, *J* = 6.3 Hz, 3H), 1.09 (d, *J* = 6.3 Hz, 3H). **^{13}C NMR** (126 MHz, $CDCl_3$) δ 167.18, 166.61, 149.98, 148.28, 145.03, 144.41, 139.01, 134.74, 129.06, 128.70, 128.37, 127.16, 123.32, 121.46, 104.02, 103.34, 67.49, 62.68, 62.12, 55.74, 42.56, 40.08, 22.25, 21.94, 19.96, 19.79. **LCMS** (ESI) $C_{28}H_{33}N_3O_6$ requires 507.23, found 508.3 (M/Z + H).

3-(2-(Benzyl(methyl)amino)ethyl) 5-isopropyl 2,6-dimethyl-4-(3-nitrophenyl)-1,4-dihydropyridine-3,5-dicarboxylate-4-d (D-iPr nicardipine, 8d)

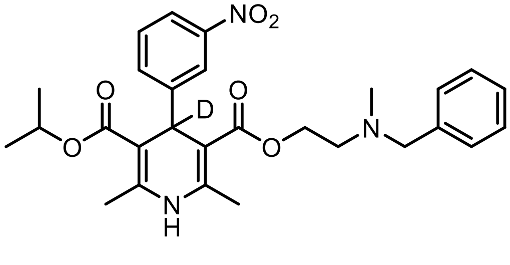

Isopropyl 3-aminocrotonate (188 mg, 1.31 mmol, 1.0 equiv), 2-(benzyl(methyl)amino)ethyl 3-oxobutanoate (328 mg, 1.31 mmol, 1.0 equiv), and 2-propanol (4.00 mL) were added 8 mL MWV equipped with a magnetic stir bar containing 3-nitrobenzaldehyde-α-D_1 (200 mg, 1.31 mmol, 1.0 equiv).The reaction mixture was sti The reaction mixture was stirred at 83 °C for 3 hours. The reaction mixture was allowed to cool to room temperature and was concentrated under reduced pressure. The crude residue was purified by flash column chromatography to give **D-iPr nicardipine, 8d** (441 mg, 0.86 mmol, 66% yield, 96% D), a yellow semisolid. ^{1}H NMR (400 MHz, $CDCl_3$) δ 8.12 (t, *J* = 2.0 Hz, 1H), 7.97 (ddd, *J* = 8.2, 2.3, 1.1 Hz, 1H), 7.65 (ddd, *J* = 7.7, 1.7, 1.1 Hz, 1H), 7.37 – 7.21 (m, 6H), 5.77 (s, 1H), 5.10 (s, 96% D), 4.95 (hept, *J* = 6.3 Hz, 1H), 4.19 (t, *J* = 6.0 Hz, 2H), 3.53 (s, 2H), 2.74 – 2.60 (m, 2H), 2.37 (s, 6H), 2.23 (s, 3H), 1.25 (d, *J* = 6.2 Hz, 3H), 1.10 (d, *J* = 6.2 Hz, 3H). ^{13}C NMR (101 MHz, $CDCl_3$) δ 166.61, 157.66, 149.92, 148.26, 144.49, 134.69, 129.15, 128.72, 128.45, 128.41, 123.25, 121.45, 103.90, 67.49, 62.55, 60.55, 55.66, 42.42, 22.24, 21.93, 19.90, 19.72. **LCMS** (ESI) $C_{28}H_{32}DN_3O_6$ requires 508.24, found 509.3 (M/Z + H).

3-Isopropyl 5-(2-methoxyethyl) 2,6-dimethyl-4-(3-nitrophenyl)-1,4-dihydropyridine-3,5-dicarboxylate (H-nimodipine, 8e)

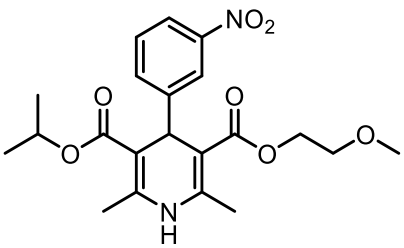

Isopropyl 3-aminocrotonate (600 mg, 4.19 mmol, 1.0 equiv), 2-methoxyethl 3-oxobutanoate (0.61 mL, 4.19 mmol, 1.0 equiv), and 2-propanol (12.5 mL) were added to a sealed 20 mL MWV equipped with a magnetic stir bar containing 3-nitrobenzaldehyde (633 mg, 4.19 mmol, 1.0 equiv). The reaction mixture was stirred at 83 °C for 3 hours. The reaction mixture was allowed to cool to room temperature and was concentrated under reduced pressure. The crude residue was purified by flash column chromatography to **H-nimodipine** (1.43 g, 3.42 mmol, 82% yield, eluting at 21% ethyl acetate in hexanes), a yellow oil. ^{1}H NMR (400 MHz, $CDCl_3$) δ 8.13 (t, *J* = 2.0 Hz, 1H), 7.99 (ddd, *J* = 8.2, 2.3, 1.1 Hz, 1H), 7.66 (dt, *J* = 7.8, 1.4 Hz, 1H), 7.36 (t, *J* = 7.9 Hz, 1H), 5.75 (s, 1H), 5.10 (s, 1H), 4.94 (hept, *J* = 6.2 Hz, 1H), 4.25 – 4.07 (m, 2H), 3.61 – 3.47 (m, 2H), 3.35 (s, 3H), 2.36 (s, 6H), 1.26 (d, *J* = 6.3 Hz, 3H), 1.09 (d, *J* = 6.2 Hz, 3H). **^{13}C NMR** (101 MHz, $CDCl_3$) δ 167.14, 166.60, 150.08, 148.22, 145.18, 144.41, 134.83, 128.67, 123.41, 121.42, 104.05, 103.24, 70.62, 67.47, 63.11, 58.98, 40.19, 22.22, 21.90, 19.85, 19.69. **LCMS** (ESI) $C_{21}H_{26}N_2O_7$ requires 418.17, found 419.3 (M/Z + H).

3-Isopropyl 5-(2-methoxyethyl) 2,6-dimethyl-4-(3-nitrophenyl)-1,4-dihydropyridine-3,5-dicarboxylate-4-*d* (D-nimodipine, 8f)

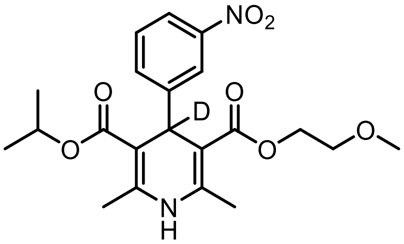

Isopropyl 3-aminocrotonate (0.20 mL, 1.40 mmol, 1.0 equiv) and 2-propanol (5.00 mL) were added to a sealed 20 mL MWV equipped with a magnetic stir bar containing 3-nitrobenzaldehyde-α-D_1 (212 mg, 1.40 mmol, 1.0 equiv) and isopropyl (E)-3-aminobut-2-enoate (200 mg, 1.40 mmol, 1.0 equiv). The reaction mixture was stirred at 83 °C for 3 hours where it was allowed to cool to room temperature and then was concentrated under reduced pressure. The crude residue was purified by flash column chromatography to give **D-nimodipine** (304 mg, 0.72 mmol, 52% yield, eluting at 32% ethyl acetate in hexanes, 95% D), a yellow oil. **^{1}H NMR** (400 MHz, $CDCl_3$) δ 8.13 (t, J = 2.0 Hz, 1H), 7.99 (ddd, J = 8.2, 2.3, 1.1 Hz, 1H), 7.66 (dt, J = 7.6, 1.4 Hz, 1H), 7.36 (t, J = 7.9 Hz, 1H), 5.77 (s, 1H), 5.09 (s, 95% D), 4.94 (hept, J = 6.2 Hz, 1H), 4.24 – 4.07 (m, 2H), 3.61 – 3.46 (m, 2H), 3.35 (s, 3H), 2.36 (s, 6H), 1.26 (t, J = 6.2 Hz, 3H), 1.09 (d, J = 6.3 Hz, 3H). **^{13}C NMR** (101 MHz, $CDCl_3$) δ 167.14, 166.60, 150.03, 148.22, 145.22, 144.45, 134.80, 128.68, 123.39, 121.42, 103.97, 103.15, 70.62, 67.46, 63.11, 58.98, 22.23, 21.91, 19.83, 19.67. **LCMS** (ESI) $C_{21}H_{25}DN_2O_7$ requires 419.18, found 420.3 (M/Z + H).

General deuterated isocyanide reaction procedure 8

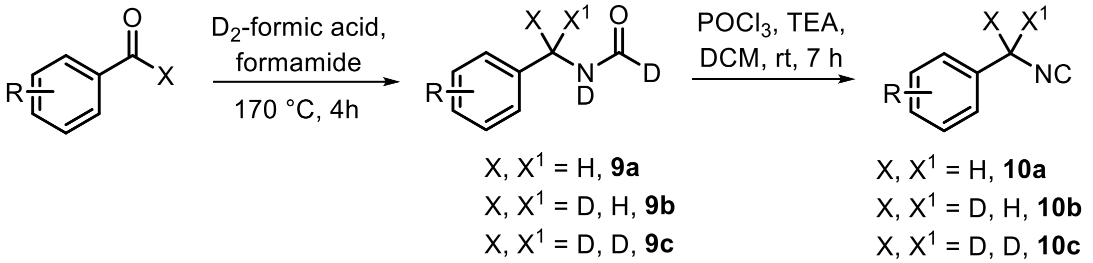

8a: 9a/b, [D$_1$]-Formamide: To a 5 mL MWV equipped with a magnetic stir bar containing d_1-aldehyde (1.0 equiv, 1.0 mmol) was added formamide (12.0 equiv, 12.0 mmol) and formic acid (6.6 equiv, 6.6 mmol). The reaction mixture was stirred at 170 °C for 2 hours and was then cooled to room temperature, diluted with DCM, and concentrated under reduced pressure. The crude solid was washed with saturated sodium bicarbonate and extracted with DCM (3 × 10 mL). The organic layers were recombined dried over sodium sulfate, filtered, and concentrated under reduced pressure. The resulting crude residue was purified by flash column chromatography to give the deuterated formamide intermediate.

8b: 9c, D$_2$-Formamide, optimal conditions: To a 5 mL MWV equipped with a magnetic stir bar containing [1,1'-biphenyl]-4-carbaldehyde-d (1.0 equiv, 1.0 mmol) and d_2-formic acid (20.0 equiv, 20.0 mmol) was added formamide (1.0 equiv, 1.0 mmol). The reaction mixture was stirred at 170

°C for 5 minutes in a microwave reactor where it was then allowed to cool to room temperature, diluted with DCM, ad concentrated under reduced pressure. The crude solid was washed with saturated sodium bicarbonate and extracted with DCM (3 × 10 mL). The organic layers were recombined, dried over sodium sulfate, filtered, and concentrated under reduced pressure. The resulting crude residue was purified by flash column chromatography to give the deuterated formamide intermediate.

8c: 10a-c, $D_{1/2}$-Isocyanide: DCM (10.00 mL, 0.15M) and triethylamine (2.7 equiv, 2.7 mmol) were added to a 250 mL 3 neck RBF, equipped with an addition funnel, argon balloon, and a magnetic stir bar was added deuterated formamide intermediate (1.0 equiv, 1.0 mmol). The solution was cooled to 0 °C where phosphorous oxychloride (1.2 equiv, 1.2 mmol) was added dropwise over 15 minutes. The reaction was stirred for 7 hours at room temperature where 25 mL of saturated sodium bicarbonate was carefully added to the reaction mixture with vigorous stirring for 15 minutes. The reaction mixture was extracted with DCM (3 × 15 mL) and washed with water. The organic layers were recombined, dried over sodium sulfate, filtered, and concentrated under reduced pressure. The crude residue was purified by flash column chromatography to give the desired $D_{1/2}$-Isocyanide.

***N*-(*p*-Tolylmethyl-*d*)formamide (9ba)**

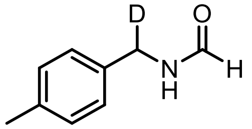

Preparation according to general procedure 8a where formic acid (6.6 equiv, 1.4 mL, 37 mmol) and formamide (12 equiv, 2.66 mL, 66.9 mmol) were added to a dry 20 mL MWV equipped with a magnetic stir bar containing [α-D_1]-4-methylbenzaldehyde (1.0 equiv, 0.73 mL, 5.57 mmol, 98% D). The resulting crude residue was purified by flash column chromatography to give title compound (541 mg, 3.60 mmol, 64%, eluting at 50% ethyl acetate in hexanes, 98% D), a white solid. **^{1}H NMR** (400 MHz, $CDCl_3$) δ 8.24 (s, 1H), 7.21 – 7.10 (m, 4H), 5.91 (s, 1H), 4.43 (bs, 1H, 96% d_1), 2.34 (s, 3H), *minor rotamer*: 8.16 (d, J = 11.7 Hz, 1H), 4.36 (s, 98% d_1), 2.35 (s, 3H). **^{13}C NMR** (101 MHz, $CDCl_3$) δ 161.1, 137.6, 134.6, 129.7, 129.6, 128.0, 127.1, 41.9 (t, J = 21.1 Hz), 21.2.

1-(Isocyanomethyl-*d*)-4-methylbenzene (10ba)

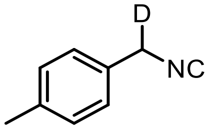

Preparation according to general procedure 8c where DCM (33.0 mL) and triethylamine (1.7 mL, 12.6 mmol) was added to a dry 250 mL 3 neck RBF under an atmosphere of argon equipped with an addition funnel, and magnetic stir bar The reaction mixture was cooled to 0 °C where phosphorous oxychloride was added dropwise over 15 minutes. The reaction mixture was then stirred for 7 hours at room temperature. The resulting crude residue was purified by flash column

chromatography to give title compound (319 mg, 2.41 mmol, 46%, eluting at 100% DCM, 98% D), an orange liquid. Note: Some isocyanide product was lost during the concentration under reduced pressure phase due to its low boiling point (~80 °C). For higher yields it would be recommended to evaporate off solvent in ice. **^{1}H NMR** (400 MHz, $CDCl_3$) δ 7.30 – 7.17 (m, 4H), 4.59 (bs, 1H, 98% D), 2.37 (s, 3H). **^{13}C NMR** (101 MHz, $CDCl_3$) δ 157.4, 138.4, 129.7, 129.50, 126.8, 45.5, 21.2.

***N*-([1,1'-Biphenyl]-4-ylmethyl)formamide (9aa)**

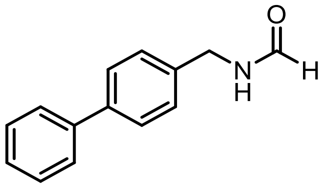

Preparation according to 8a where formamide was added to a dry 5 mL MWV equipped with a magnetic stir bar containing [1,1'-biphenyl]-4-carbaldehyde (150 mg, 0.82 mmol) and was stirred at 170 °C for 1 hour. Formic acid-d_2 (0.22 mL, 5.93 mmol) was added to the reaction mixture and was stirred at 170 C for 2 hours. The resulting crude residue was purified by flash column chromatography to give title compound (93 mg, 0.44 mmol, 53%, eluting at 100% ethyl acetate in hexanes), a white solid. **^{1}H NMR** (400 MHz, $CDCl_3$) δ 8.30 (dt, J = 1.7, 0.8 Hz, 1H), 7.60 – 7.54 (m, 4H), 7.50 – 7.40 (m, 2H), 7.40 – 7.31 (m, 3H), 5.88 (s, 1H), 4.54 (dd, J = 5.9, 0.8 Hz, 2H). **^{13}C NMR** (101 MHz, $CDCl_3$) δ 161.07, 140.89, 140.73, 136.72, 128.96, 128.42, 127.67, 127.58, 127.21, 42.05. [2]Dhake, K. P., Tambade, P. J., Singhal, R. S., & Bhanage, B. M. (2011). An efficient, catalyst- and solvent-free N-formylation of aromatic and aliphatic amines. Green Chemistry Letters and Reviews, 4(2), 151–157.

4-(Isocyanomethyl)-1,1'-biphenyl (10aa)

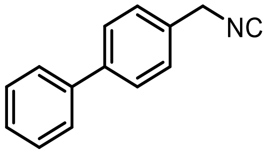

Preparation according to general procedure[3] 8c where DCM (11.70 mL) and triethylamine (0.35 mL, 2.56 mmol) was added to a dry 250 mL 3 neck RBF under an atmosphere of argon equipped with an addition funnel, and magnetic stir bar The reaction mixture was cooled to 0 °C where phosphorous oxychloride was added dropwise over 15 minutes. The reaction mixture was then stirred for 7 hours at room temperature. The resulting crude residue was purified via flash column chromatography to give title compound (319 mg, 2.41 mmol, 46%, eluting at 100% DCM, 98% D), an orange liquid. **^{1}H NMR** (400 MHz, $CDCl_3$) δ 7.67 – 7.55 (m, 4H), 7.51 – 7.41 (m, 4H), 7.41 – 7.34 (m, 1H), 4.70 (t, J = 2.1 Hz, 2H). **^{13}C NMR** (101 MHz, $CDCl_3$) δ 141.65, 140.41, 131.42, 129.02, 127.84, 127.80, 127.26, 127.23, 45.45 (t, J = 7.1 Hz).

N-([1,1'-Biphenyl]-4-ylmethyl-d_2)formamide-1-d (9ca)

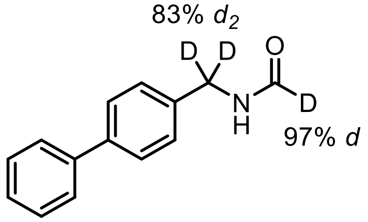

Preparation according to 8b where formamide (12 mg, 0.27 mmol) was added to a dry 1 mL MWV containing [1,1'-biphenyl]-4-carbaldehyde-d (50 mg, 0.27 mmol, 95% d_1). The reaction mixture was stirred at 170 °C for 1 hour where formic acid-d_2 was added and the reaction was stirred at 170 C for hours. The resulting crude residue was purified by automated flash column chromatography using a CombiFlash® to give title compound (16 mg, 0.75 mmol, 27%, 83% d_2, eluting at 23% ethyl acetate in hexanes), a white solid. **^{1}H NMR** (400 MHz, $CDCl_3$) δ 8.50 (s, 1D, 97% D), 7.68 – 7.57 (m, 4H), 7.52 – 7.45 (m, 2H), 7.43 – 7.36 (m, 1H), 7.31 (ddt, J = 10.5, 8.6, 2.3 Hz, 2H), 4.53 (s, 1D, 90% D), 4.38 (s, 1D, 90% D). **^{13}C NMR** (101 MHz, $CDCl_3$) δ 161.2, 141.1, 140.8, 140.7, 136.7, 136.7, 129.0, 128.9, 128.4, 127.7, 127.6, 127.6, 127.5, 127.2, 41.9 (t, J = 5.7 Hz).

4-(Isocyanomethyl-d_2)-1,1'-biphenyl (10ca)

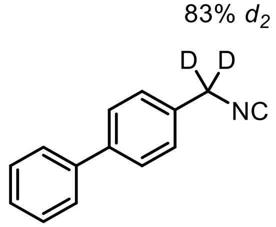

Preparation according to general procedure 8c where DCM (1.80 mL) and triethylamine (0.10 mL, 0.70 mmol) was added to a dry 5 mL MWV equipped with a stir bar containing *N*-([1,1'-biphenyl]-4-ylmethyl-d_2)formamide (55 mg, 0.26 mmol, ~80% d_2). The reaction mixture was cooled to 0 °C where phosphorous oxychloride (0.03 mL, 0.31 mmol) was added dropwise over 15 minutes. The reaction mixture was then stirred for 6.5 hours at room temperature. The resulting crude residue was purified by flash column chromatography to give title compound (46 mg, 0.26 mmol, 91%, eluting at 11% ethyl acetate in hexanes, 83% d_2), a white solid. **^{1}H NMR** (400 MHz, $CDCl_3$) δ 7.68 – 7.56 (m, 4H), 7.52 – 7.35 (m, 5H), 4.68 (bs, 83% d_2). **^{13}C NMR** (101 MHz, $CDCl_3$) δ 157.95 (t, J = 5.4 Hz), 141.59, 140.35, 131.29, 128.98, 127.78, 127.76, 127.23, 127.21. Note: formamide deuteration was harder to discern than isocyanide deuteration, thus it was determined that given that 10ca is 83% deuterated, the formed formamide must also be 83% deuterated.

N-((3-Nitrophenyl)methyl-*d2*)formamide-1-*d* (9cb)

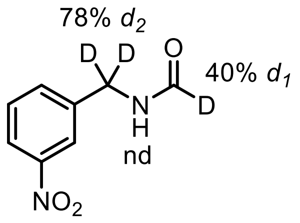

Formamide (11 µL, 0.28 mmol, 2.0 equiv) was added to a 5 mL MWV equipped with a magnetic stir bar containing 3-nitrobenzaldehyde-α_1-d1 (21 mg, 0.14 mmol, 1.0 equiv, 98% D) and the reaction mixture was stirred in a microwave reactor at 180 °C for 5 minutes to give N-((3-nitrophenyl)methyl-d2)formamide-N,1-d2 (21 mg, 0.11 mmol, 83% yield, 78% D). ^{1}**H NMR** (400 MHz, DMSO-D_6) δ 8.61 (s, 1H), 8.17 – 7.97 (m, 2H), 7.75 – 7.64 (m, 1H), 7.63 – 7.53 (m, 1H), 4.64 – 4.46 (m, 90% d_1), 4.38 (d, J = 6.4 Hz, 88% d_1).

N-([1,1'-Biphenyl]-4-ylmethyl)formamide-1-*d* (9ab)

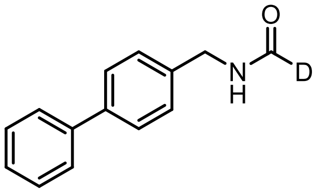

Formic acid-d_2 (47 µL, 1.2 mmol, 10.0 equiv) was added to a 5 mL MWV equipped with a magnetic stir bar containing *N*-([1,1'-biphenyl]-4-ylmethyl)formamide (25 mg, 0.12 mmol, 1.0 equiv). The reaction mixture was stirred at 170 °C for 8 hours where it was cooled down to room temperature, diluted with DCM, washed with saturated sodium bicarbonate, and extracted with DCM (3 × 10 mL). The organic layers were recombined, dried over sodium sulfate, and purified by flash column chromatography to give *N*-([1,1'-biphenyl]-4-ylmethyl)formamide-1-d (25 mg, 0.12 mmol, >99% yield, 98% D), a white solid. ^{1}**H NMR** (400 MHz, $CDCl_3$) δ 8.31 (s, 98% *d*), 7.70 – 7.54 (m, 4H), 7.54 – 7.43 (m, 2H), 7.42 – 7.31 (m, 3H), 6.00 (s, 1H), 4.58 – 4.44 (m, 2H). ^{13}**C NMR** (101 MHz, $CDCl_3$) δ 140.84, 140.70, 136.72, 128.95, 128.39, 127.77, 127.64, 127.56, 127.19, 41.97.

Mouse liver microsome stability

5 µL of compound (working solution) and control stock solution (10 mM in dimethyl sulfoxide) were diluted with 495 µL of acetonitrile (intermediate solution concentration: 100 µM, 99% acetonitrile). NADPH Cofactor was prepared where the appropriate amount of NADPH was weighed out and diluted into a 10 mM $MgCl_2$ solution (working solution concentration: 10 mM; final concentration in reaction system: 1 mM). The appropriate concentrations of CD-1 mouse liver microsomes were prepared in 100 mM potassium phosphate buffer. Cold (4 C) acetonitrile containing 200 ng/mL tolbutamide and 200 ng/mL labetalol as internal standards (IS) were used as the stop solution. From here, incubation plates T60 and NCF60 were pre-warmed for 10 minutes. Liver microsomes were diluted to 0.56 mg/mL in 100 mM phosphate buffer. 445 µL of microsome working solutions were transferred into pre-warmed incubation plates and pre-

incubated for 10 minutes at 37 °C with constant shaking. 54 µL of liver microsomes were transferred to blank plate, then 6 µL of NADPH cofactor and 180 µL quenching solution were added to the blank late. From here, 5 µL of compound working solution (100 µM) was added to incubation plates containing microsomes and were mixed thoroughly. To the NCF60 plate was added 50 µL of buffer and was mixed thoroughly. The plate was incubated at 37 °C for 60 minutes with shaking. To quenching plate T0 was added 180 µL quenching solution and 6 µL NAPDH cofactor, the plate was chilled to prevent evaporation. The T60 plate was mixed thoroughly and 54 µL of mixture was removed immediately for the 0-min time point to quenching plate. From here, 44 µL of NADPH cofactor was added to incubation plate and the plate was incubated at 37 C for 60 minutes while shaking. At 5, 15, 30, 45, and 60 minutes, 180 µL of quenching solution was added and serially transferred 60 µL of sample from T60 plate per time point to quenching plates. The NCF60 plate was mixed once and 60 µL of sample from the NCF60 incubation was transferred to quenching plate containing quenching solution at the 60-minute time point. All sampling plates were shaken for 10 minutes and centrifuged at 4000 rpm for 20 minutes at 4 °C. 80 µL of supernatant was transferred into 240 µL of water (HPLC-grade) and was mixed via a plate shaker for 10 minutes. Each bioanalysis plate was sealed and shaken for 10 minutes prior to LC-MS analysis.

References

1.) H. Geng, X. Chen, J. Gui, Y. Zhang, Z. Shen, P. Qian, J. Chen, S. Zhang, W. Wang, *Nat. Catal.* **2019**, *2* (12), 1071–1077.
2.) Sokoloff, P.; Maillos, P.; Cuisiat, F.; Vidaluc, J.-L.; Imbert, T. Novel Benzodioxane-Piperidine Derivatives and Therapeutic Uses Thereof for the Treatment of Neuropsychiatric Disorders. EP2941427B1, November 9, 2016. https://patents.google.com/patent/EP2941427B1/en (accessed 2023-07-05).
3.) Patil, P.; Ahmadian-Moghaddam, M.; Domling, A., *Green Chem.*, **2020**, 22, 6902.

NMR Spectra

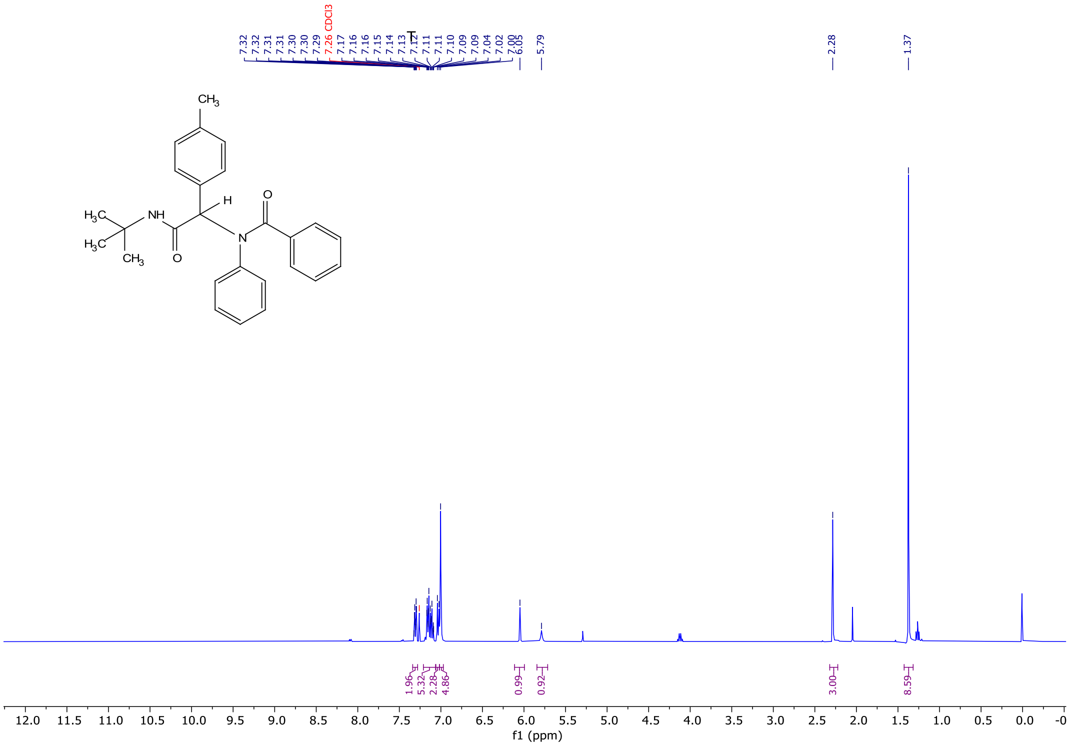

Figure S1. ^{1}H NMR (400 MHz, $CDCl_3$) of **1a**.

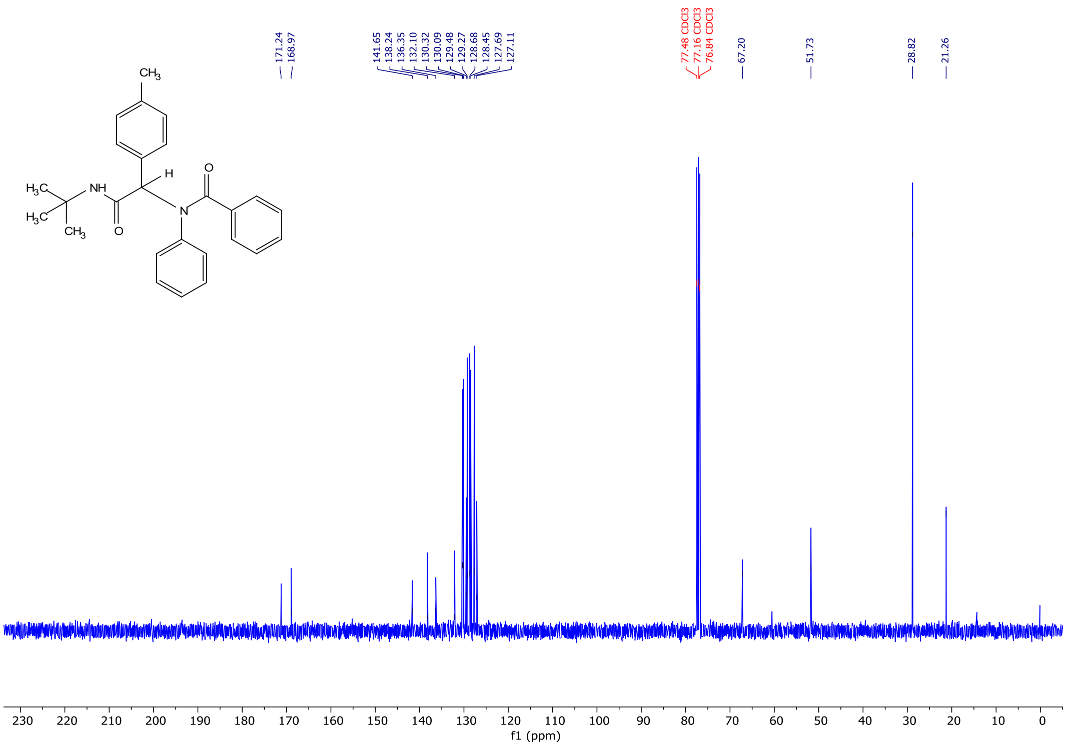

Figure S2. ^{13}C NMR (101 MHz, $CDCl_3$) of **1a**.

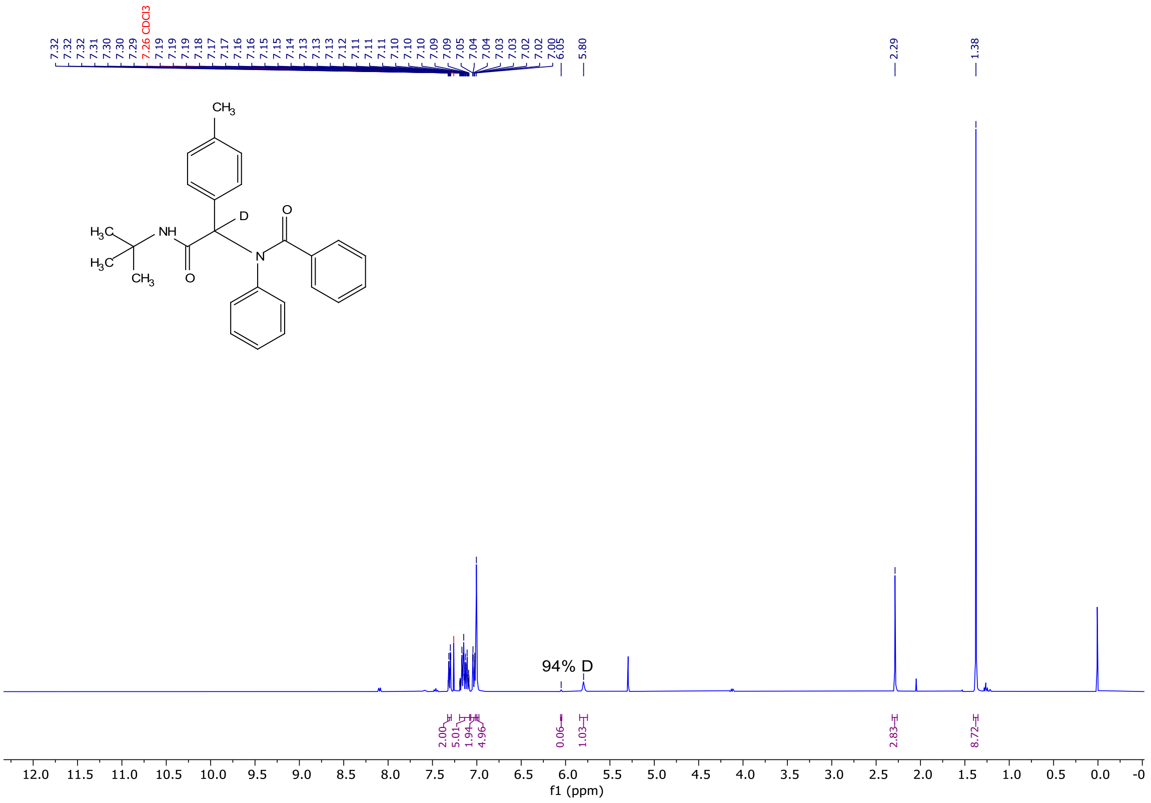

Figure S3. ^{1}H NMR (400 MHz, $CDCl_3$) of **1b**.

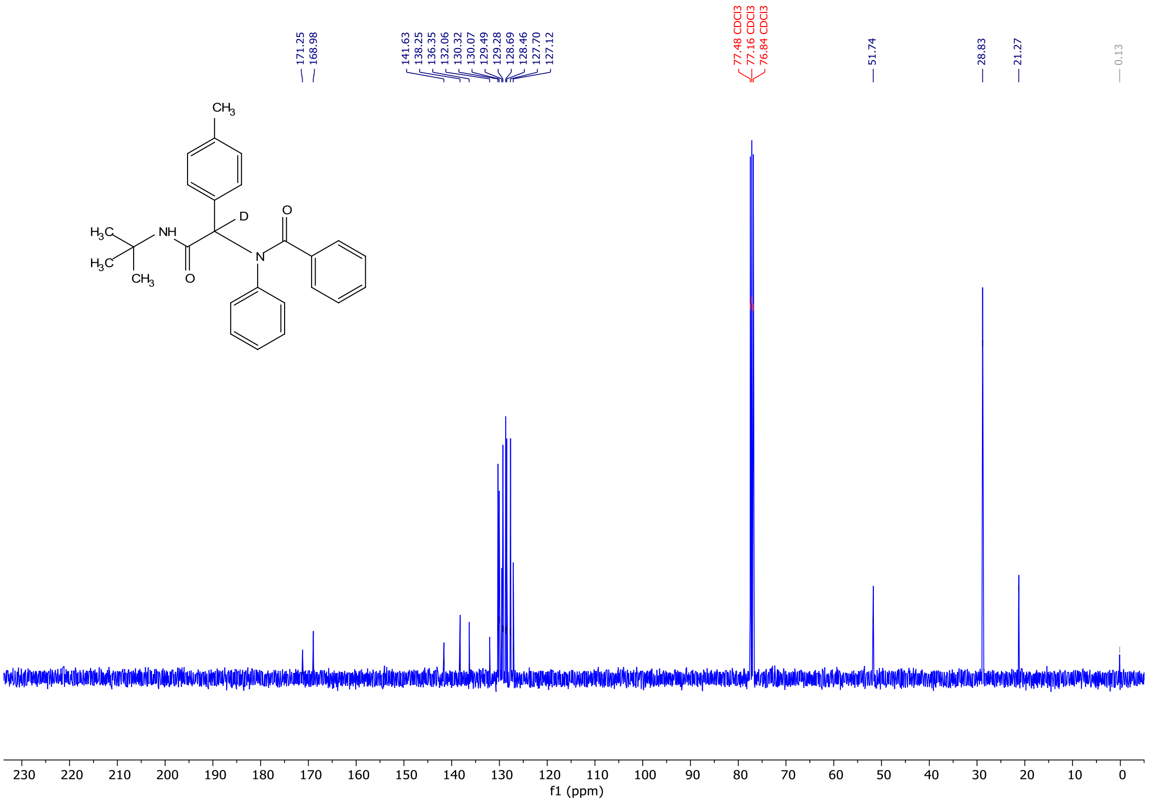

Figure S4. ^{13}C NMR (101 MHz, $CDCl_3$) of **1b**

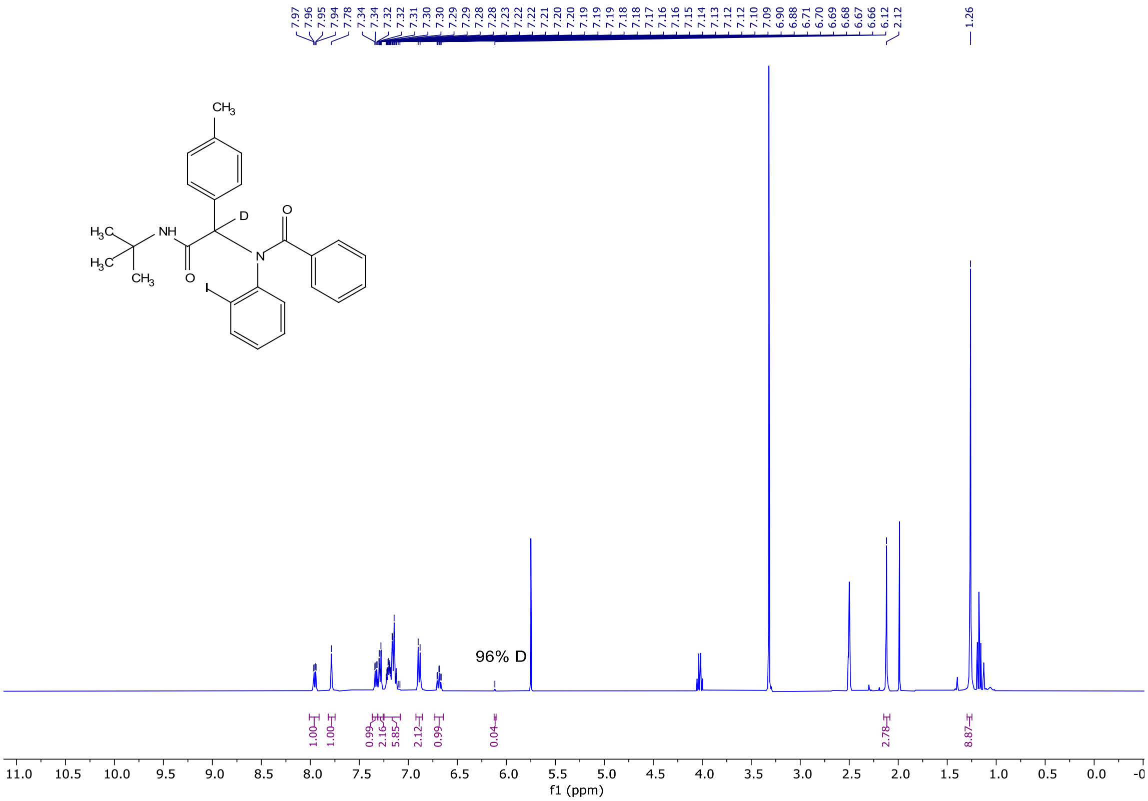

Figure S5. ^{1}H NMR (400 MHz, DMSO) of **1c**.

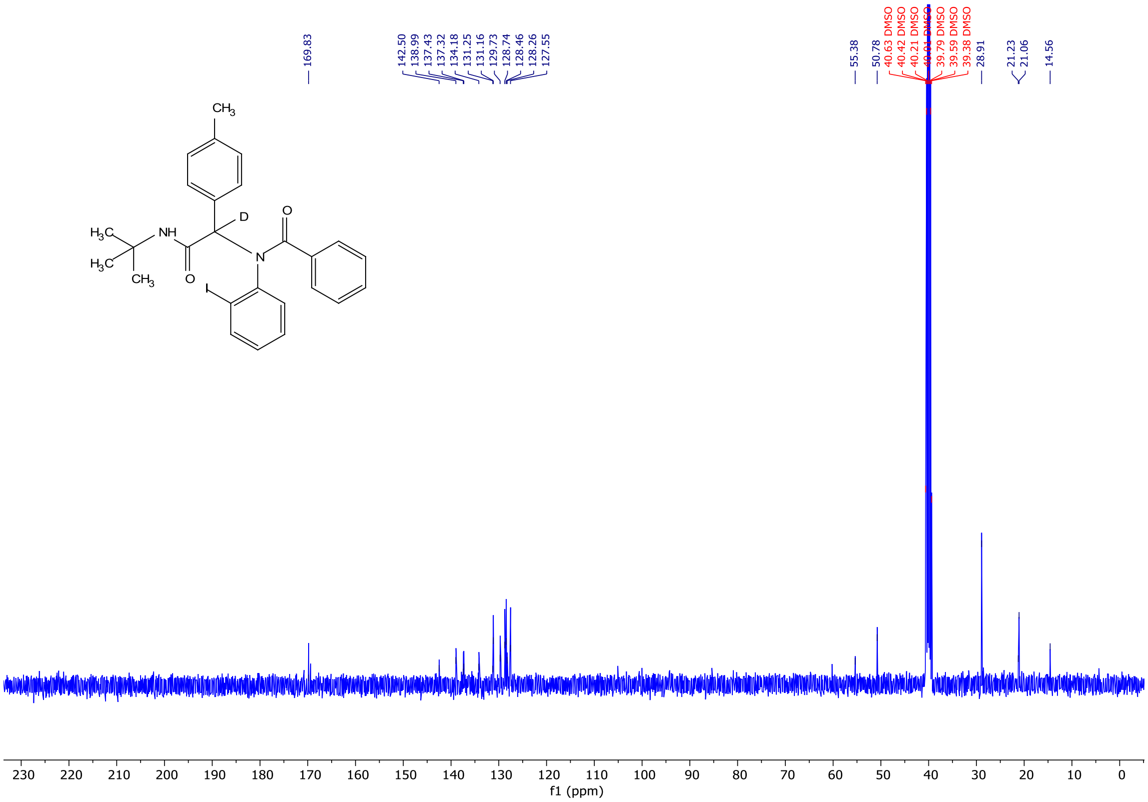

Figure S6. ^{13}C NMR (101 MHz, $CDCl_3$) of **1c.**

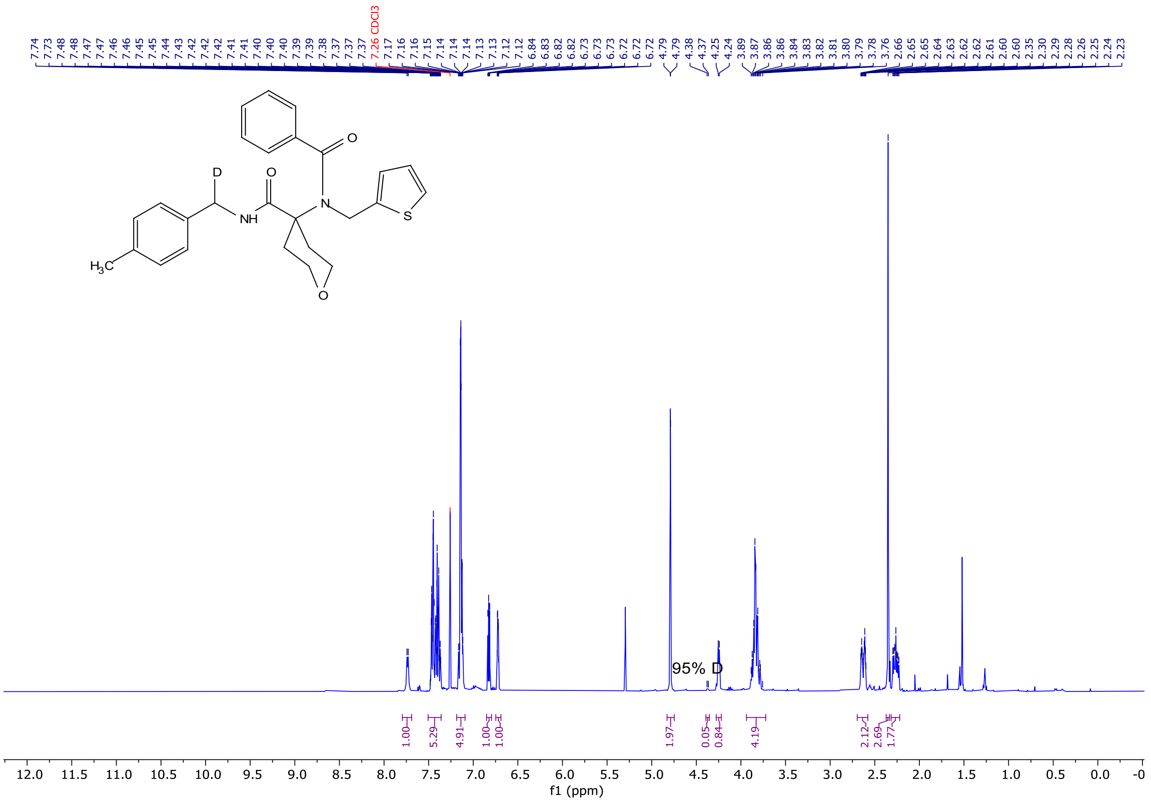

Figure S7. ^{1}H NMR (400 MHz, $CDCl_3$) of **1e**.

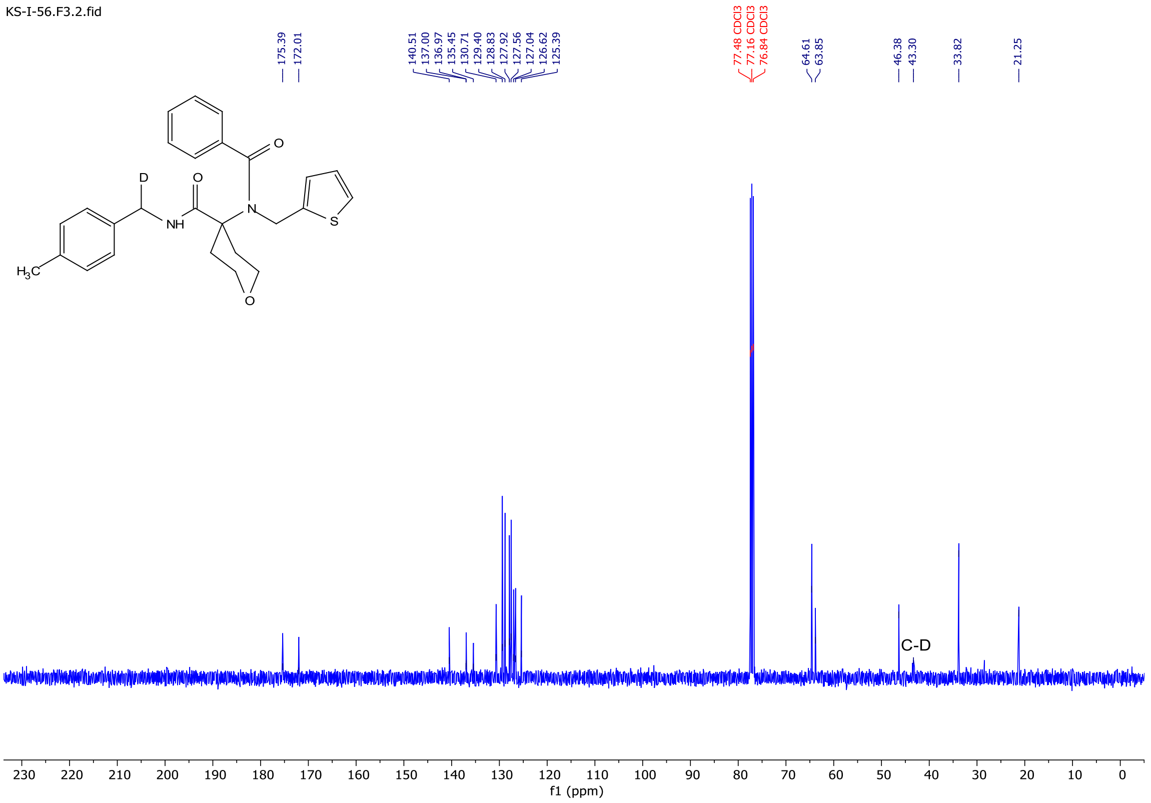

Figure S8. ^{13}C NMR (101 MHz, $CDCl_3$) of **1e**.

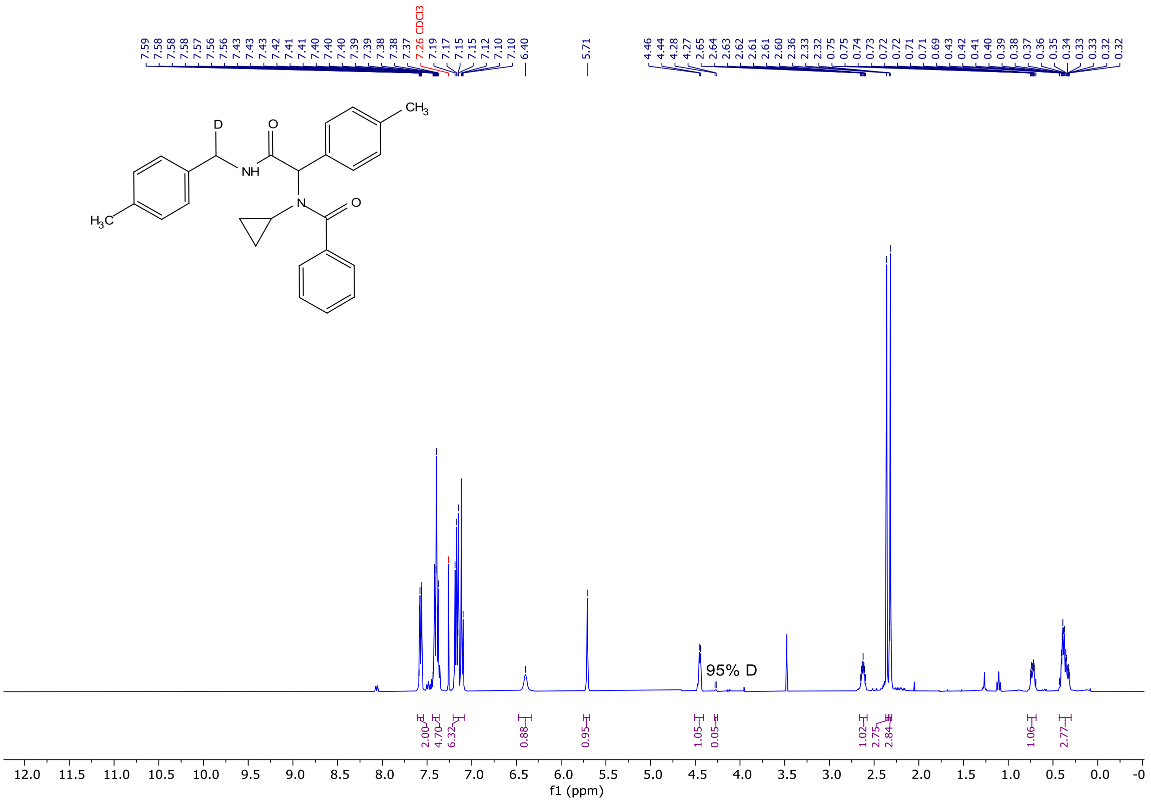

Figure S9. ^{1}H NMR (400 MHz, $CDCl_3$) of **1f**.

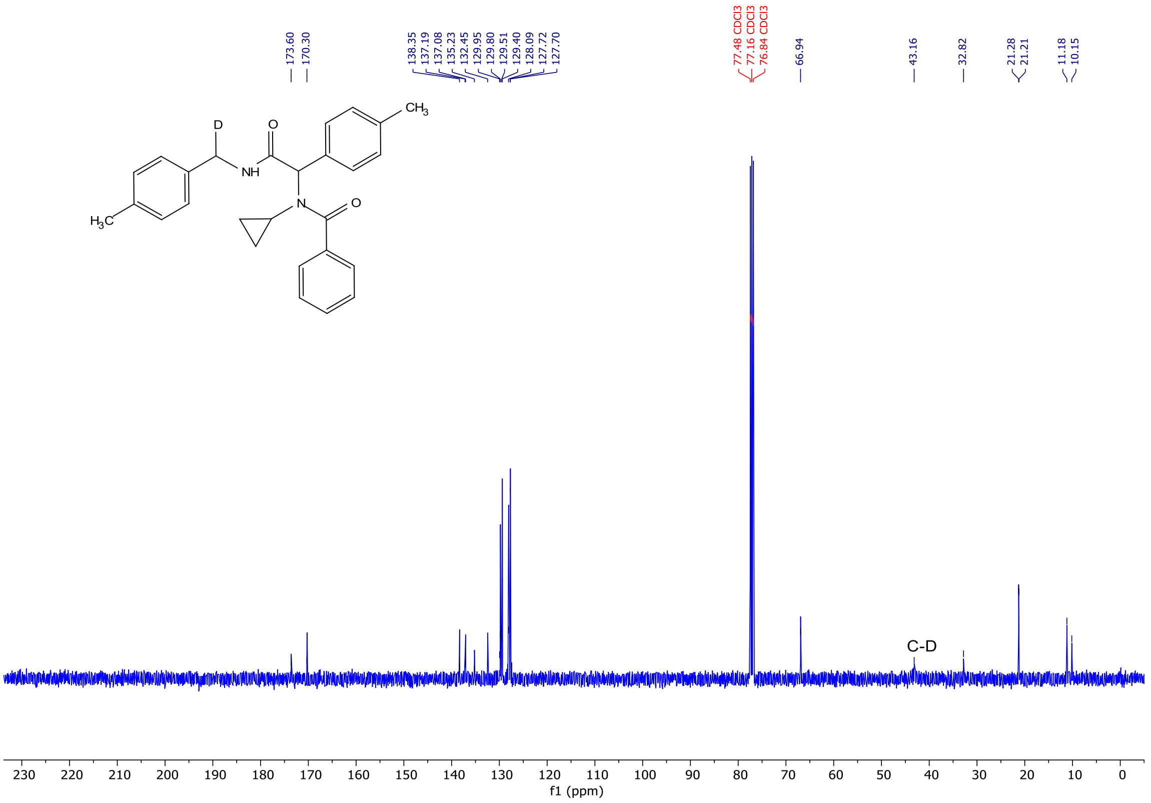

Figure S10. ^{13}C NMR (101 MHz, $CDCl_3$) of **1f**.

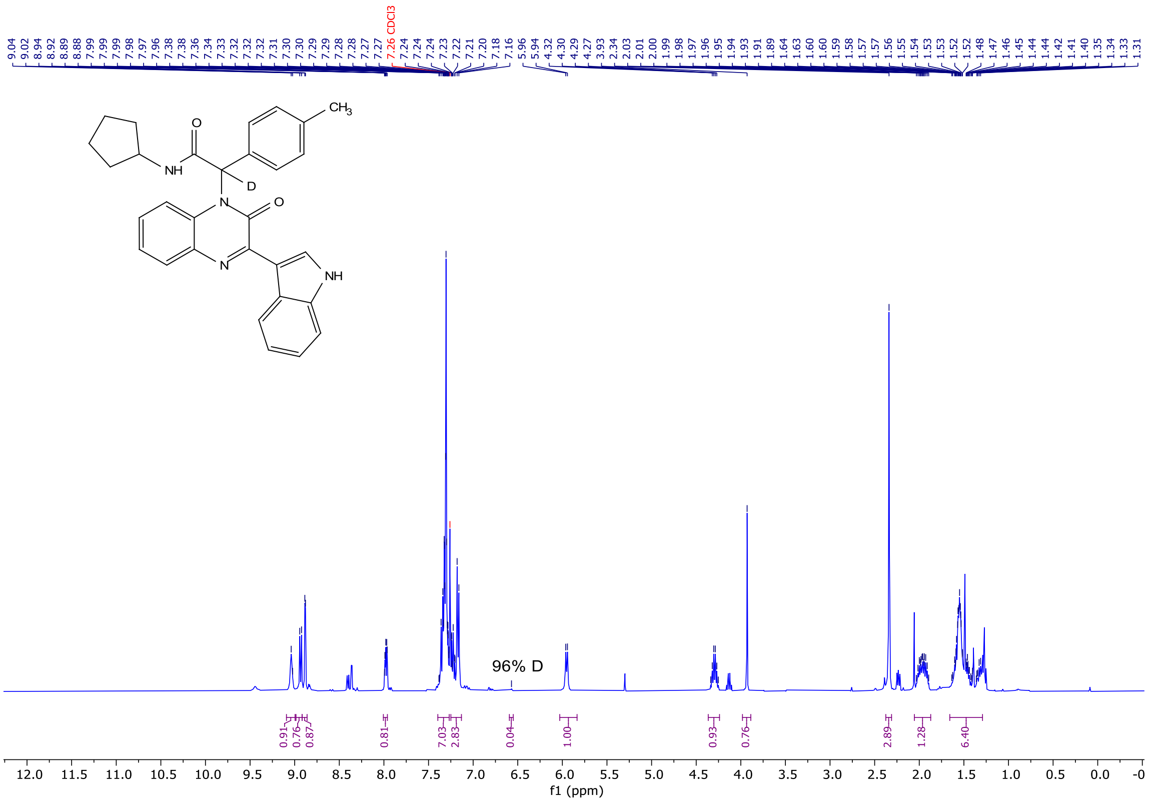

Figure S11. ^{1}H NMR (400 MHz, $CDCl_3$) of **1g**.

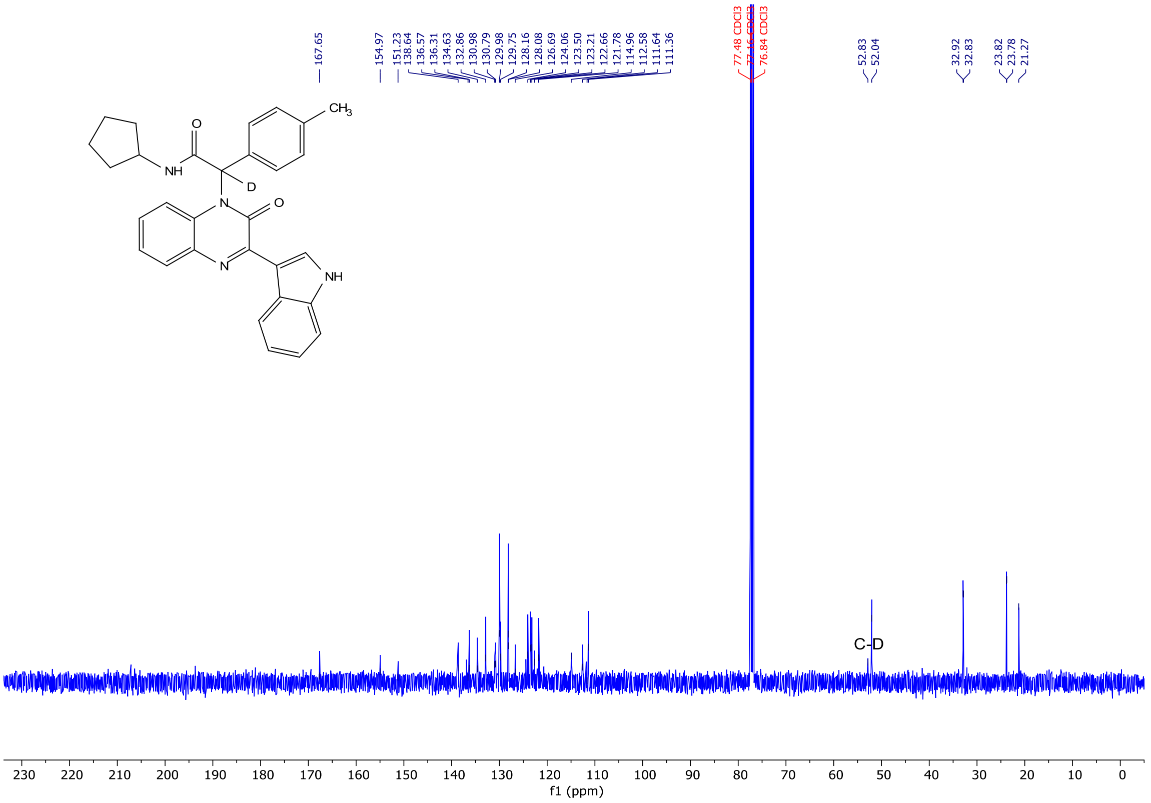

Figure S12. ^{13}C NMR (101 MHz, $CDCl_3$) of **1g**.

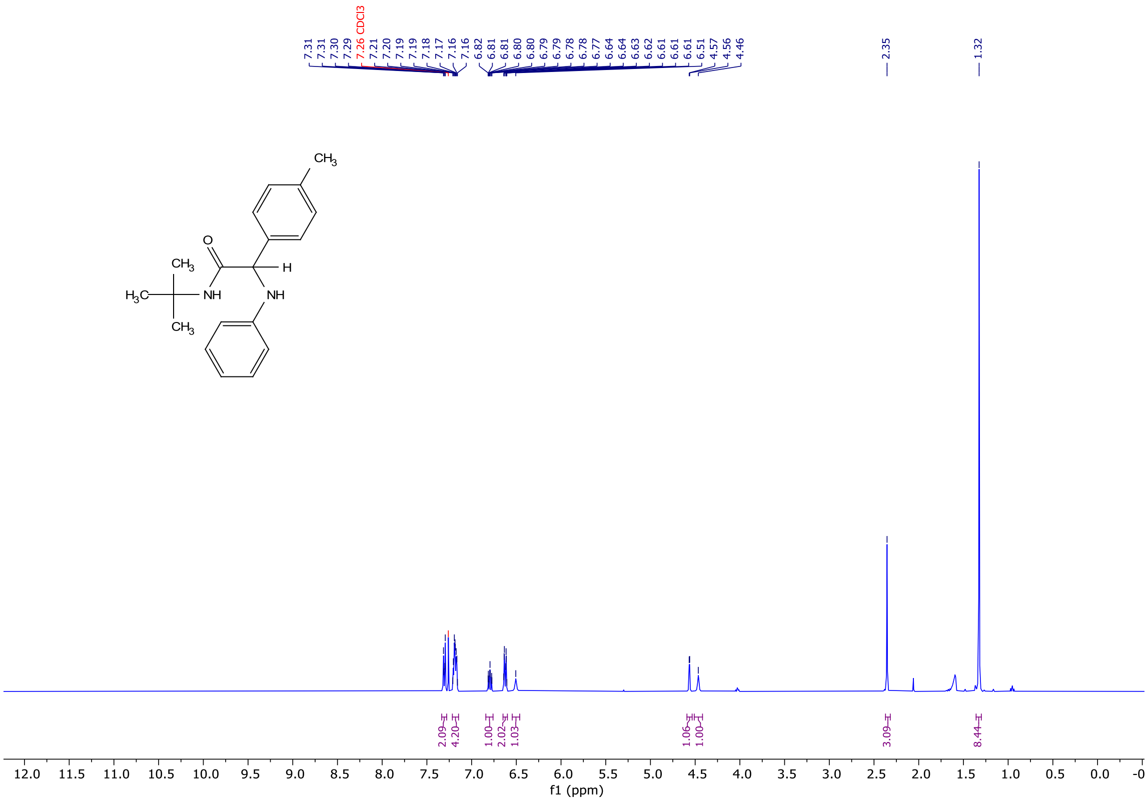

Figure S13. ^{1}H NMR (400 MHz, $CDCl_3$) of **2a**.

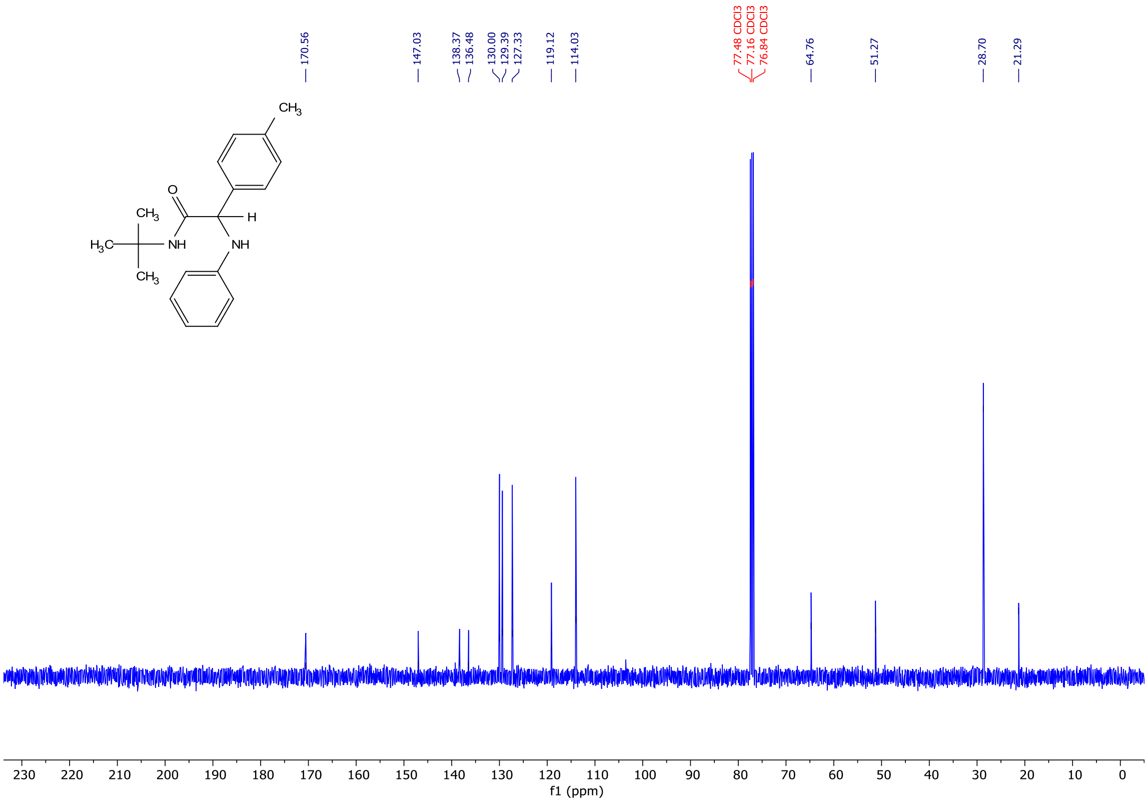

Figure S14. ^{13}C NMR (101 MHz, $CDCl_3$) of **2a**.

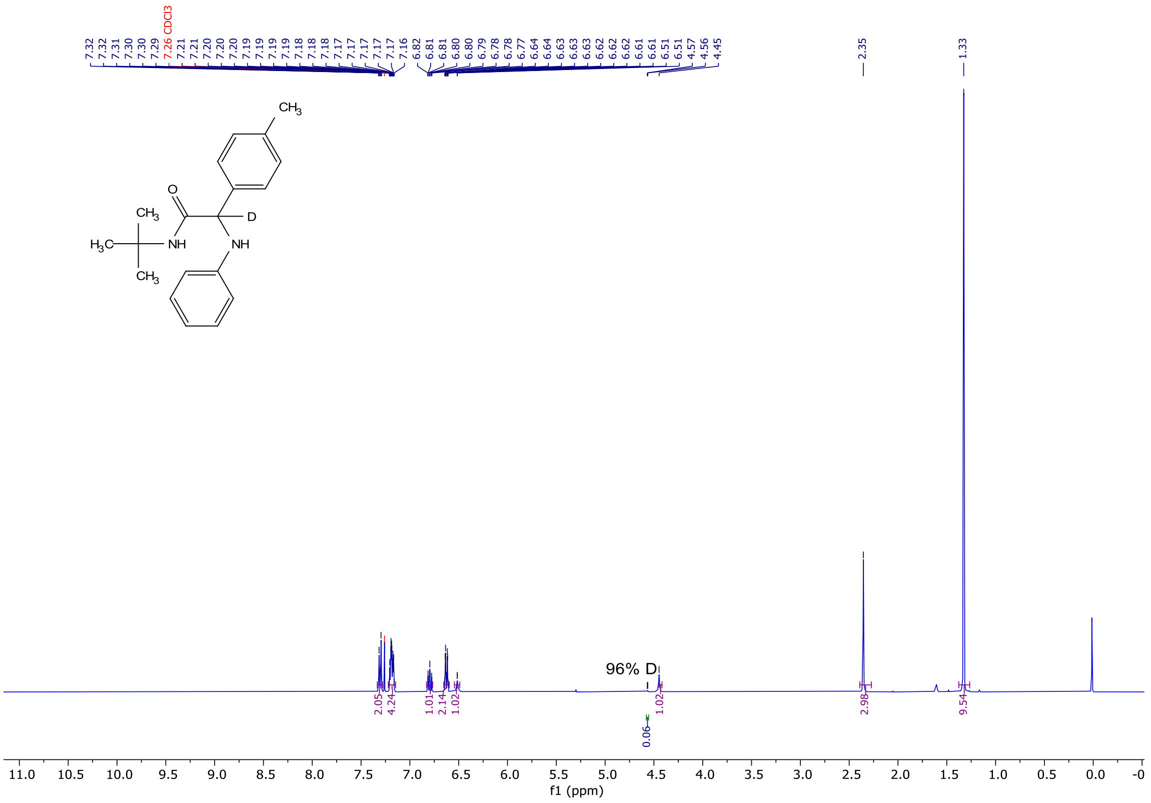

Figure S15. ^{1}H NMR (400 MHz, $CDCl_3$) of **2b**.

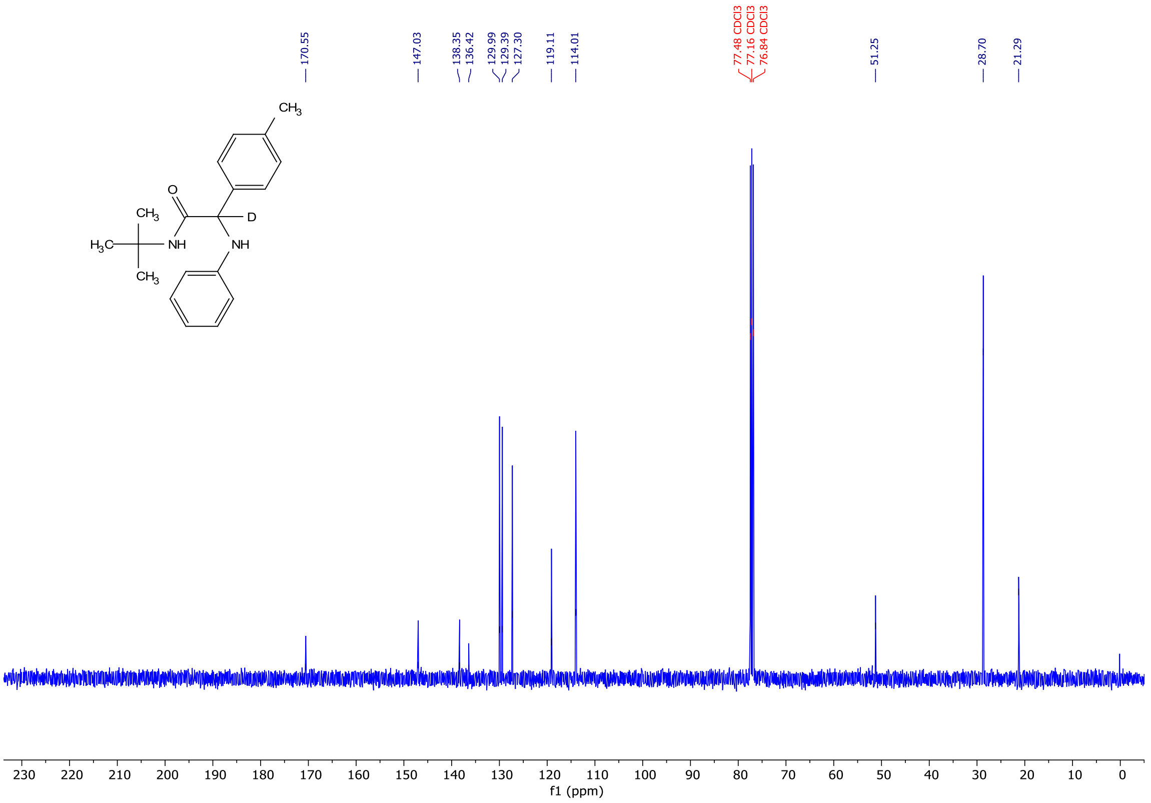

Figure S16. ^{13}C NMR (101 MHz, $CDCl_3$) of **2b**.

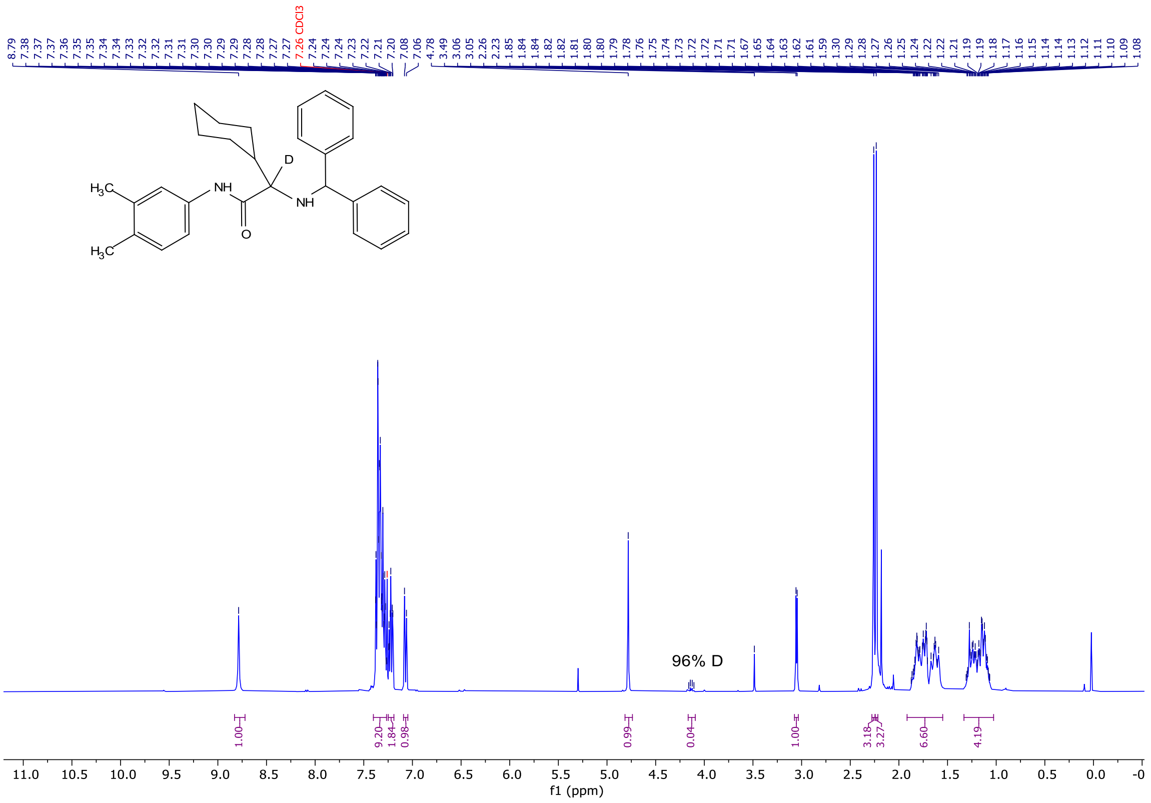

Figure S17. ^{1}H NMR (400 MHz, $CDCl_3$) of **2c**.

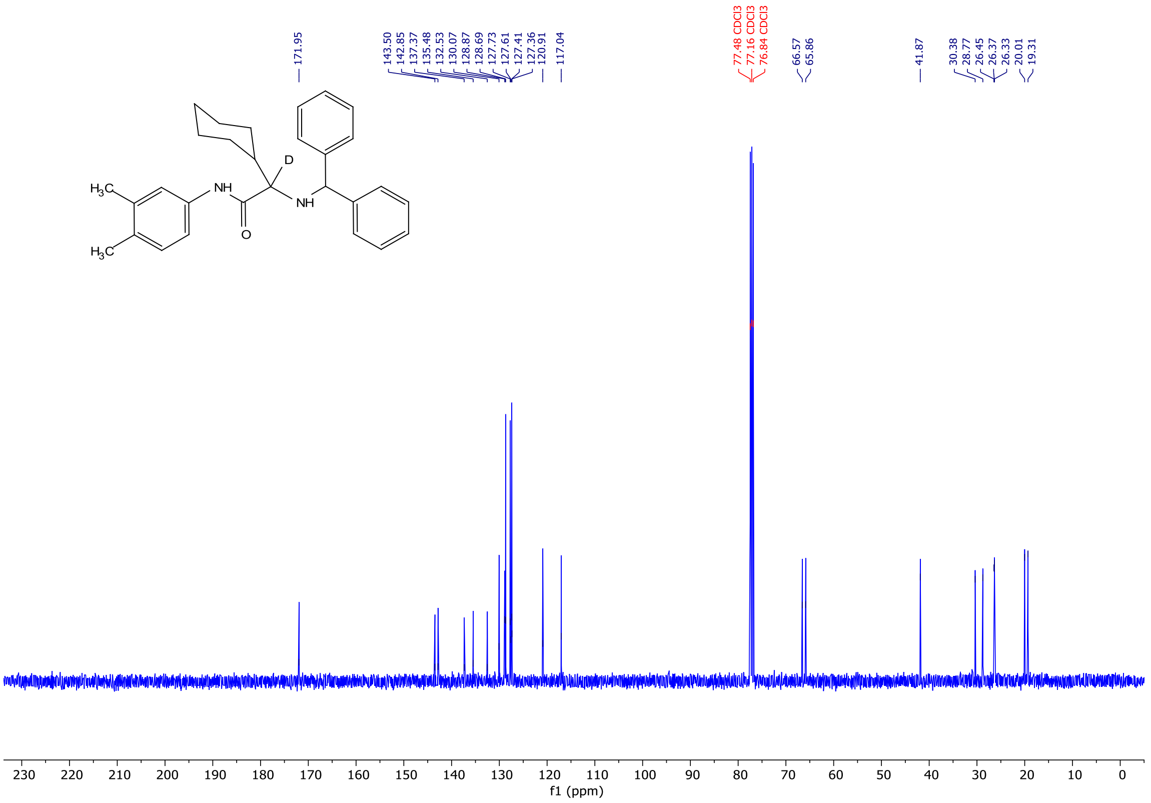

Figure S18. ^{13}C NMR (101 MHz, $CDCl_3$) of **2c**.

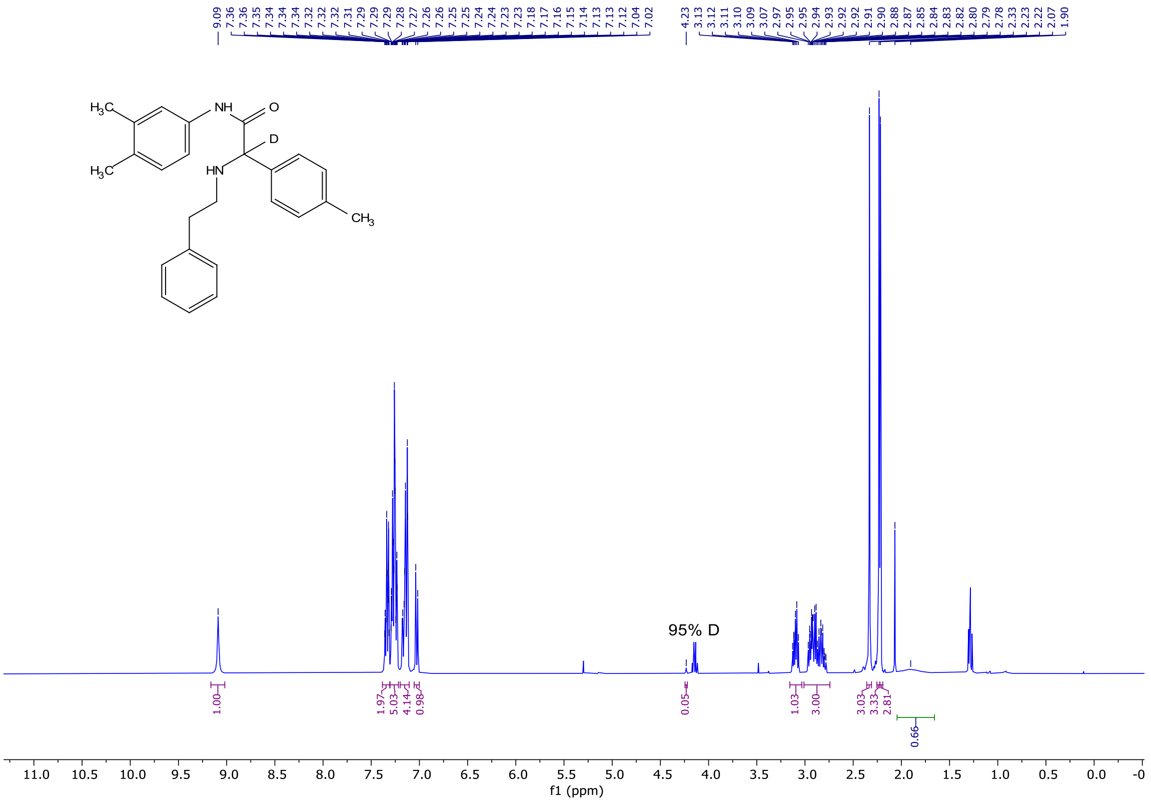

Figure S19. ^{1}H NMR (400 MHz, $CDCl_3$) of **2d**.

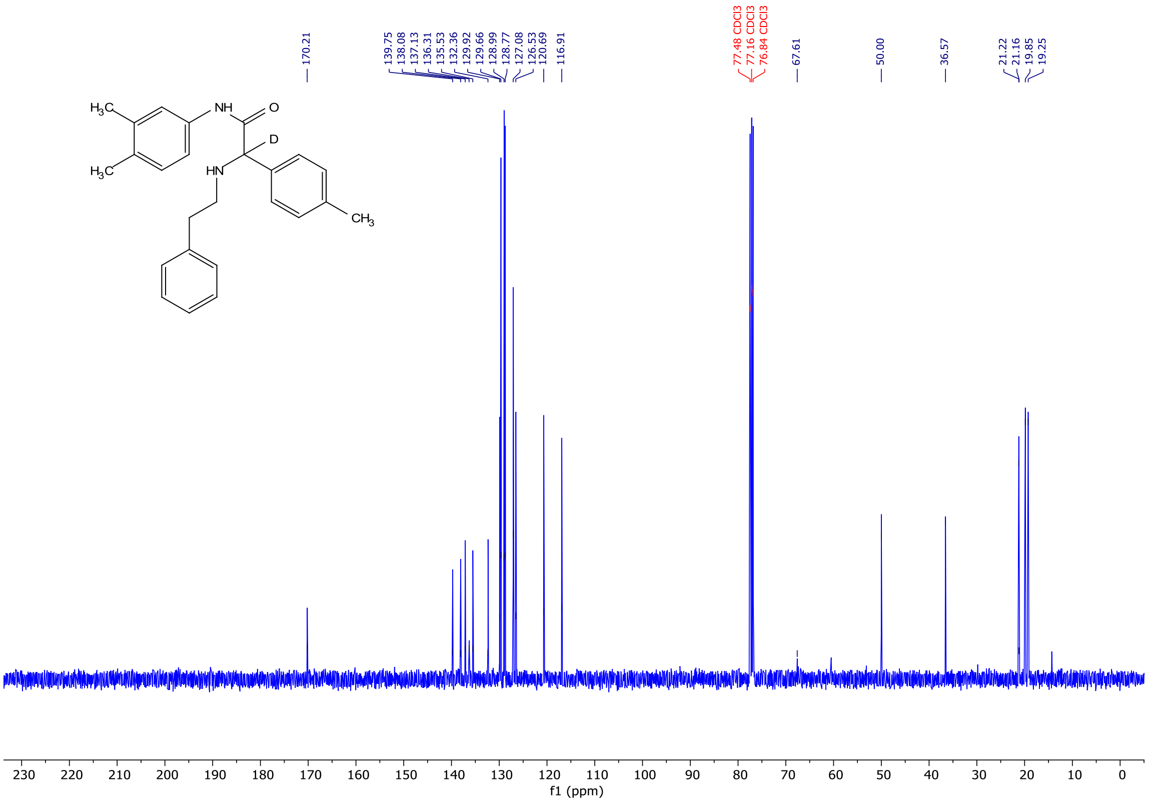

Figure S20. ^{13}C NMR (101 MHz, $CDCl_3$) of **2d**.

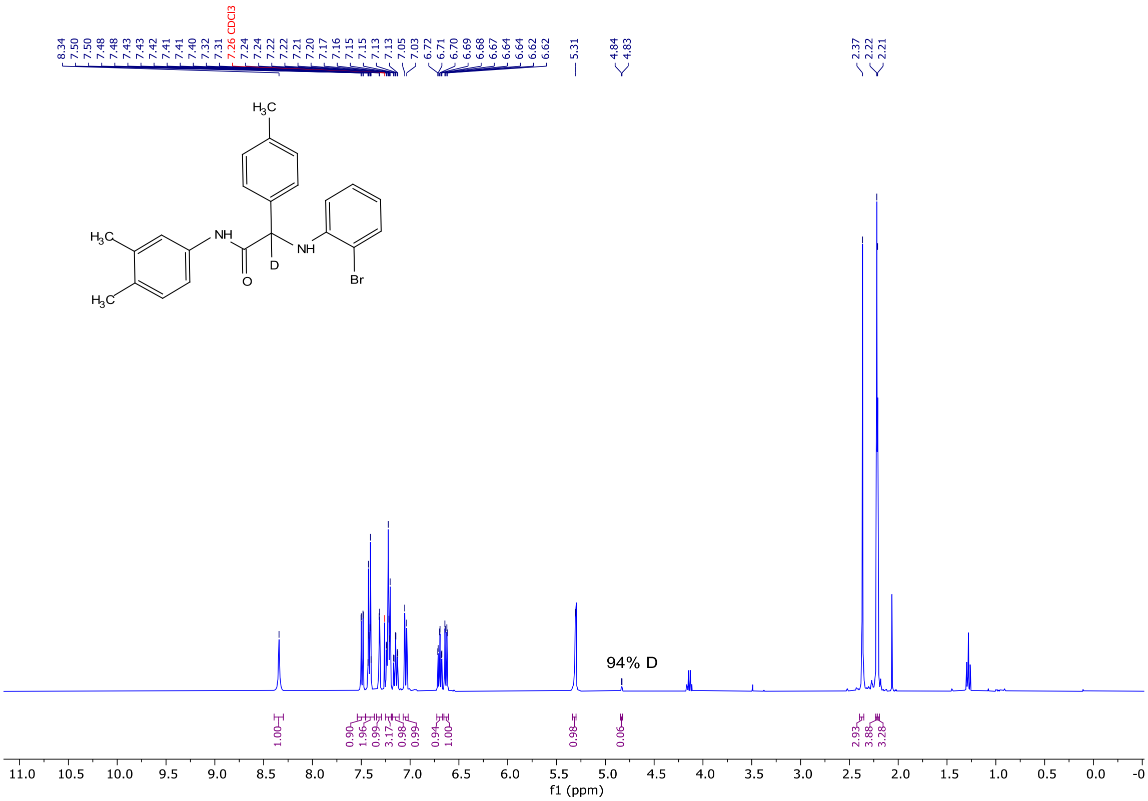

Figure S21. ^{1}H NMR (400 MHz, $CDCl_3$) of **2e**.

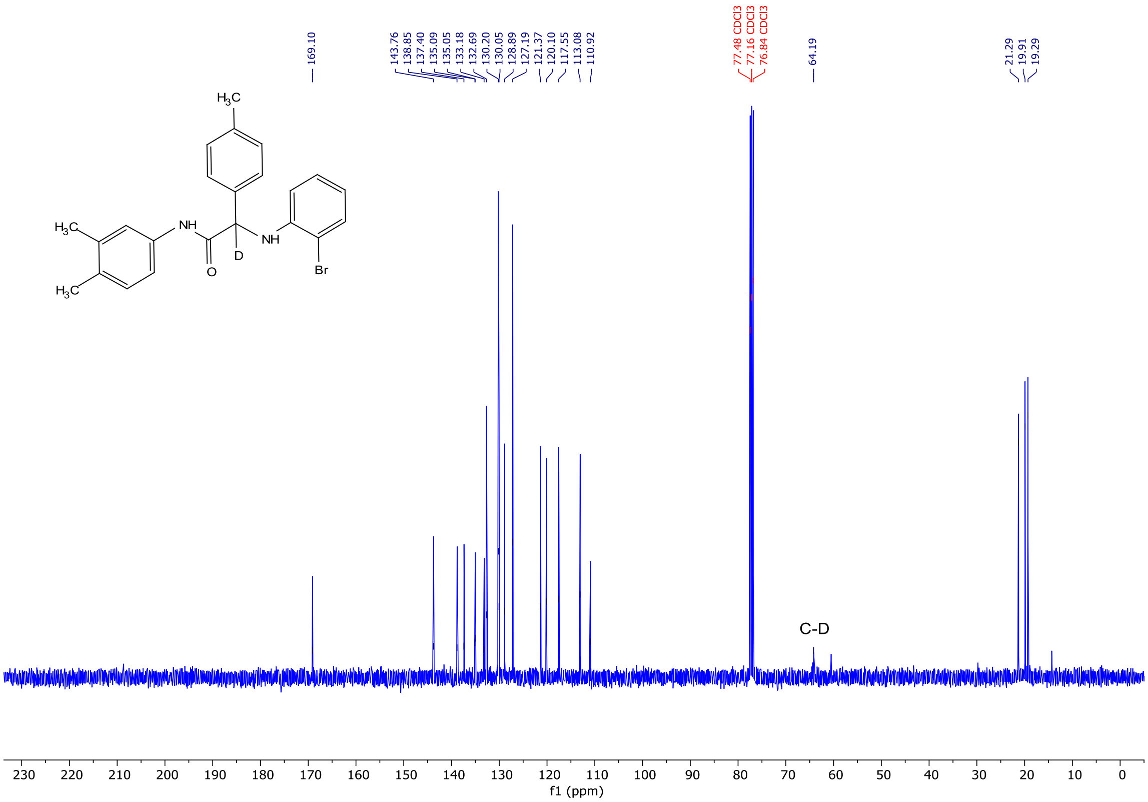

Figure S22. ^{13}C NMR (101 MHz, $CDCl_3$) of **2e**.

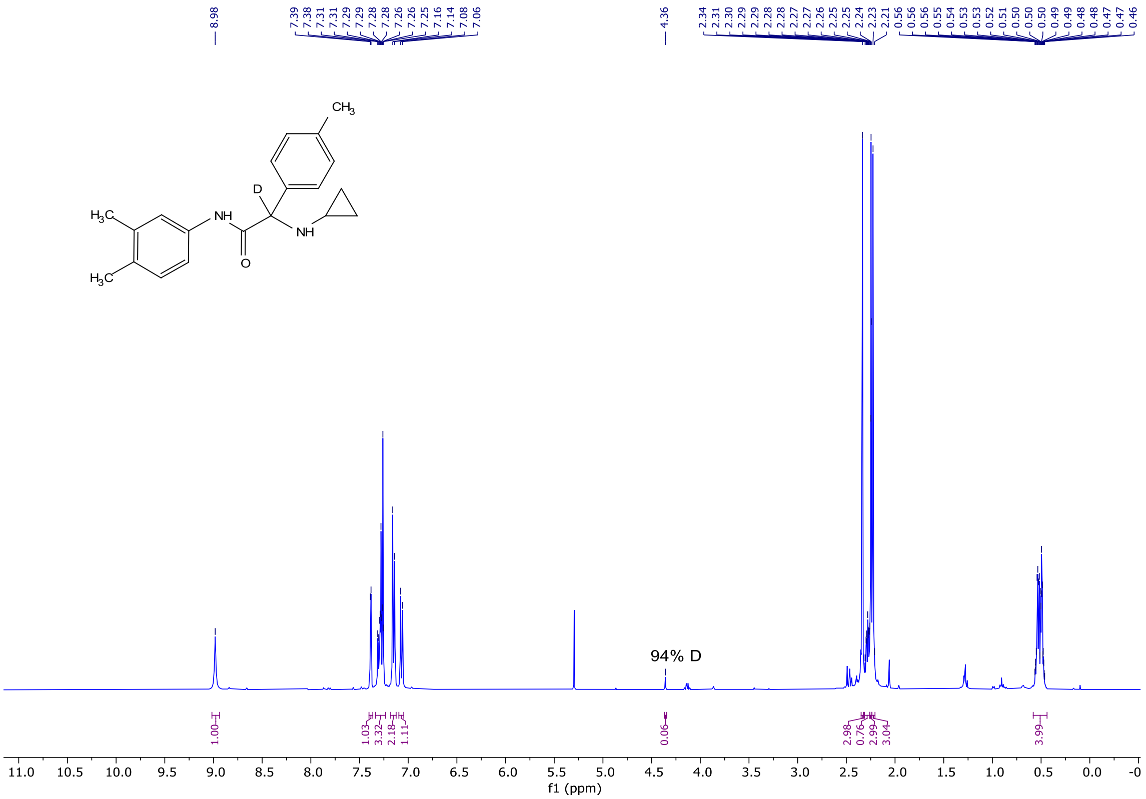

Figure S23. ^{1}H NMR (400 MHz, $CDCl_3$) of **2f**.

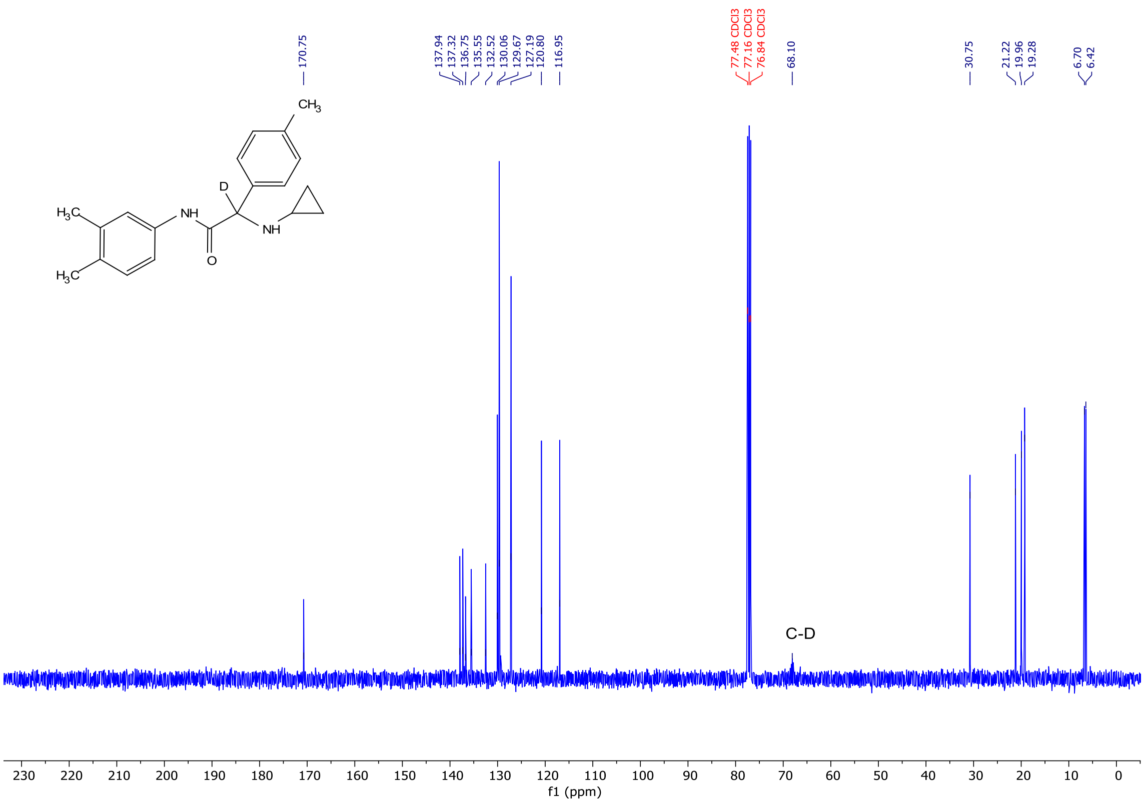

Figure S24. ^{13}C NMR (101 MHz, $CDCl_3$) of **2f**.

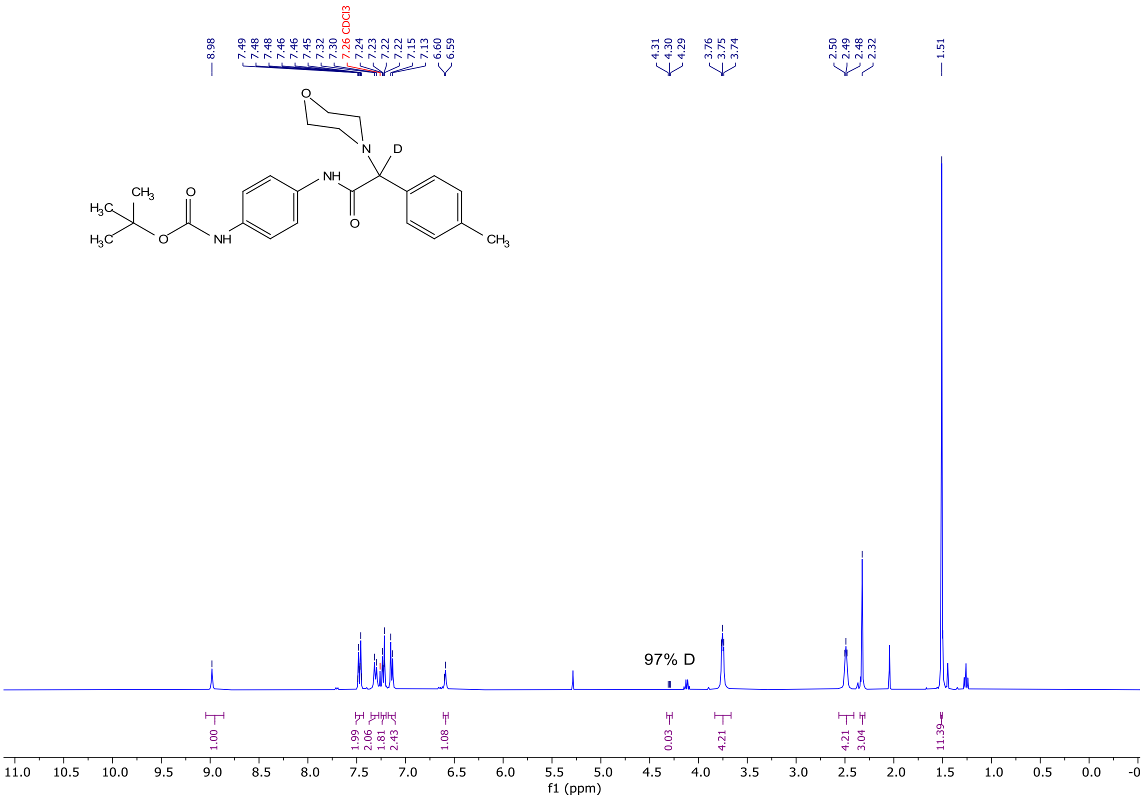

Figure S25. ^{1}H NMR (400 MHz, $CDCl_3$) of **2g**.

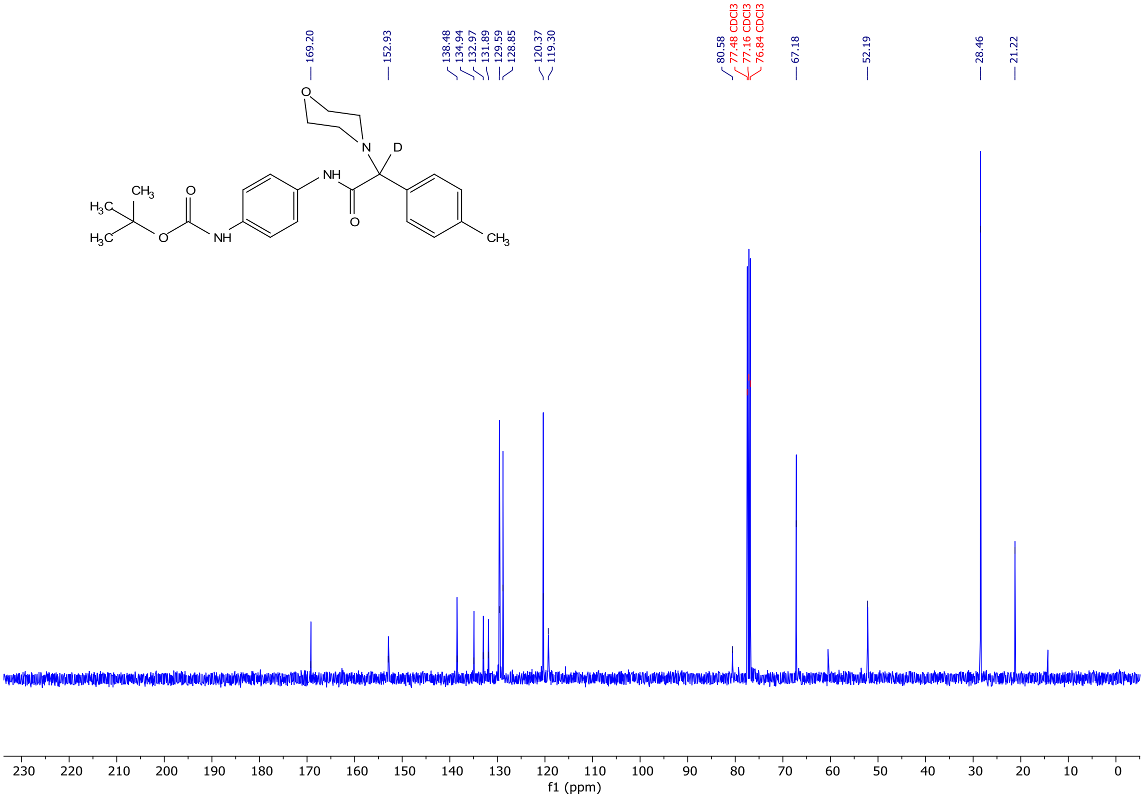

Figure S26. ^{13}C NMR (101 MHz, $CDCl_3$) of **2g**.

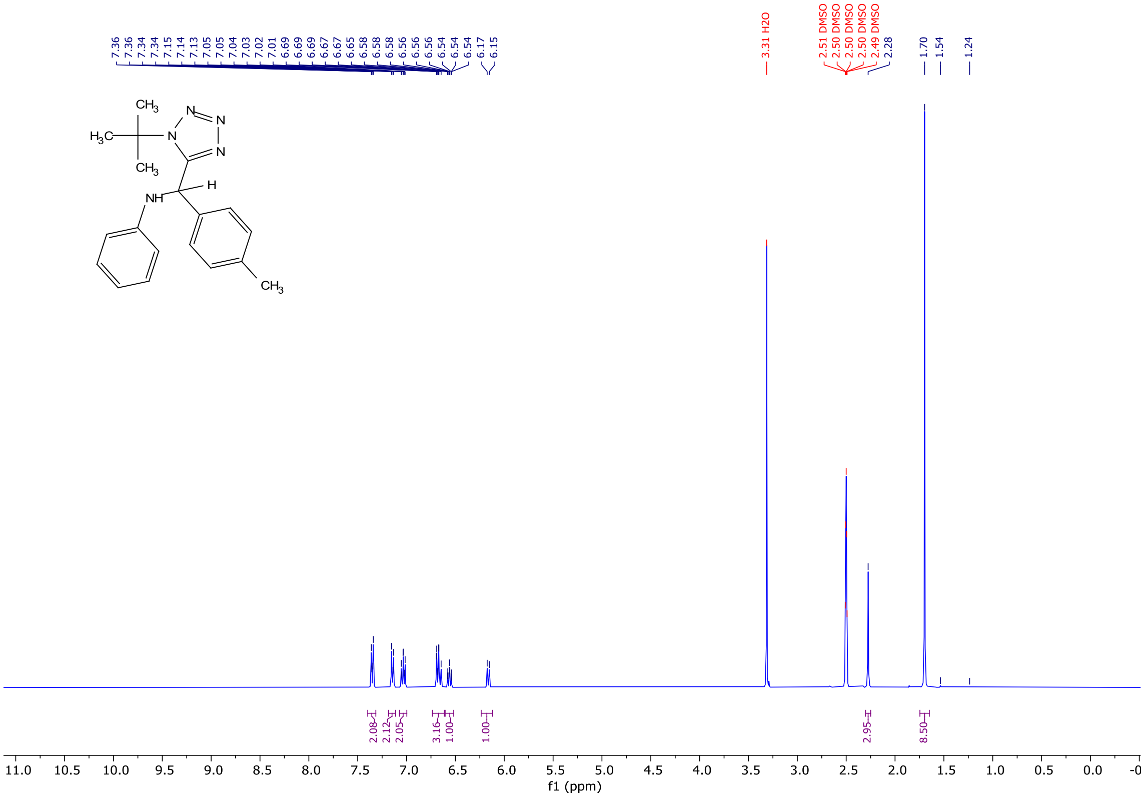

Figure S27. ^{1}H NMR (400 MHz, $CDCl_3$) of **3a**.

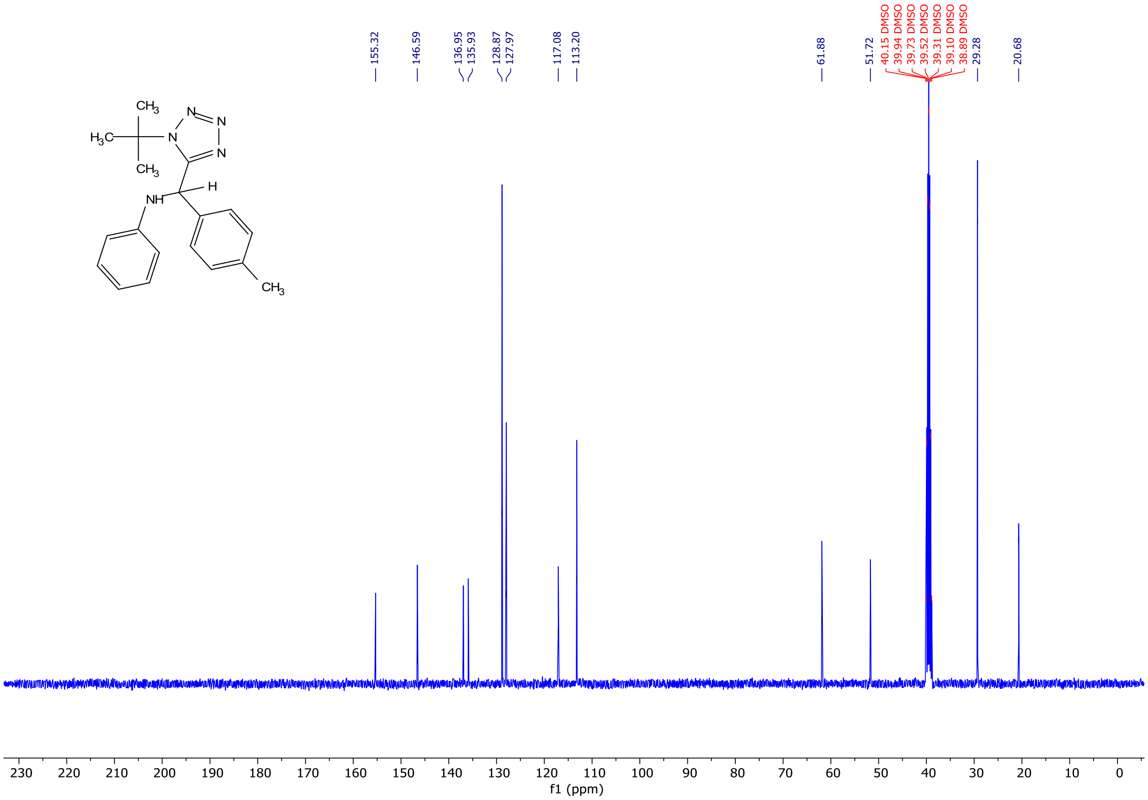

Figure S28. ^{13}C NMR (101 MHz, $CDCl_3$) of **3a**.

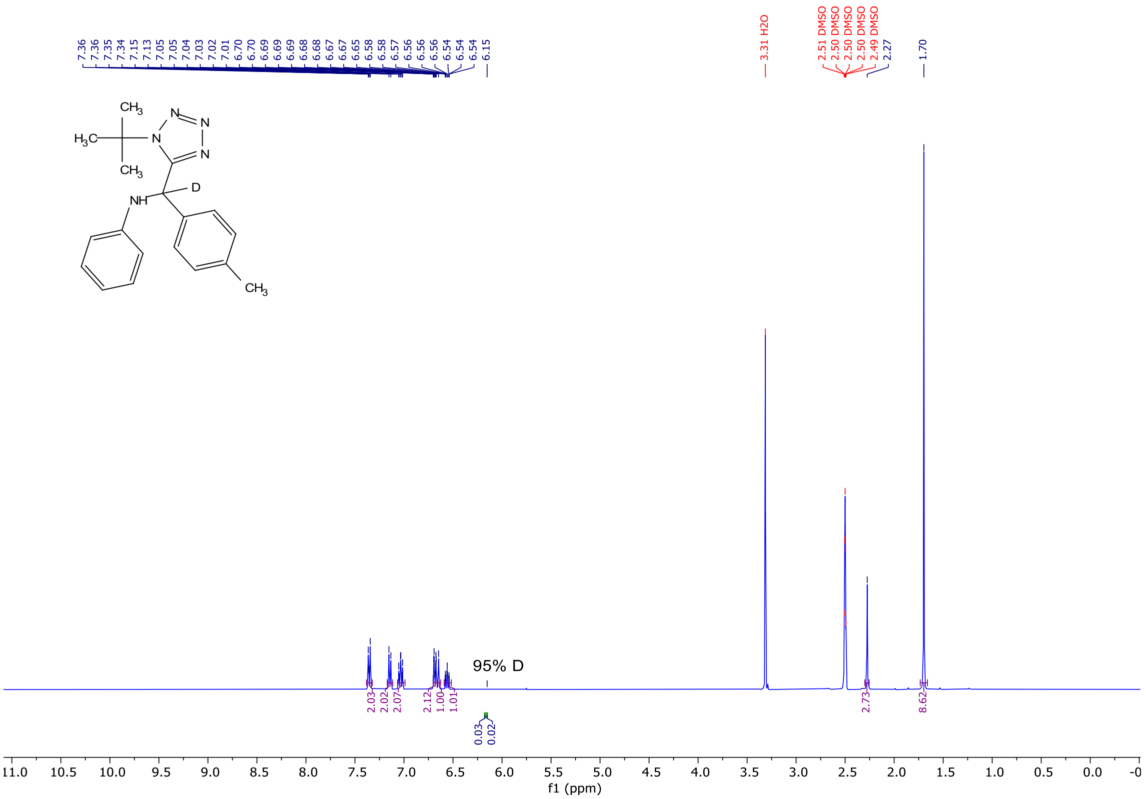

Figure S29. ^{1}H NMR (400 MHz, $CDCl_3$) of **3b**.

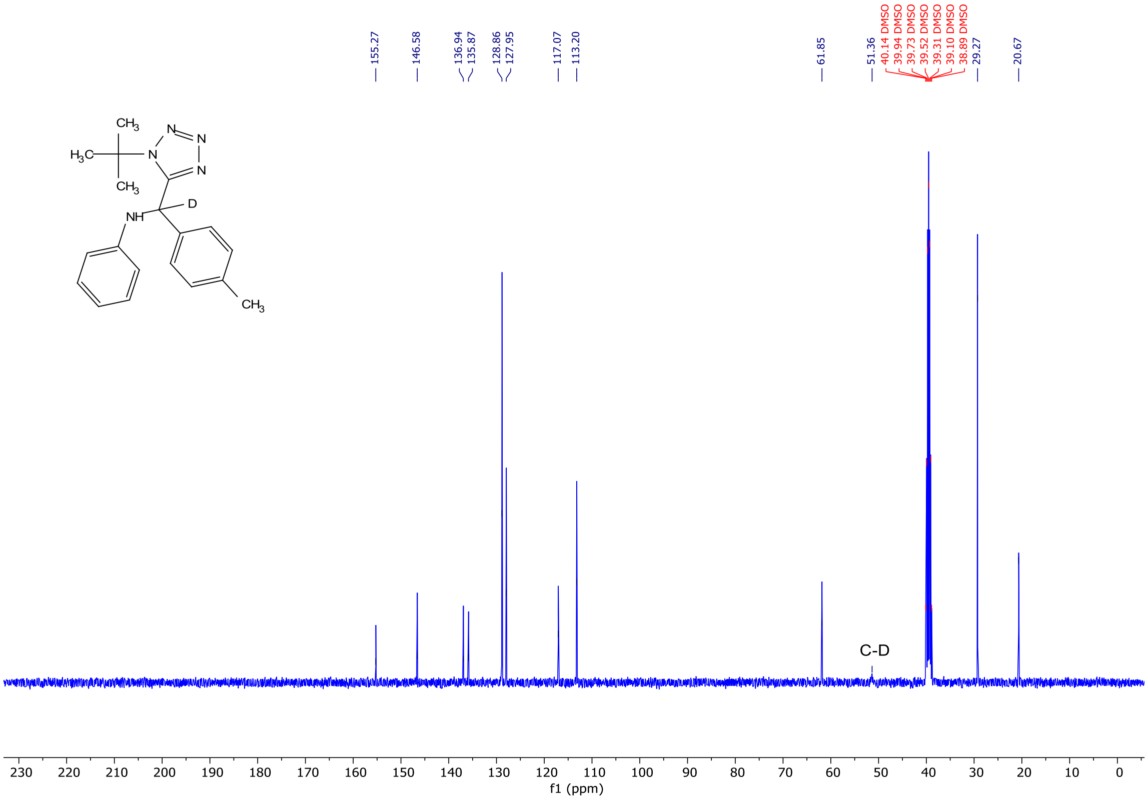

Figure S30. ^{13}C NMR (101 MHz, $CDCl_3$) of **3b**.

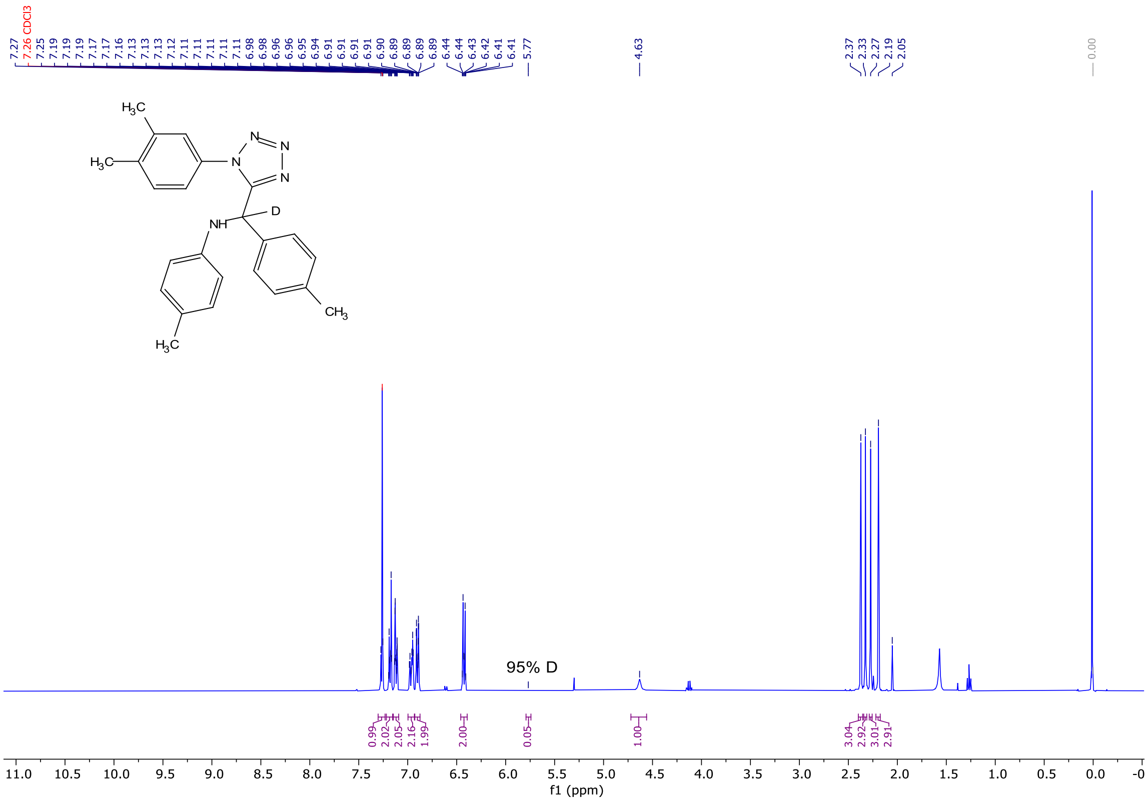

Figure S31. ^{1}H NMR (400 MHz, $CDCl_3$) of **3c**.

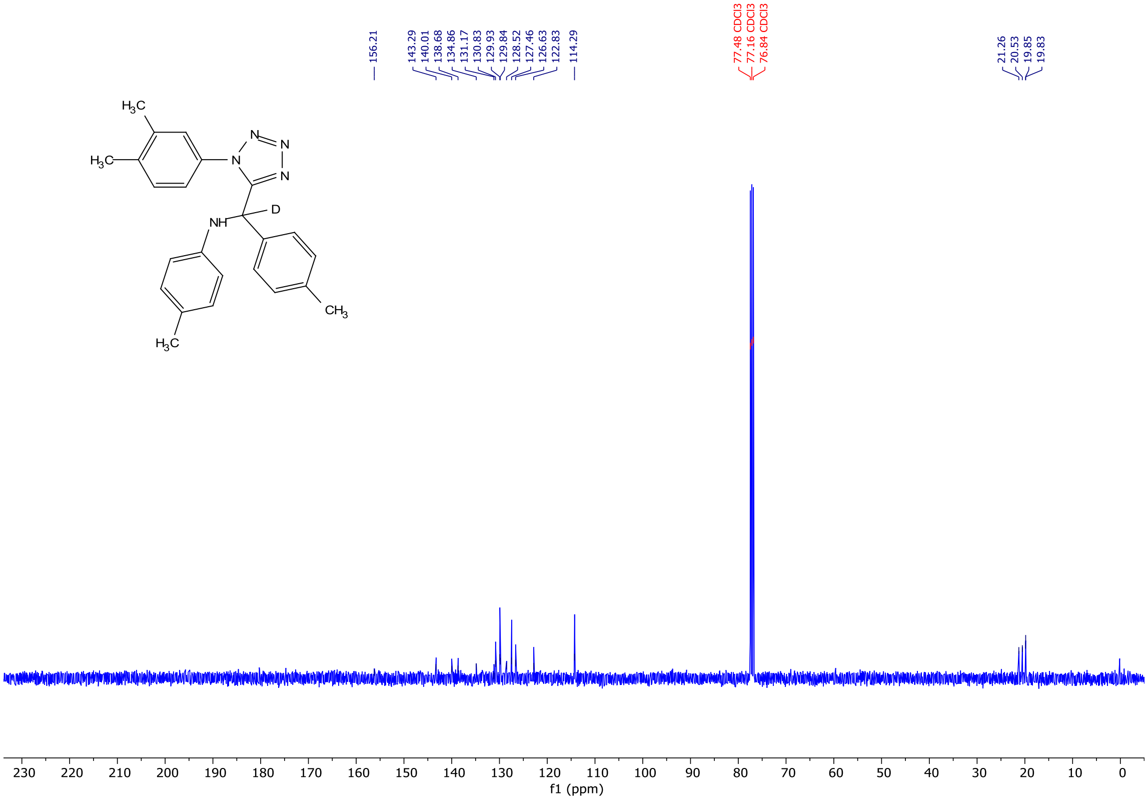

Figure S32. ^{13}C NMR (101 MHz, $CDCl_3$) of **3c**.

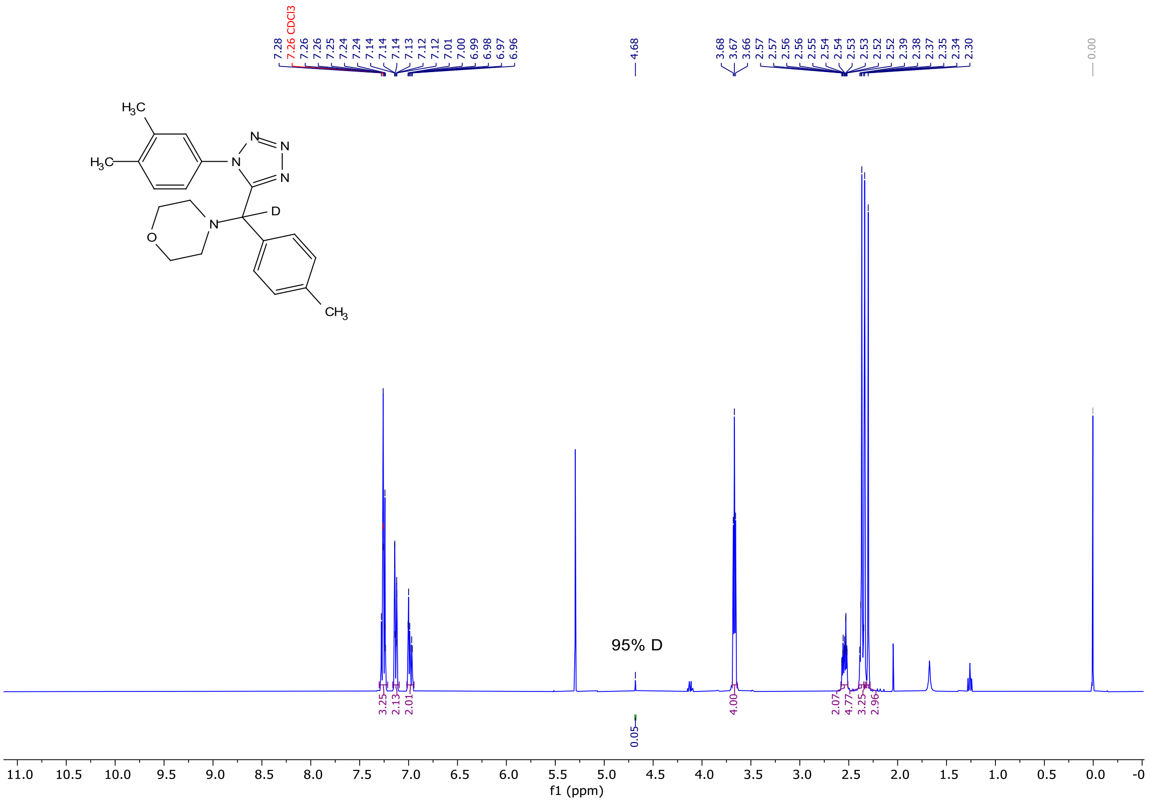

Figure S33. ^{1}H NMR (400 MHz, $CDCl_3$) of **3d**.

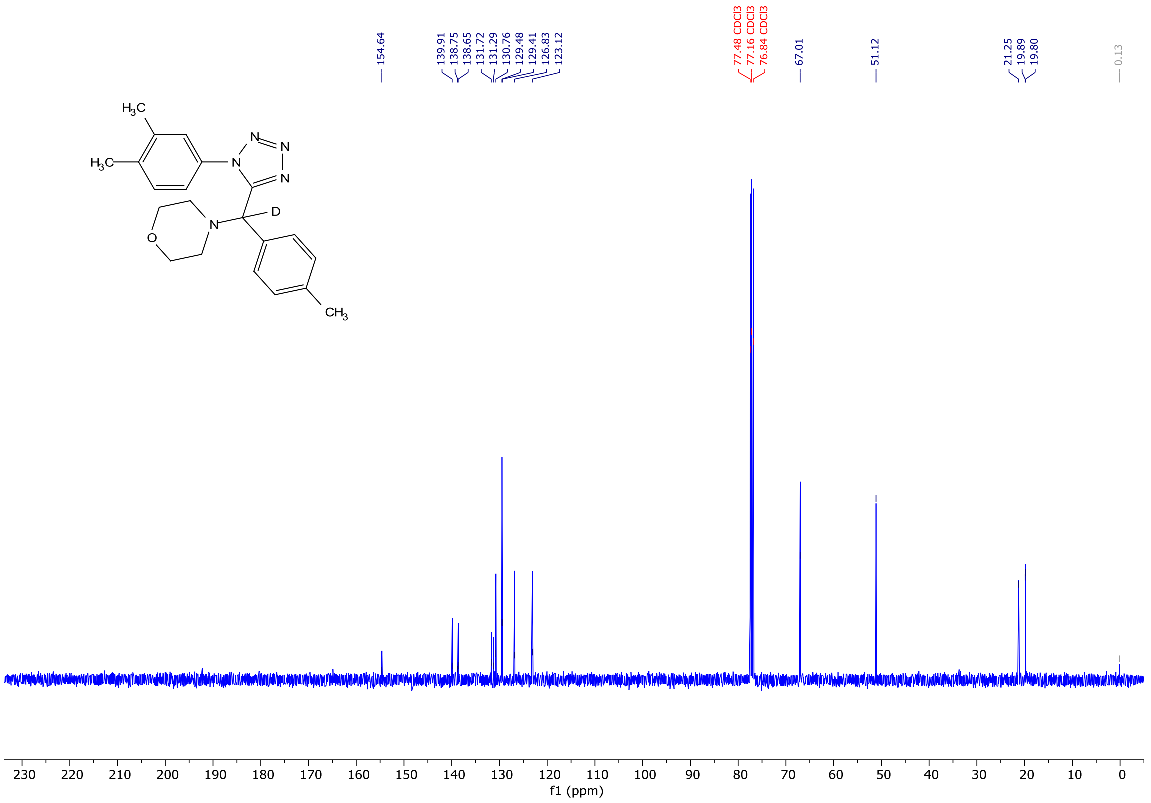

Figure S34. ^{13}C NMR (101 MHz, $CDCl_3$) of **3d**.

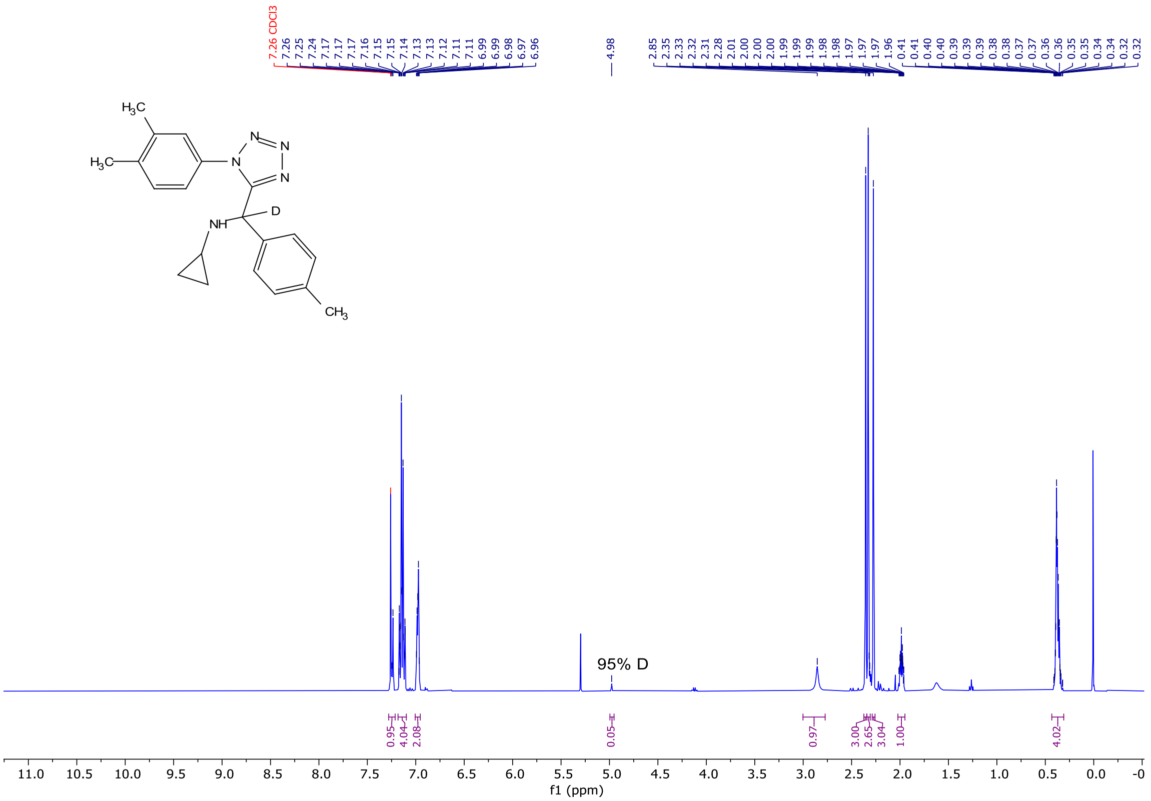

Figure S35. ^{1}H NMR (400 MHz, $CDCl_3$) of **3e**.

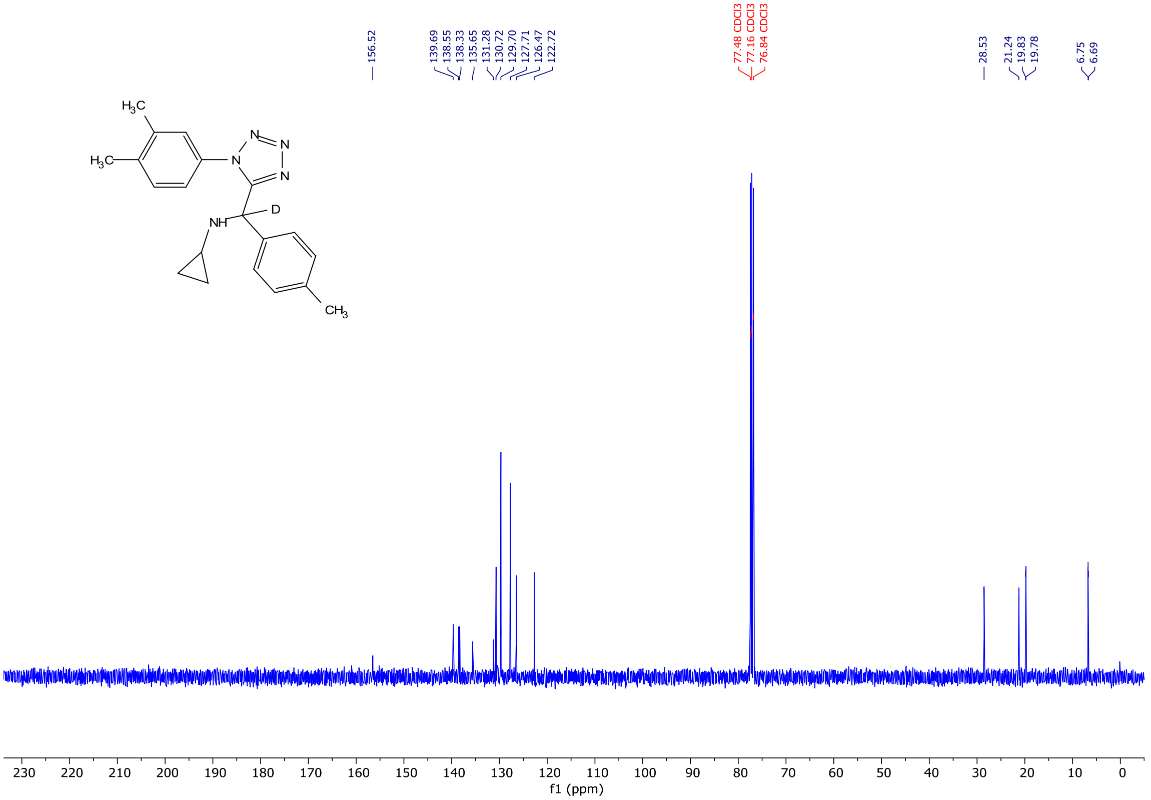

Figure S36. ^{13}C NMR (101 MHz, $CDCl_3$) of **3e**.

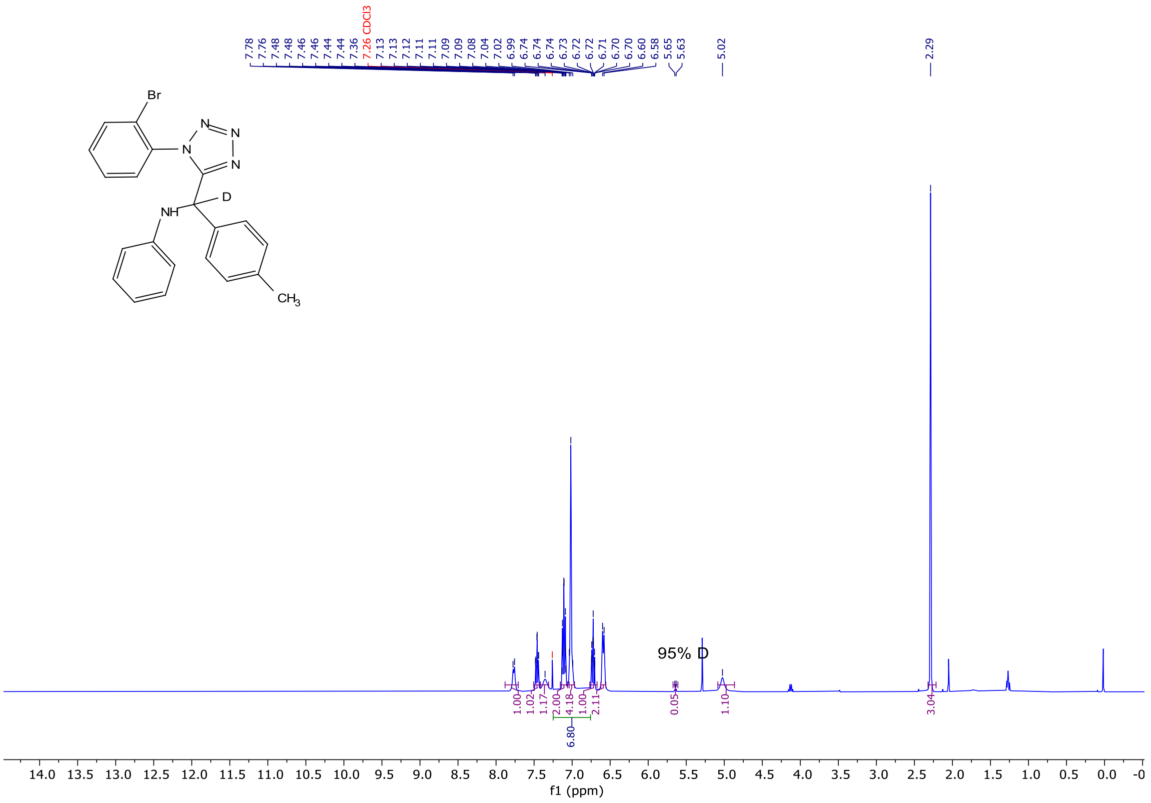

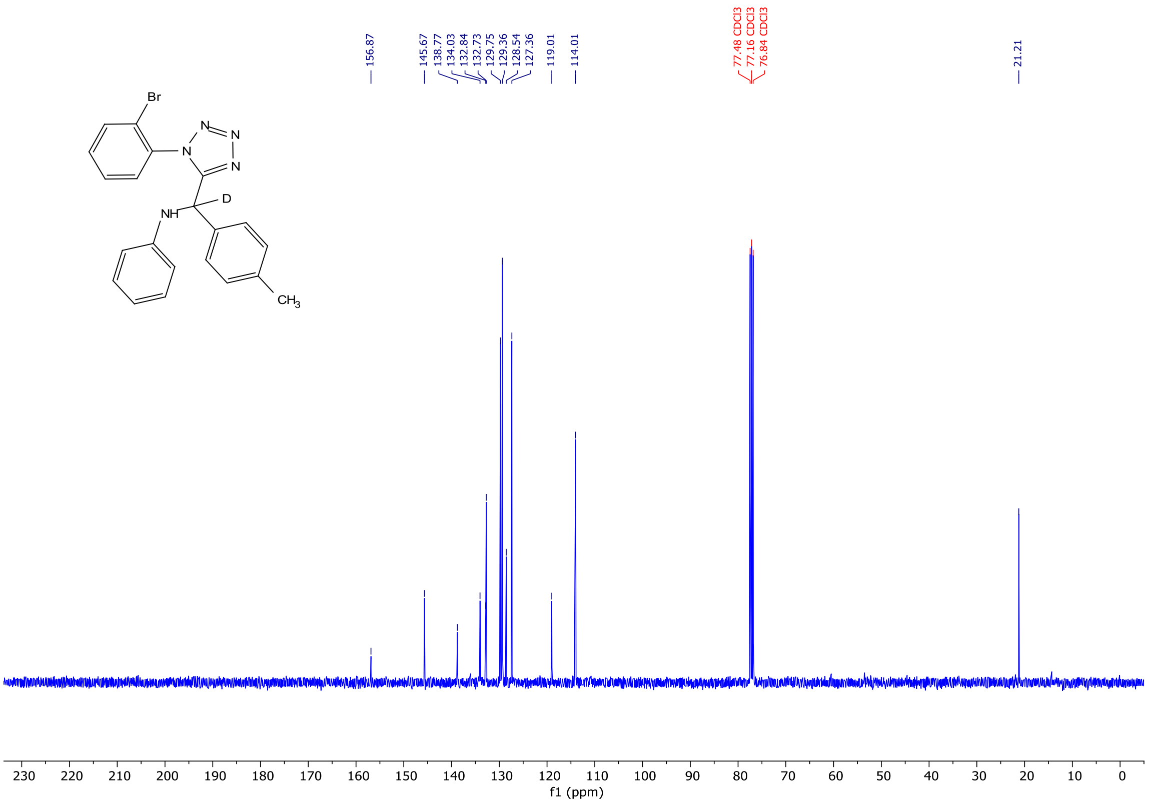

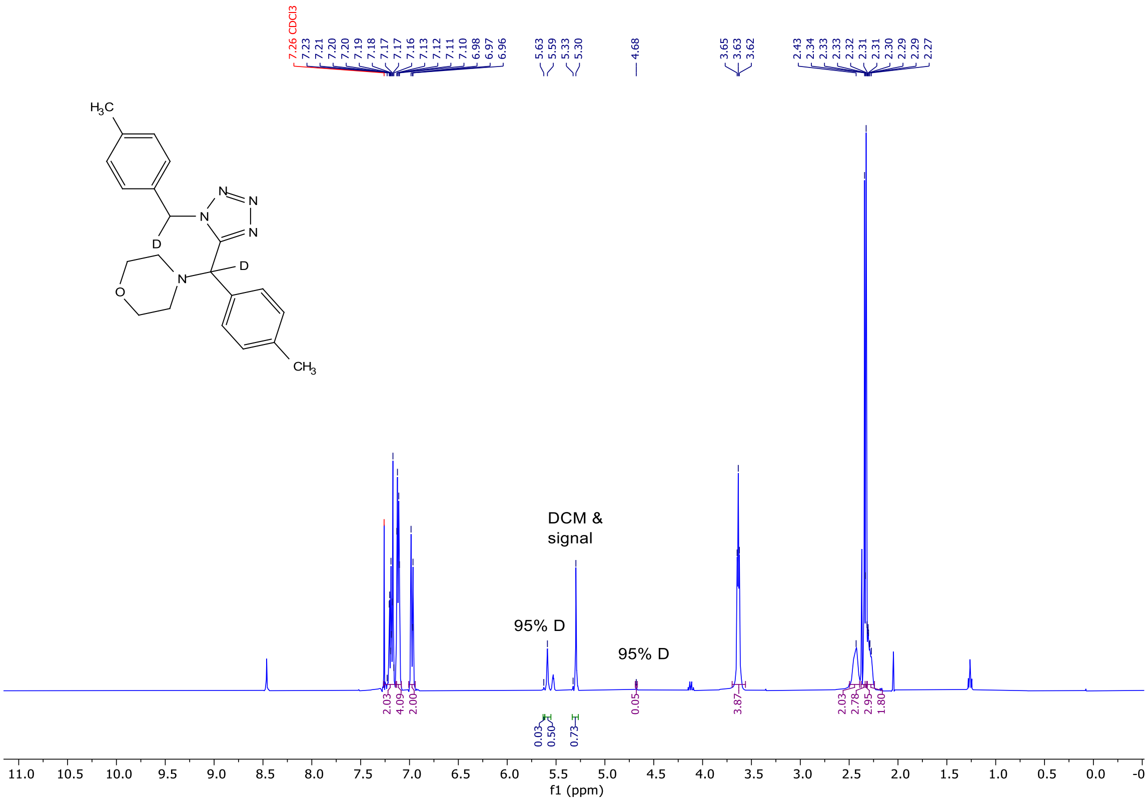

Figure S37. ^{1}H NMR (400 MHz, $CDCl_3$) of **3g**.

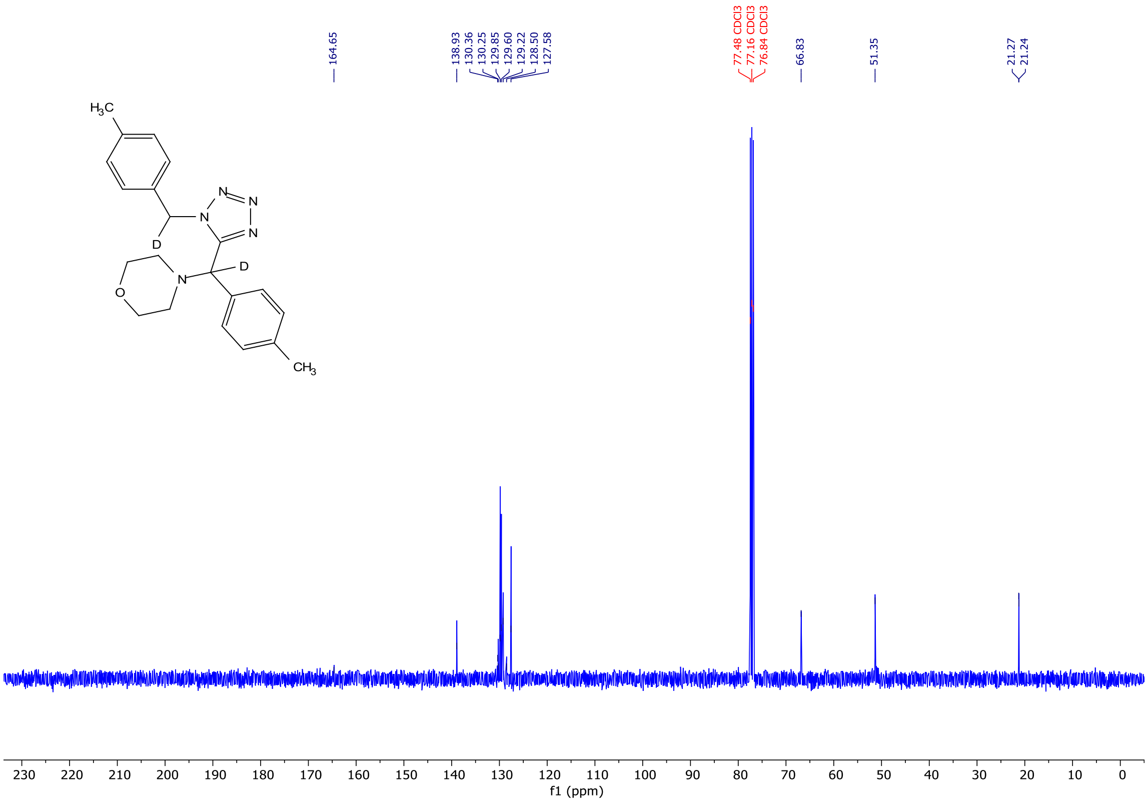

Figure S38. ^{13}C NMR (101 MHz, $CDCl_3$) of **3g**.

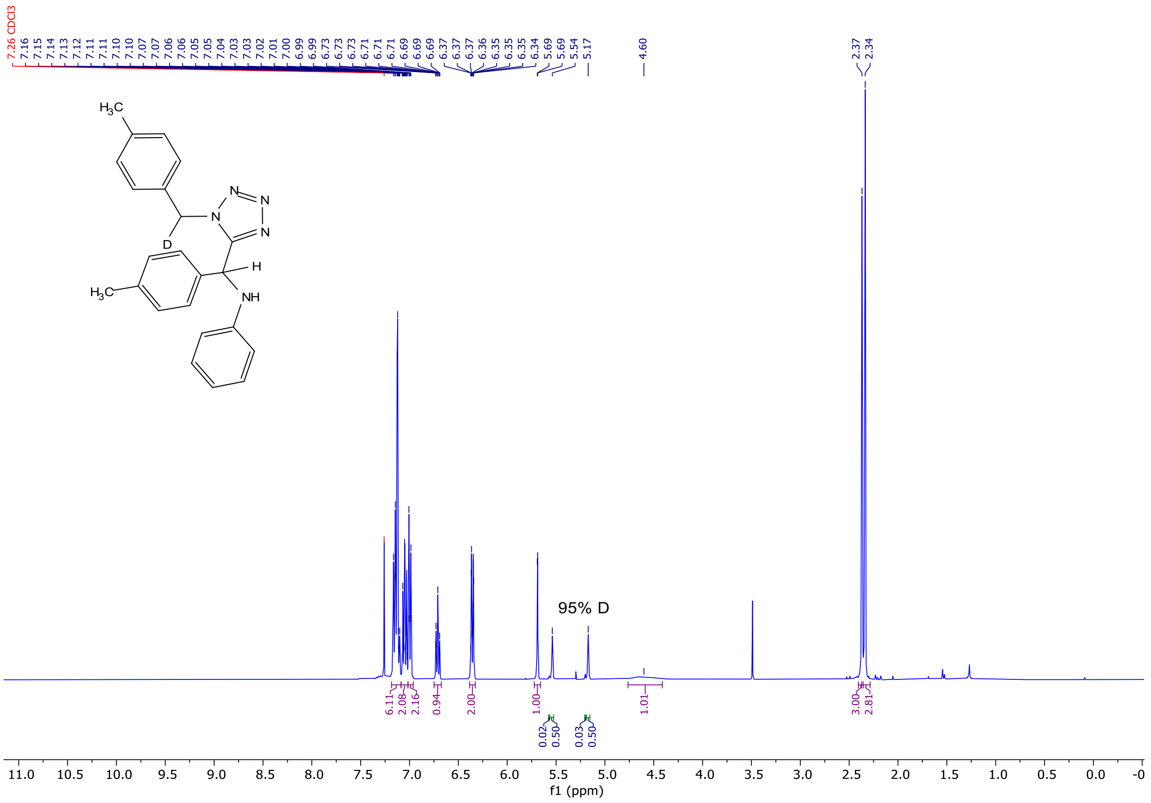

Figure S39. ^{1}H NMR (400 MHz, $CDCl_3$) of **3h**.

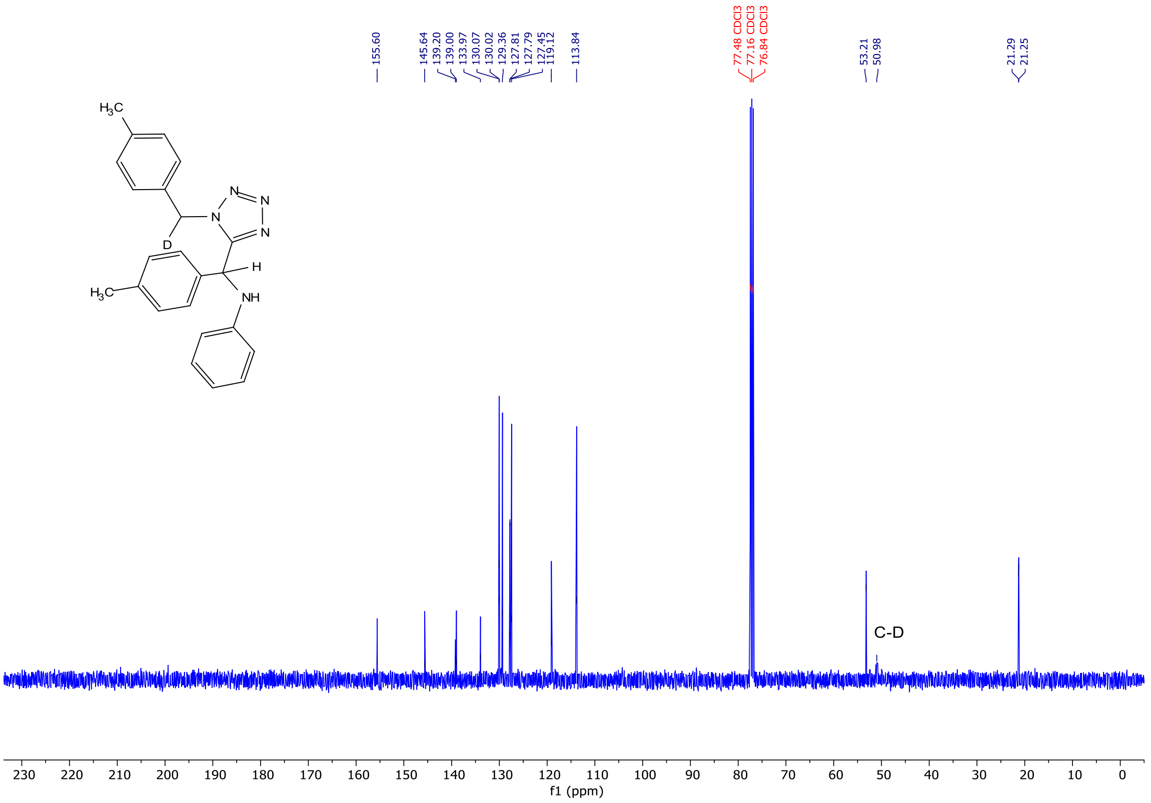

Figure S40. ^{13}C NMR (101 MHz, $CDCl_3$) of **3h**.

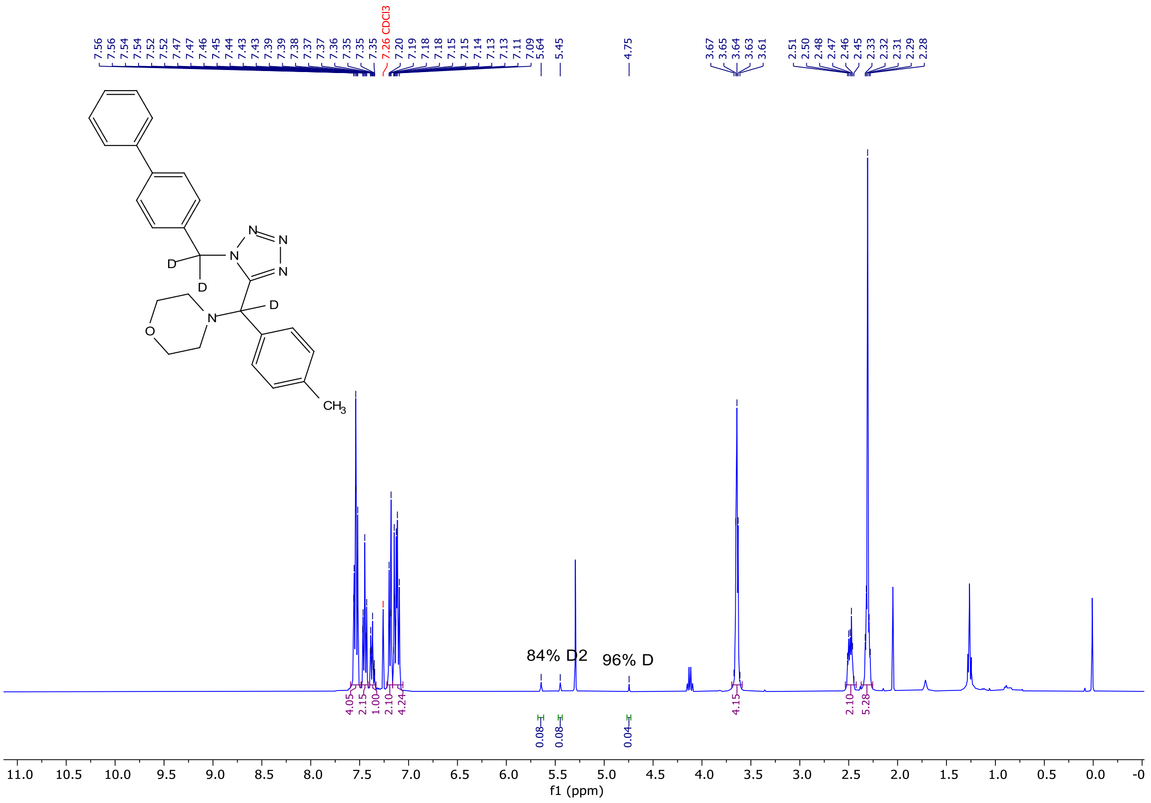

Figure S41. ^{1}H NMR (400 MHz, $CDCl_3$) of **3i**.

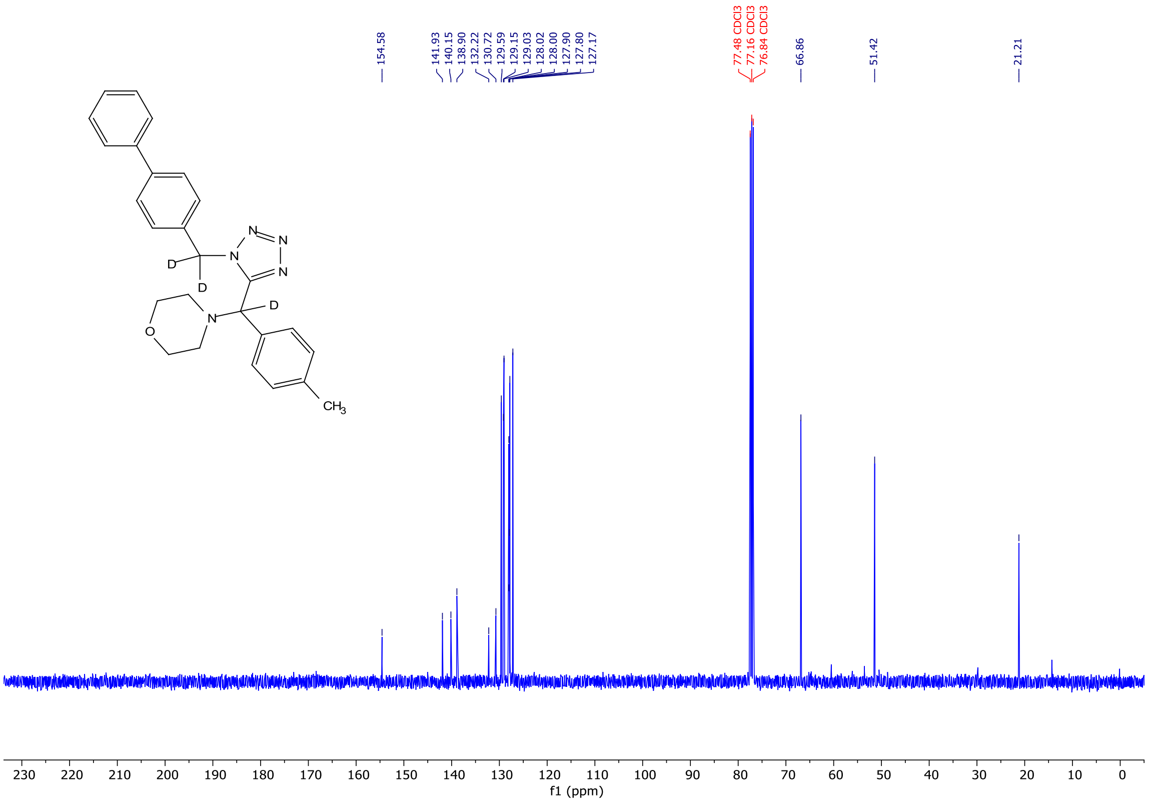

Figure S42. ^{13}C NMR (101 MHz, $CDCl_3$) of **3i**.

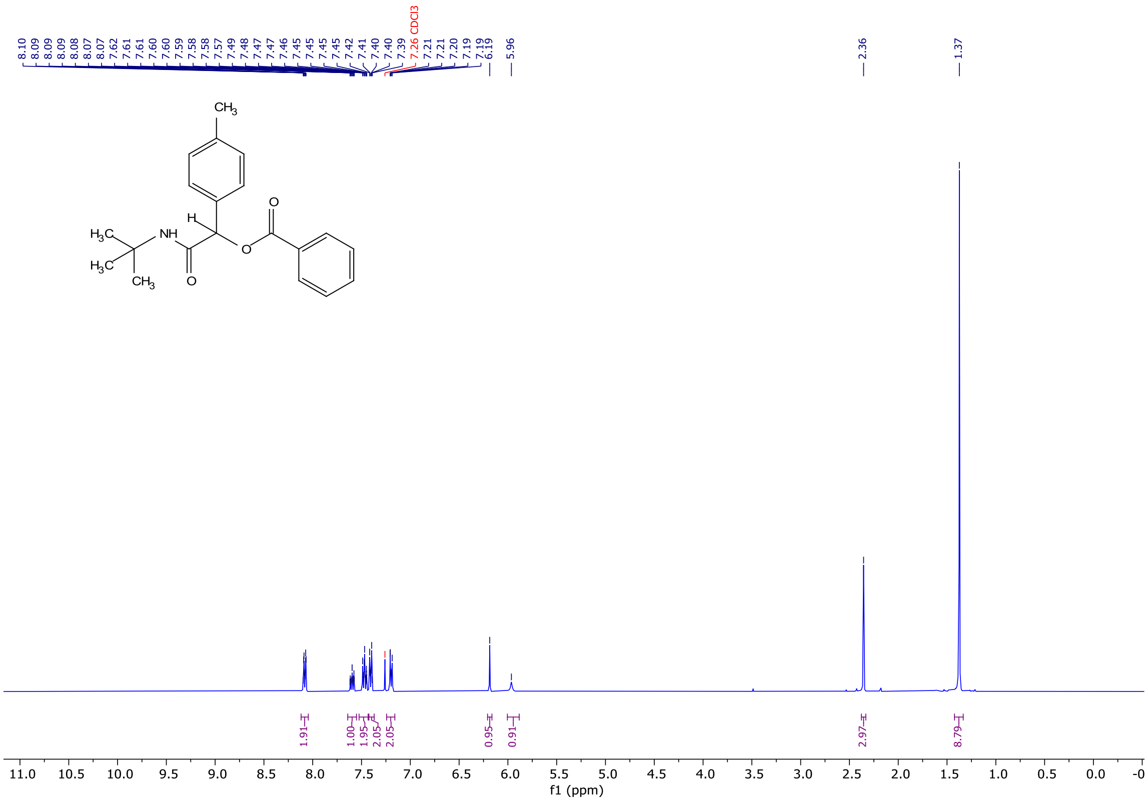

Figure S43. ^{1}H NMR (400 MHz, $CDCl_3$) of **4a**.

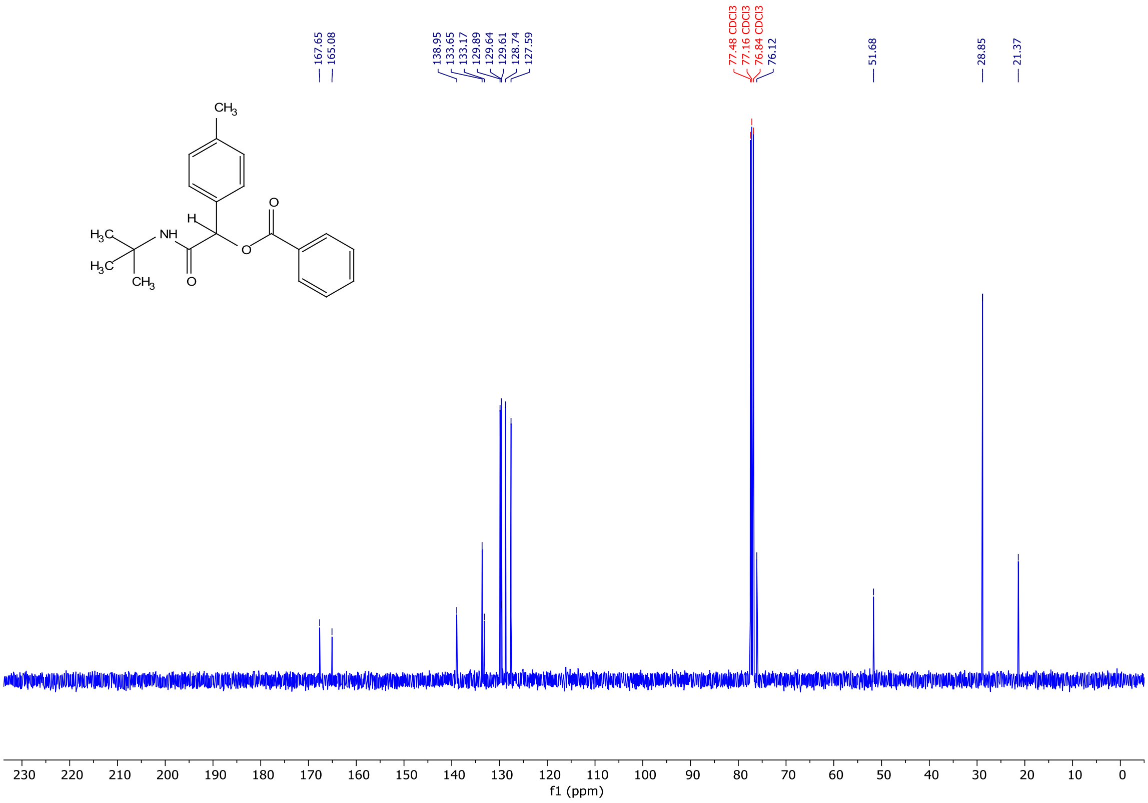

Figure S44. ^{1}H NMR (400 MHz, $CDCl_3$) of **4a**.

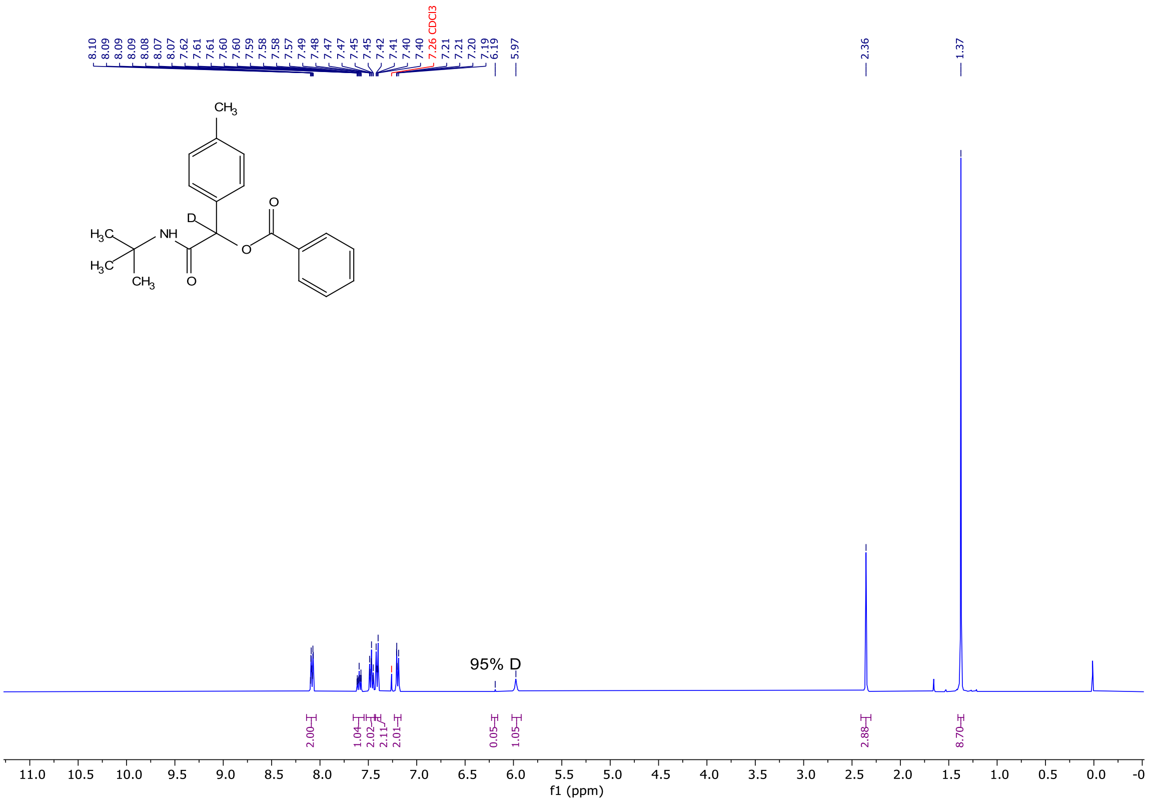

Figure S45. ^{1}H NMR (400 MHz, $CDCl_3$) of **4b**.

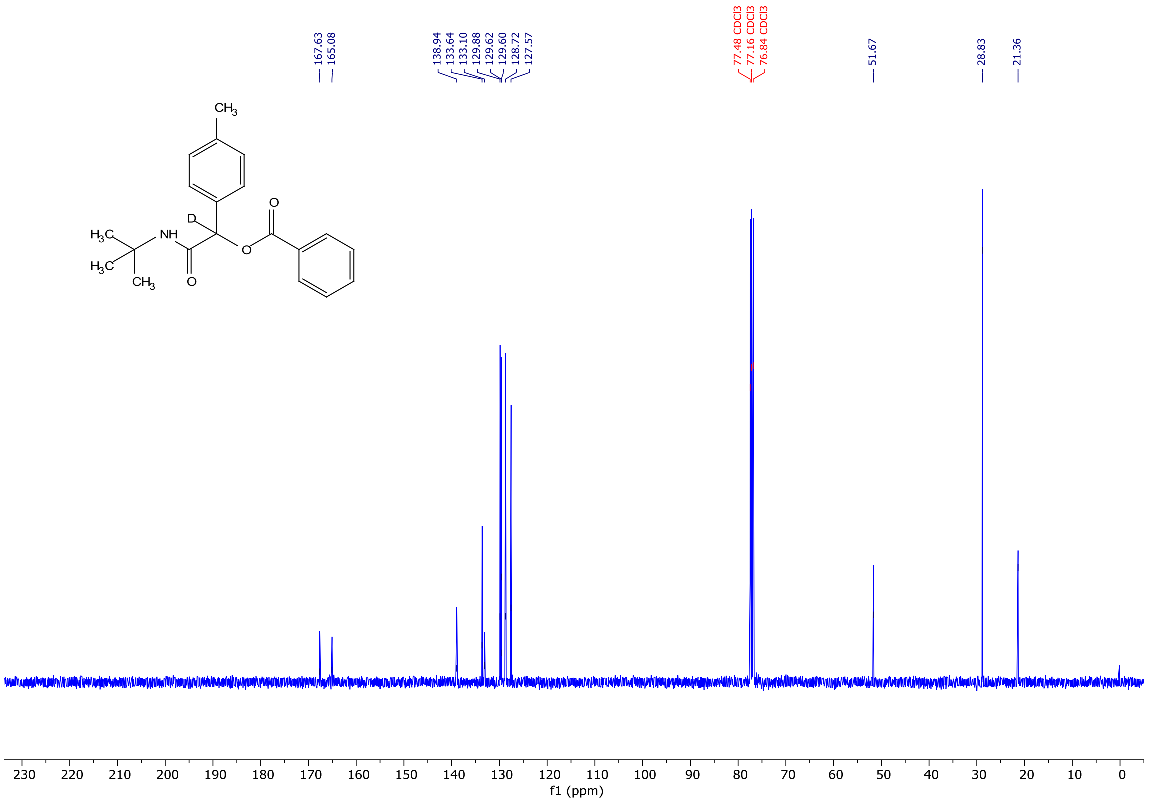

Figure S46. ^{13}C NMR (101 MHz, $CDCl_3$) of **4b**.

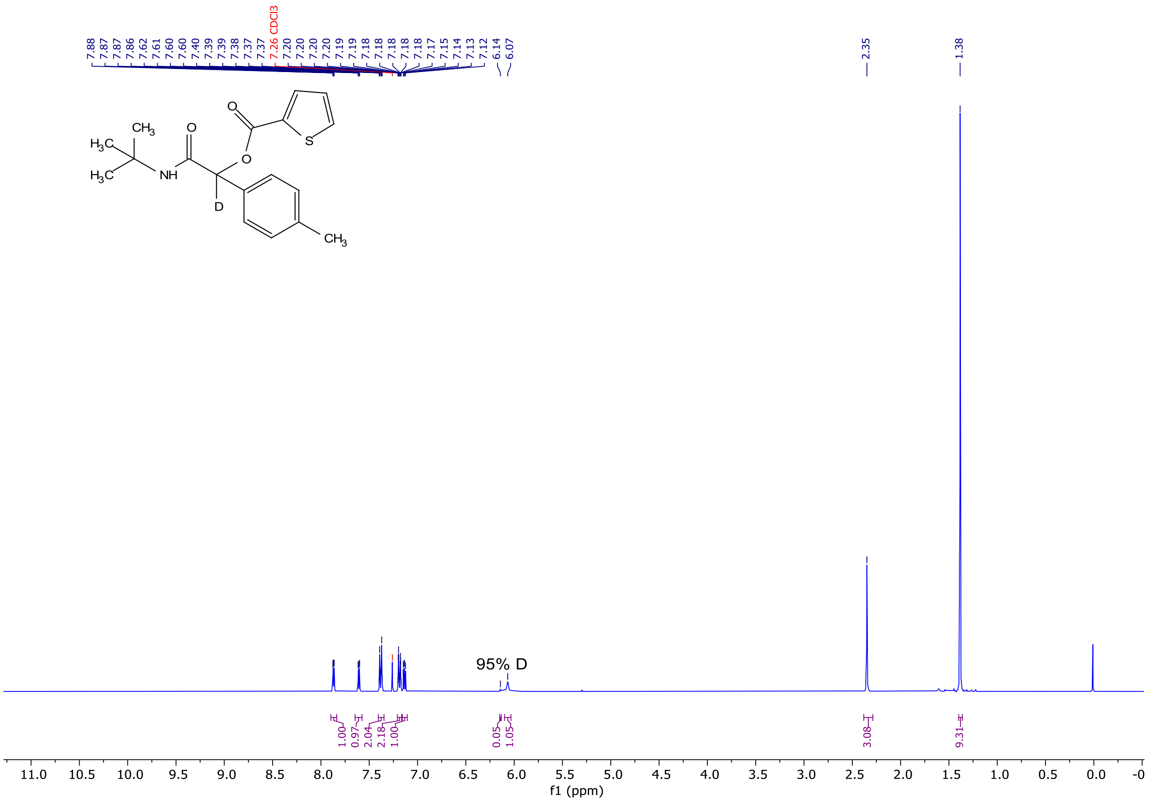

Figure S47. ^{1}H NMR (400 MHz, $CDCl_3$) of **4c**.

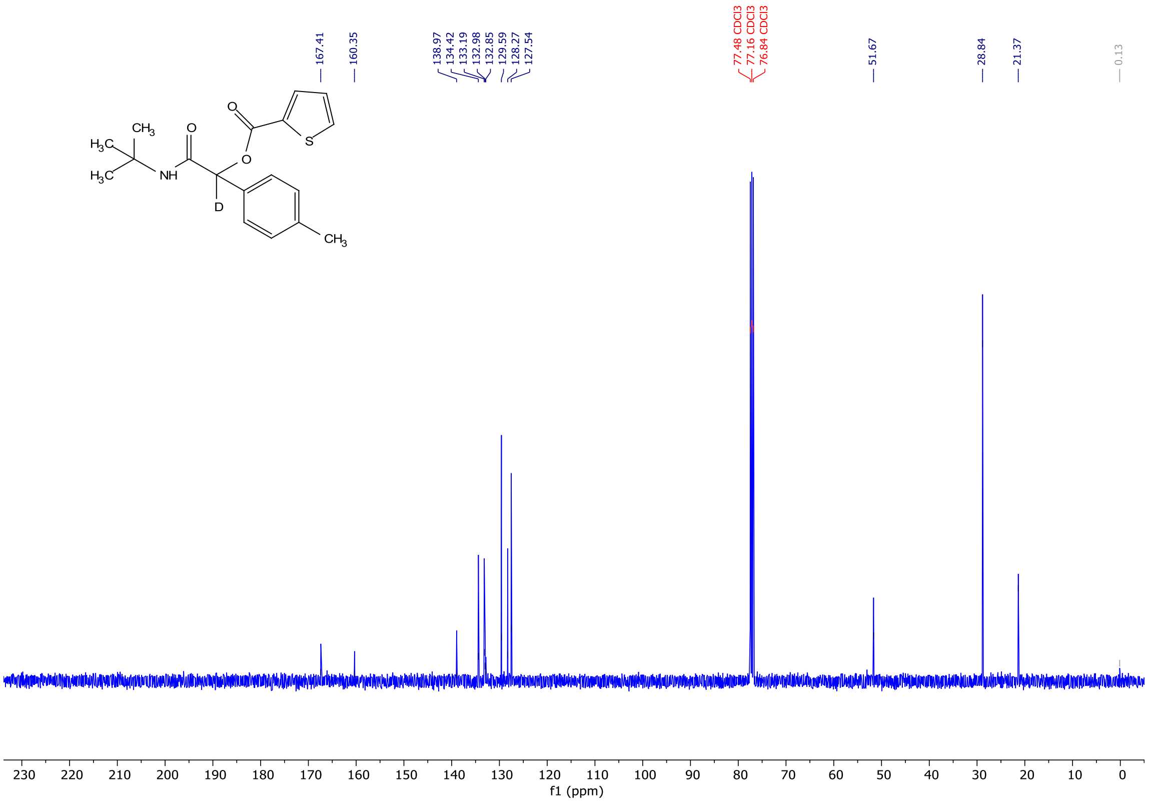

Figure S48. ^{13}C NMR (101 MHz, $CDCl_3$) of **4c**.

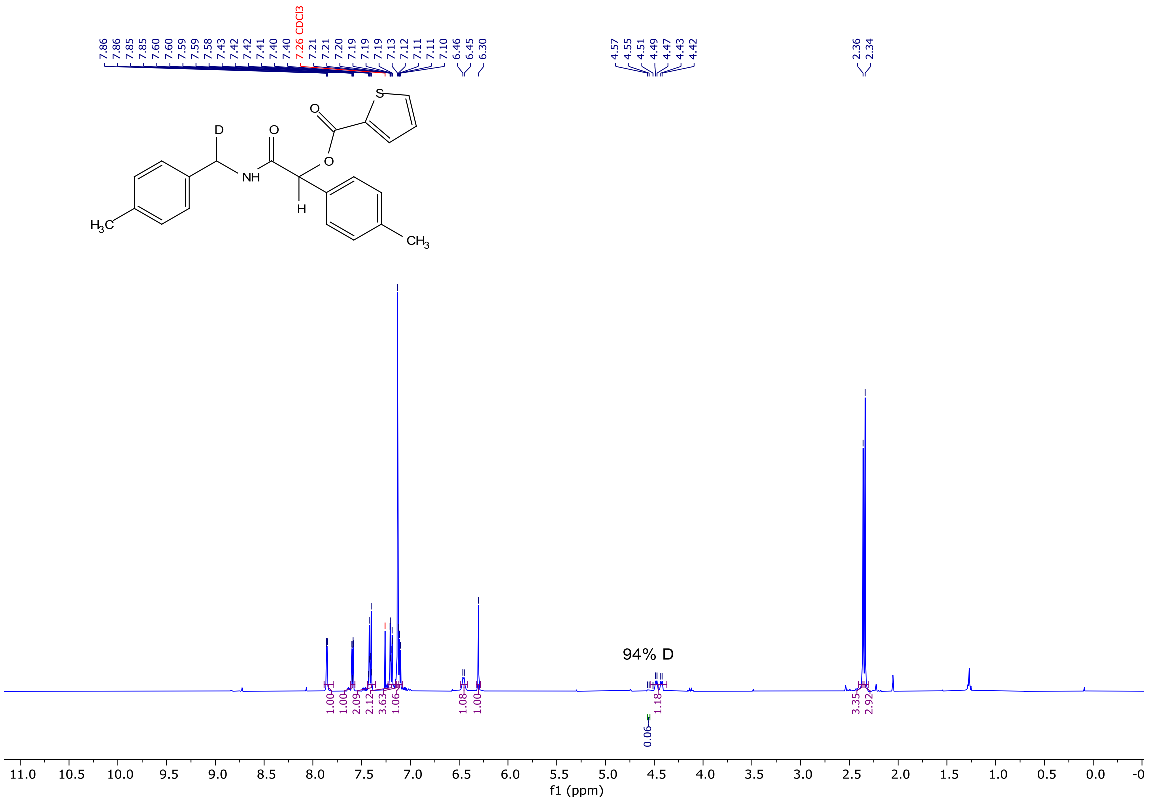

Figure S49. ^{1}H NMR (400 MHz, $CDCl_3$) of **4d**.

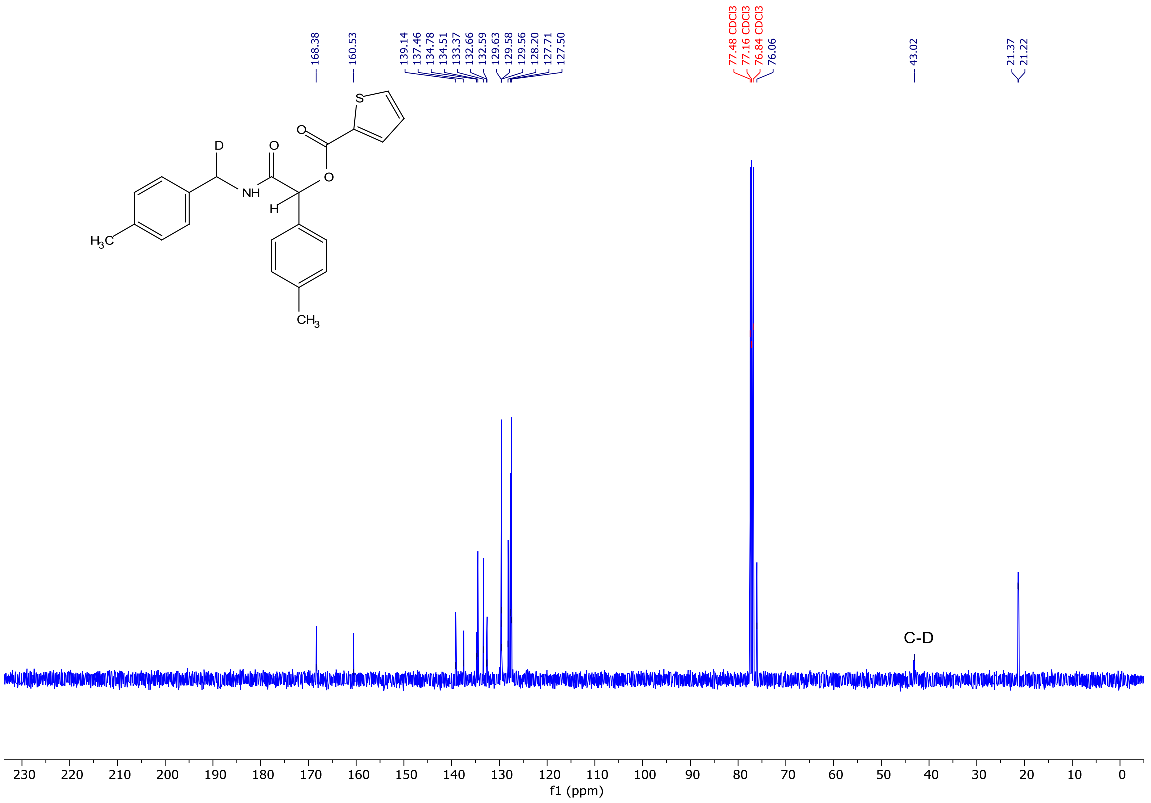

Figure S50. ^{13}C NMR (101 MHz, $CDCl_3$) of **4d**.

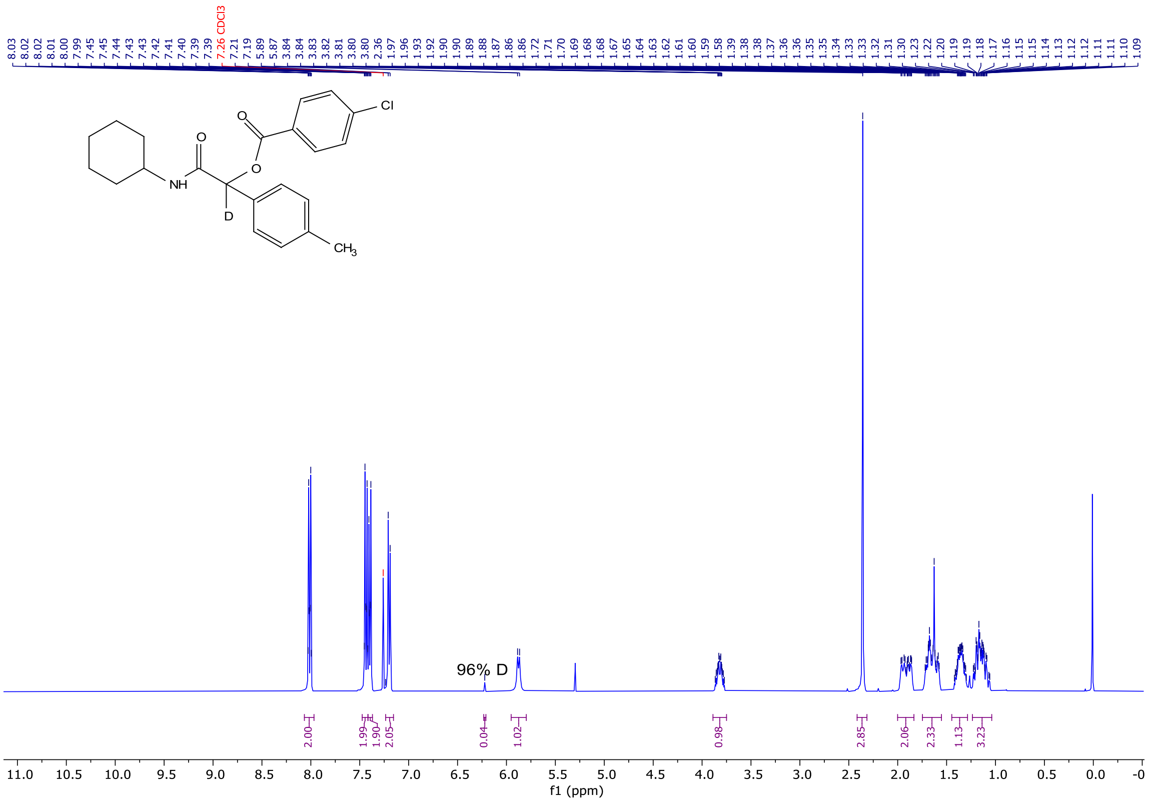

Figure S51. ^{1}H NMR (400 MHz, $CDCl_3$) of **4e**.

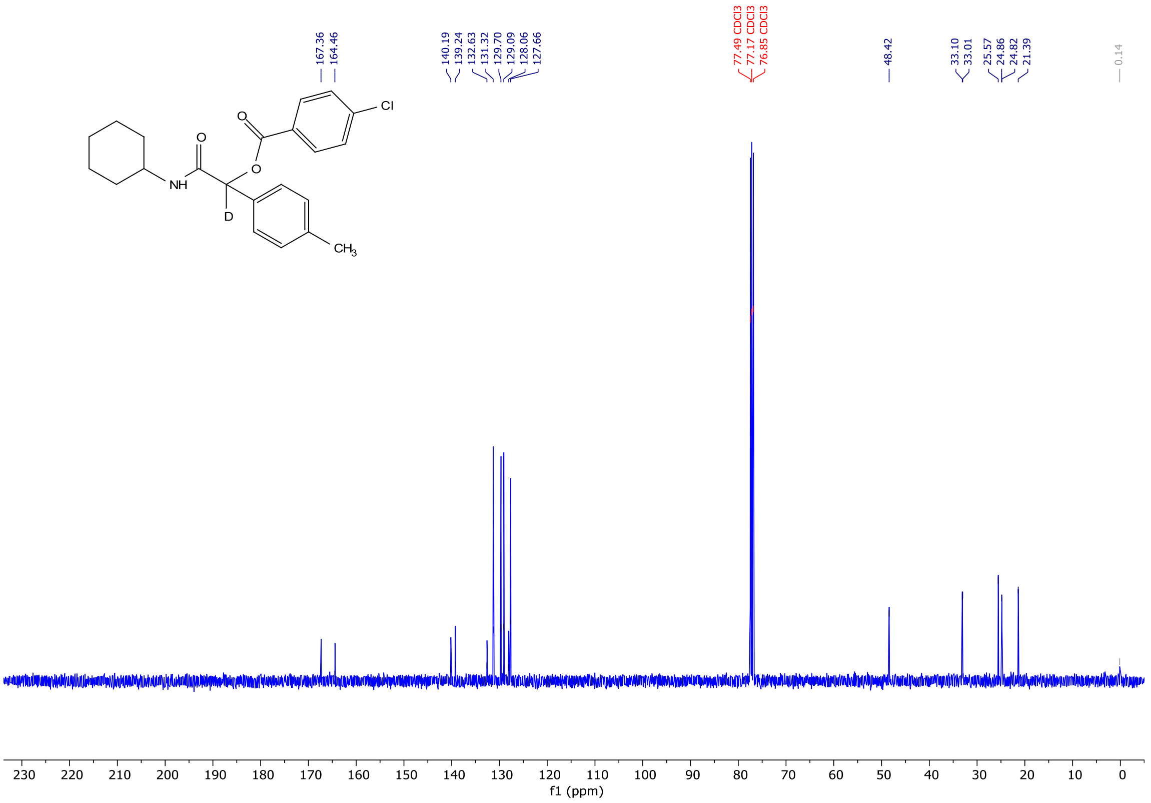

Figure S52. ^{13}C NMR (101 MHz, $CDCl_3$) of **4e**.

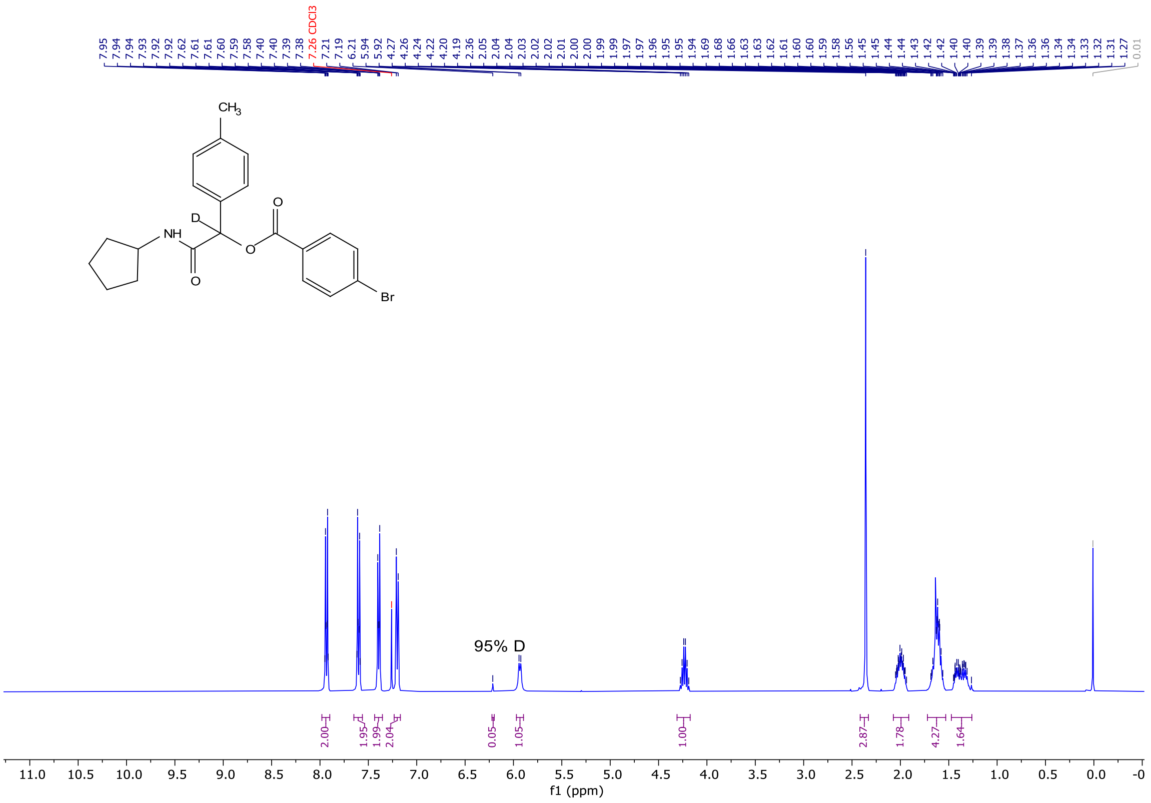

Figure S53. ^{1}H NMR (400 MHz, $CDCl_3$) of **4f**.

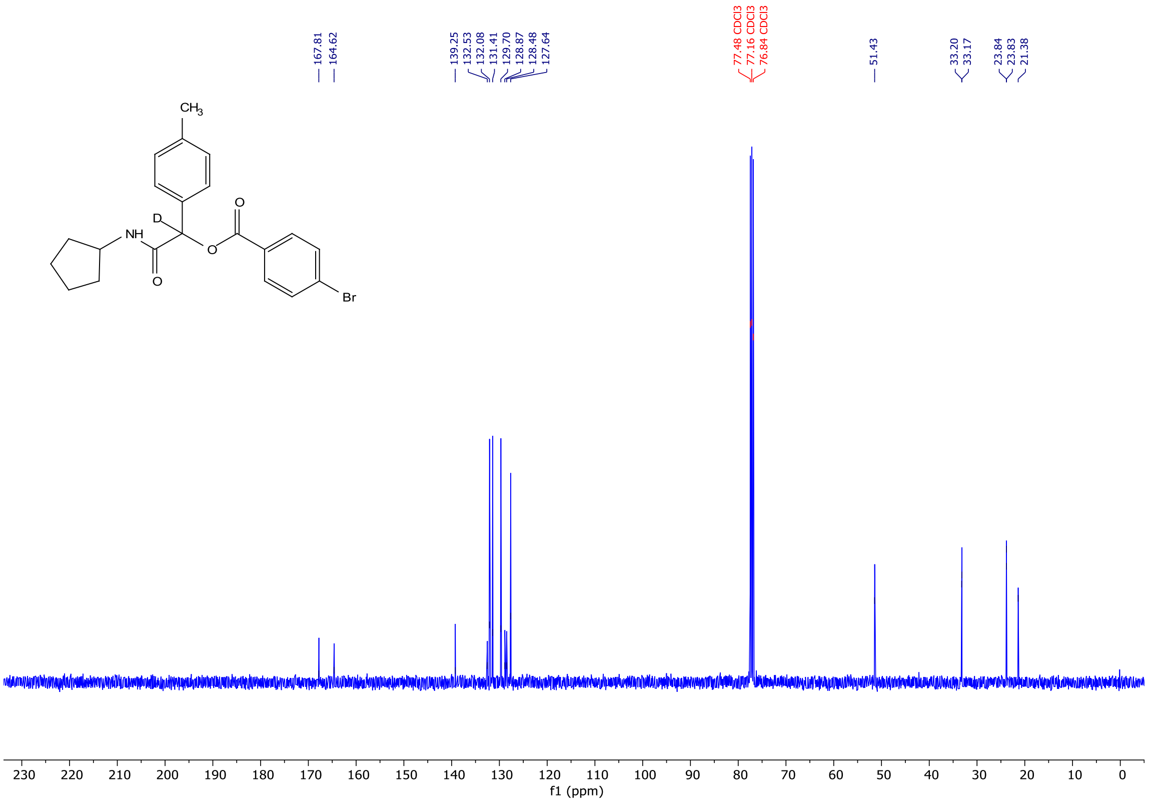

Figure S54. ^{13}C NMR (101 MHz, $CDCl_3$) of **4f**.

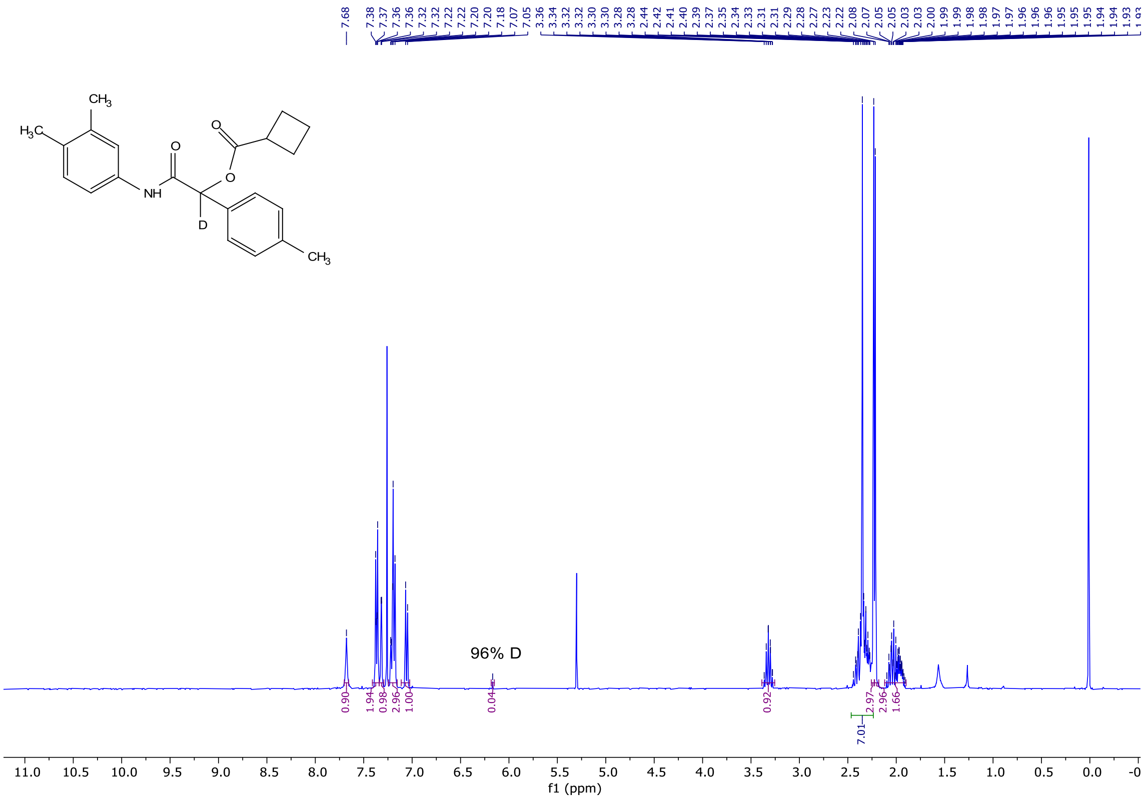

Figure S55. ^{1}H NMR (400 MHz, $CDCl_3$) of **4g.**

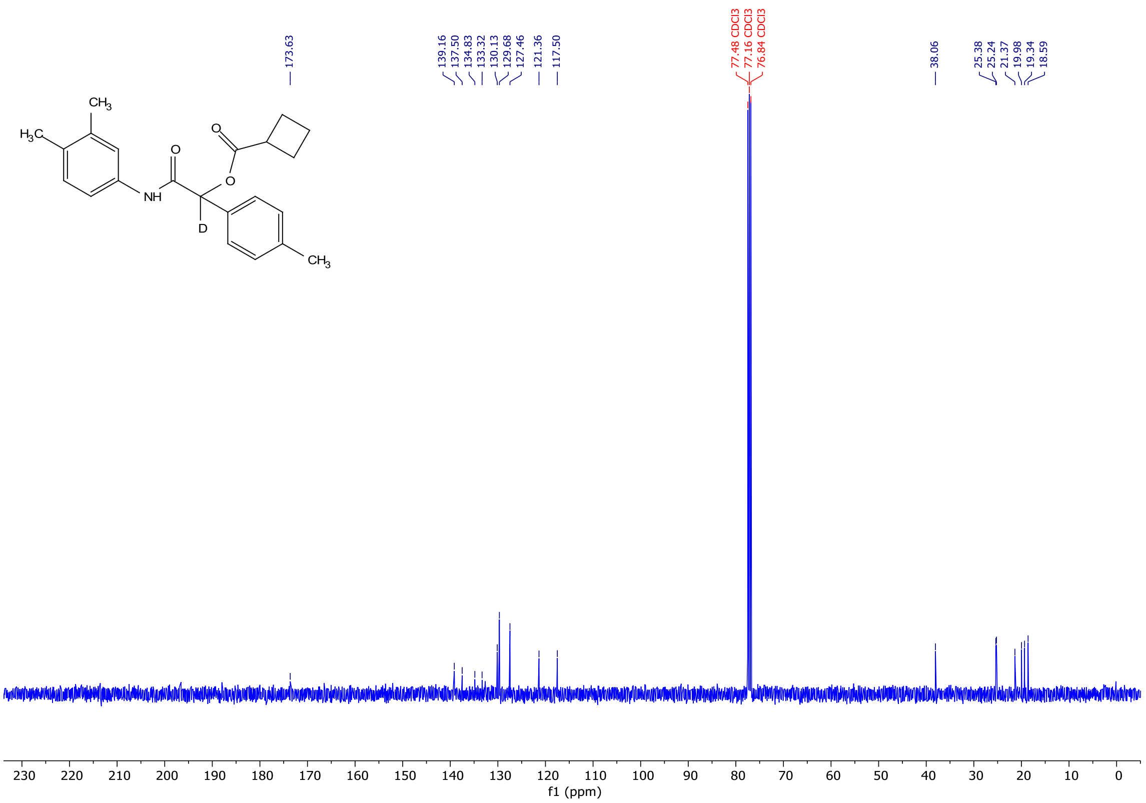

Figure S56. ^{13}C NMR (101 MHz, $CDCl_3$) of **4g**.

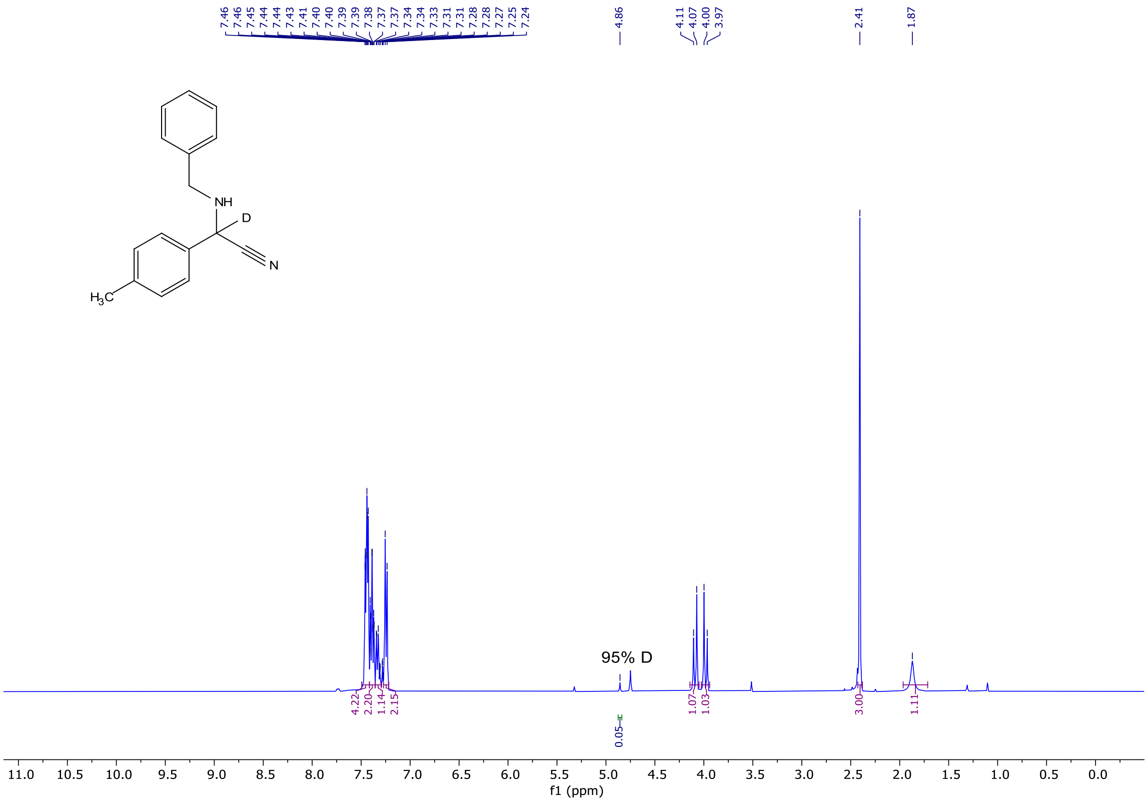

Figure S57. ^{1}H NMR (400 MHz, $CDCl_3$) of **5a**.

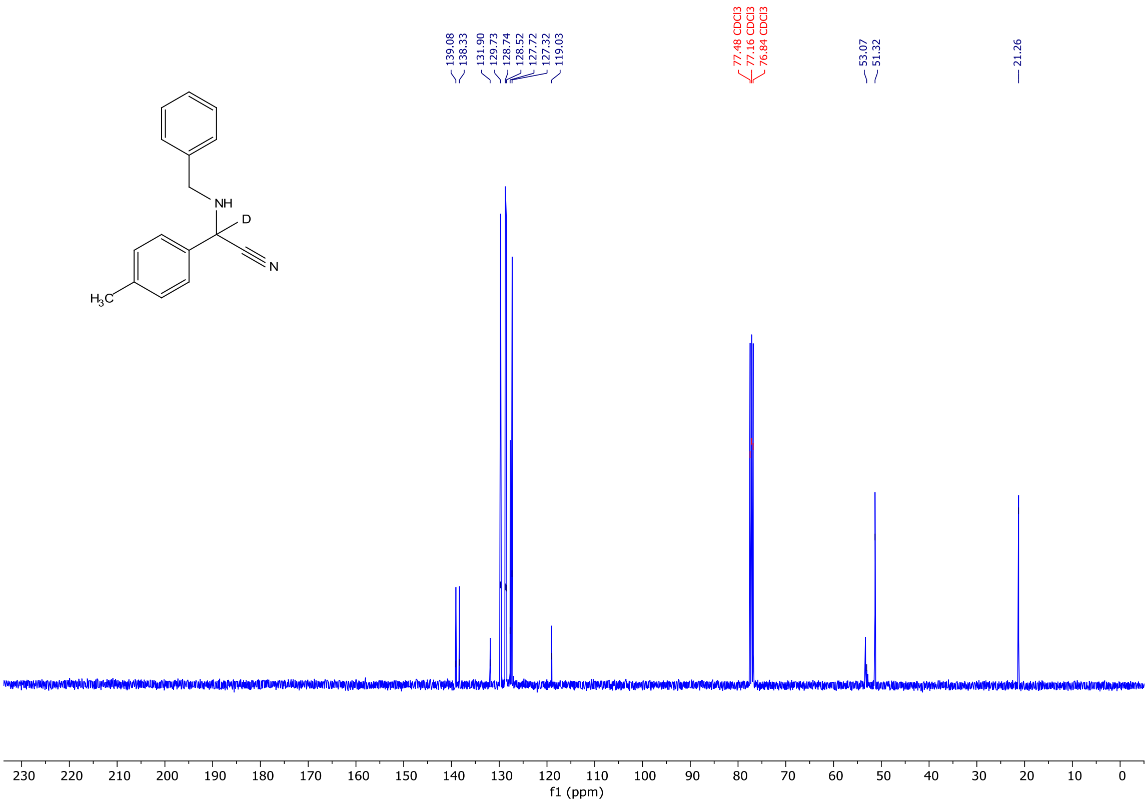

Figure S58. ^{13}C NMR (101 MHz, $CDCl_3$) of **5a**.

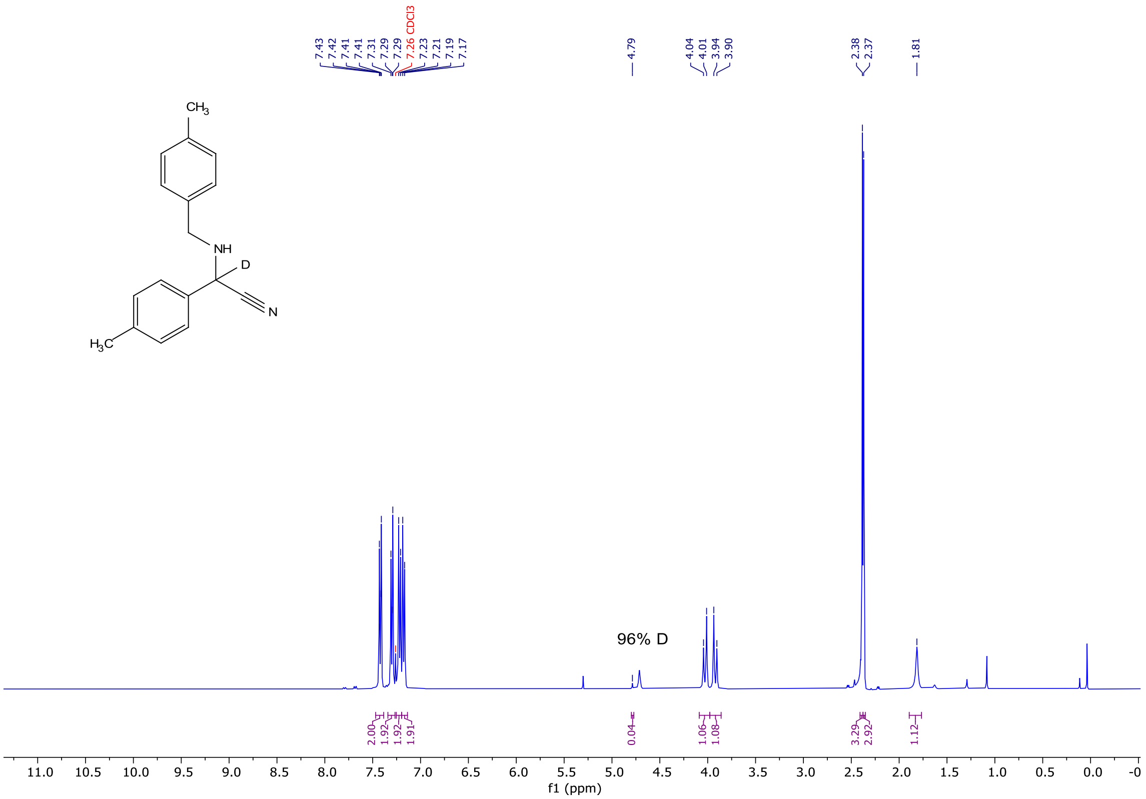

Figure S57. ^{1}H NMR (400 MHz, $CDCl_3$) of **5b**.

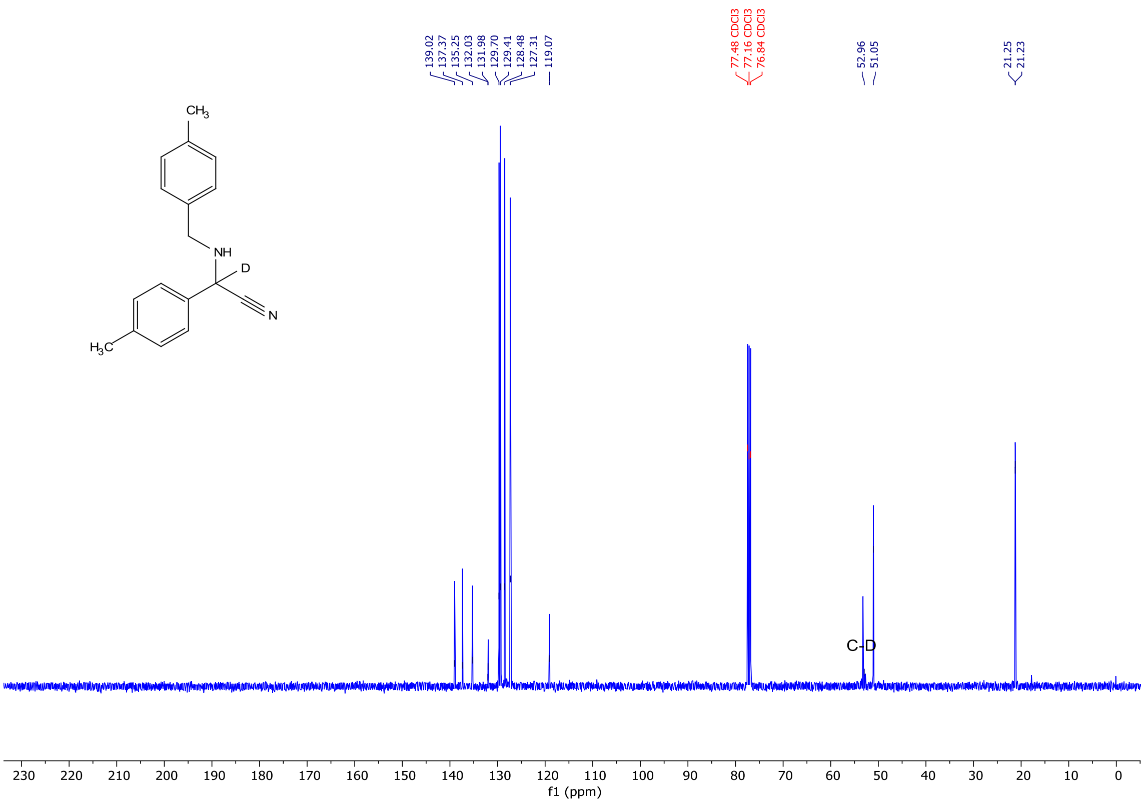

Figure S58. ^{13}C NMR (101 MHz, $CDCl_3$) of **5b**.

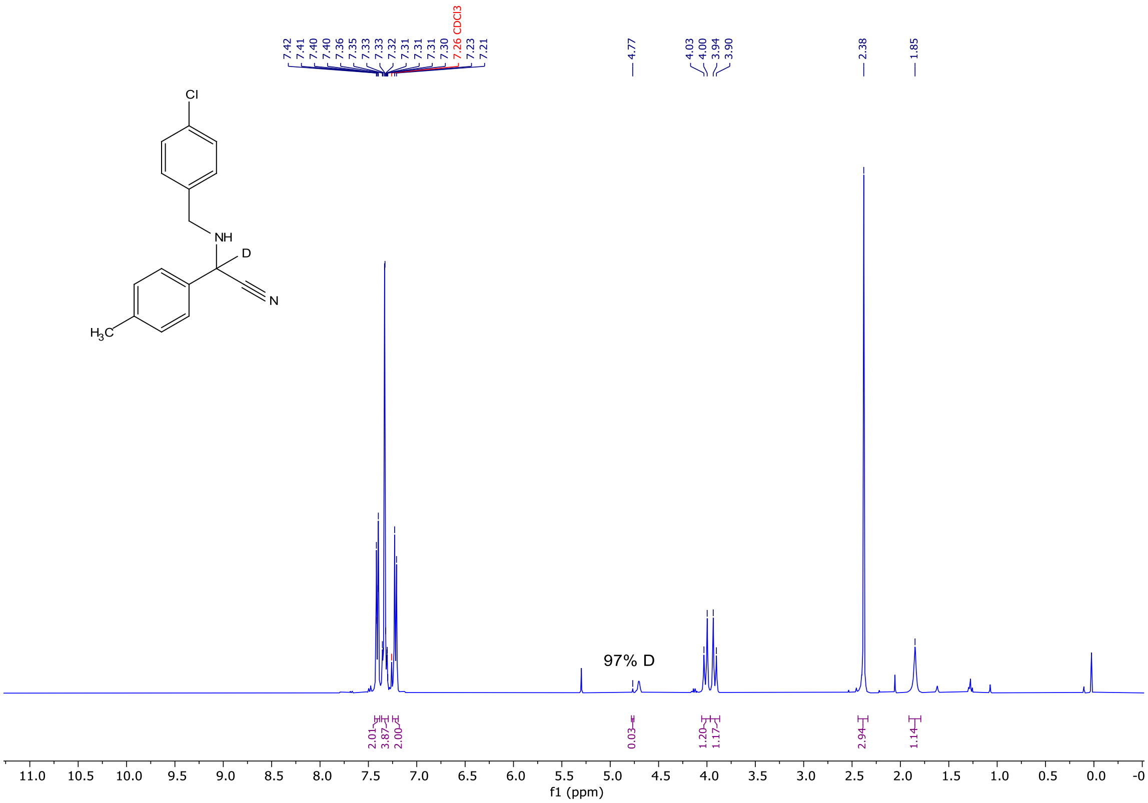

Figure S59. ^{1}H NMR (400 MHz, $CDCl_3$) of **5c**.

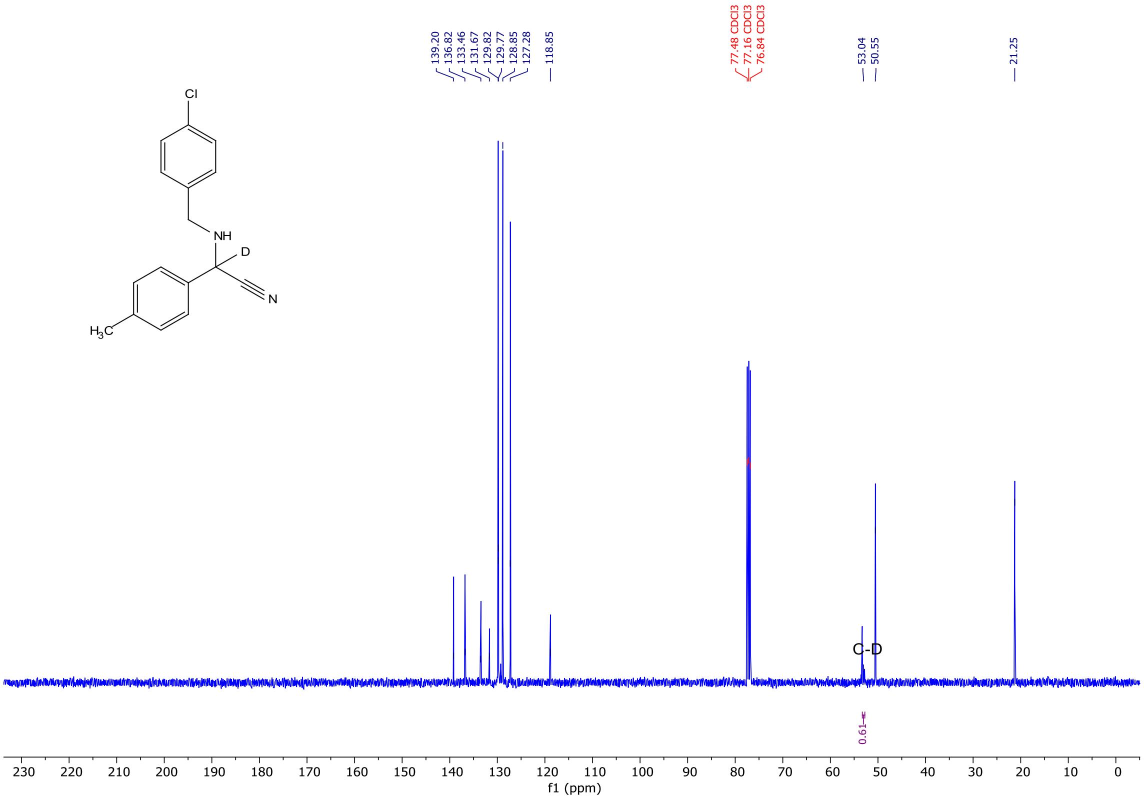

Figure S60. ^{13}C NMR (101 MHz, $CDCl_3$) of **5c**.

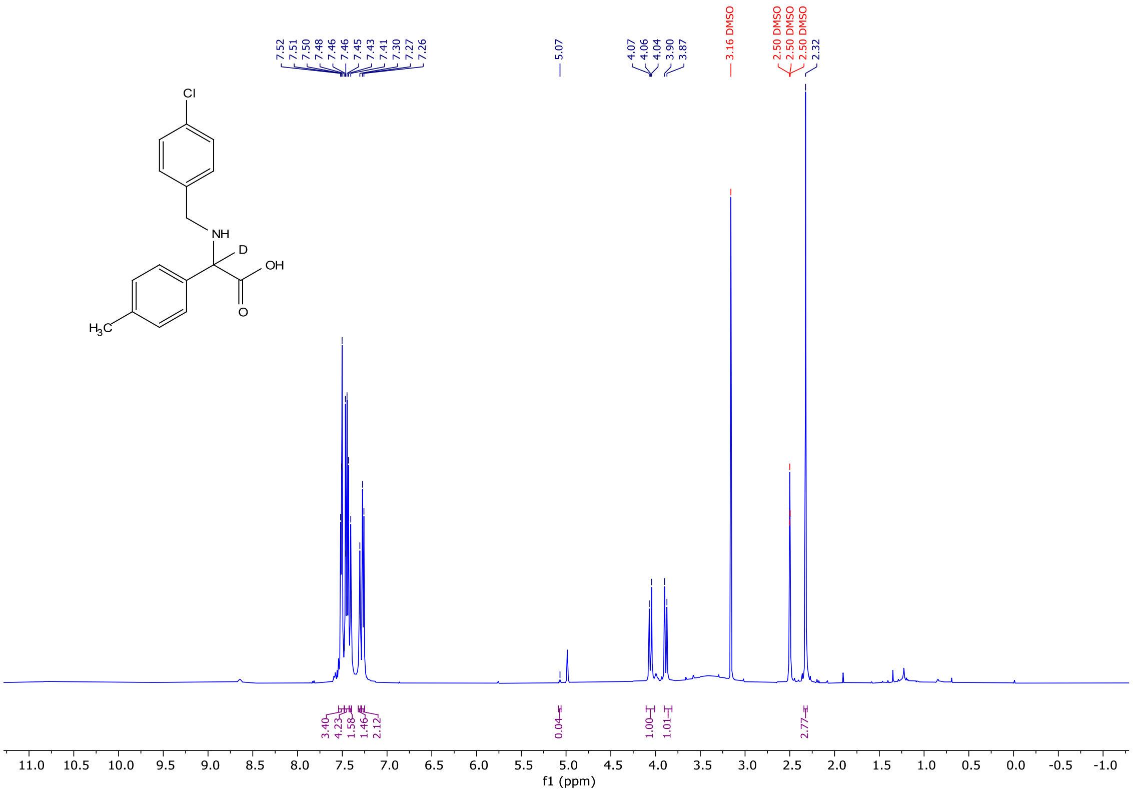

Figure S61. ^{1}H NMR (400 MHz, $CDCl_3$) of **5d**.

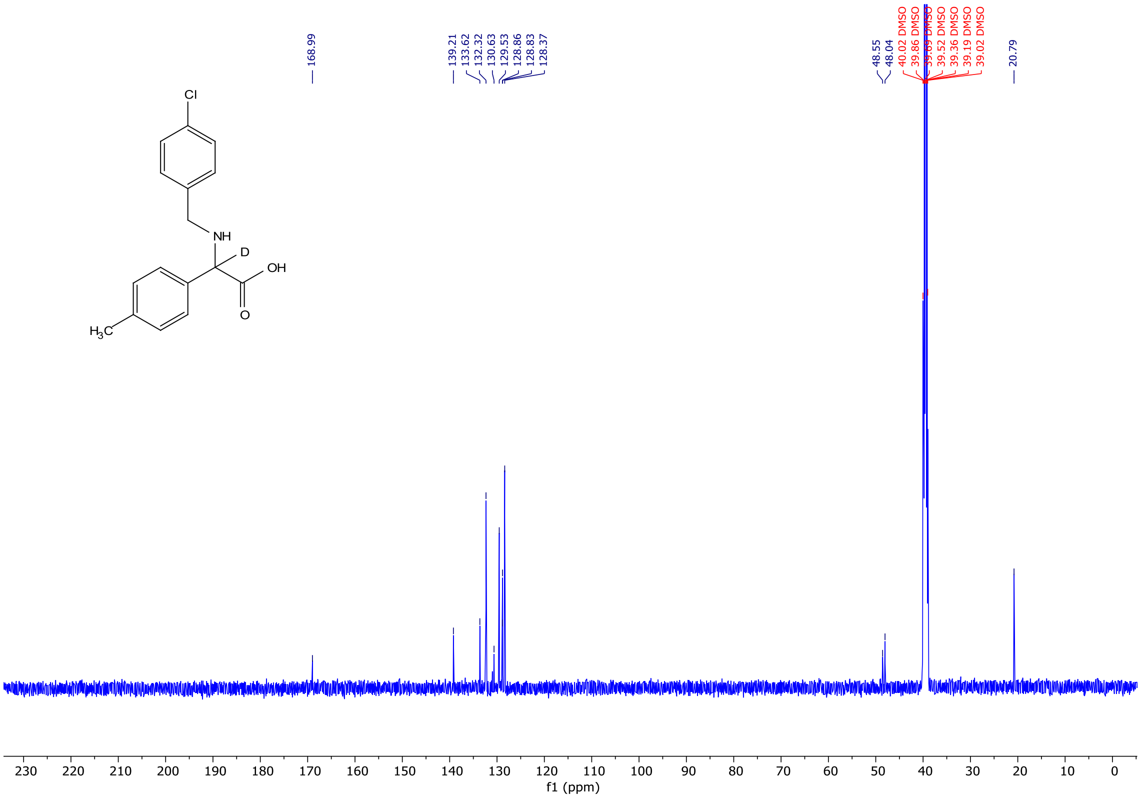

Figure S62. ^{13}C NMR (101 MHz, $CDCl_3$) of **5d.**

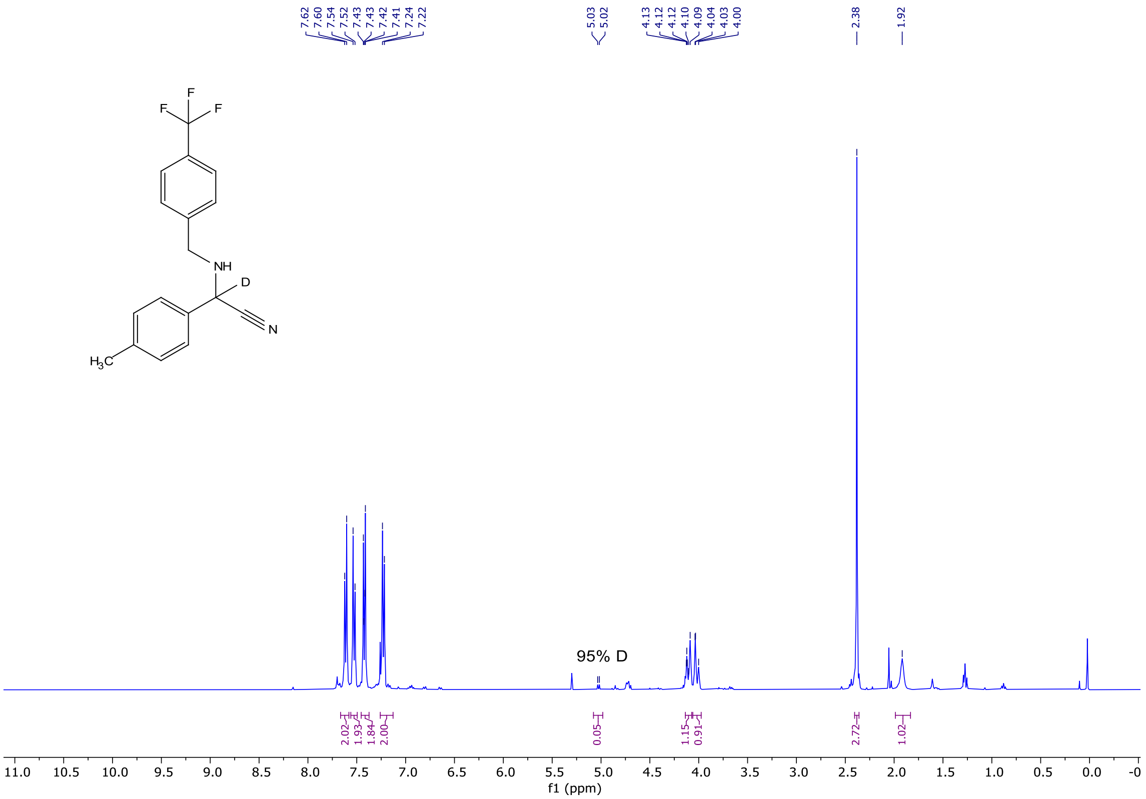

Figure S63. ^{1}H NMR (400 MHz, $CDCl_3$) of **5e**.

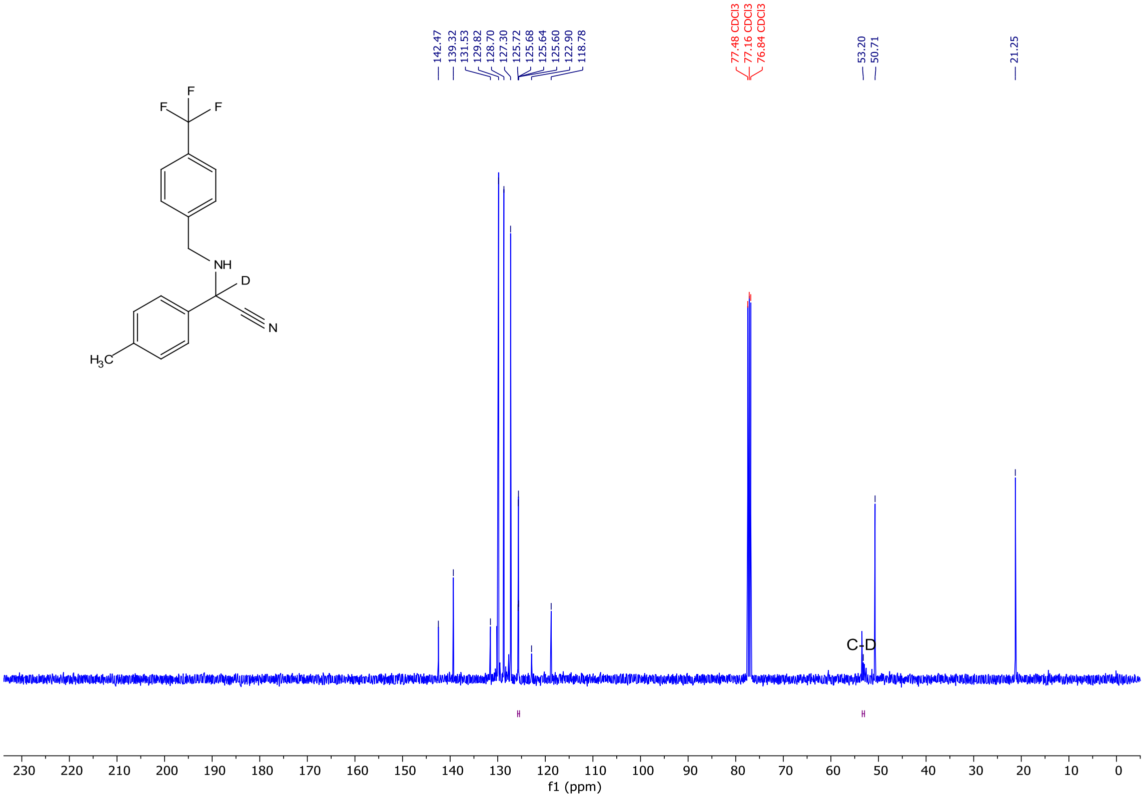

Figure S64. ^{13}C NMR (101 MHz, $CDCl_3$) of **5e**.

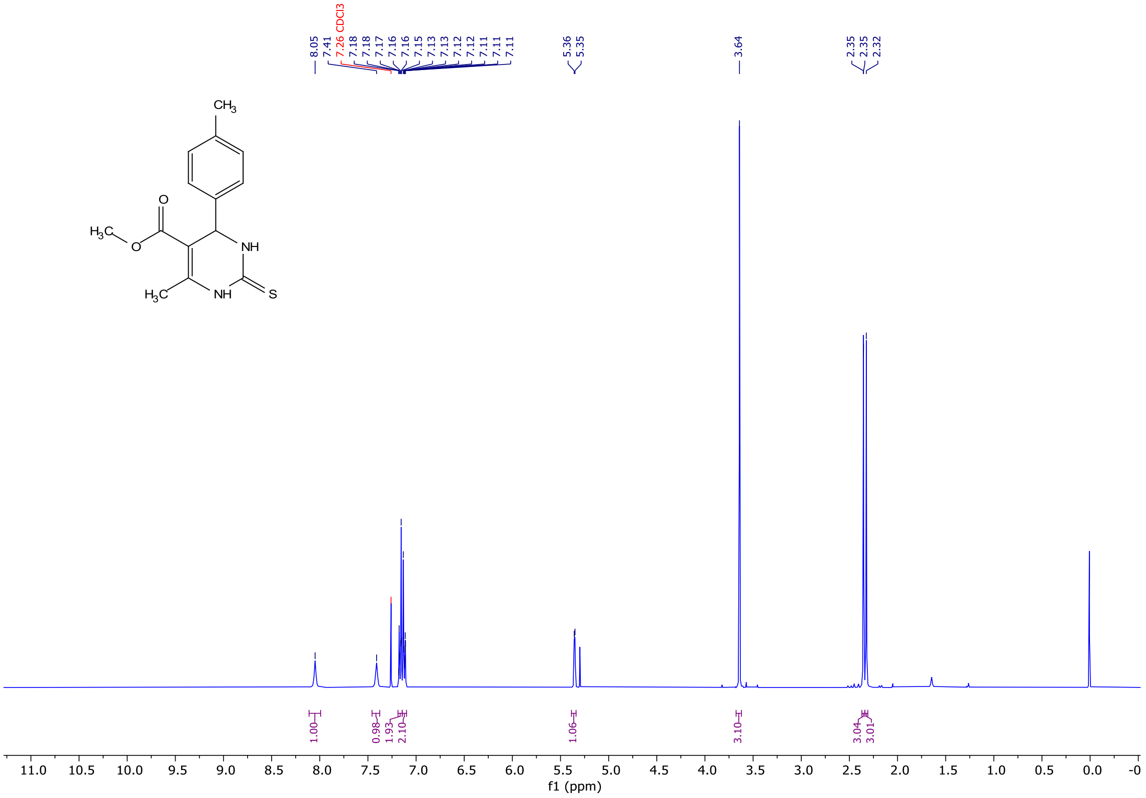

Figure S65. ^{1}H NMR (400 MHz, $CDCl_3$) of **6a**.

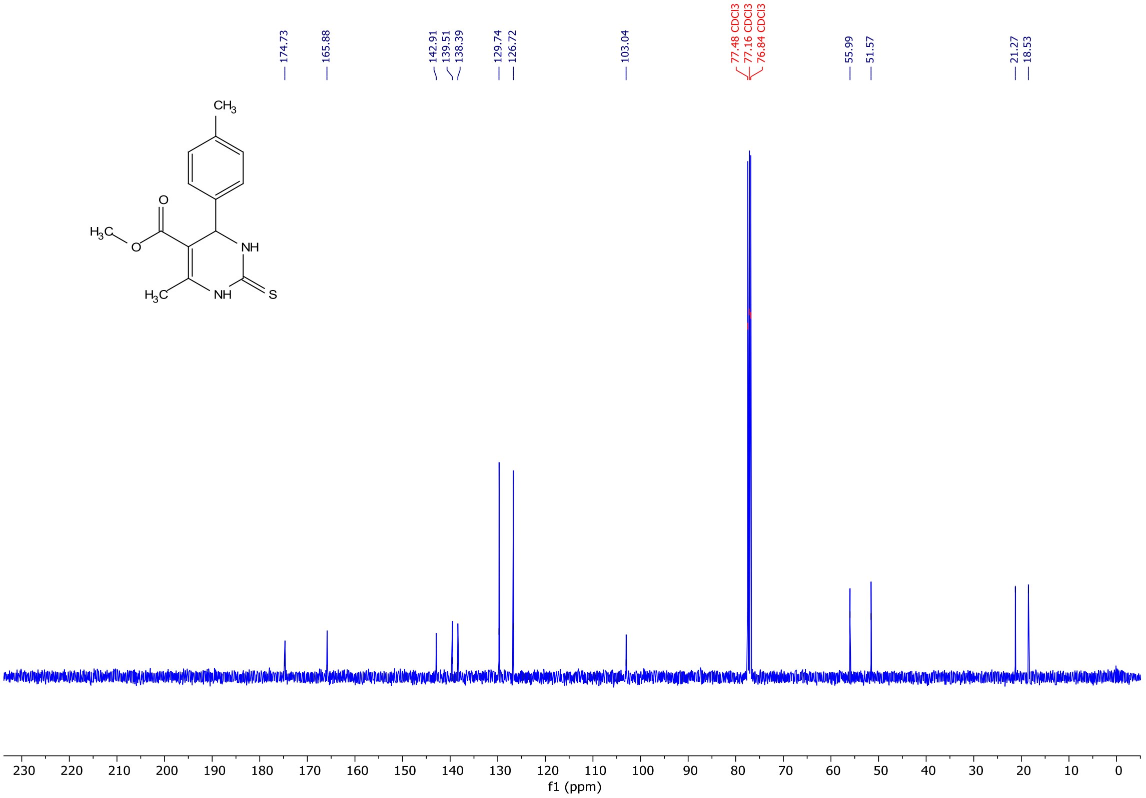

Figure S66. ^{13}C NMR (101 MHz, $CDCl_3$) of **6a**.

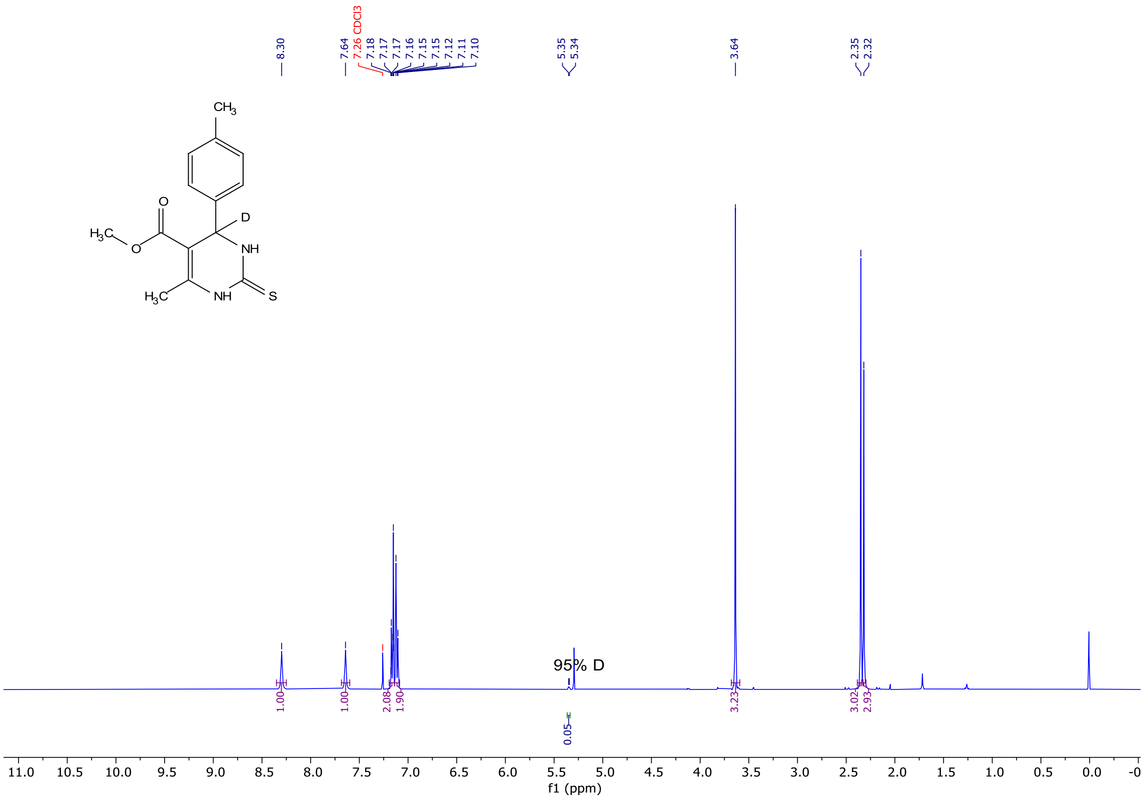

Figure S67. ^{1}H NMR (400 MHz, $CDCl_3$) of **6b**.

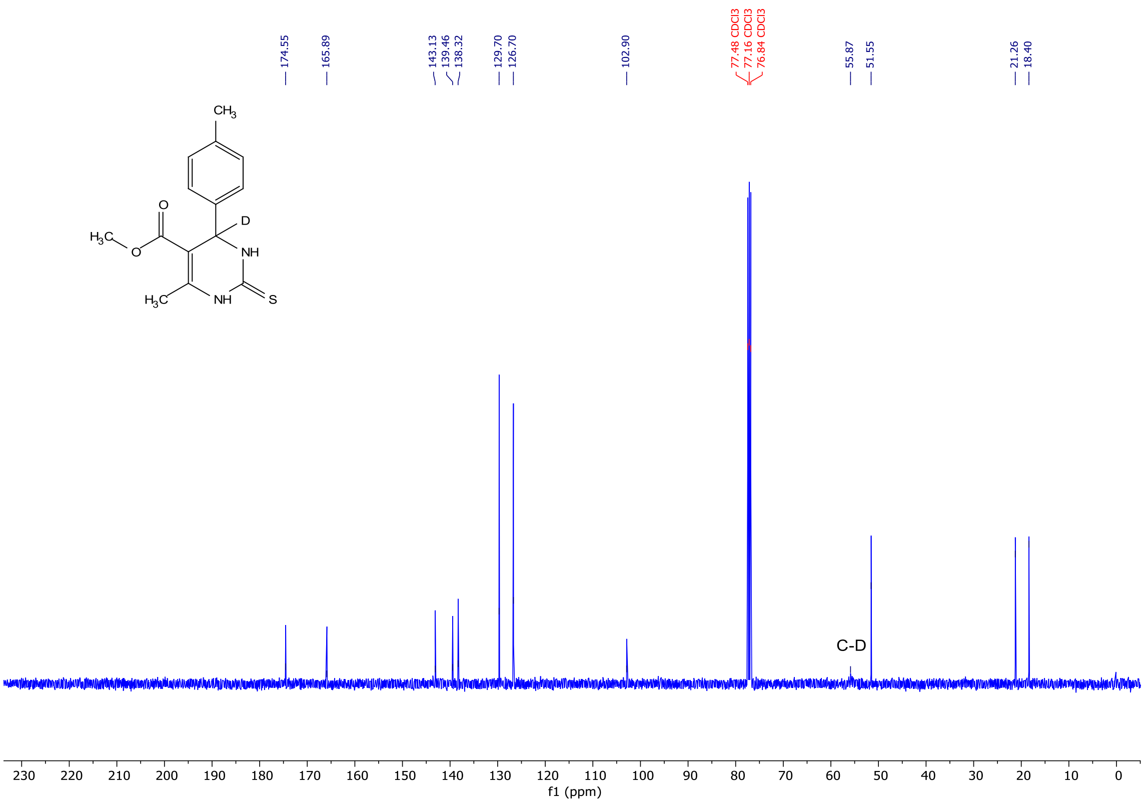

Figure S68. ^{13}C NMR (101 MHz, $CDCl_3$) of **6b**.

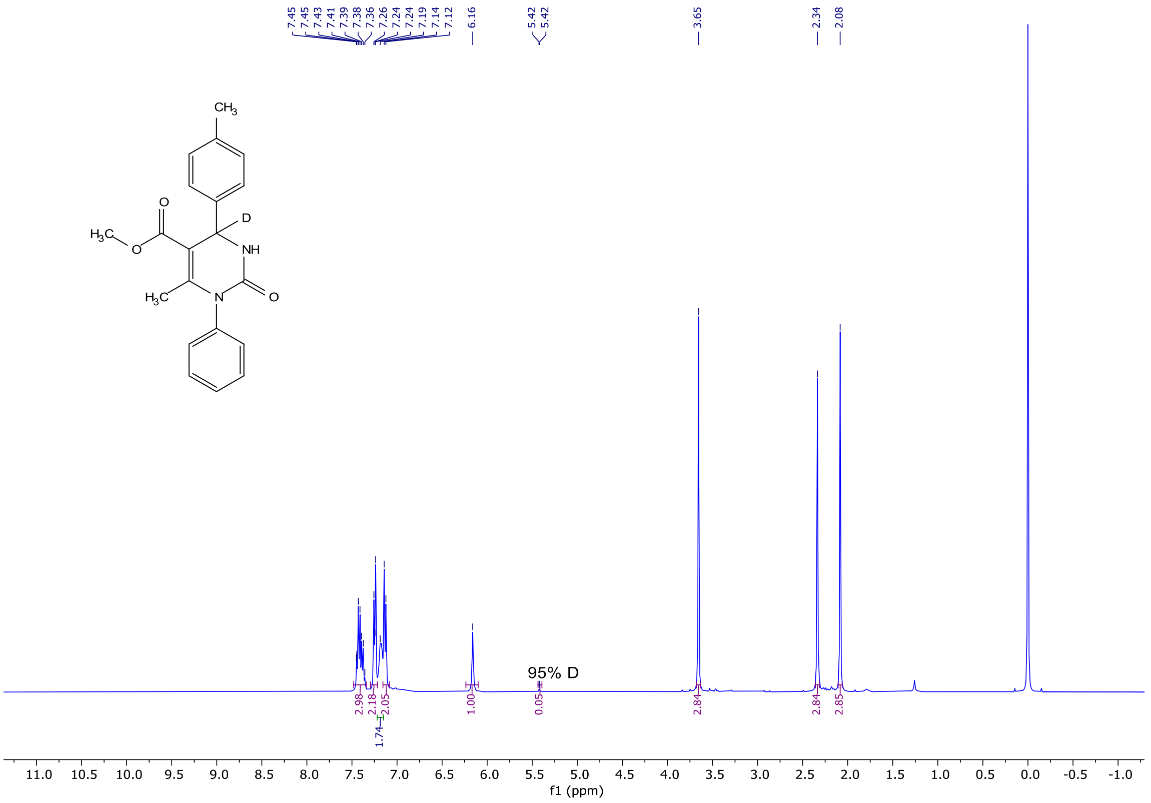

Figure S69. ^{1}H NMR (400 MHz, $CDCl_3$) of **6c**.

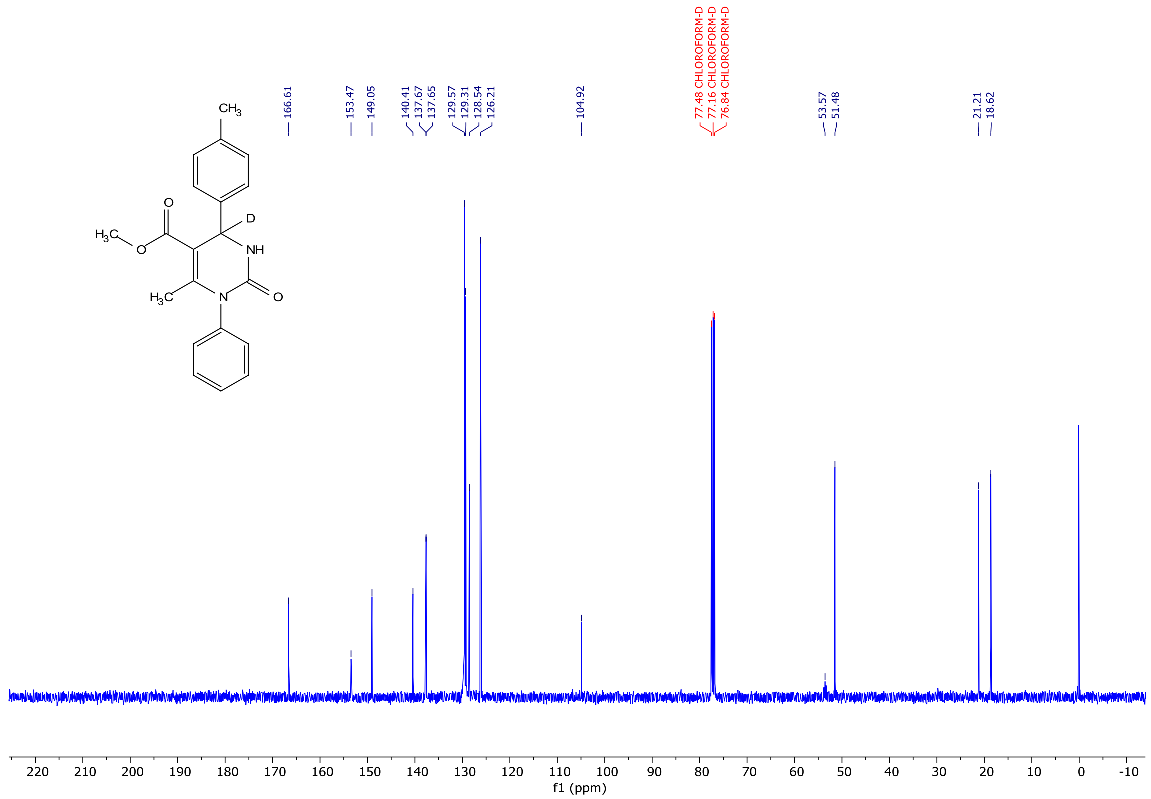

Figure S70. ^{13}C NMR (101 MHz, $CDCl_3$) of **6c**.

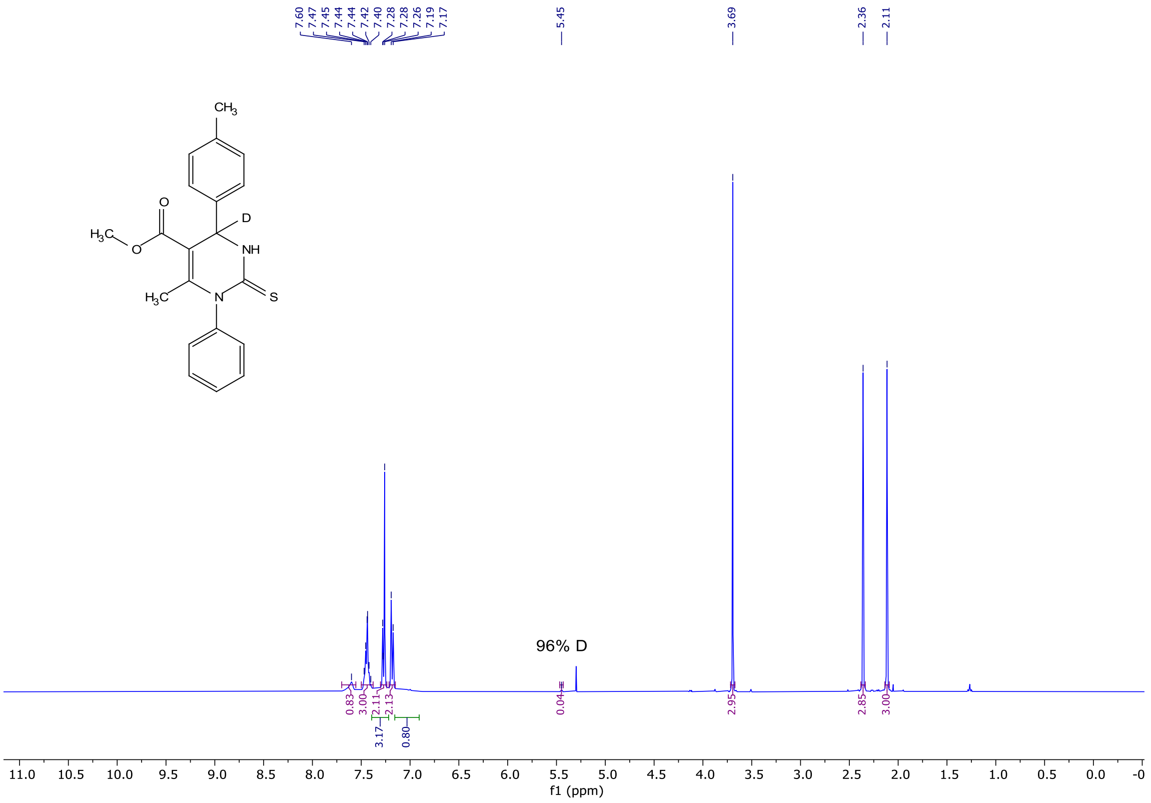

Figure S71. ^{1}H NMR (400 MHz, $CDCl_3$) of **6d**.

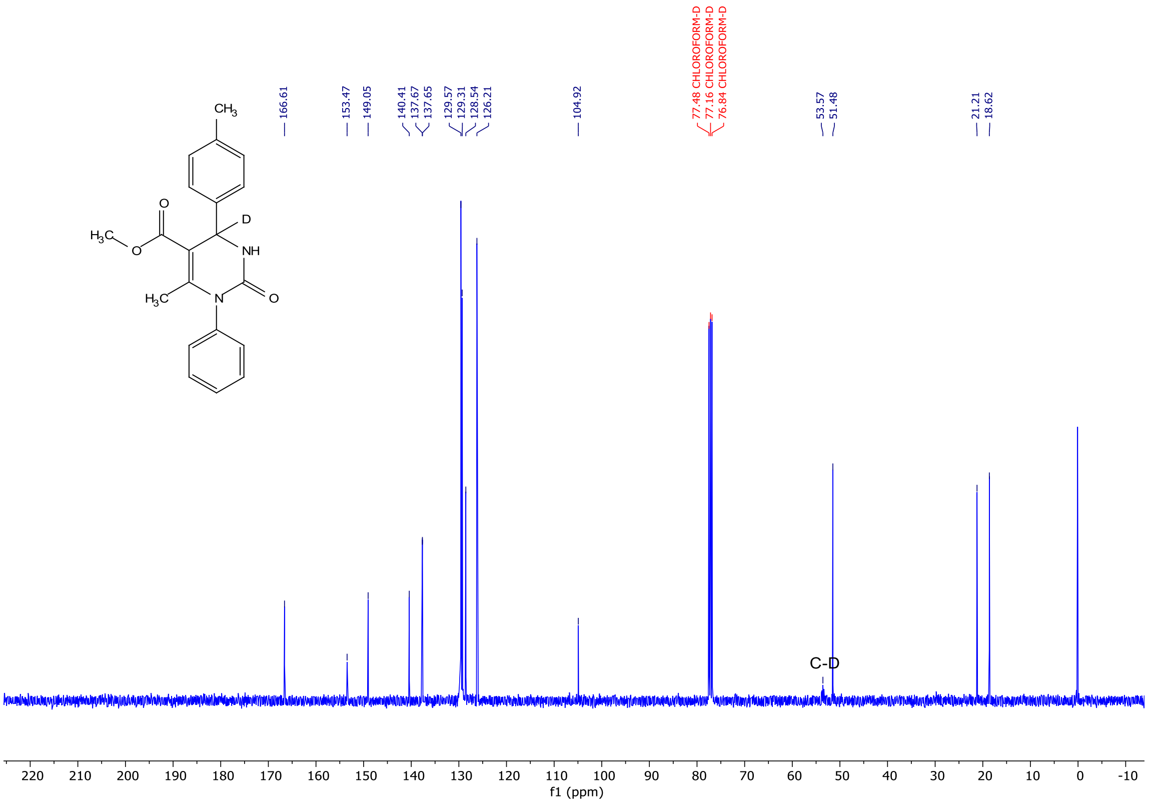

Figure S72. ^{13}C NMR (101 MHz, $CDCl_3$) of **6d**.

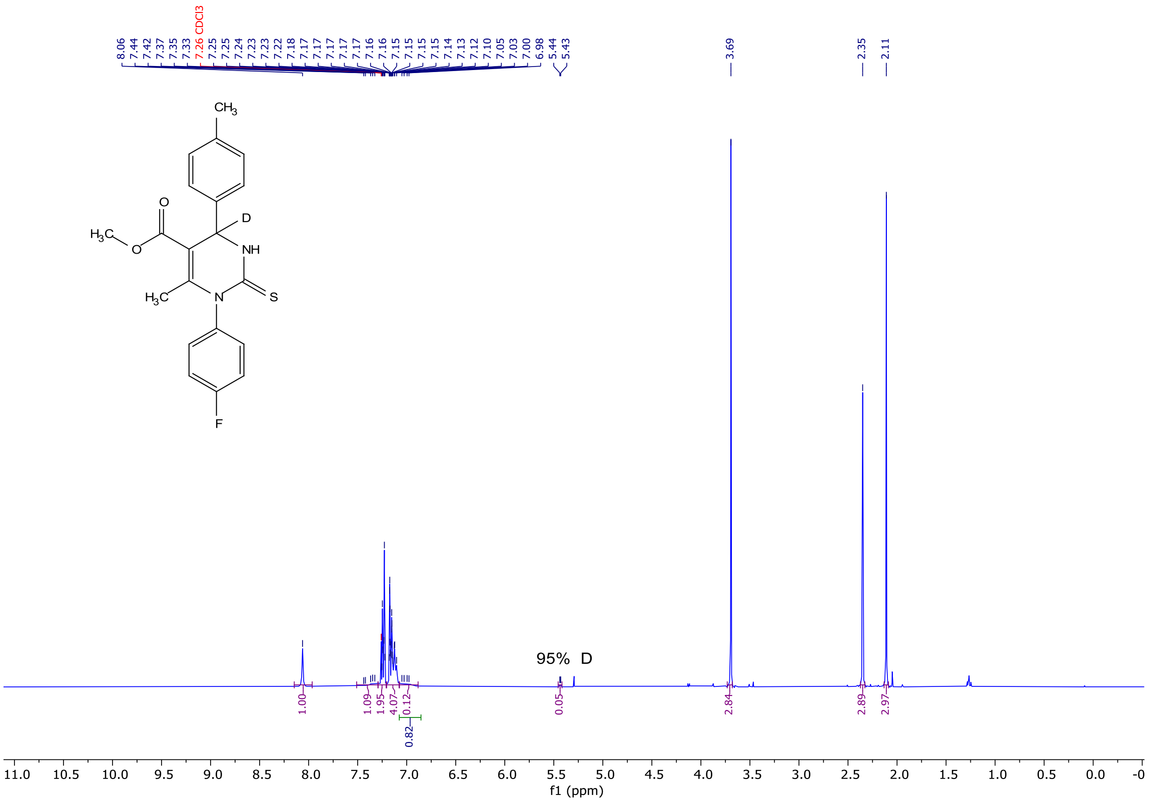

Figure S73. ^{1}H NMR (400 MHz, $CDCl_3$) of **6e**.

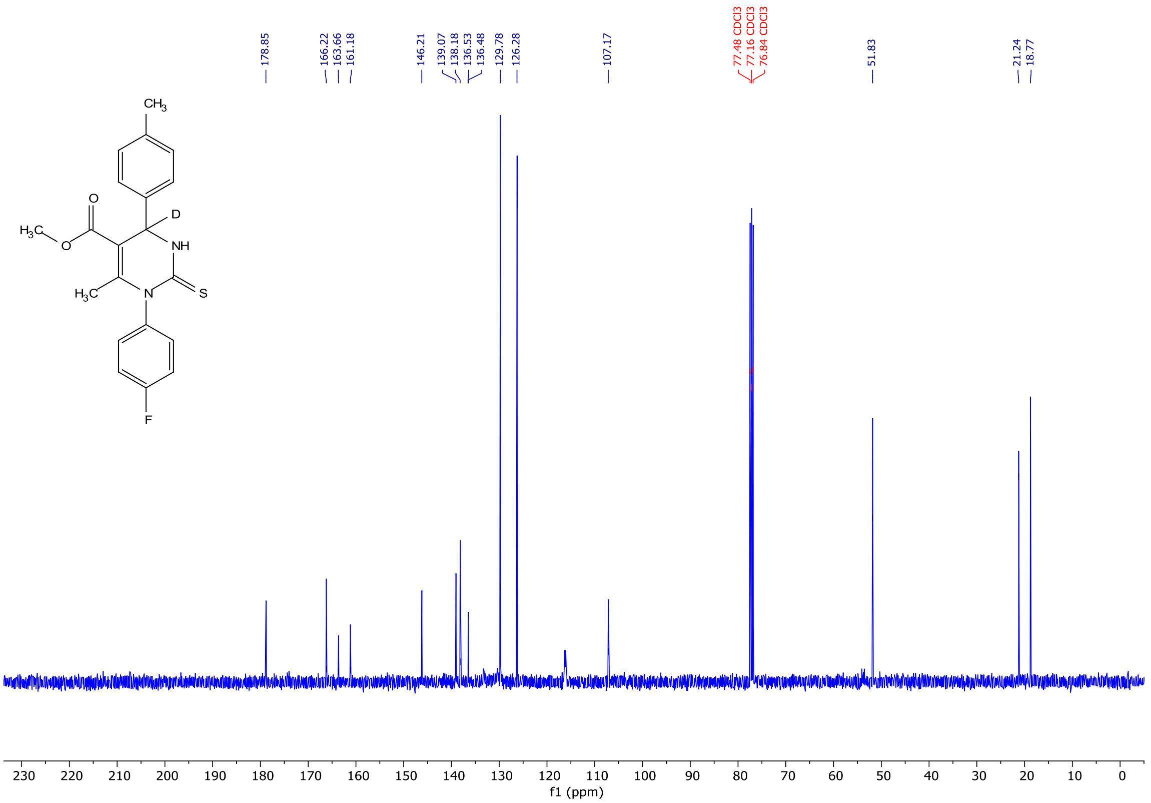

Figure S74. ^{13}C NMR (101 MHz, $CDCl_3$) of **6e**.

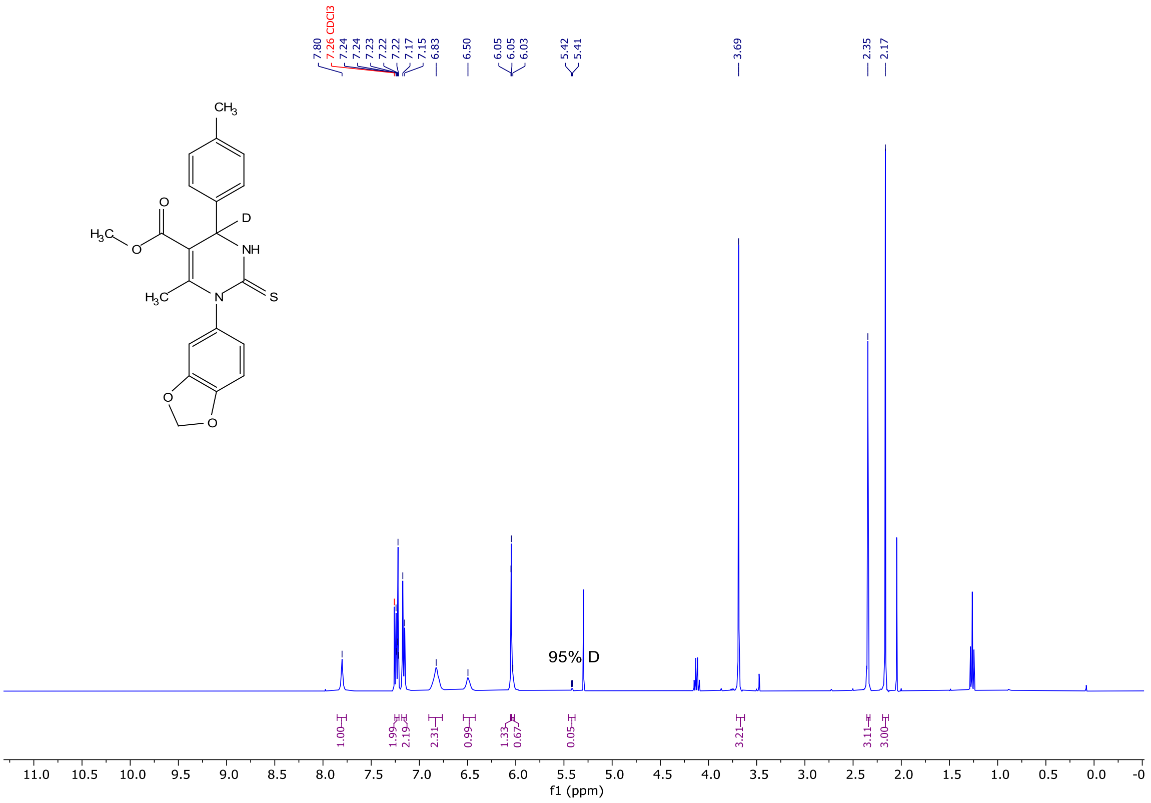

Figure S75. ^{1}H NMR (400 MHz, $CDCl_3$) of **6f**.

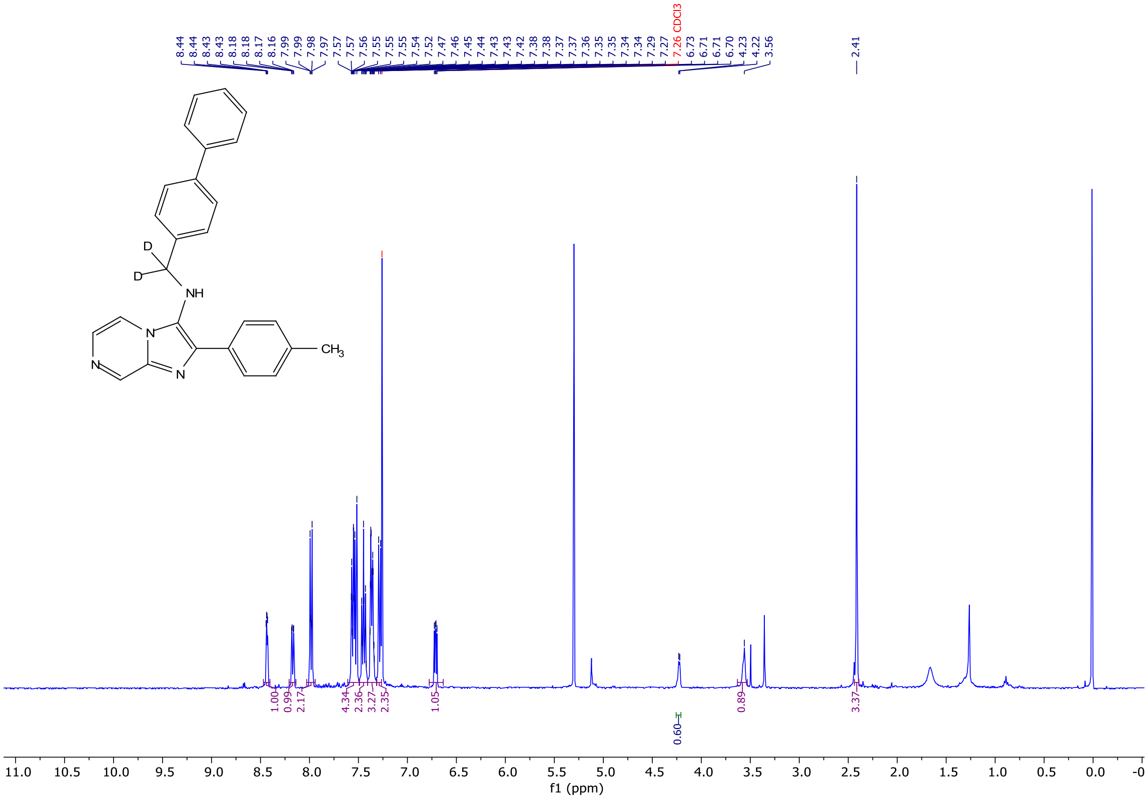

Figure S76. ^{1}H NMR (400 MHz, $CDCl_3$) of **7a**.

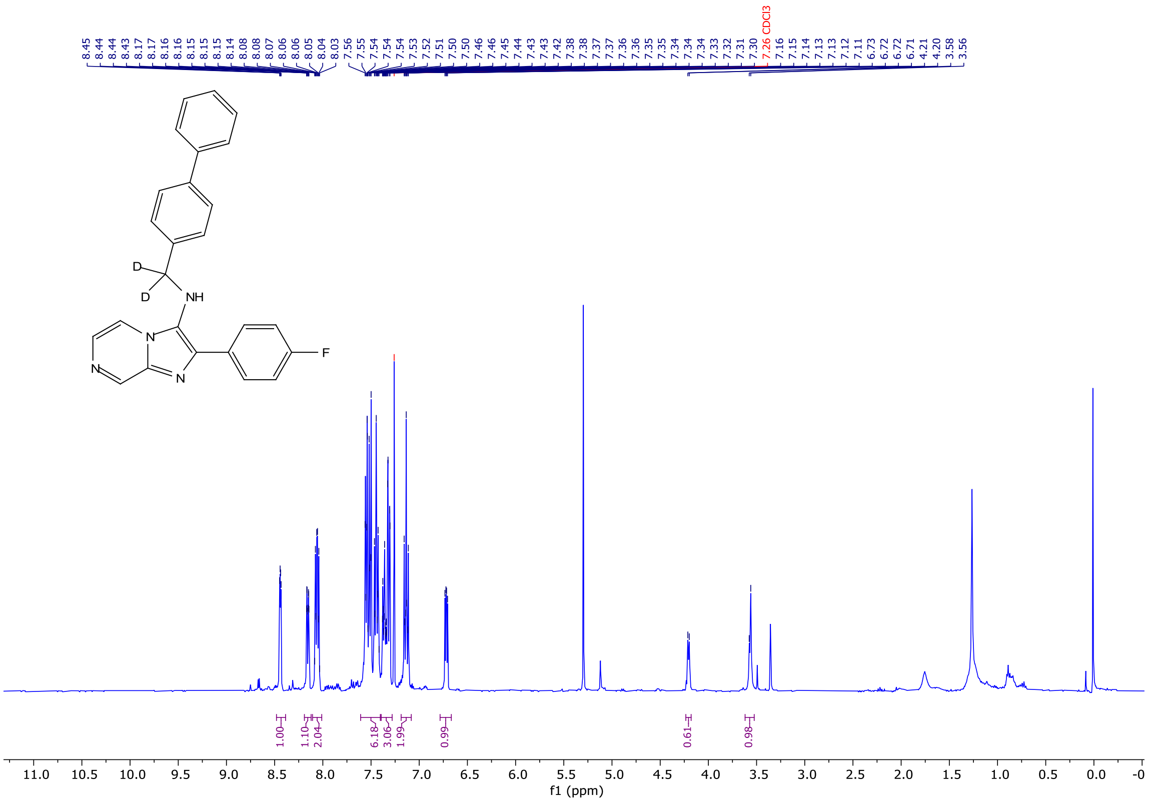

Figure S77. **[1]**H NMR (400 MHz, $CDCl_3$) of **7b**.

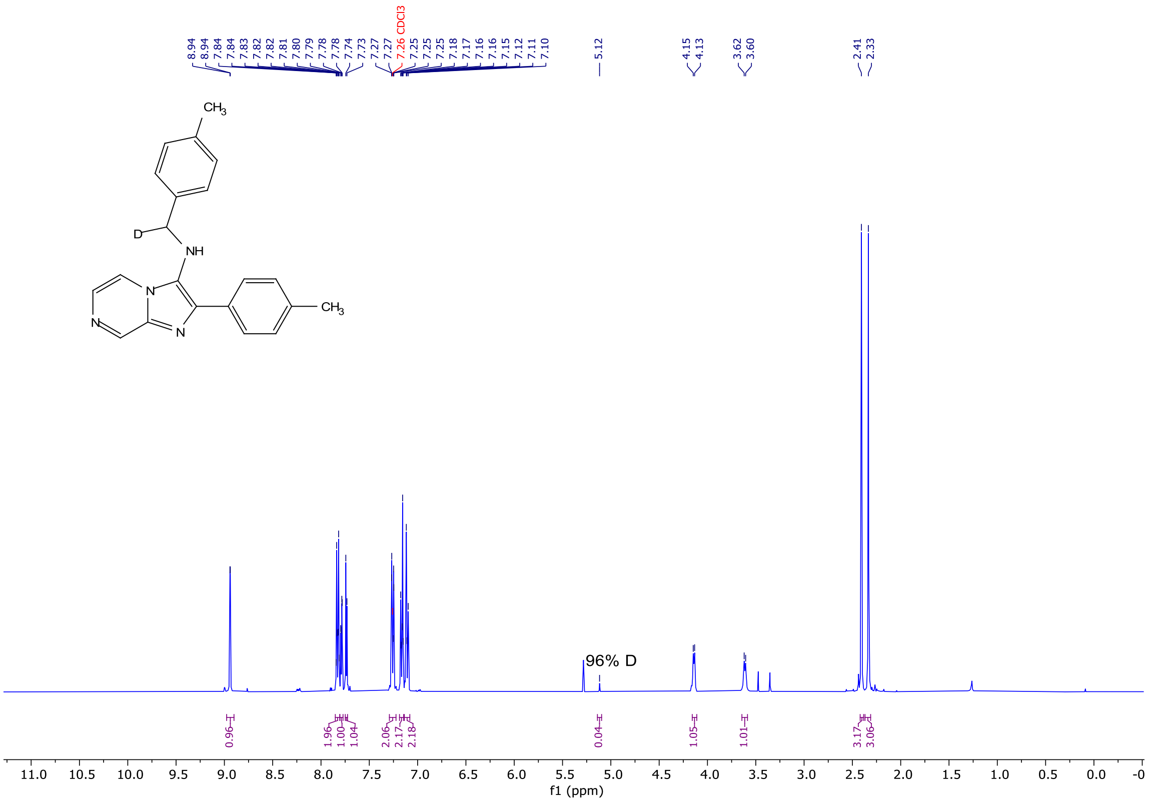

Figure S78. ^{1}H NMR (400 MHz, $CDCl_3$) of **7c**.

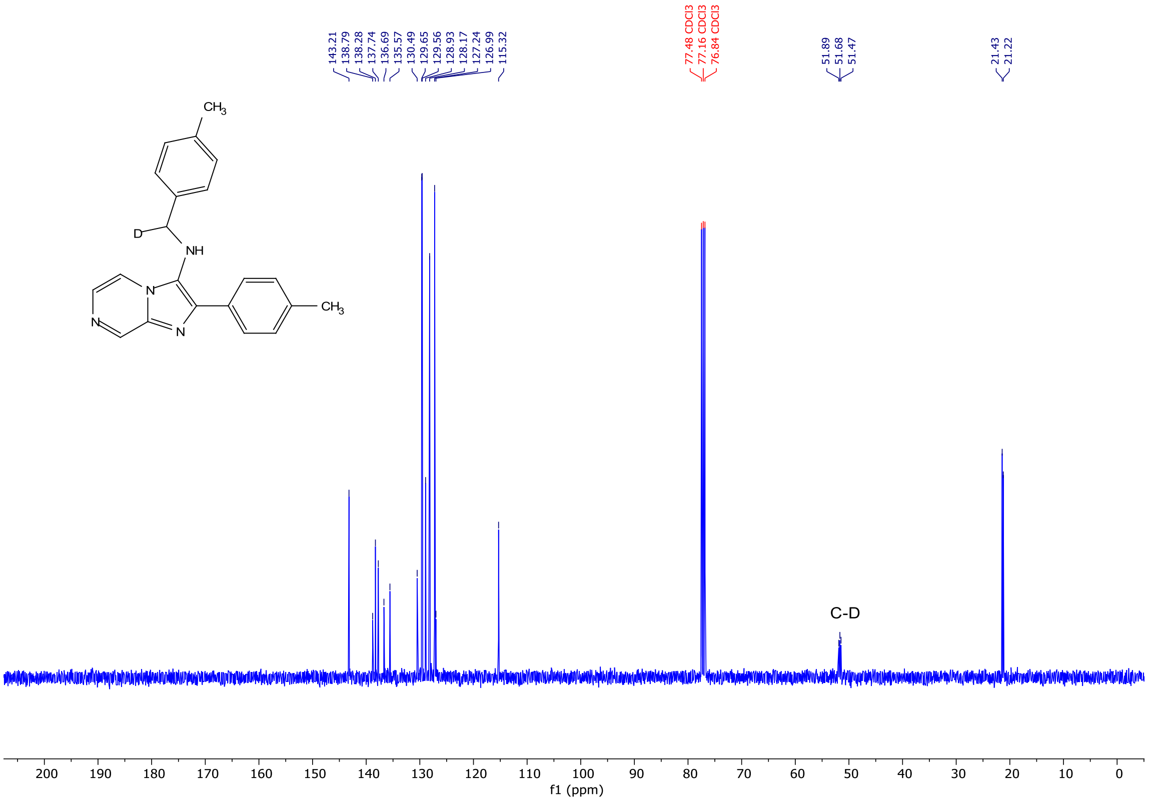

Figure S79. ^{13}C NMR (101 MHz, $CDCl_3$) of **7c**.

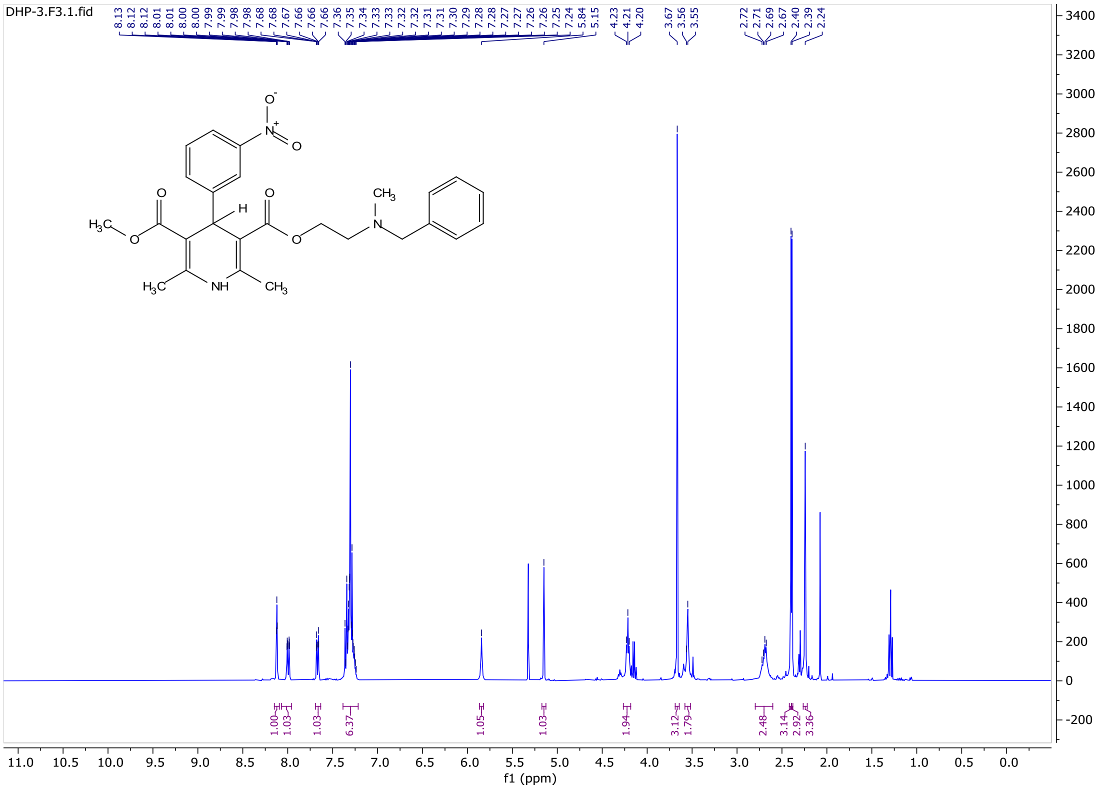

Figure S80. ^{1}H NMR (400 MHz, $CDCl_3$) of **8a**.

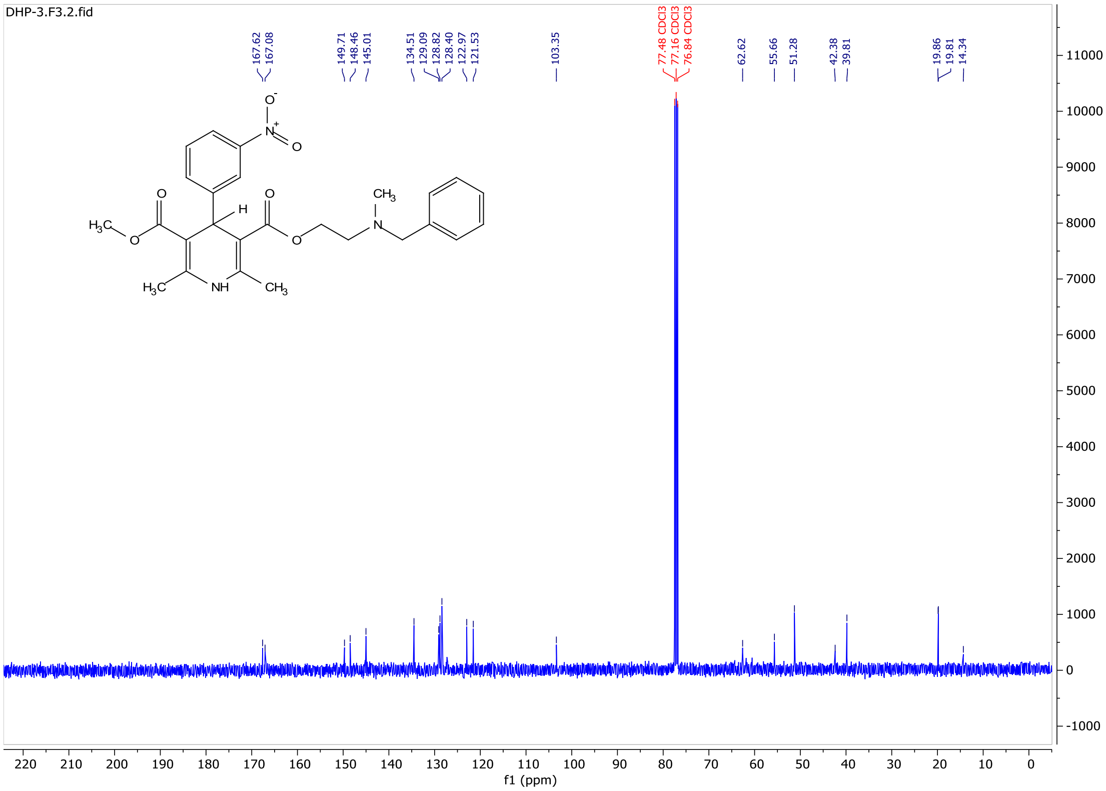

Figure S81. ^{13}C NMR (101 MHz, $CDCl_3$) of **8a**.

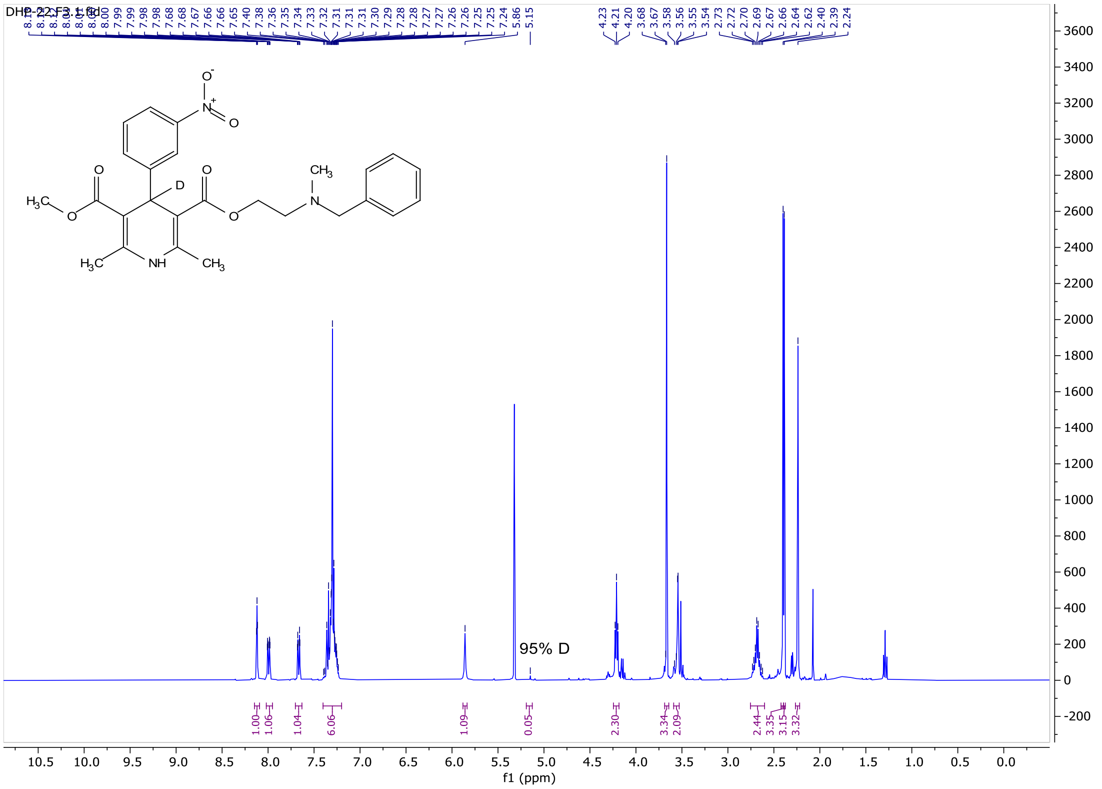

Figure S82. ^{1}H NMR (400 MHz, $CDCl_3$) of **8b**.

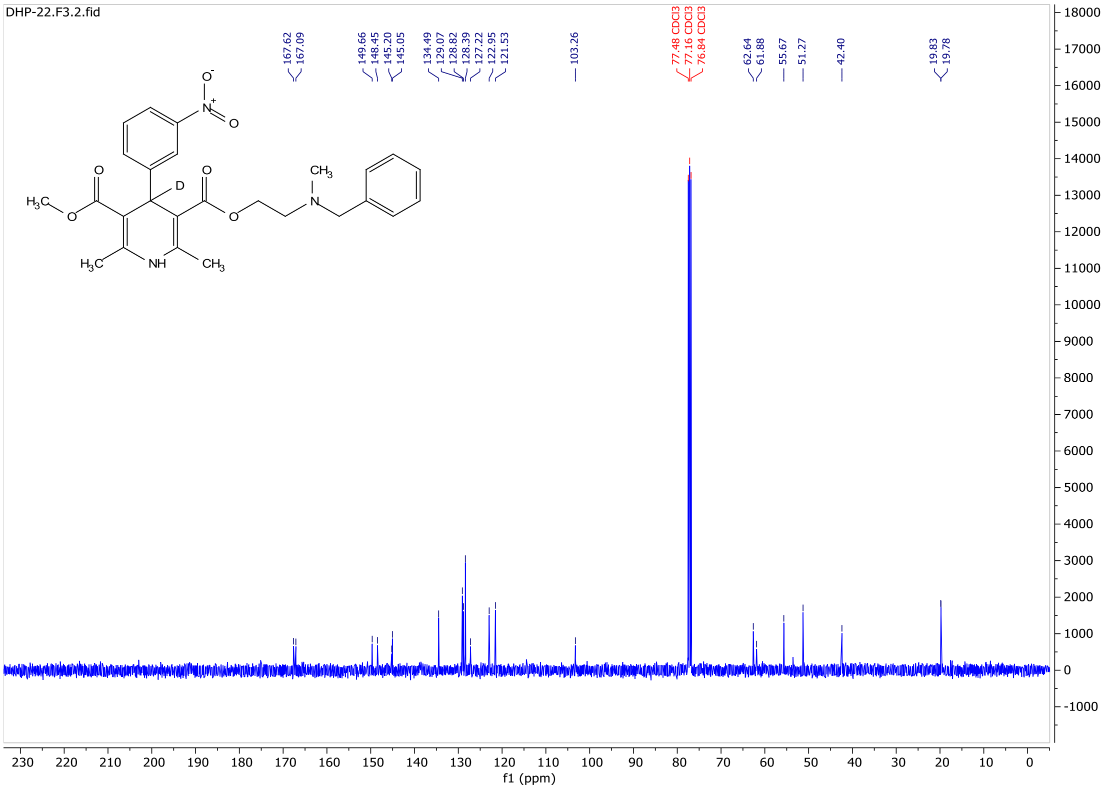

Figure S83. ^{13}C NMR (101 MHz, $CDCl_3$) of **8b**.

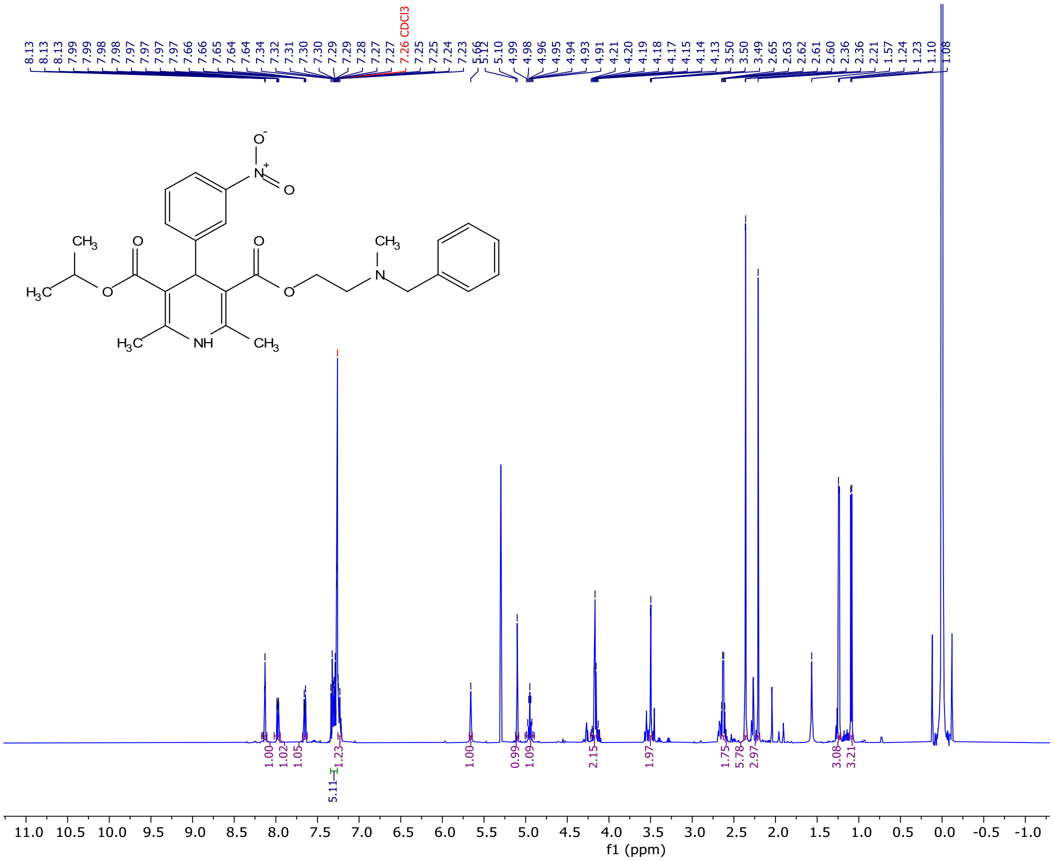

Figure S84. ^{1}H NMR (400 MHz, $CDCl_3$) of **8c**.

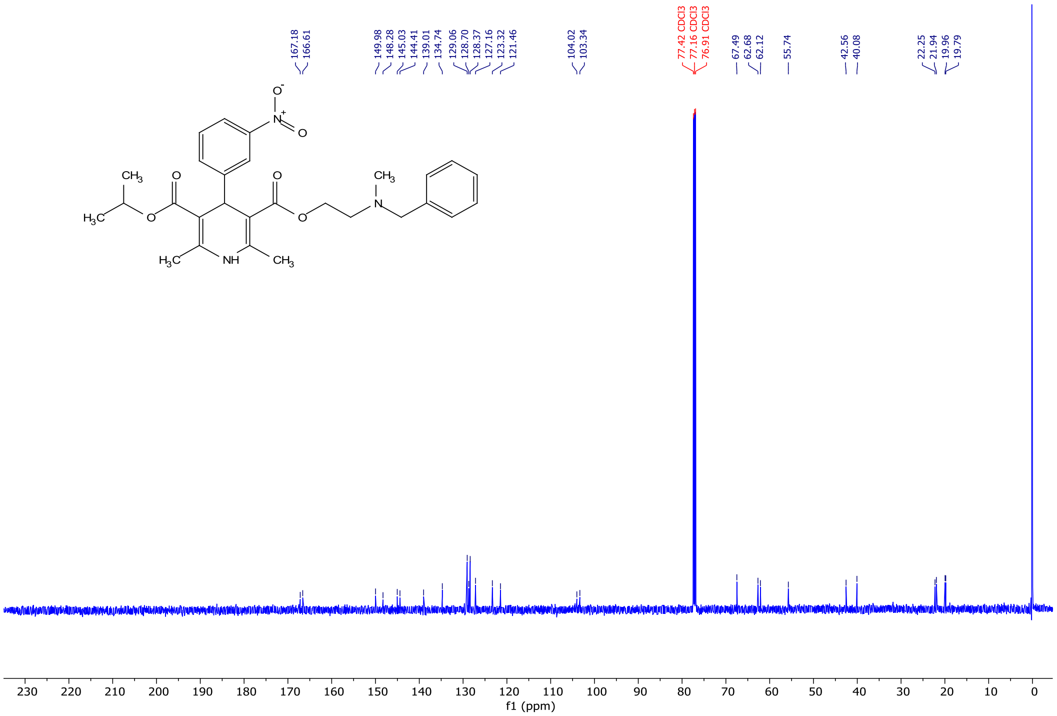

Figure S85. ^{13}C NMR (101 MHz, $CDCl_3$) of **8c**.

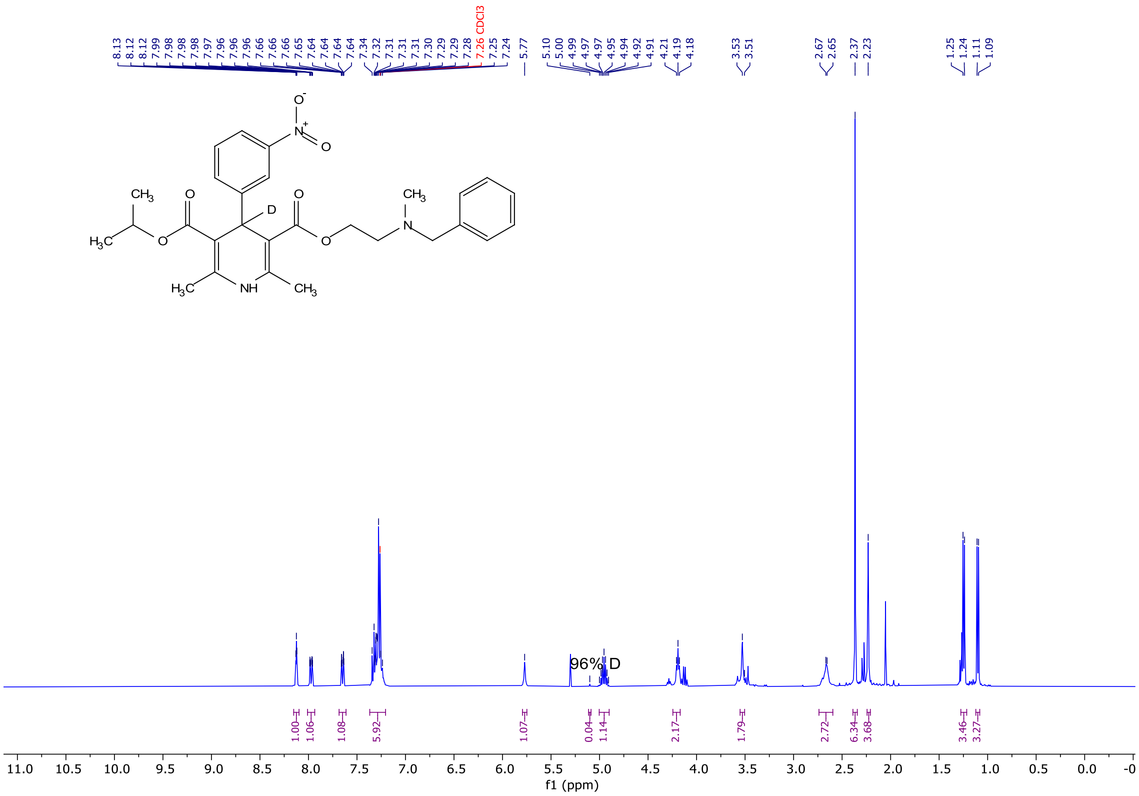

Figure S86. ^{1}H NMR (400 MHz, $CDCl_3$) of **8d**.

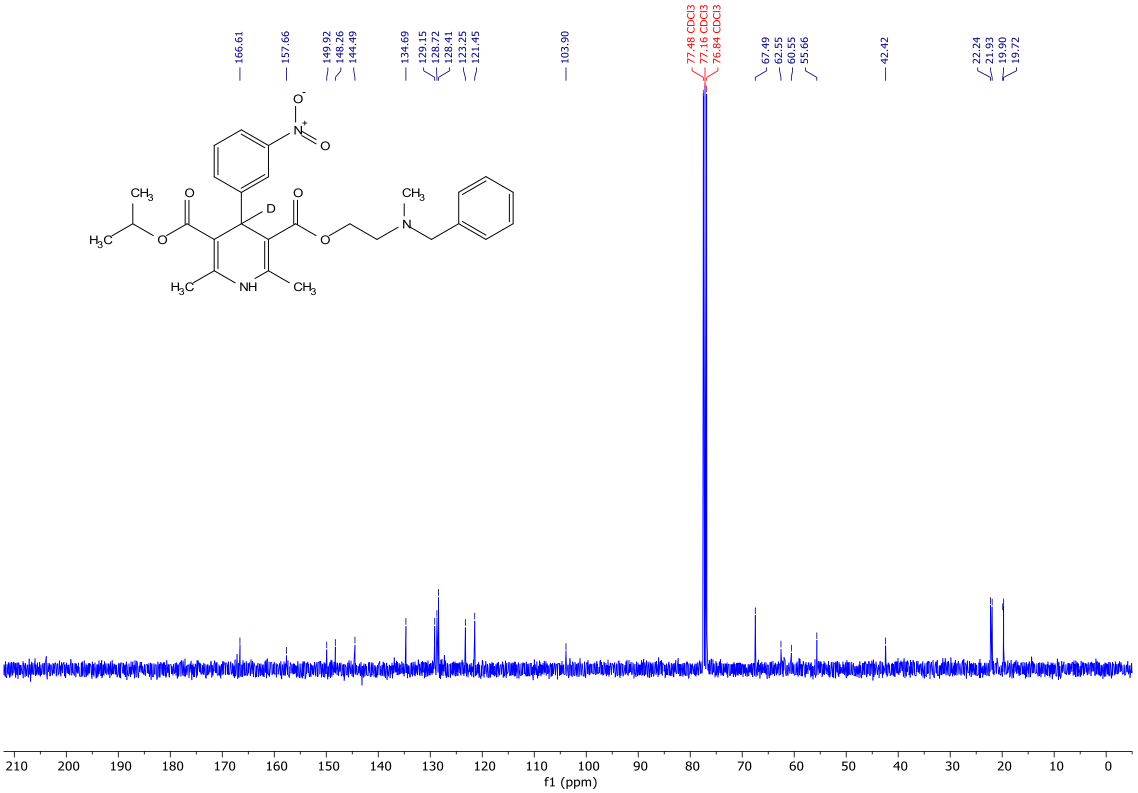

Figure S87. ^{13}C NMR (101 MHz, $CDCl_3$) of **8d**.

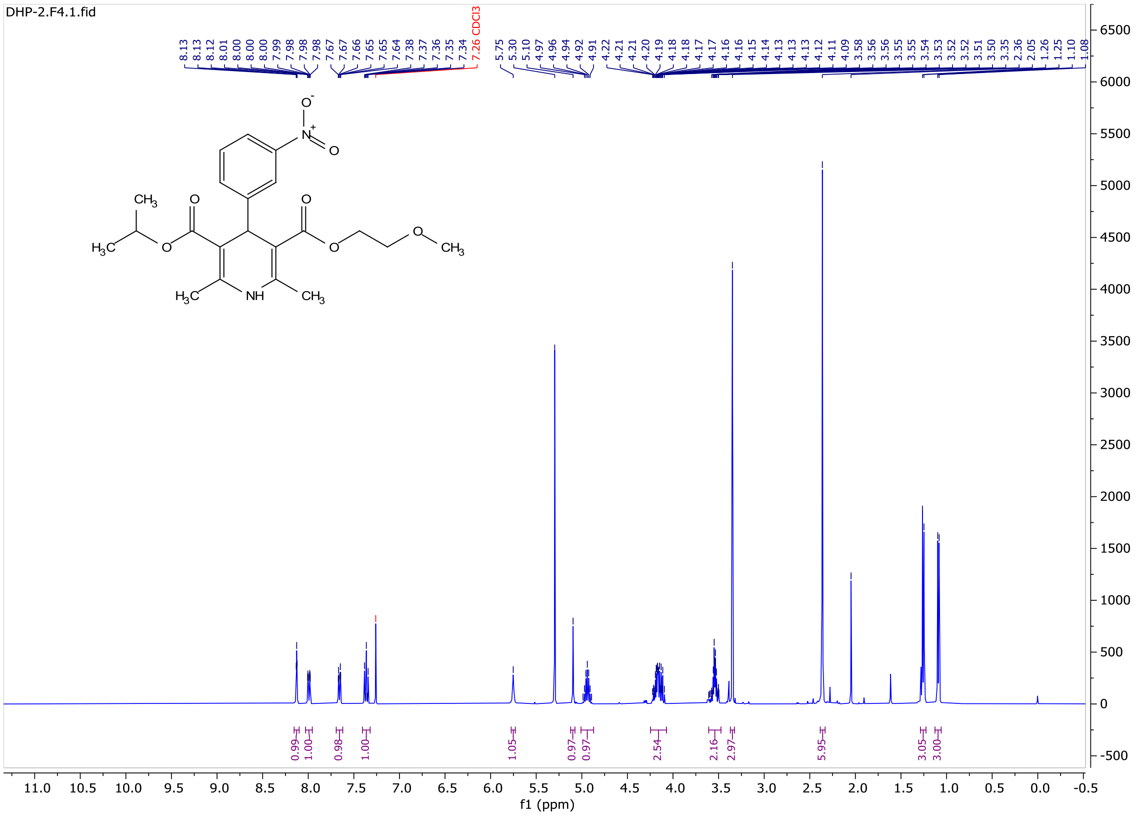

Figure S88. ^{1}H NMR (400 MHz, $CDCl_3$) of **8e**.

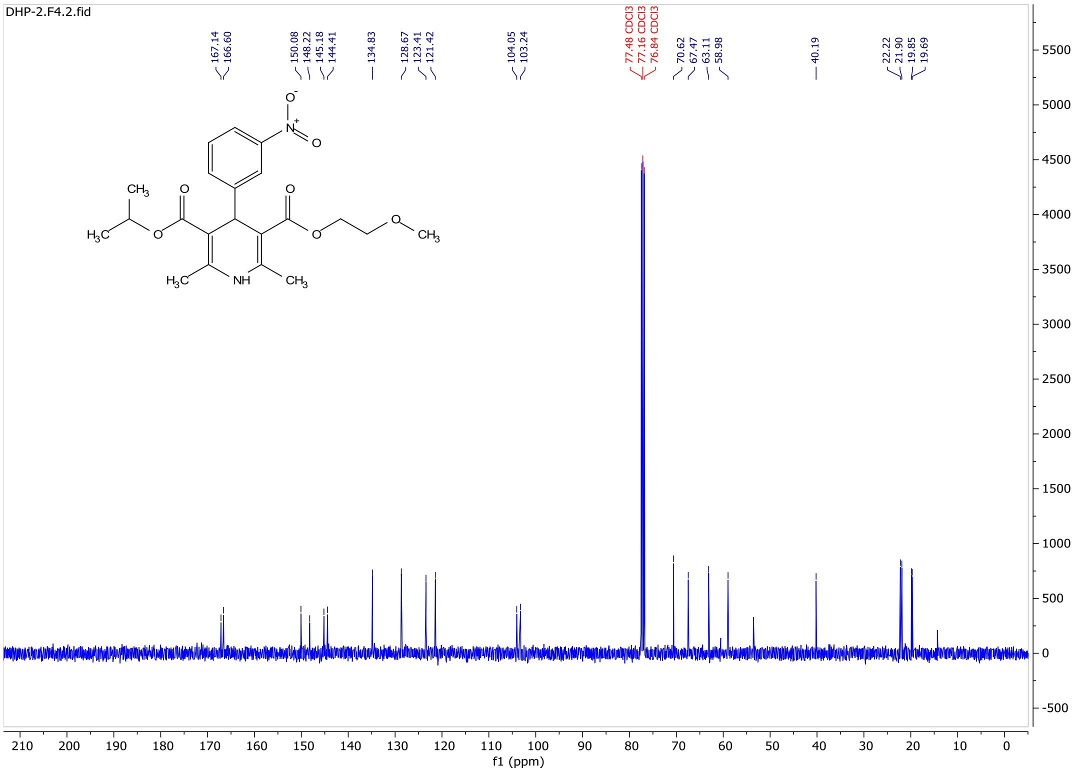

Figure S88. ^{13}C NMR (101 MHz, $CDCl_3$) of **8e**.

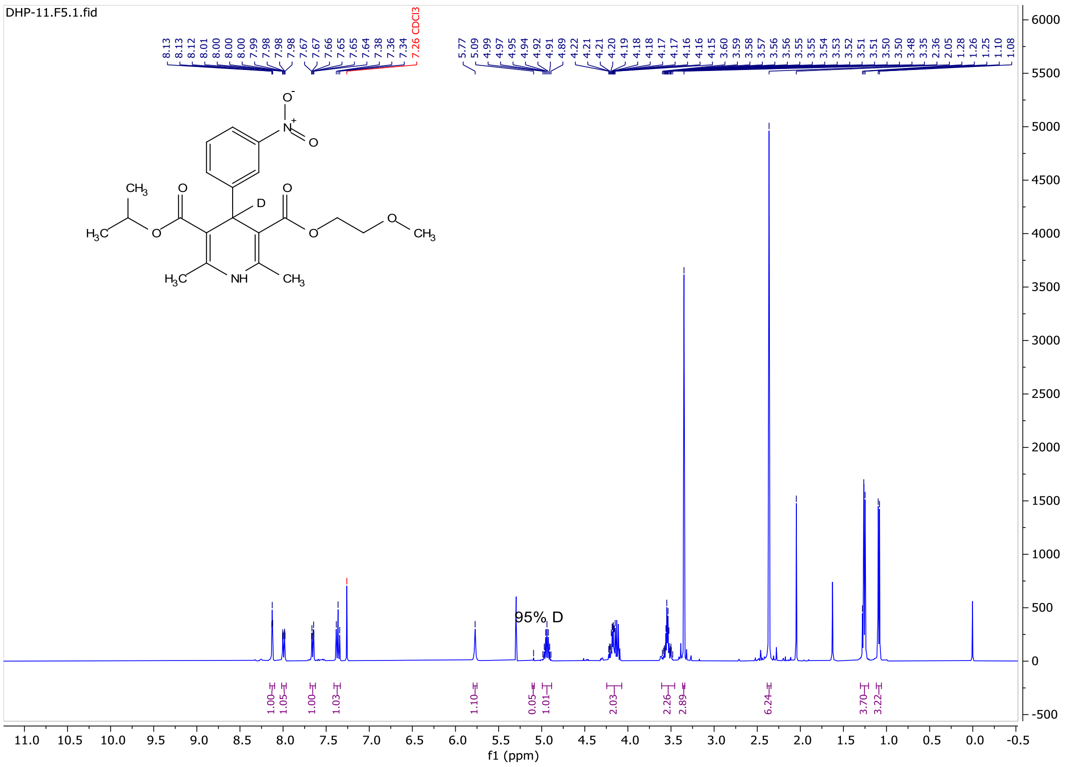

Figure S89. ^{1}H NMR (400 MHz, $CDCl_3$) of **8f**.

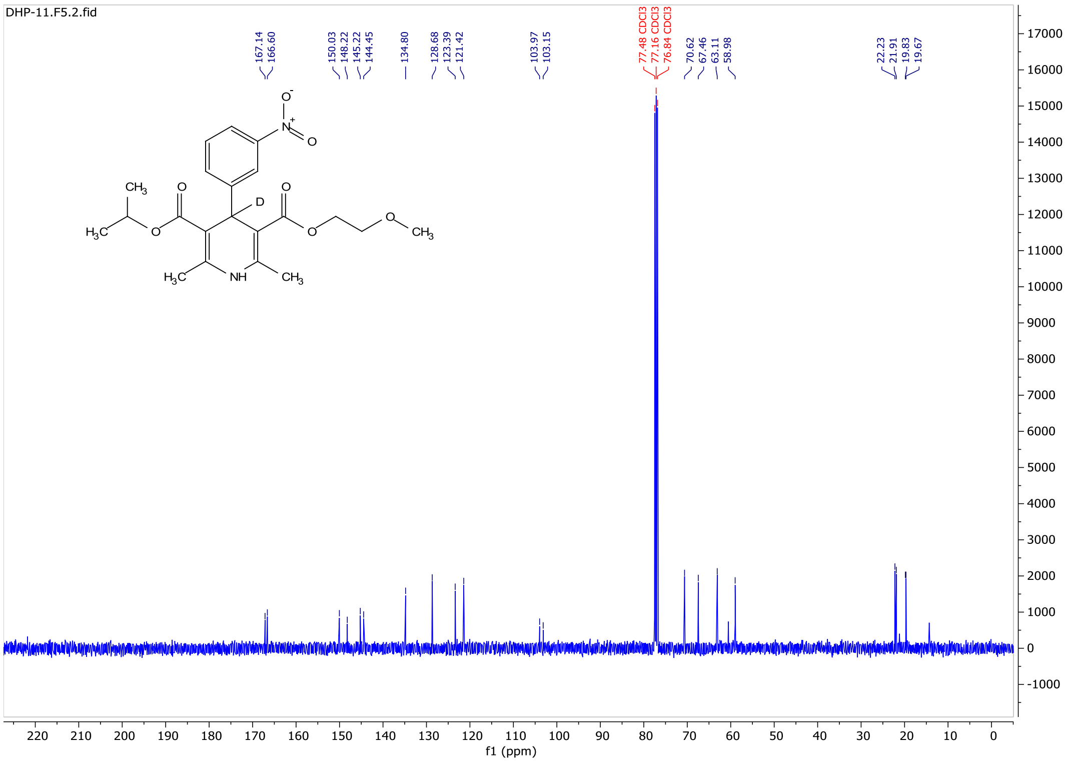

Figure S90. ^{13}C NMR (101 MHz, $CDCl_3$) of **8f**.

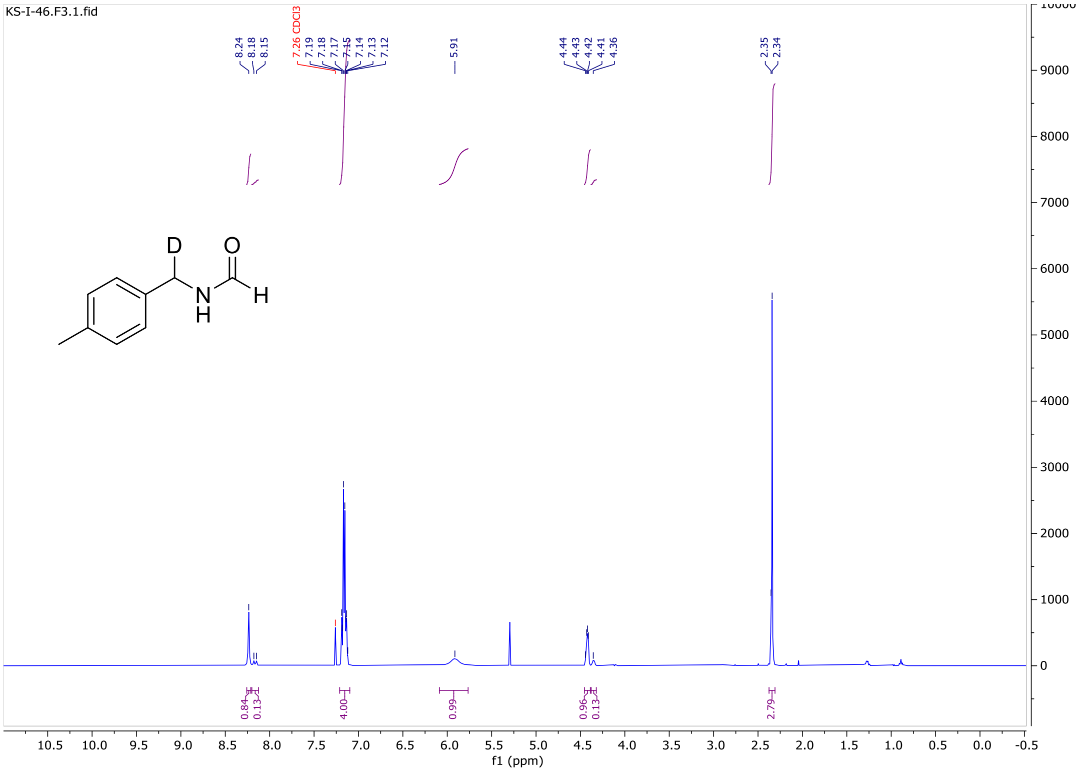

Figure S91. ^{1}H NMR (400 MHz, $CDCl_3$) of **9ba**.

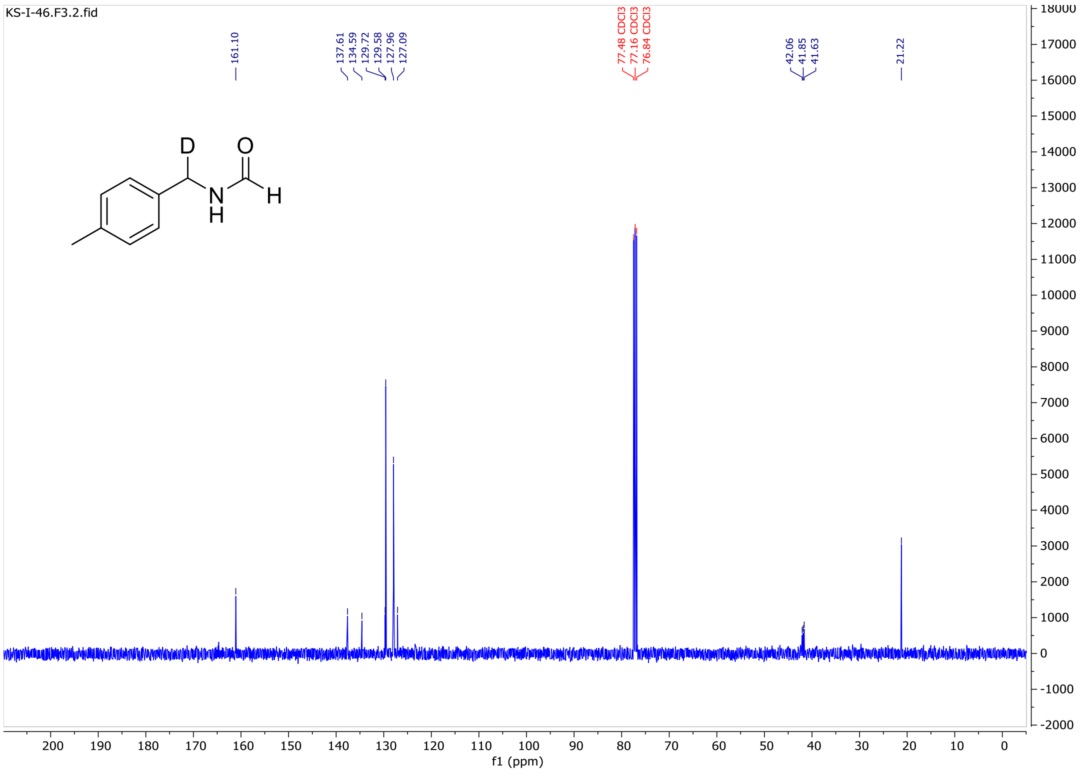

Figure S92. ^{13}C NMR (101 MHz, $CDCl_3$) of **9ba**.

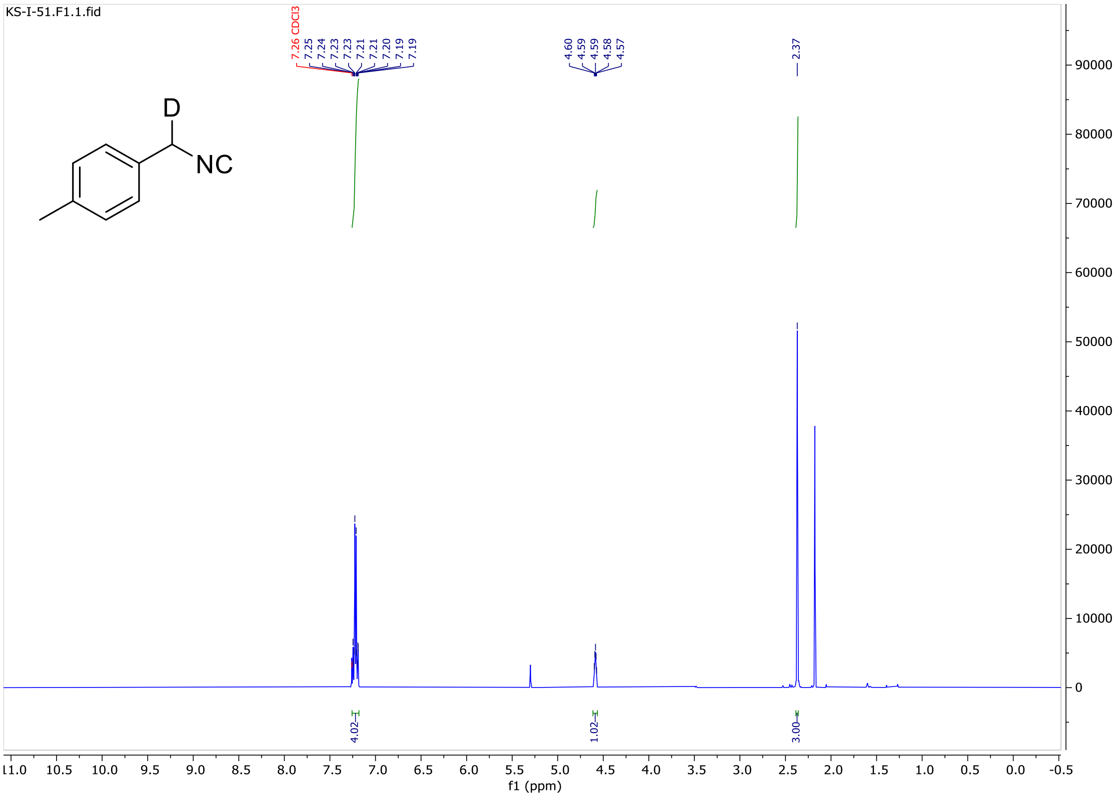

Figure S93. ^{1}H NMR (400 MHz, $CDCl_3$) of **10ba**.

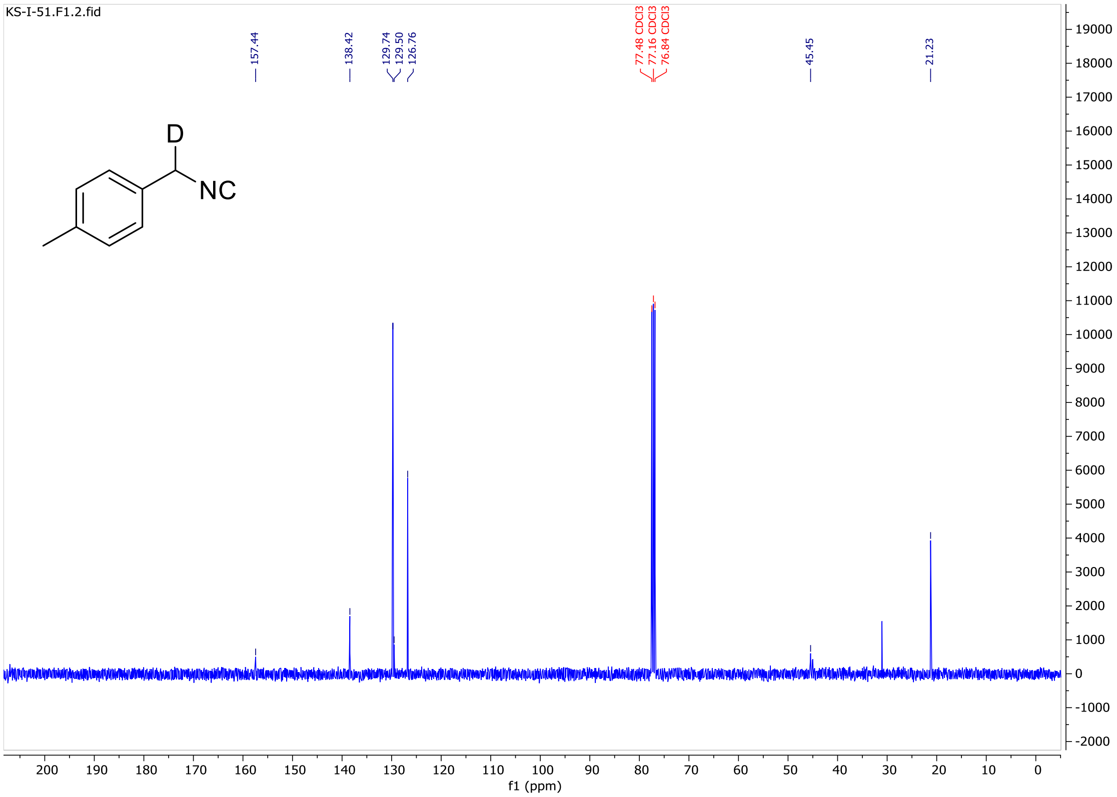

Figure S94. ^{13}C NMR (101 MHz, $CDCl_3$) of **10ba**.

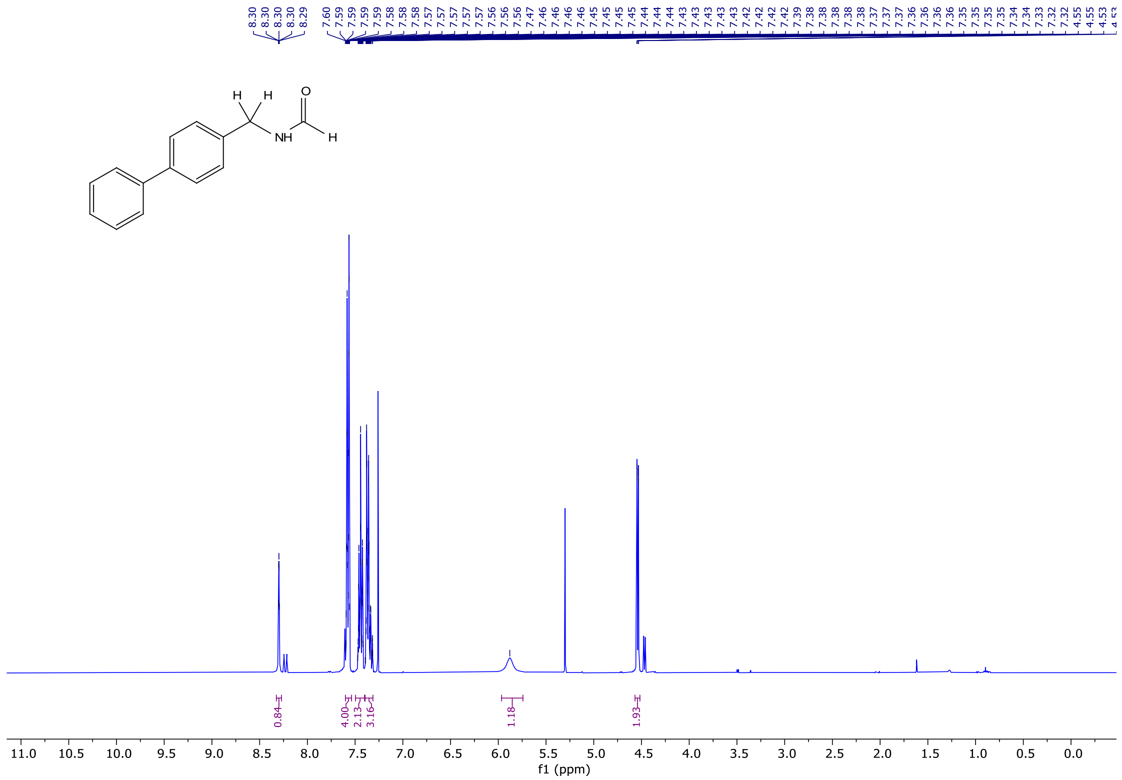

Figure S95. ¹H NMR (400 MHz, $CDCl_3$) of **9aa**.

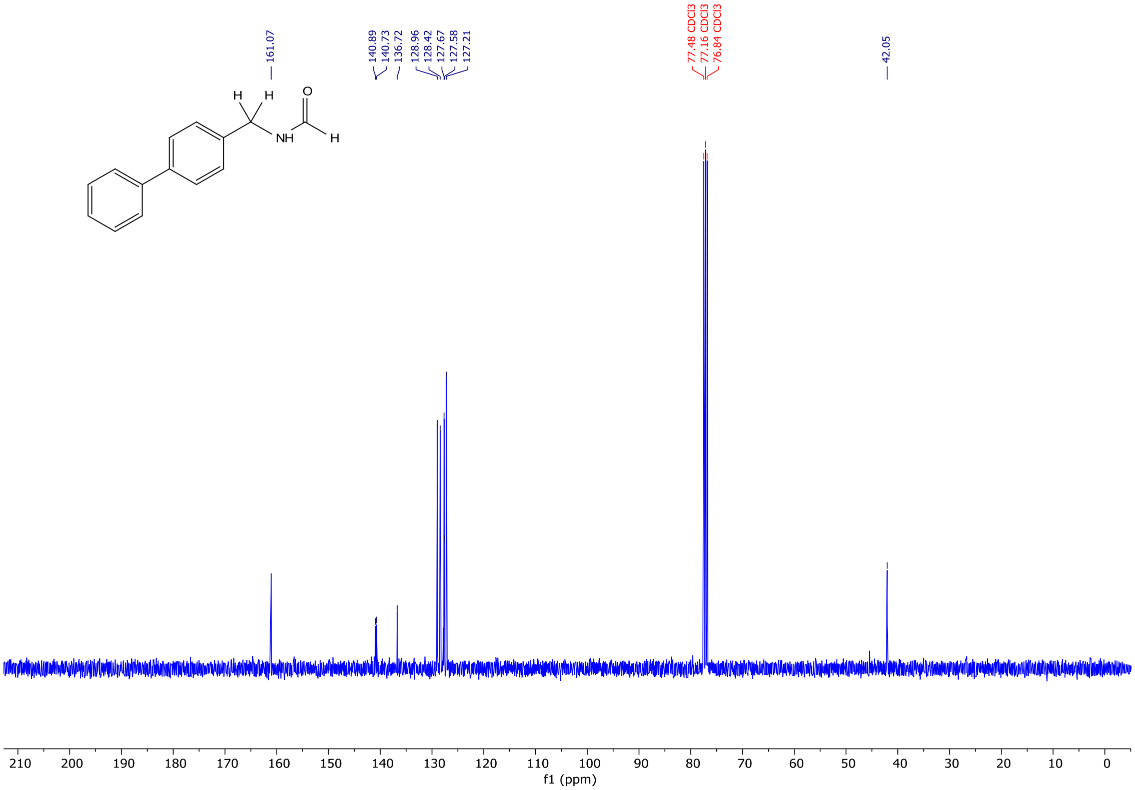

Figure S96. ¹³C NMR (101 MHz, $CDCl_3$) of **9aa**

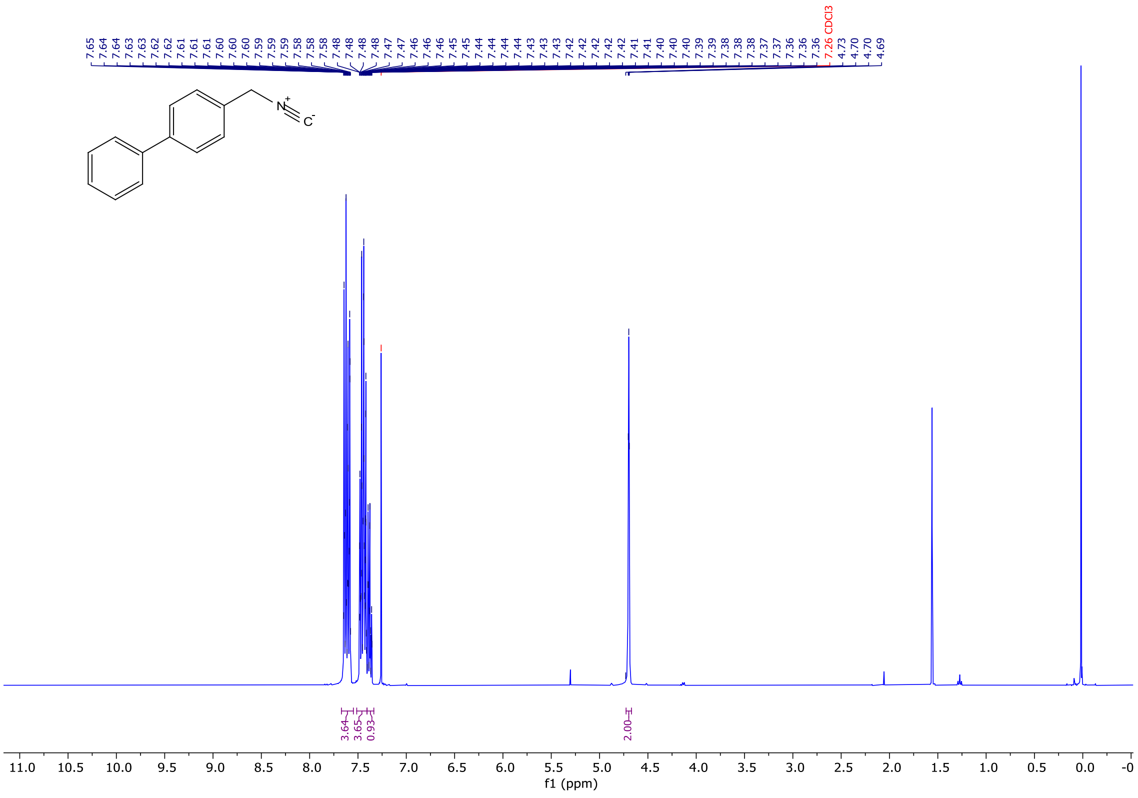

Figure S97. ^{1}H NMR (400 MHz, $CDCl_3$) of **10aa**.

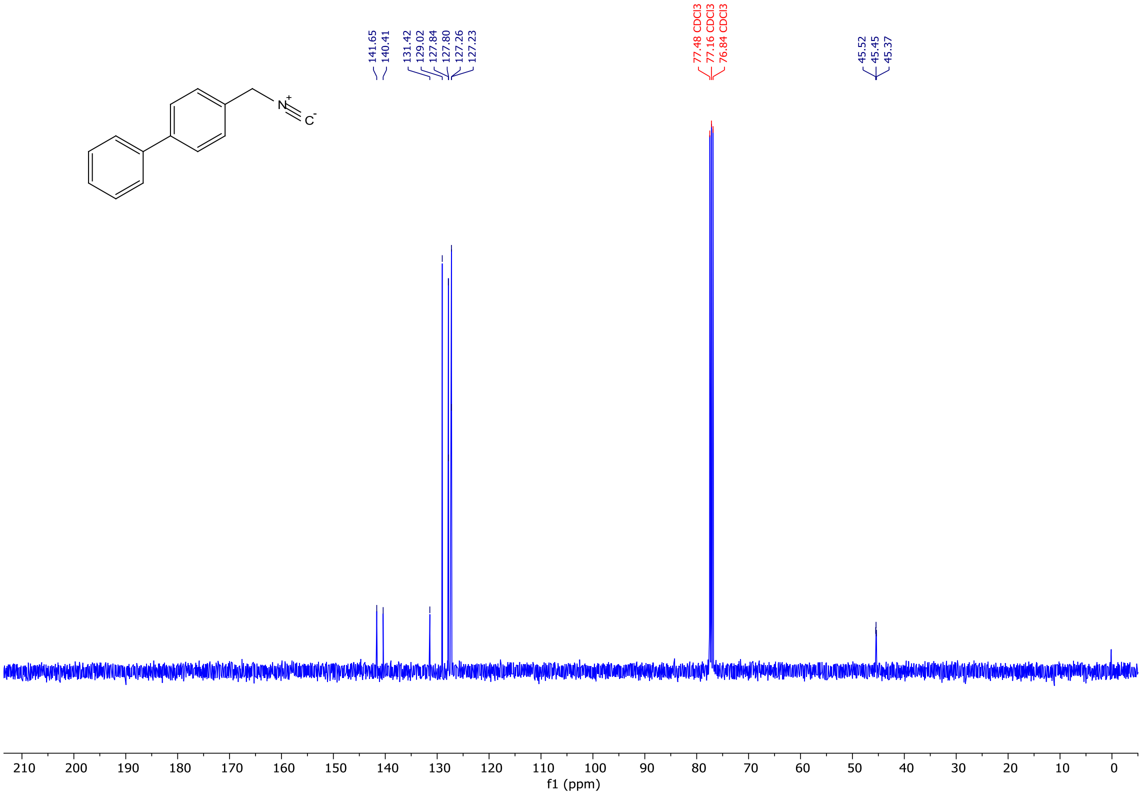

Figure S98. ^{13}C NMR (101 MHz, $CDCl_3$) of **10aa**.

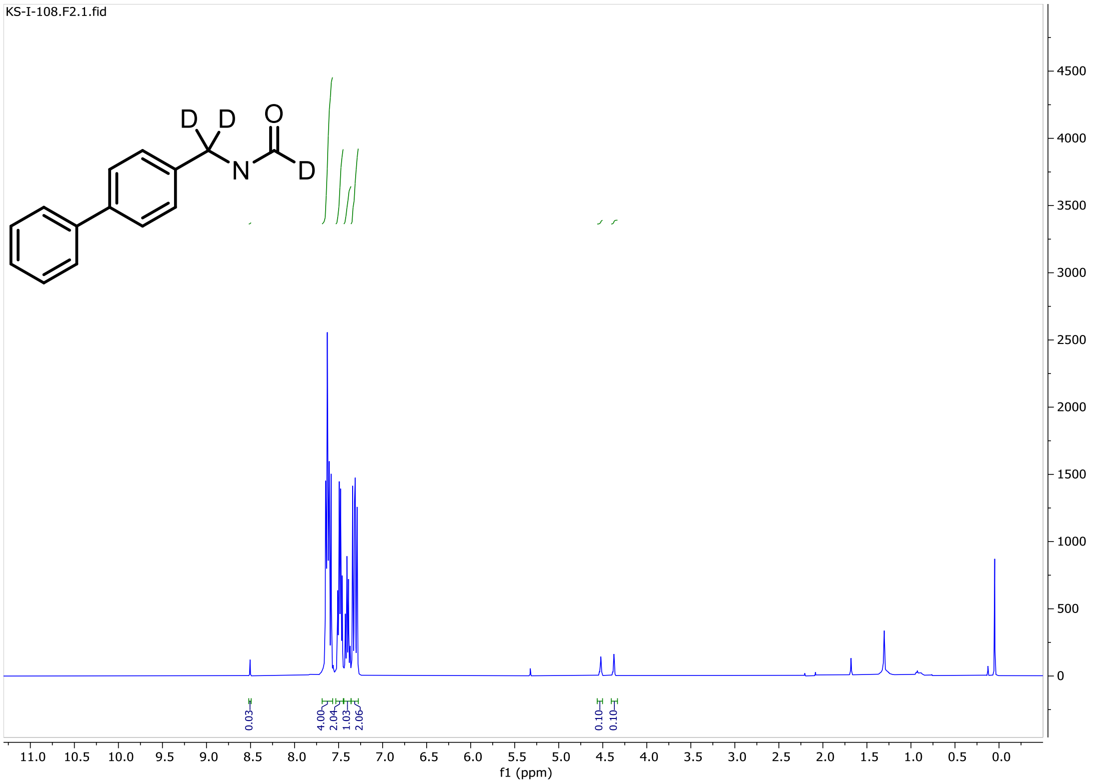

Figure S99. ^{1}H NMR (400 MHz, $CDCl_3$) of **9ca**.

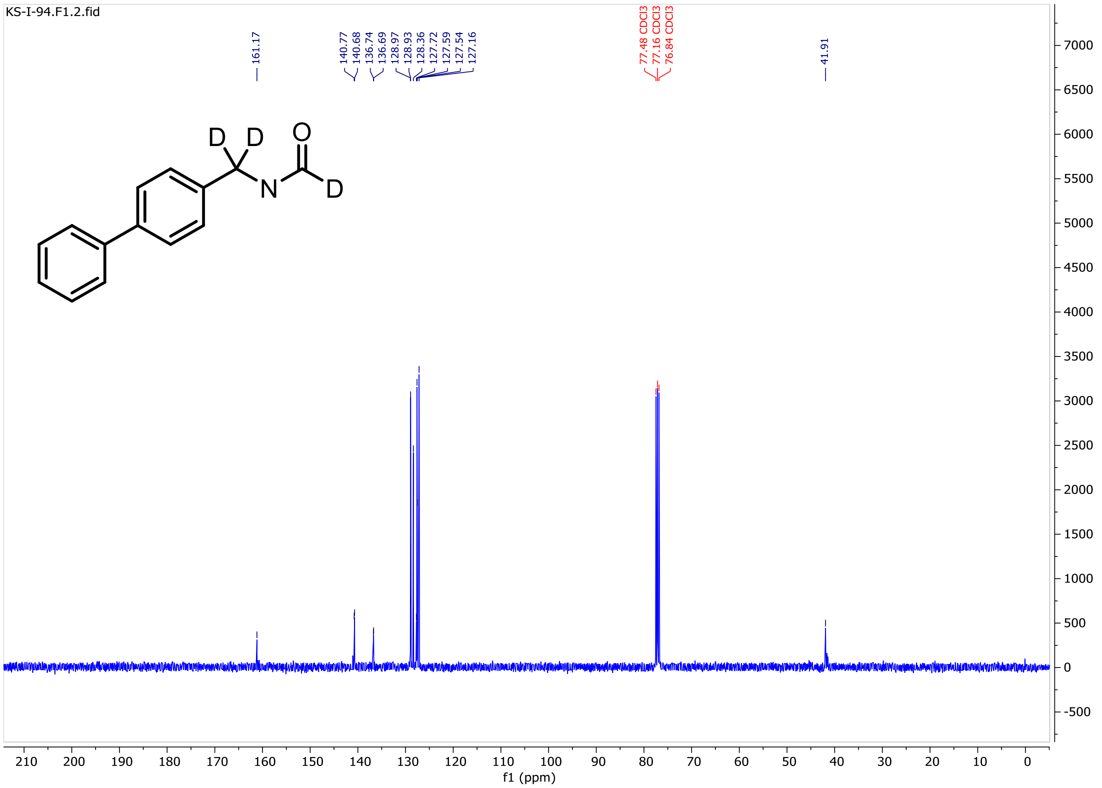

Figure S100. ^{13}C NMR (101 MHz, $CDCl_3$) of **9ca**.

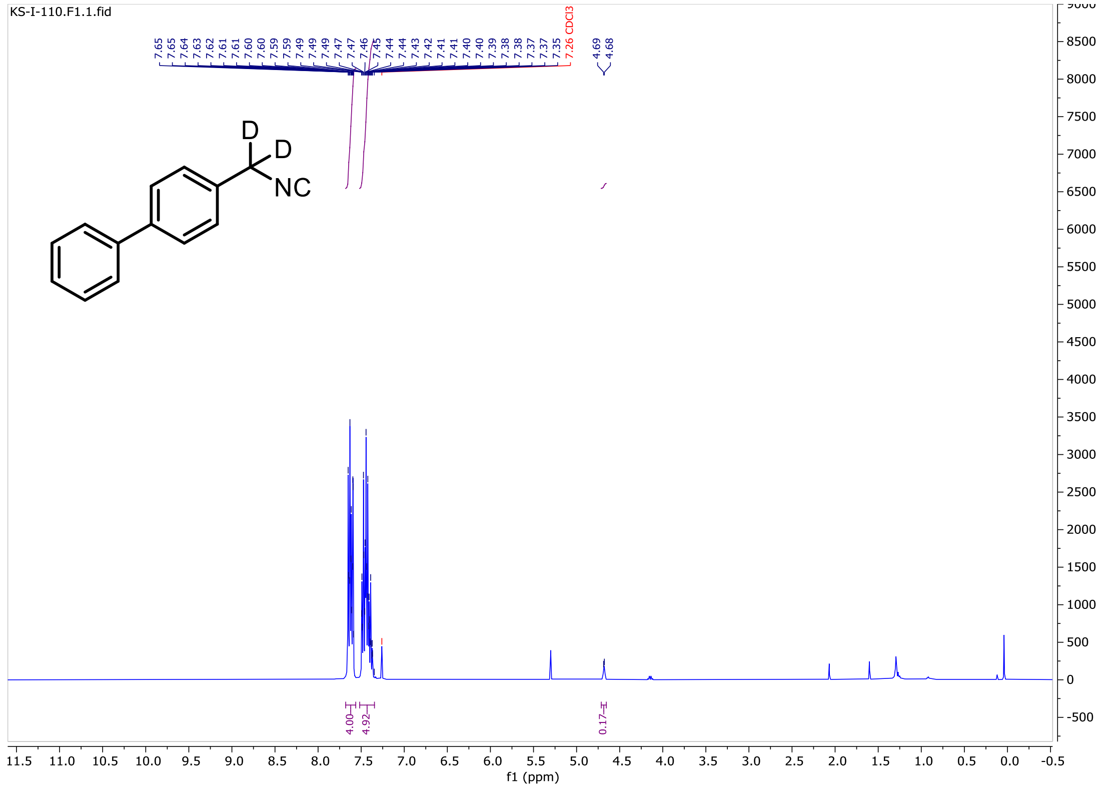

Figure S101. ^{1}H NMR (400 MHz, $CDCl_3$) of **9ca**.

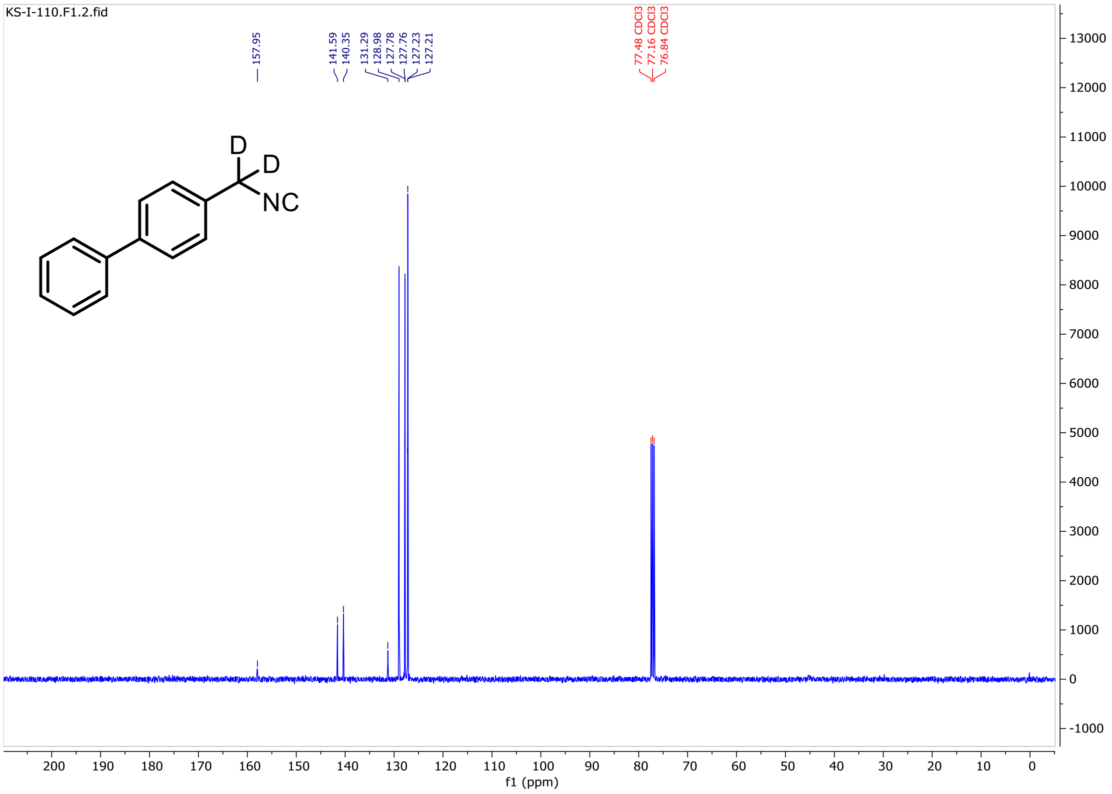

Figure S102. ^{13}C NMR (101 MHz, $CDCl_3$) of **10ca**.

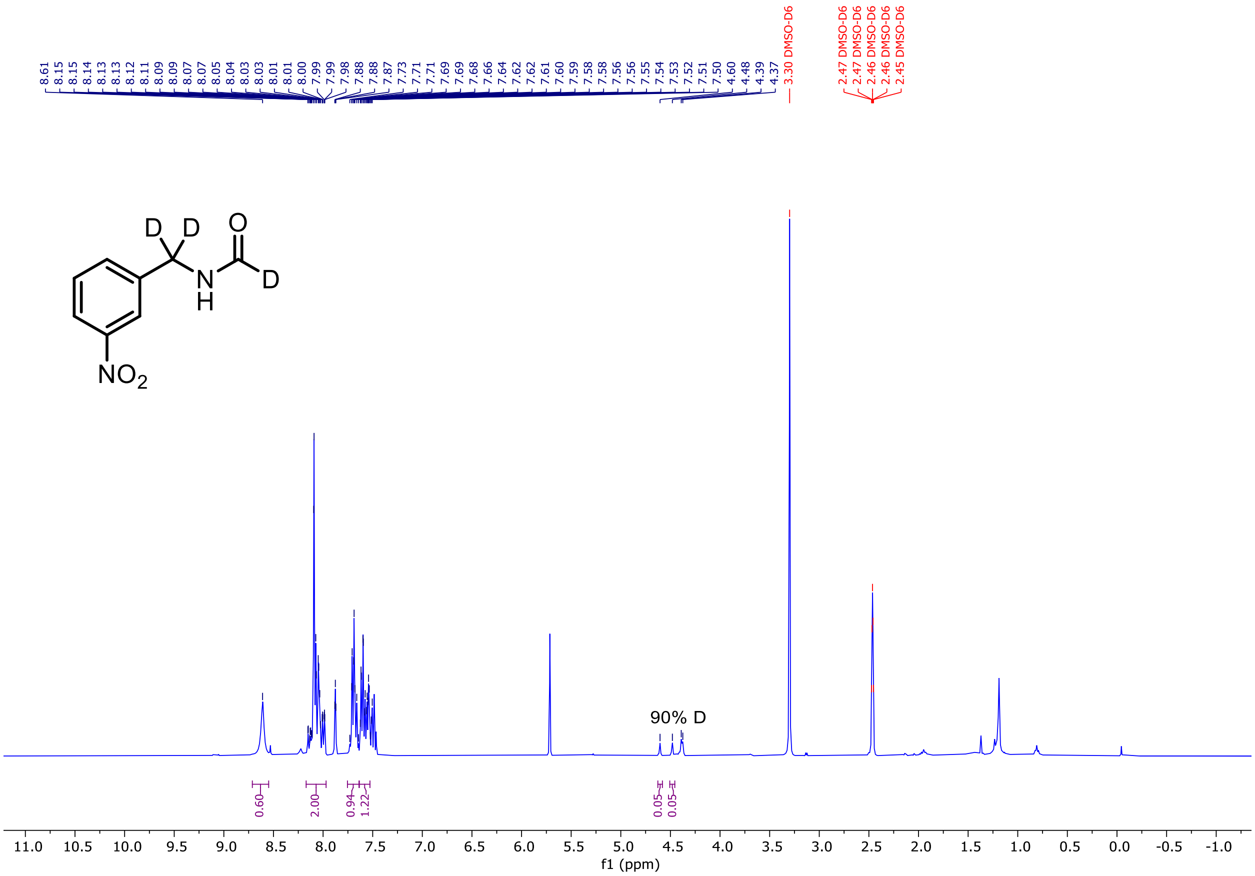

Figure S103. ^{1}H NMR (400 MHz, $CDCl_3$) of **9cb.**

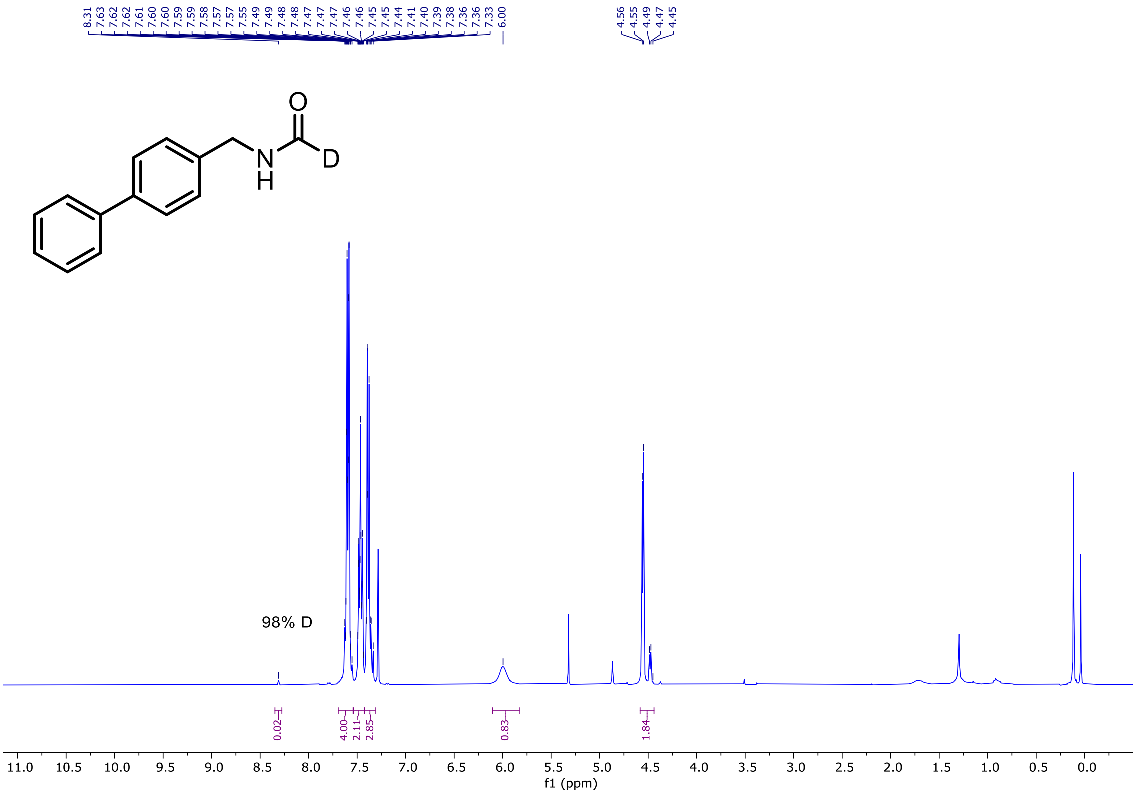

Figure S104. ^{1}H NMR (400 MHz, $CDCl_3$) of **9cb**.

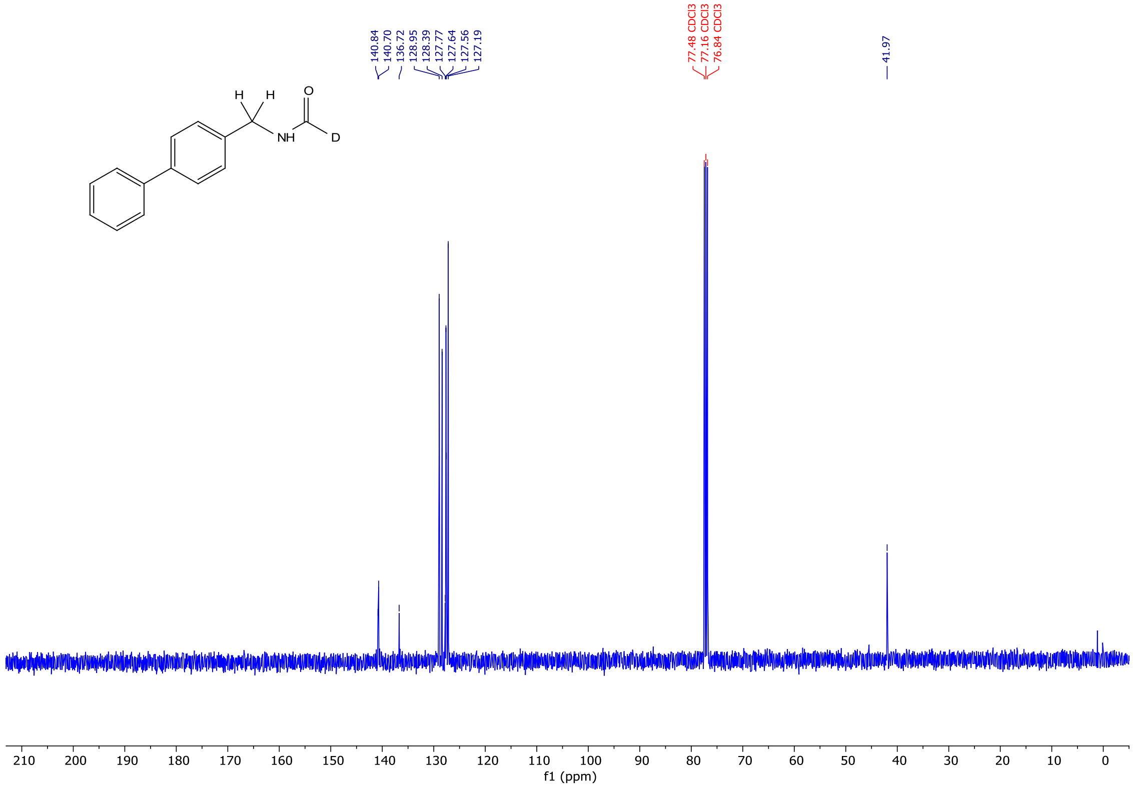

Figure S105. ^{13}C NMR (101 MHz, $CDCl_3$) of **9cb**.
